# Supplementary material for: SLC25A1 and ACLY maintain cytosolic acetyl-CoA and regulate ferroptosis susceptibility via FSP1 acetylation
Source: EMBO J. 2025 Jan 29;44(6):1641–62. doi: 10.1038/s44318-025-00369-5 (PMC11914110; doi:10.1038/s44318-025-00369-5)
Supplement: Supplementary file 1 — Appendix [file 44318_2025_369_MOESM1_ESM.pdf]

Appendix

Cytosolic citrate is maintained by SLC25A1 and ACLY and governs ferroptosis susceptibility of cancer cells via FSP1 acetylation

Wei Li[1, 6], Jing Han[2, 6], Bin Huang[1, 6], Tengteng Xu[1, 3, 6], Yihong Wan[1, 5], Dan Luo[1], Weiyao Kong[1], Ying Yu[1, 4], Lei Zhang[1, 3, 5], Yong Nian[4, *], Bo Chu[2, *], Chengqian Yin[1, 3, 7, *]

Appendix Table of Contents

Appendix Figure S1. Identification of SLC25A1 as a critical regulator of ferroptosis sensitivity (pages 2-4).

Appendix Figure S2. SLC25A1 is a critical regulator of ferroptosis sensitivity *in vivo* (pages 5-6).

Appendix Figure S3. Breakdown of citrate to acetyl-CoA by SLC25A1 and ACLY inhibits ferroptosis (pages 7-9).

Appendix Figure S4. Sodium acetate supplementation alleviates the enhanced ferroptosis induced by SLC25A1 or ACLY deletion (pages 10-12).

Appendix Figure S5. Analysis of lipidomics and transcriptomics following SLC25A1 depletion (pages 13-14).

Appendix Figure S6. FSP1 is acetylated at lysine 168, leading to increased protein stability (pages 15-16).

Appendix Figure S7. FSP1 acetylation and deacetylation is predominantly mediated by KAT2B/HDAC3 (pages 17-19).

Appendix Figure S8. SLC25A1 and ACLY regulate FSP1 stability (pages 20-21).

Appendix Figure S9. SLC25A1 and ACLY modulate ferroptosis sensitivity in a FSP1 acetylation-dependent manner (pages 22-24).

Appendix Figure S10. Targeting SLC25A1 and ACLY increases ferroptosis sensitivity *in vivo* (pages 25-26).

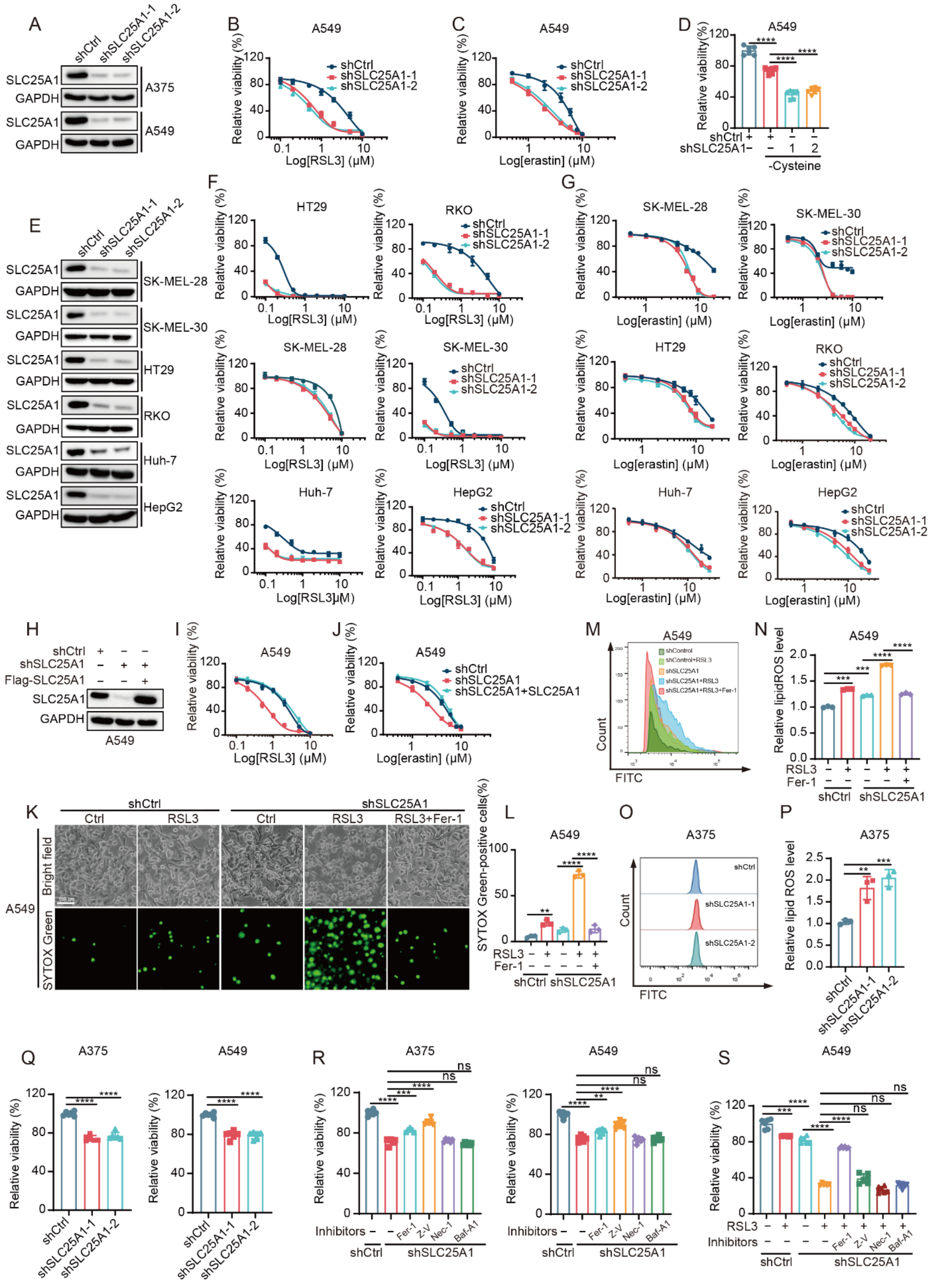
A
shCtrl
shSLC25A1-1
shSLC25A1-2
SLC25A1
GAPDH
A375
A549
B
A549
Relative viability(%)
Log[RSL3] (μM)
C
Log[erastin] (μM)
D
-Cysteine
E
SK-MEL-28
SK-MEL-30
HT29
RKO
Huh-7
HepG2
F
G
H
Flag-SLC25A1
shSLC25A1
I
J
shSLC25A1+SLC25A1
K
Ctrl
RSL3
RSL3+Fer-1
Bright field
SYTOX Green
L
SYTOX Green-positive cells(%)
Fer-1
M
shControl
shControl+RSL3
shSLC25A1
shSLC25A1+RSL3
shSLC25A1+RSL3+Fer-1
Count
FITC
N
Relative lipidROS level
O
P
Relative lipid ROS level
Q
R
Inhibitors
Z-V
Nec-1
Baf-A1
S
ns

Appendix Figure S1. Identification of SLC25A1 as a critical regulator of ferroptosis sensitivity

(A) Immunoblot confirming the knockdown of SLC25A1in shSLC25A1-A375/A549 cells.

(B-D) Measurement of cell viability in shCtrl versus shSLC25A1-A549 cells following treatment with increasing doses of RSL3 (B) or erastin (C) over 48 hours, or culture in cysteine-depleted medium for 24 hours (D). n = 3 for B-C, and n = 6 for D, n represents biological independent experiments. (D) Statistical analysis by were calculated using two-way ANOVA tests; mean + SD, *p* values from left to right: ****p = 4.37E-06, ****p = 7.86E-07, ****p = 6.72E-07.

(E) Immunoblot confirming the knockdown of SLC25A1 in shSLC25A1-SK-MEL-28/SK-MEL-30/HT29/RKO/Huh-7/HepG2 cells.

(F-G) Measurement of cell viability in shCtrl versus shSLC25A1-SK-MEL-28/SK-MEL-30/HT29/RKO/Huh-7/HepG2 cells following treatment with increasing doses of RSL3 (F) or erastin (G) over 48 hours. n = 3 for F-G, n represents biological independent experiments.

(H) Immunoblot of SLC25A1 protein levels in indicated cell groups. SLC25A1-knockdown A549 cells were reintroduced with a shRNA-resistant SLC25A1 construct (shSLC25A1 + SLC25A1-A549).

(I-J) Evaluation of cell viability in shCtrl, shSLC25A1, and shSLC25A1 + SLC25A1 + A549 cells after 48 hours of treatment with increasing concentrations of RSL3 (I) or erastin (J). n = 3 for I-J, n represents biological independent experiments.

(K-L) Detection of cell death using SYTOX Green in shCtrl and shSLC25A1-A549 cells treated as indicated for 48 hours. RSL3 at 0.5 μM, Fer-1 at 1 μM. Upper panel: phase-contrast images, lower panel: SYTOX Green staining for dead cells. Scale bars: left, 100 μm (K). Quantitative analysis of cell death percentage is presented, n = 3, n represents biological independent experiments. Statistical analysis by two-way ANOVA tests; mean + SD, *p* values from left to right: **p = 0.0016, ****p = 1.86E-05, ****p = 4.54E-05 (L).

(M-N) Detection of lipid peroxidation through flow cytometer in shCtrl and shSLC25A1-A549 cells under indicated treatments for 6 hours. RSL3 at 0.5 μM, Fer-1 at 1 μM (M). Quantification of lipid peroxidation percentage is presente. Quantification of lipid peroxidation percentage is presented. n = 3, n represents biological

independent experiments. Statistical analysis by two-way ANOVA tests; mean + SD, p values from left to right: ***p = 0.00018, ***p = 0.00064, ****p = 3.15E-07, ****p = 1.61E-06 (N).

(O-P) Detection of lipid peroxidation through flow cytometer in shCtrl and shSLC25A1-A375 cells (O). Quantification of lipid peroxidation percentage is presented, n = 3, n represents biological independent experiments. Statistical analysis by two-tailed, unpaired Student's t-test; mean + SD, p values from left to right: **p = 0.0075, ***p = 0.0010 (P).

(Q) Measurement of cell viability in shCtrl versus shSLC25A1-A375/A549 cells cultured for 48 hours. n = 6, n represents biological independent experiments. Statistical analysis by two-tailed, unpaired Student's t-test; mean + SD, p values from left to right: ****p = 1.32E-08, ****p = 1.40E-07 (A375 cells); ****p = 7.52E-07, ****p = 2.90E-07 (A549 cells).

(R) Comparison of cell viability between shCtrl and shSLC25A1-A375/A549 cells exposed to indicated treatments for 48 hours. Treatments include Fer-1 at 1 μM, Z-VAD-FMK (Z-V) at 10 μM, Necrostatin-1 (Nec-1) at 1 μM, and Bafilomycin A1 (Baf-A1) at 50 nM. n = 6, n represents biological independent experiments. Statistical analysis by paired Student's t-test; mean + SD, p values from left to right: ****p = 1.02E-08, ***p = 0.00035, ****p = 4.73E-07, ns: not significant (A375 cells); ****p = 8.23E-08, **p = 0.0024, ****p = 3.05E-05, ns: not significant (A549 cells).

(S) Measurement of cell viability in shCtrl and shSLC25A1-A549 cells subjected to indicated treatments for 48 hours. RSL3 at 0.5 μM, Fer-1 at 1 μM, Z-V at 10 μM, Necrostatin-1 (Nec-1) at 1 μM, and Baf-A1 at 50 nM. n = 6, n represents biological independent experiments. Statistical analysis by two-way ANOVA tests; mean + SD, p values from left to right: ***p = 0.00011, ****p = 4.51E-05, ****p = 2.63E-11, ****p = 2.66E-10, ns: not significant.

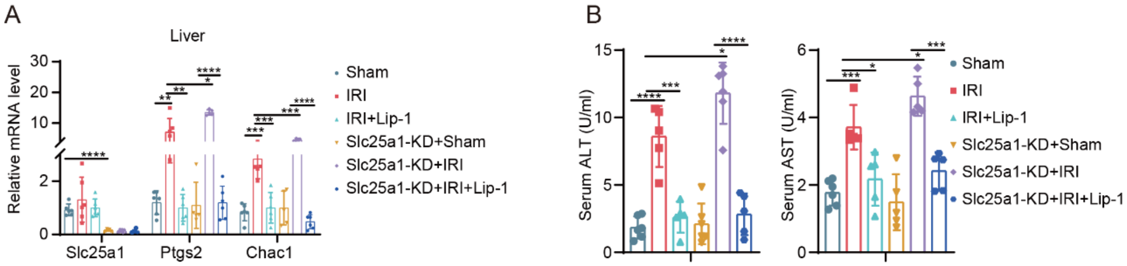
A
Liver
Relative mRNA level
Slc25a1
Ptgs2
Chac1
Sham
IRI
IRI+Lip-1
Slc25a1-KD+Sham
Slc25a1-KD+IRI
Slc25a1-KD+IRI+Lip-1
B
Serum ALT (U/ml)
Serum AST (U/ml)

Appendix Figure S2. SLC25A1 is a critical regulator of ferroptosis sensitivity *in vivo*

(A) Quantitative RT-PCR (qRT-PCR) analysis of mRNA expressions of Slc25a1, Ptgs2, and Chac1 from mice liver under the indicated treatment conditions. Treatments include Vehicle + Sham, Vehicle + IRI, Vehicle + IRI + Lip-1, Slc25a1-KD + Sham, Slc25a1-KD + IRI and Slc25a1-KD + IRI + Lip-1. n = 6, 6, 6, 5, 6, 6 (Slc25a1); 6, 6, 6, 5, 5, 6 (Ptgs2); 6, 6, 6, 5, 5, 6 (Chac1), n represents biological independent experiments. Statistical analysis by two-way ANOVA tests; mean + SD, *p* values from left to right: ****p = 3.27E-05 (Slc25a1); **p = 0.0039, **p = 0.0034, *p = 0.011, ****p = 6.05E-10 (Ptgs2); ***p = 0.00011, ***p = 0.00072, ***p = 0.0016, ****p = 2.41E-05 (Chac1).

(B) Measurement of ALT and AST levels in the serum from mice under the indicated treatment conditions. Treatments include Vehicle + Sham, Vehicle + IRI, Vehicle +IRI + Lip-1, Slc25a1-KD + Sham, Slc25a1-KD + IRI and Slc25a1-KD + IRI + Lip-1. n = 6, 5, 5, 6, 6, 5 (ALT); 6, 5, 5, 5, 5, 5 (AST), n represents biological independent experiments. Statistical analysis by two-way ANOVA tests; mean + SD, *p* values from left to right: ****p = 7.46E-05, ***p = 0.00074, *p = 0.044, ****p = 3.83E-05 (ALT), ***p = 0.00017, *p = 0.011, *p = 0.047, ***p = 0.00027 (AST).

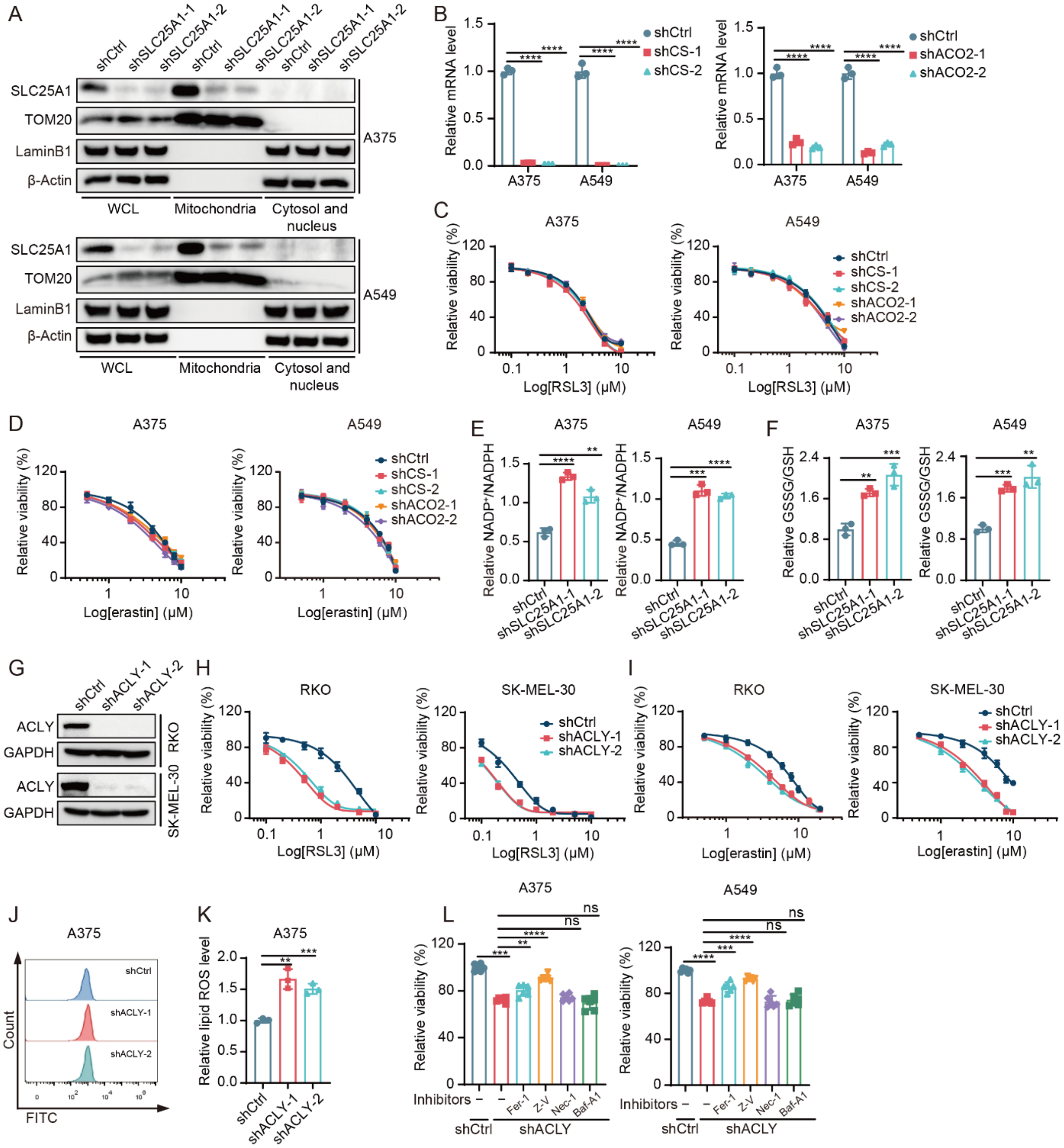
A
shCtrl
shSLC25A1-1
shSLC25A1-2
SLC25A1
TOM20
LaminB1
β-Actin
A375
WCL
Mitochondria
Cytosol and nucleus
A549
B
Relative mRNA level
shCtrl
shCS-1
shCS-2
shACO2-1
shACO2-2
C
Relative viability (%)
Log[RSL3] (μM)
D
Log[erastin] (μM)
E
Relative NADP+/NADPH
F
Relative GSSG/GSH
G
ACLY
GAPDH
RKO
SK-MEL-30
shACLY-1
shACLY-2
H
I
J
Count
FITC
K
Relative lipid ROS level
L
Inhibitors
Fer-1
Z-V
Nec-1
Baf-A1
shACLY
ns

Appendix Figure S3. Breakdown of citrate to acetyl-CoA by SLC25A1 and ACLY inhibits ferroptosis

(A) Immunoblot confirming mitochondrial and cytosolic fractionation in shSLC25A1-A375/A549 cells. TOM20 as a marker for mitochondria, β-actin and LaminB1 as a marker for cytosol and nucleus.

(B) qRT-PCR analysis of mRNA expressions of CS or ACO2 in shCtrl, shCS or shACO2-A375/A549 cells. n = 3, n represents biological independent experiments. Statistical analysis by two-way ANOVA tests; mean + SD, *p* values from left to right: ****p = 1.18E-06, ****p = 1.14E-06, ****p = 1.91E-05, ****p = 1.91E-05 (CS); ****p = 4.17E-05, ****p = 2.12E-05, ****p = 1.93E-05, ****p = 3.47E-05 (A549 cells).

(C-D) Measurement of cell viability in shCtrl, shCS, and shACO2-A375/A549 cells after 48 hours of treatment with increasing concentrations of RSL3 (C) or erastin (D). n = 3 for C-D, n represents biological independent experiments.

(E-F) Measurement of NADPH (E) and GSH (F) levels in shCtrl and shSLC25A1-A375/A549 cells. n = 3 for E-F, n represents biological independent experiments. Statistical analysis by paired Student's t-test; mean + SD, *p* values from left to right: ****p = 6.99E-05, **p = 0.0013, ***p = 0.00012, ****p = 2.59E-05 (E); ***p = 0.00077, **p = 0.0015, ***p = 0.00016, **p = 0.0016 (F).

(G) Immunoblot confirming the knockdown of ACLY in shACLY-RKO/SK-MEL-30 cells.

(H-I) Measurement of cell viability in shCtrl and shACLY-RKO/SK-MEL-30 cells after 48 hours of treatment with increasing concentrations of RSL3 (H) or erastin (I). n = 3 for H-I, n represents biological independent experiments.

(J-K) Detection of lipid peroxidation through flow cytometer in shCtrl and shACLY-A375 cells (J). Quantification of lipid peroxidation percentage is presented. n = 3, n represents biological independent experiments. Statistical analysis by two-tailed, unpaired Student's t-test; mean + SD, *p* values from left to right: **p = 0.0021, ***p = 0.00062 (K).

(L) Measurement of cell viability in shCtrl and shACLY-A375/A549 cells subjected to indicated treatments for 48 hours. Fer-1 at 1 μM, Z-V at 10 μM, Nec-1 at 1 μM, and Baf-A1 at 50 nM. n = 6, n represents biological independent experiments. Statistical analysis by paired Student's t-test; mean + SD, *p* values from left to right:

***p = 0.0003, **p = 0.0029, ****p = 1.44436E-07, ns: not significant (A375 cells); ****p = 6.62852E-07, ***p = 0.00013, ****p = 7.51E-09, ns: not significant (A549 cells).

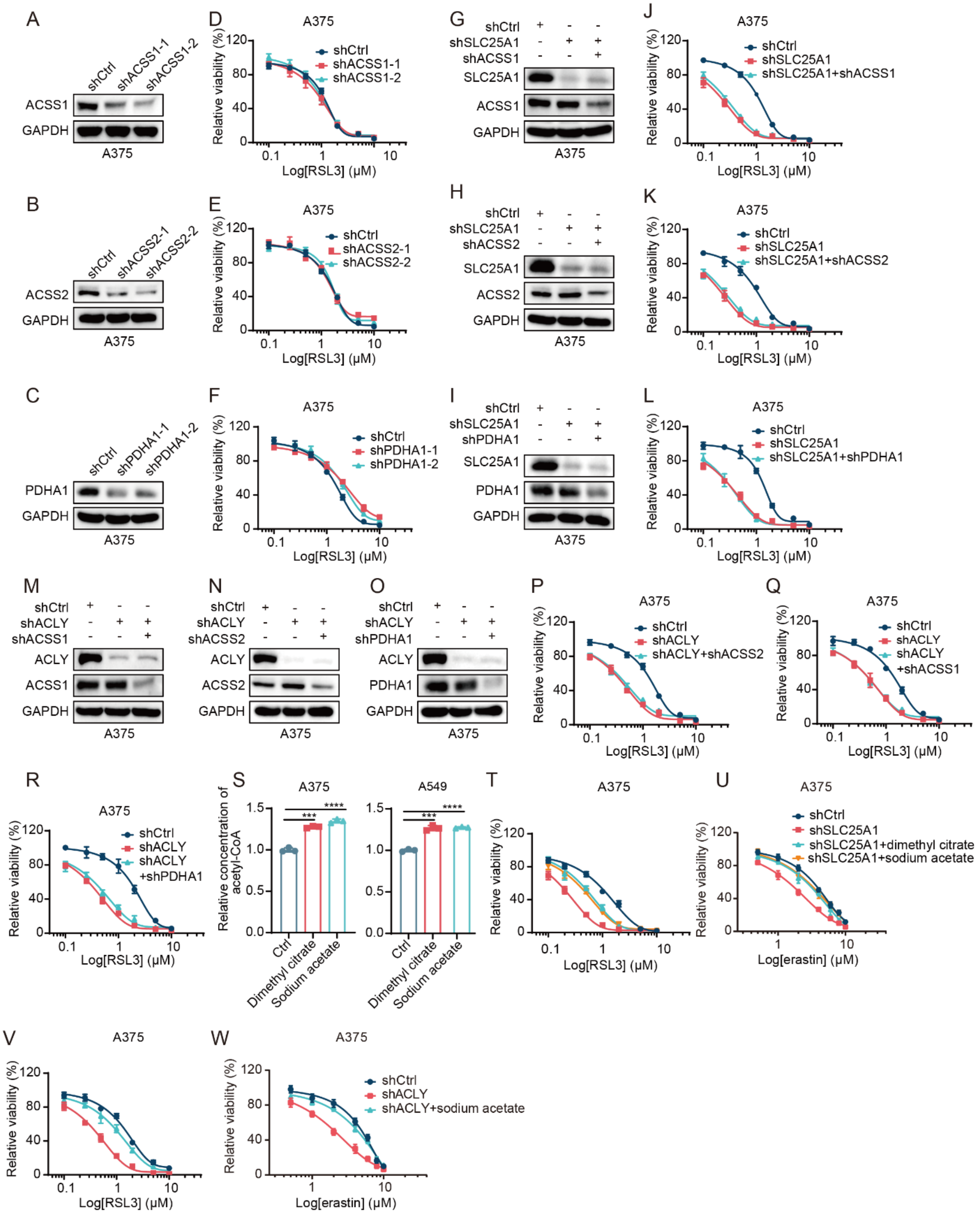
A
shCtrl
shACSS1-1
shACSS1-2
ACSS1
GAPDH
A375
D
A375
Relative viability (%)
Log[RSL3] (μM)
shCtrl
shACSS1-1
shACSS1-2
G
shCtrl
shSLC25A1
shACSS1
SLC25A1
ACSS1
GAPDH
A375
J
A375
shCtrl
shSLC25A1
shSLC25A1+shACSS1
B
shCtrl
shACSS2-1
shACSS2-2
ACSS2
GAPDH
A375
E
A375
shCtrl
shACSS2-1
shACSS2-2
H
shCtrl
shSLC25A1
shACSS2
SLC25A1
ACSS2
GAPDH
A375
K
A375
shCtrl
shSLC25A1
shSLC25A1+shACSS2
C
shCtrl
shPDHA1-1
shPDHA1-2
PDHA1
GAPDH
A375
F
A375
shCtrl
shPDHA1-1
shPDHA1-2
I
shCtrl
shSLC25A1
shPDHA1
SLC25A1
PDHA1
GAPDH
A375
L
A375
shCtrl
shSLC25A1
shSLC25A1+shPDHA1
M
shCtrl
shACLY
shACSS1
ACLY
ACSS1
GAPDH
A375
N
shCtrl
shACLY
shACSS2
ACLY
ACSS2
GAPDH
A375
O
shCtrl
shACLY
shPDHA1
ACLY
PDHA1
GAPDH
A375
P
A375
shCtrl
shACLY
shACLY+shACSS2
Q
A375
shCtrl
shACLY
shACLY
+shACSS1
R
A375
shCtrl
shACLY
shACLY
+shPDHA1
S
A375
A549
Relative concentration of acetyl-CoA
Ctrl
Dimethyl citrate
Sodium acetate
T
A375
Log[RSL3] (μM)
U
A375
shCtrl
shSLC25A1
shSLC25A1+dimethyl citrate
shSLC25A1+sodium acetate
Log[erastin] (μM)
V
A375
Log[RSL3] (μM)
W
A375
shCtrl
shACLY
shACLY+sodium acetate
Log[erastin] (μM)

Appendix Figure S4. Sodium acetate supplementation alleviates the enhanced ferroptosis induced by SLC25A1 or ACLY deletion

(A-C) Immunoblot confirming the knockdown of ACSS1 (A), ACSS2 (B) or PDHA1 (C) in shACSS1-, shACSS2- or shPDHA1-A375 cells.

(D-F) Measurement of cell viability in shCtrl, shACSS1 (D), shACSS2 (E) and shPDHA1-A37 cells (F) after 48 hours of treatment with increasing concentrations of RSL3. n = 3 for D-E, n represents biological independent experiments.

(G-I) Immunoblots of SLC25A1, ACSS1, ACSS2 and PDHA1 in A375 cells following knockdown of SLC25A1 with ACSS1 (G), ACSS2 (H) or PDHA1 (I).

(J-L) Cell viability assays of A375 cells expressing control shRNA (shCtrl), shRNA against SLC25A1 (shSLC25A1), shSLC25A1 with shRNA against ACSS1 (shSLC25A1 + shACSS1) (J), shRNA against ACSS2 (shSLC25A1 + shACSS2) (K), or shRNA against PDHA1 (shSLC25A1 + shPDHA1) (L) treated with increasing concentrations of RSL3 for 48 hours. n = 3 for J-L, n represents biological independent experiments.

(M-O) Immunoblots of ACLY, ACSS1, ACSS2 and PDHA1 in A375 cells following knockdown of ACLY with ACSS1 (M), ACSS2 (N) or PDHA1 (O).

(P-R) Cell viability assays of A375 cells expressing control shRNA (shCtrl), shRNA against ACLY (shACLY), shACLY with shRNA against ACSS1 (shACLY + shACSS1) (P), shRNA against ACSS2 (shACLY + shACSS2) (Q), or shRNA against PDHA1 (shACLY + shPDHA1) (R) treated with increasing concentrations of RSL3 for 48 hours. n = 3 for P-R, n represents biological independent experiments.

(S) Acetyl-CoA concentrations were measured in A375 and A549 cells treated with dimethyl citrate and sodium acetate for 48 hours. n = 3, n represents biological independent experiments. Statistical analysis by paired Student's t-test; mean + SD, *p* values from left to right: ****p* = 0.00027, *****p* = 8.39E-05 (A375 cells); ****p* = 0.00016, *****p* = 1.08E-05 (A549 cells).

(T-U) Cell viability assays for shCtrl, shSLC25A1-A375 cells treated with increasing concentrations of RSL3 (T) or erastin (U) supplemented with dimethyl citrate and sodium acetate for 48 hours. n = 3 for T-U, n represents

biological independent experiments.

(V-W) Cell viability assays for shCtrl, shACLY-A375 cells treated with increasing concentrations of RSL3 (V) or erastin (W) supplemented with sodium acetate for 48 hours. n = 3 for V-W, n represents biological independent experiments.

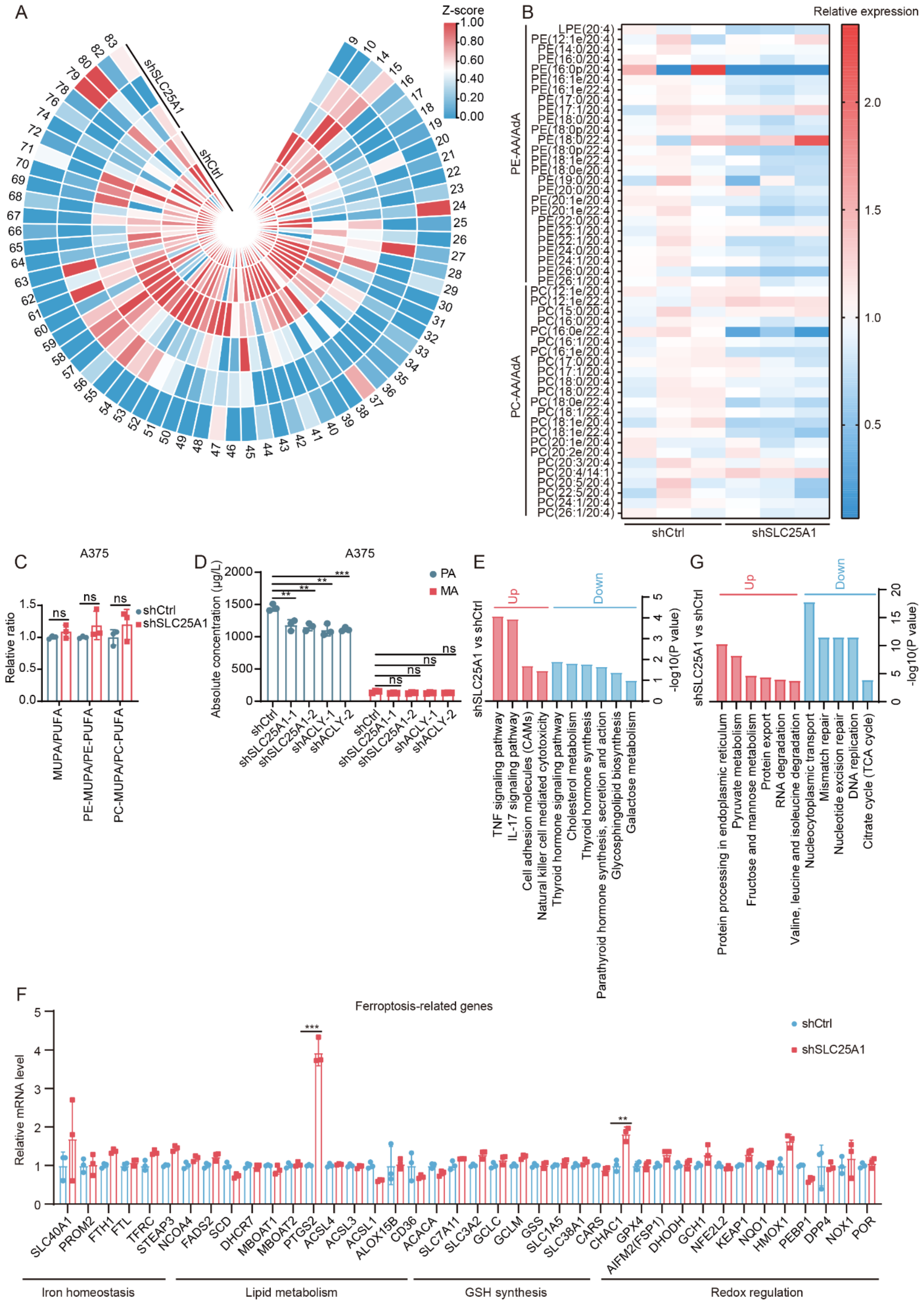
A
Z-score
shSLC25A1
shCtrl
B
Relative expression
PE-AA/AdA
PC-AA/AdA
shCtrl
shSLC25A1
C
A375
Relative ratio
shCtrl
shSLC25A1
MUPA/PUFA
PE-MUPA/PE-PUFA
PC-MUPA/PC-PUFA
D
A375
Absolute concentration (μg/L)
PA
MA
shCtrl
shSLC25A1-1
shSLC25A1-2
shACLY-1
shACLY-2
E
shSLC25A1 vs shCtrl
-log10(P value)
Up
Down
TNF signaling pathway
IL-17 signaling pathway
Cell adhesion molecules (CAMs)
Natural killer cell mediated cytotoxicity
Thyroid hormone signaling pathway
Cholesterol metabolism
Thyroid hormone synthesis
Parathyroid hormone synthesis, secretion and action
Glycosphingolipid biosynthesis
Galactose metabolism
G
Protein processing in endoplasmic reticulum
Pyruvate metabolism
Fructose and mannose metabolism
Protein export
RNA degradation
Valine, leucine and isoleucine degradation
Nucleocytoplasmic transport
Mismatch repair
Nucleotide excision repair
DNA replication
Citrate cycle (TCA cycle)
F
Ferroptosis-related genes
Relative mRNA level
Iron homeostasis
Lipid metabolism
GSH synthesis
Redox regulation

Appendix Figure S5. Analysis of lipidomics and transcriptomics following SLC25A1 depletion

(A) Heat map representing of the lipid content of the different carbon chains in shCtrl and shSLC25A1-A375 cells from lipidomics.

(B) Heat map representing of the PE-AA/AdA and PC-AA/AdA ratio of shSLC25A1 to shCtrl in A375 cells from lipidomics.

(C) Analysis of total MUFA/PUFA, PC-MUFA/PC-PUFA and PE-MUFA/PE-PUFA ratios in shCtrl and shSLC25A1-A375 cells from lipidomics. n = 3. n represents biological independent experiments.

(D) LC-MS/MS for the quantitative determination of myristic acid and palmitic acid in shCtrl, shSLC25A1 and shACLY-A375 cells. n = 3, n represents biological independent experiments. Statistical analysis by two-way ANOVA tests; mean + SD, p values from left to right: **$p = 0.0086$, **$p = 0.0027$, **$p = 0.0044$, ***$p = 0.00063$. ns, not significant.

(E) KEGG enrichment of differentially expressed genes in shSLC25A1-A375 cells compared to shCtrl-A375 cells. Upregulated genes are in red, and downregulated genes are in blue from RNA-seq.

(F) The relative expression of genes involved in ferroptosis in shCtrl and shSLC25A1-A375 cells from RNA-seq. n = 3, n represents biological independent experiments. Statistical analysis by two-way ANOVA tests; mean + SD, p values from left to right: ***$p = 0.00012$, **$p = 0.0035$.

(G) KEGG enrichment of differentially expressed proteins in shSLC25A1-A375 cells compared to shCtrl-A375 cells. Upregulated proteins are in red, and downregulated proteins are in blue from proteomics.

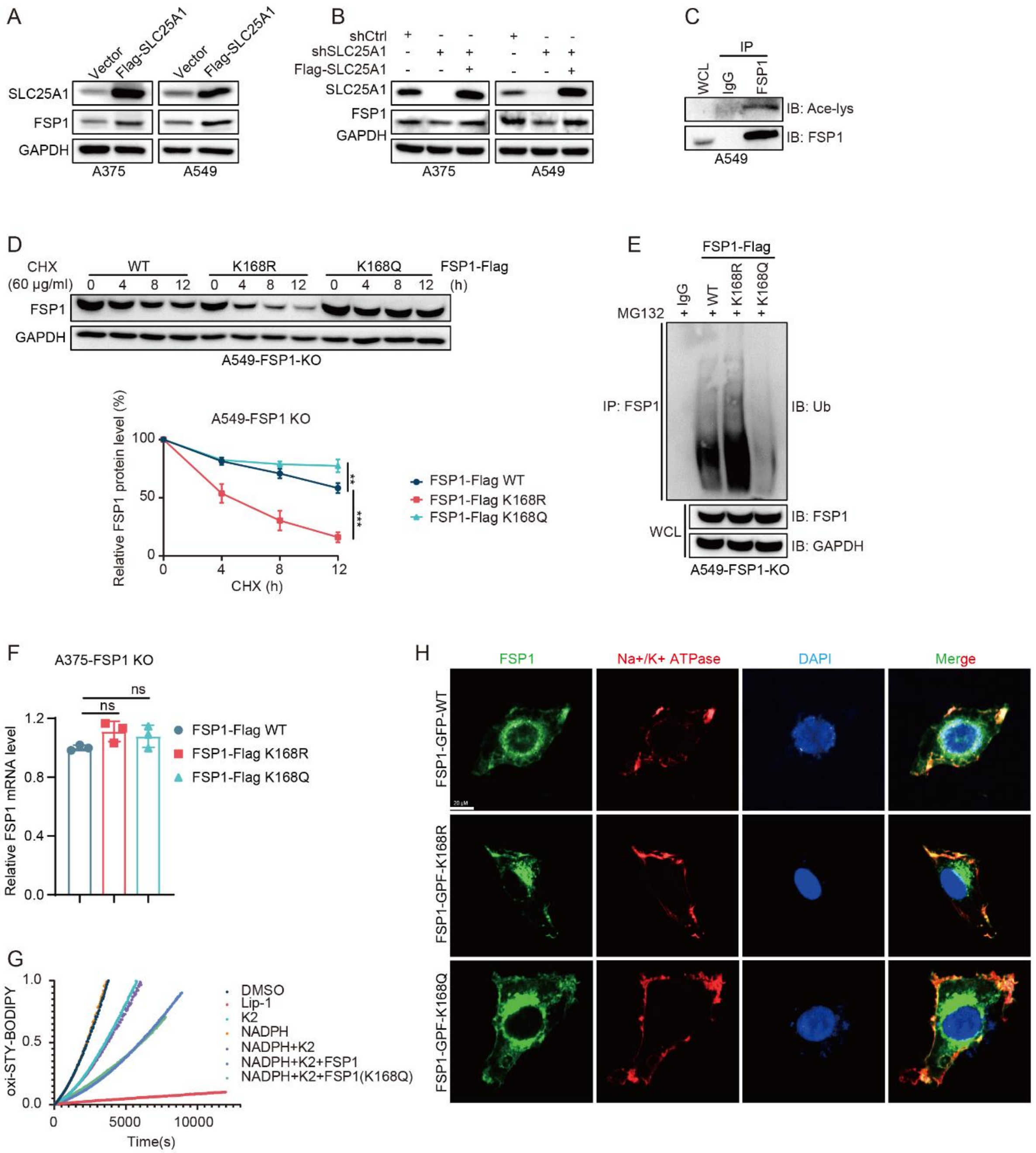

A
Vector
Flag-SLC25A1
SLC25A1
FSP1
GAPDH
A375
A549
B
shCtrl
shSLC25A1
Flag-SLC25A1
SLC25A1
FSP1
GAPDH
A375
A549
C
IP
WCL
IgG
FSP1
IB: Ace-lys
IB: FSP1
A549
D
CHX
(60 μg/ml)
WT
K168R
K168Q
FSP1-Flag
0 4 8 12 0 4 8 12 0 4 8 12 (h)
FSP1
GAPDH
A549-FSP1-KO
A549-FSP1 KO
Relative FSP1 protein level (%)
CHX (h)
FSP1-Flag WT
FSP1-Flag K168R
FSP1-Flag K168Q
E
FSP1-Flag
MG132
IgG
WT
K168R
K168Q
IP: FSP1
IB: Ub
WCL
IB: FSP1
IB: GAPDH
A549-FSP1-KO
F
A375-FSP1 KO
ns
Relative FSP1 mRNA level
FSP1-Flag WT
FSP1-Flag K168R
FSP1-Flag K168Q
G
oxi-STY-BODIPY
Time(s)
DMSO
Lip-1
K2
NADPH
NADPH+K2
NADPH+K2+FSP1
NADPH+K2+FSP1(K168Q)
H
FSP1
Na+/K+ ATPase
DAPI
Merge
FSP1-GFP-WT
FSP1-GPF-K168R
FSP1-GPF-K168Q

Appendix Figure S6. FSP1 is acetylated at lysine 168, leading to increased protein stability

(A) Immunoblot of SLC25A1 and FSP1 protein levels in indicated cell groups. Wild-type (WT) A375 and A549 cells overexpressing Flag-SLC25A1, with empty vector as a negative control.

(B) Immunoblot of SLC25A1 and FSP1 protein levels in indicated cell groups. SLC25A1-knockdown A375/A549 cells were reintroduced with a shRNA-resistant Flag-SLC25A1 construct.

(C) Immunoblot of acetylation of endogenous FSP1 in A549 cells. IgG was employed as a negative control.

(D) Immunoblot of FSP1 protein stability in A549-FSP1-KO cells re-expressing Flag-tagged FSP1 WT, K168R, or K168Q mutants, following 60 μg/mL cycloheximide (CHX) treatment over specified durations and quantification of FSP1 protein levels. n = 3, n represents biological independent experiments. Statistical analysis by two-tailed, unpaired Student's t-test; mean + SD, *p* values from left to right: $^{**}p = 0.0093$, $^{***}p = 0.00028$.

(E) Immunoblot of FSP1 ubiquitination in A549-FSP1-KO cells stably expressing Flag-tagged FSP1 WT, K168R, or K168Q mutants, following 10 μM MG132 treatment for 12 hours. IgG served as a negative control.

(F) qRT-PCR analysis of mRNA expressions of FSP1 in FSP1-knockout A375 cells re-expressing FSP1 WT/K168R/K168Q. n = 3, n represents biological independent experiments.

(G) Representative autoxidations of STY-BODIPY (1 μM)-embedded liposomes of egg PC lipids (1 mM, extruded to 100 nm) initiated by 0.2 mM DTUN and inhibited by DMSO, K2, NADPH, NADPH + K2, NADPH + K2 + recombinant human FSP1 proteins (rhFSP1) WT, NADPH + K2 + rhFSP1 K168Q or liproxstatin-1 (Lip-1).

(H) IF staining of GPF and Na+/K+ ATPase in HT1080 cells transduced with FSP1 WT, K168R or K168Q mutant-GFP lentivirus. GFP was displayed in green, and Na+/K+ ATPase was displayed in red. Scale bars: left, 20 μm.

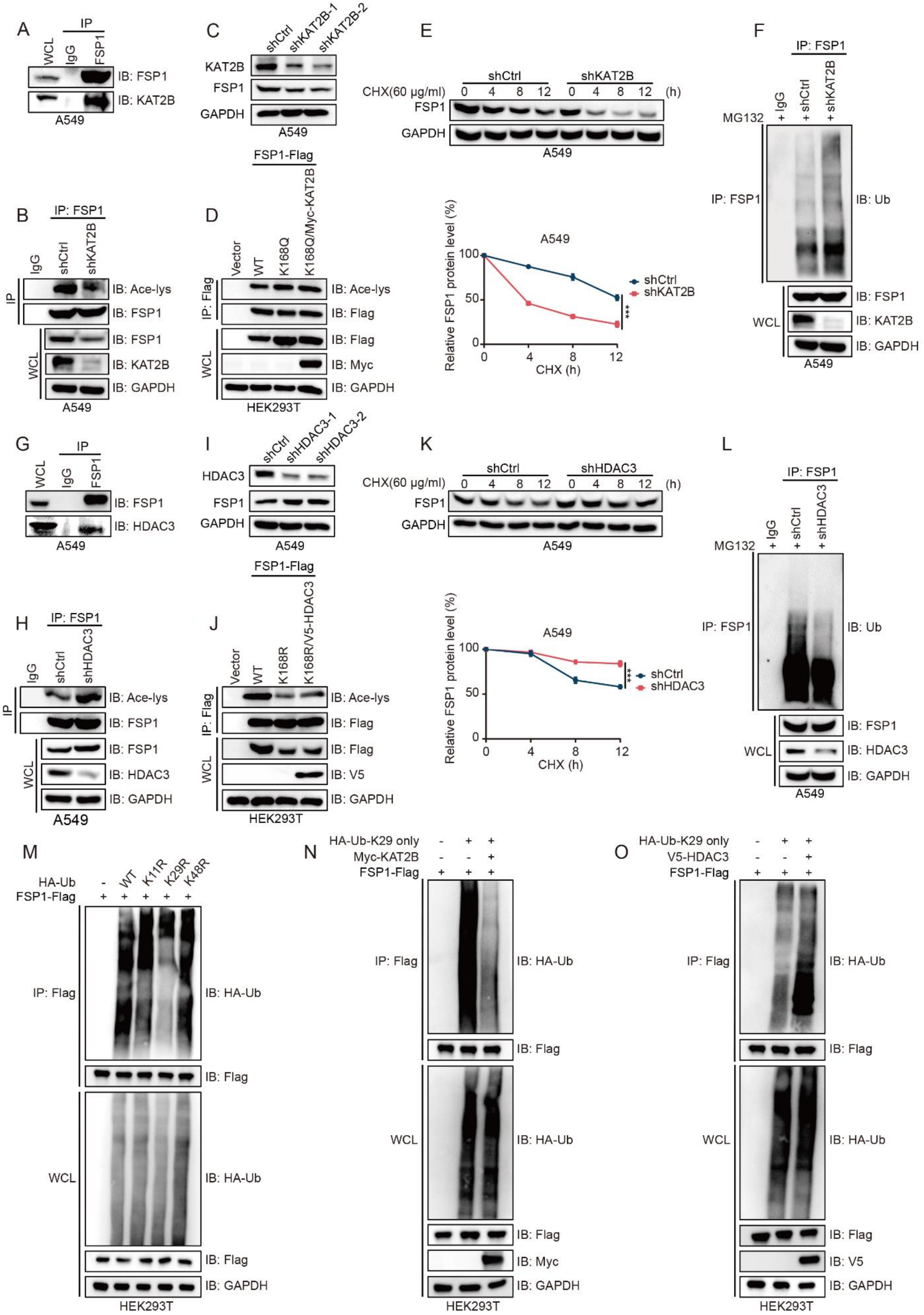
A
IP
WCL
IgG
FSP1
IB: FSP1
IB: KAT2B
A549
C
shCtrl
shKAT2B-1
shKAT2B-2
KAT2B
FSP1
GAPDH
A549
E
shCtrl
shKAT2B
CHX(60 μg/ml) 0 4 8 12 0 4 8 12 (h)
FSP1
GAPDH
A549
F
IP: FSP1
MG132
IgG
shCtrl
shKAT2B
IP: FSP1
IB: Ub
IB: FSP1
WCL
IB: KAT2B
IB: GAPDH
A549
B
IP: FSP1
IgG
shCtrl
shKAT2B
IP
IB: Ace-lys
IB: FSP1
WCL
IB: FSP1
IB: KAT2B
IB: GAPDH
A549
D
FSP1-Flag
Vector
WT
K168Q
K168Q/Myc-KAT2B
IP: Flag
IB: Ace-lys
IB: Flag
WCL
IB: Flag
IB: Myc
IB: GAPDH
HEK293T
Relative FSP1 protein level (%)
A549
shCtrl
shKAT2B
CHX (h)
G
IP
WCL
IgG
FSP1
IB: FSP1
IB: HDAC3
A549
I
shCtrl
shHDAC3-1
shHDAC3-2
HDAC3
FSP1
GAPDH
A549
K
shCtrl
shHDAC3
CHX(60 μg/ml) 0 4 8 12 0 4 8 12 (h)
FSP1
GAPDH
A549
L
IP: FSP1
MG132
IgG
shCtrl
shHDAC3
IP: FSP1
IB: Ub
IB: FSP1
WCL
IB: HDAC3
IB: GAPDH
A549
H
IP: FSP1
IgG
shCtrl
shHDAC3
IP
IB: Ace-lys
IB: FSP1
WCL
IB: FSP1
IB: HDAC3
IB: GAPDH
A549
J
FSP1-Flag
Vector
WT
K168R
K168R/V5-HDAC3
IP: Flag
IB: Ace-lys
IB: Flag
WCL
IB: Flag
IB: V5
IB: GAPDH
HEK293T
Relative FSP1 protein level (%)
A549
shCtrl
shHDAC3
CHX (h)
M
HA-Ub
WT
K11R
K29R
K48R
FSP1-Flag
IP: Flag
IB: HA-Ub
IB: Flag
WCL
IB: HA-Ub
IB: Flag
IB: GAPDH
HEK293T
N
HA-Ub-K29 only
Myc-KAT2B
FSP1-Flag
IP: Flag
IB: HA-Ub
IB: Flag
WCL
IB: HA-Ub
IB: Flag
IB: Myc
IB: GAPDH
HEK293T
O
HA-Ub-K29 only
V5-HDAC3
FSP1-Flag
IP: Flag
IB: HA-Ub
IB: Flag
WCL
IB: HA-Ub
IB: Flag
IB: V5
IB: GAPDH
HEK293T

Appendix Figure S7. FSP1 acetylation and deacetylation is predominantly mediated by KAT2B/HDAC3

(A) Immunoblot detecting endogenous interaction between FSP1 and KAT2B in A549 cells, with IgG as a negative control for IP.

(B) Immunoblot of FSP1 acetylation following KAT2B knockdown in A549 cells with IgG as a negative control.

(C) Immunoblot of FSP1 protein levels in shCtrl versus shKAT2B-A549 cells.

(D) Immunoblot of the acetylation of exogenous FSP1 in indicated cells. HEK293T cells transfected with Flag-tagged FSP1 WT, K168Q mutants, or both FSP1 K168Q-Flag and Myc-tagged KAT2B.

(E) Time-dependent FSP1 protein stability assessed via immunoblot in shCtrl and shKAT2B-A549 cells treated with CHX (60 μg/ml) for indicated durations and quantification of FSP1 protein levels. n = 3, n represents biological independent experiments. Statistical analysis by two-tailed, unpaired Student's t-test; mean + SD, ***p = 0.0003.

(F) Immunoblot of FSP1 ubiquitination in shCtrl and shKAT2B-A549 cells following treatment with MG132 (10 μM) for 8 hours, with IgG as a negative control.

(G) Immunoblot of endogenous interaction between FSP1 and HDAC3 in A549 cells, with IgG as a negative control.

(H) Immunoblot of FSP1 acetylation following HDAC3 knockdown in A549 cells with IgG as a negative control.

(I) Immunoblot of FSP1 protein levels in shCtrl versus shHDAC3-A549 cells.

(J) Immunoblot of the acetylation of exogenous FSP1 in indicated cells. HEK293T cells transfected with Flag-tagged FSP1 WT, K168R, or both FSP1 K168R-Flag and V5-tagged HDAC3.

(K) Immunoblot of FSP1 protein stability in shCtrl and shHDAC3-A549 cells treated with CHX (60 μg/ml) for indicated times and quantification of FSP1 protein levels. n = 3, n represents biological independent experiments. Statistical analysis by two-tailed, unpaired Student's t-test; mean + SD, ***p = 0.00032.

(L) Immunoblot of FSP1 ubiquitination in shCtrl and shHDAC3-A549 cells after MG132 treatment (10 μM, 12 hours), with IgG as a negative control.

(M) Immunoblot of FSP1-Flag ubiquitination in indicated cells. HEK293T cells cotransfected with plasmids expressing FSP1-Flag and HA-tagged wild-type Ub or HA-tagged mutant Ub (K11R, K29R and K48R).

(N-O) Immunoblot of FSP1-Flag ubiquitination in indicated cells. HEK293T cells transfected with FSP1 WT-Flag, K29-only Ub, Myc-tagged KAT2B (N) or V5-tagged HDAC3 (O).

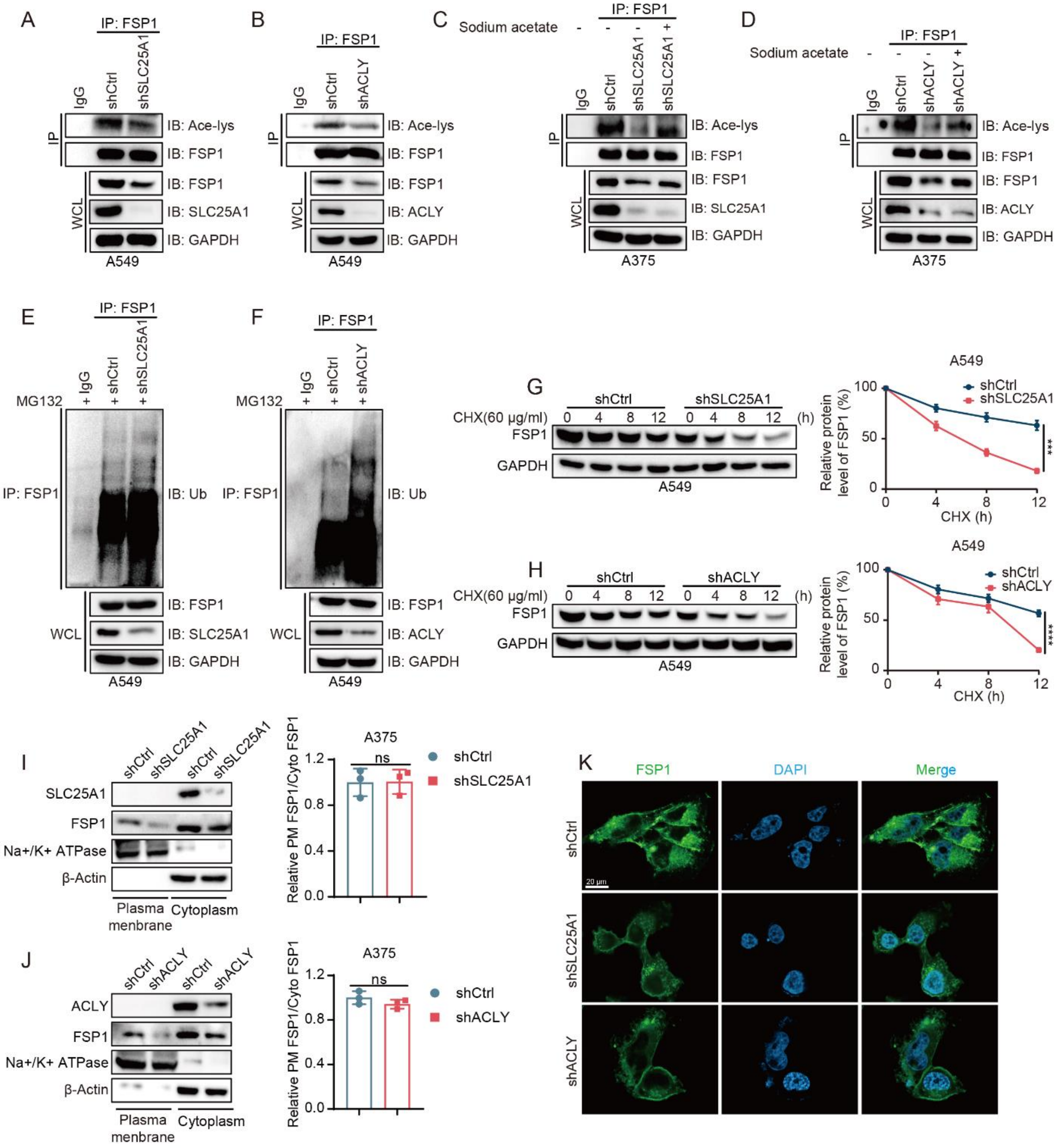

A
IP: FSP1
IgG
shCtrl
shSLC25A1
IP
IB: Ace-lys
IB: FSP1
WCL
IB: FSP1
IB: SLC25A1
IB: GAPDH
A549
B
IP: FSP1
IgG
shCtrl
shACLY
IB: Ace-lys
IB: FSP1
IB: ACLY
IB: GAPDH
A549
C
Sodium acetate
IP: FSP1
IB: SLC25A1
A375
D
Sodium acetate
shACLY
IB: ACLY
A375
E
MG132
IP: FSP1
IB: Ub
WCL
F
G
CHX(60 μg/ml)
shCtrl
shSLC25A1
(h)
FSP1
GAPDH
A549
Relative protein level of FSP1 (%)
CHX (h)
H
shACLY
I
SLC25A1
FSP1
Na+/K+ ATPase
β-Actin
Plasma menbrane
Cytoplasm
A375
ns
Relative PM FSP1/Cyto FSP1
J
ACLY
K
FSP1
DAPI
Merge
20 μm

Appendix Figure S8. SLC25A1 and ACLY regulate FSP1 stability

(A-B) Immunoblot of FSP1 acetylation levels in A549 cells stably transfected with lentiviral shRNA targeting SLC25A1 (A), ACLY (B), or a control shRNA. IgG served as a negative control.

(C-D) Immunoblot of FSP1 acetylation levels in shCtrl, shSLC25A1(C) or shACLY-A375 cells (D) supplemented with sodium acetate for 48 hours.

(E-F) Immunoblotting of ubiquitination of FSP1 in A549 cells stably expressing shRNA against SLC25A1 (E), ACLY (F), or control shRNA, following 10 µM MG132 treatment for 12 hours. IgG served as a negative control.

(G-H) Immunoblots of FSP1 protein levels in A549 cells with stable shRNA-mediated knockdown of SLC25A1 (G), ACLY (H), or control shRNA, treated with CHX (60 µg/ml) for the indicated durations and quantification of FSP1 protein levels. n = 3 for E-F, n represents biological independent experiments. Statistical analysis by two-tailed, unpaired Student's t-test; mean + SD, ****p* = 0.00012 (G), *****p* = 7.74E-05 (H).

(I-J) Immunoblots of FSP1 protein levels at the plasma membrane (PM) and cytoplasm (Cyto) in shCtrl, shSLC25A1(I) and shACLY-A375 (J) cells, and quantification of PM FSP1/Cyto FSP1. n = 3 for G-H, n represents biological independent experiments.

(K) IF staining of FSP1 in shCtrl, shSLC25A1 and shACLY-A375 cells. Scale bars: left, 20 µm.

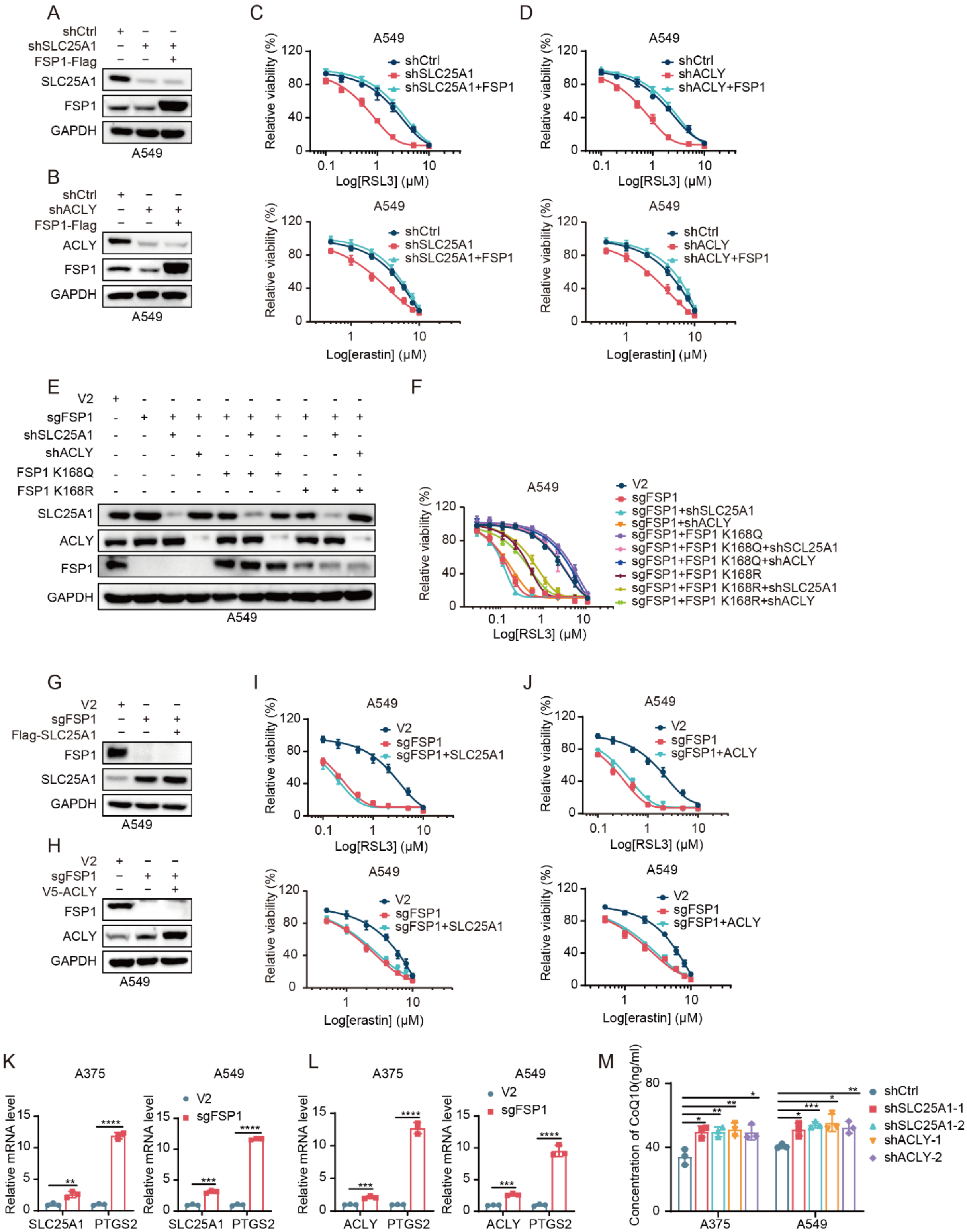
A
shCtrl
shSLC25A1
FSP1-Flag
SLC25A1
FSP1
GAPDH
A549
B
shCtrl
shACLY
FSP1-Flag
ACLY
FSP1
GAPDH
A549
C
A549
shCtrl
shSLC25A1
shSLC25A1+FSP1
Relative viability (%)
Log[RSL3] (μM)
Log[erastin] (μM)
D
A549
shCtrl
shACLY
shACLY+FSP1
Relative viability (%)
Log[RSL3] (μM)
Log[erastin] (μM)
E
V2
sgFSP1
shSLC25A1
shACLY
FSP1 K168Q
FSP1 K168R
SLC25A1
ACLY
FSP1
GAPDH
A549
F
A549
V2
sgFSP1
sgFSP1+shSLC25A1
sgFSP1+shACLY
sgFSP1+FSP1 K168Q
sgFSP1+FSP1 K168Q+shSCL25A1
sgFSP1+FSP1 K168Q+shACLY
sgFSP1+FSP1 K168R
sgFSP1+FSP1 K168R+shSLC25A1
sgFSP1+FSP1 K168R+shACLY
Relative viability (%)
Log[RSL3] (μM)
G
V2
sgFSP1
Flag-SLC25A1
FSP1
SLC25A1
GAPDH
A549
H
V2
sgFSP1
V5-ACLY
FSP1
ACLY
GAPDH
A549
I
A549
V2
sgFSP1
sgFSP1+SLC25A1
Relative viability (%)
Log[RSL3] (μM)
Log[erastin] (μM)
J
A549
V2
sgFSP1
sgFSP1+ACLY
Relative viability (%)
Log[RSL3] (μM)
Log[erastin] (μM)
K
A375
A549
V2
sgFSP1
Relative mRNA level
SLC25A1
PTGS2
L
A375
A549
V2
sgFSP1
Relative mRNA level
ACLY
PTGS2
M
Concentration of CoQ10(ng/ml)
shCtrl
shSLC25A1-1
shSLC25A1-2
shACLY-1
shACLY-2
A375
A549

Appendix Figure S9. SLC25A1 and ACLY modulate ferroptosis sensitivity in a FSP1 acetylation-dependent manner

(A-B) Immunoblots of SLC25A1, ACLY, and FSP1 in A549 cells following knockdown of SLC25A1 (A) or ACLY (B) and overexpression of FSP1.

(C-D) Cell viability assays of A549 cells expressing control shRNA (shCtrl), shRNA against SLC25A1 (shSLC25A1), shSLC25A1 with FSP1 overexpression (shSLC25A1 + FSP1), shRNA against ACLY (shACLY), and shACLY with FSP1 overexpression (shACLY + FSP1) treated with increasing concentrations of RSL3 (C) or erastin (D) for 48 hours. n = 3 for C-D, n represents biological independent experiments.

(E) Immunoblots of SLC25A1, ACLY, and FSP1 in A549 cells in indicated cell groups. FSP1-knockout A549 cells were introduced with a shRNA-against SLC25A1or ACLY (sgFSP + shSLC25A1, sgFSP1 + shACLY), or sgRNA-resistant FSP1 K168Q or FSP1K168R (sgFSP1 + FSP1 K168Q, sgFSP1 + FSP1 K168R, sgFSP1 + FSP1 K168Q + shSLC25A1, sgFSP1+ FSP1 K168Q + shACLY, sgFSP1 + FSP1 K168R + shSLC25A1, sgFSP1 + FSP1 K168R + shACLY).

(F) Evaluation of cell viability in V2, sgFSP1, sgFSP1 + shSLC25A1, sgFSP1 + shACLY, sgFSP1 + FSP1 K168Q, sgFSP1 + FSP1 K168Q + shSLC25A1, sgFSP1+ FSP1 K168Q + shACLY, sgFSP1 + FSP1 K168R, sgFSP1 + FSP1 K168R + shSLC25A1 and sgFSP1 + FSP1 K168R + shACLY-A549 cells after 48 hours of treatment with increasing concentrations of RSL3. n = 3, n represents biological independent experiments.

(G-H) Immunoblot of FSP1, SLC25A1, and ACLY levels in FSP1-knockout A549 cells overexpressing SLC25A1 (G) or ACLY (H).

(I-J) Cell viability assays of A549 cells transfected with V2, sgRNA targeting FSP1 (sgFSP1), sgFSP1 with SLC25A1 overexpression (sgFSP1 + SLC25A1), and sgFSP1 with ACLY overexpression (sgFSP1 + ACLY) exposed to increasing concentrations of RSL3 (I) or erastin (J) for 48 hours. n = 3 for I-J, n represents biological independent experiments.

(K-L) qRT-PCR analysis of mRNA expressions of SLC25A1 (K), ACLY (L) and PTGS2 in V2 and sgFSP1-A375/A549 cells. n = 3 for K-L, n represents biological independent experiments. Statistical analysis by two-way ANOVA tests; mean + SD, *p* values from left to right: ***p* = 0.0073, *****p* = 4.07E-06, ****p* = 0.00012,

****p = 7.69E-08 (K); ***p = 0.00064, ****p = 1.93E-05, ***p = 0.00011, ****p = 7.92E-05 (L).

(M) Measurement of cellular CoQ10 concentrations. n = 3, n represents biological independent experiments. Statistical analysis by two-way ANOVA tests; mean + SD, p values from left to right: *p = 0.011, **p = 0.0099, **p = 0.0098, *p = 0.018 (A375 cells), *p = 0.013, ***p = 0.00064, *p = 0.011, **p = 0.007 (A549 cells).

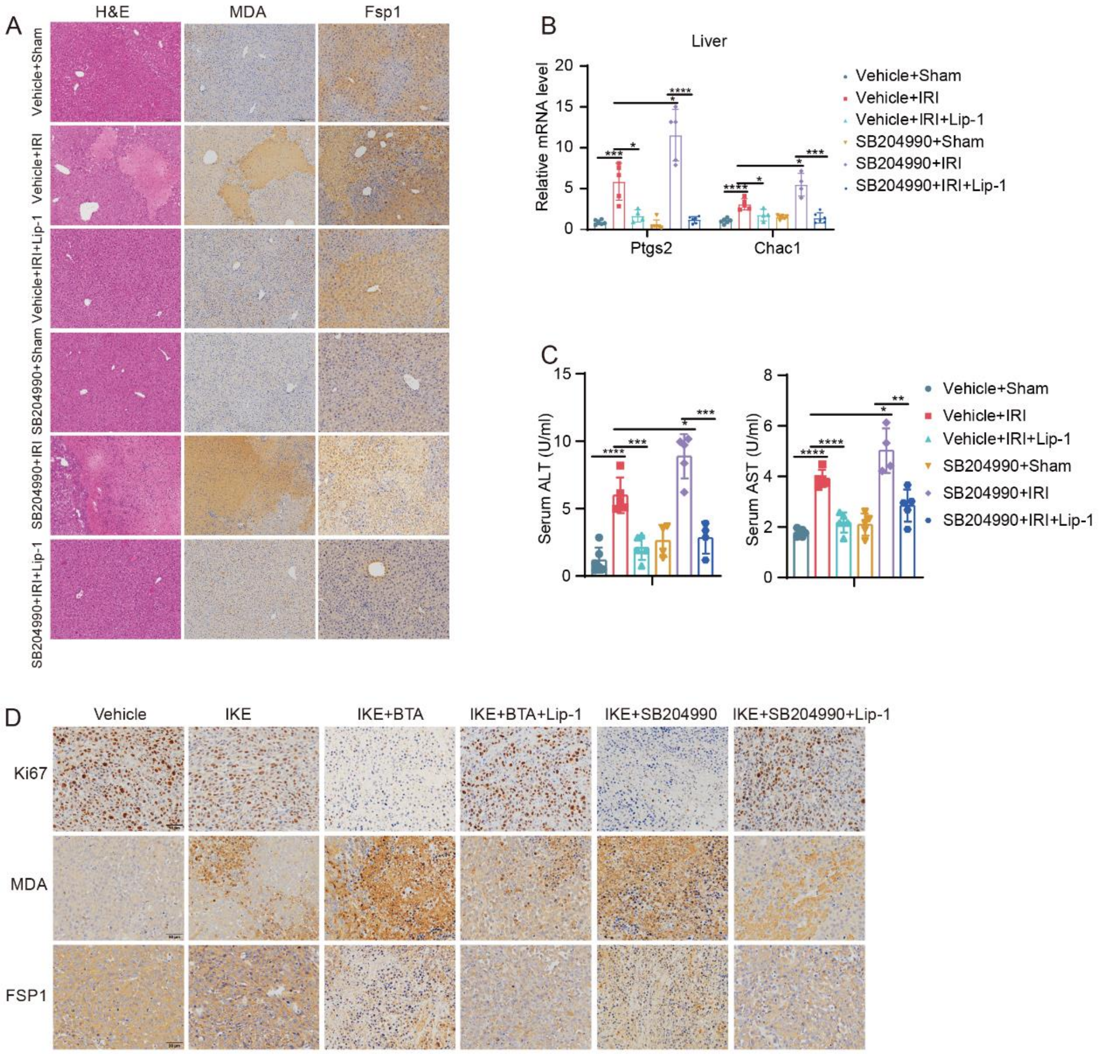
A
H&E
MDA
Fsp1
Vehicle+Sham
Vehicle+IRI
Vehicle+IRI+Lip-1
SB204990+Sham
SB204990+IRI
SB204990+IRI+Lip-1
B
Liver
Relative mRNA level
Ptgs2
Chac1
Vehicle+Sham
Vehicle+IRI
Vehicle+IRI+Lip-1
SB204990+Sham
SB204990+IRI
SB204990+IRI+Lip-1
C
Serum ALT (U/ml)
Serum AST (U/ml)
D
Vehicle
IKE
IKE+BTA
IKE+BTA+Lip-1
IKE+SB204990
IKE+SB204990+Lip-1
Ki67
MDA
FSP1

Appendix Figure S10. Targeting SLC25A1 and ACLY increases ferroptosis sensitivity *in vivo*

(A) Mice were injected with vehicle, SB204990 (50 mg/kg) for three consecutive days. Lip-1 (10 mg/kg) was injected half an hour before ischemia reperfusion (IRI) or sham-treatment. Representative images showing H&E staining, MDA and Fap1 immunohistochemical staining of livers from mice under the indicated treatment conditions. Scale bars: right, 200 nm. The experiment was repeated three times.

(B) qRT-PCR analysis of mRNA expressions of Ptgs2, and Chac1 from mice liver under the indicated treatment conditions. Treatments include Vehicle + Sham, Vehicle + IRI, Vehicle + IRI + Lip-1, SB204990 + Sham, SB204990 + IRI and SB204990 + IRI + Lip-1. n = 6, 5, 4, 6, 5, 6 (Ptgs2); 6, 5, 4, 6, 4, 6 (Chac1), n represents biological independent experiments. Statistical analysis by two-way ANOVA tests; mean + SD, p values from left to right: ****p* = 0.00045, **p* = 0.0102, **p* = 0.012, *****p* = 2.15E-05 (Ptgs2); *****p* = 9.85E-05, **p* = 0.0257, **p* = 0.011, ****p* = 0.0002 (Chac1).

(C) Measurement of ALT and AST levels in the serum from mice under the indicated treatment conditions. Treatments include Vehicle + Sham, Vehicle + IRI, Vehicle + IRI + Lip-1, SB204990 + Sham, SB204990 + IRI and SB204990 + IRI + Lip-1. n = 6, 5, 5, 4, 5, 4 (ALT); 6, 5, 5, 5, 4, 5 (AST), n represents biological independent experiments. Statistical analysis by two-way ANOVA tests; mean + SD, p values from left to right: *****p* = 5.61E-05, ****p* = 0.00059, **p* = 0.015, ****p* = 0.00011 (ALT), *****p* = 1.88E-07, *****p* = 6.93E-05, **p* = 0.035, ***p* = 0.0035 (AST).

(D) Representative images showing Ki67, MDA and FSP1 immunohistochemical staining of tumors from mice under the indicated treatment conditions. Treatments include Vehicle, IKE, SB204990 + IKE, SB204990 + IKE + Lip-1. Scale bars: right, 50 μm. The experiment was repeated three times.
