# Supplementary figures and images for: SLC25A1 and ACLY maintain cytosolic acetyl-CoA and regulate ferroptosis susceptibility via FSP1 acetylation
Source: EMBO J. 2025 Jan 29;44(6):1641–62. doi: 10.1038/s44318-025-00369-5 (PMC11914110; doi:10.1038/s44318-025-00369-5)

## Slide 1
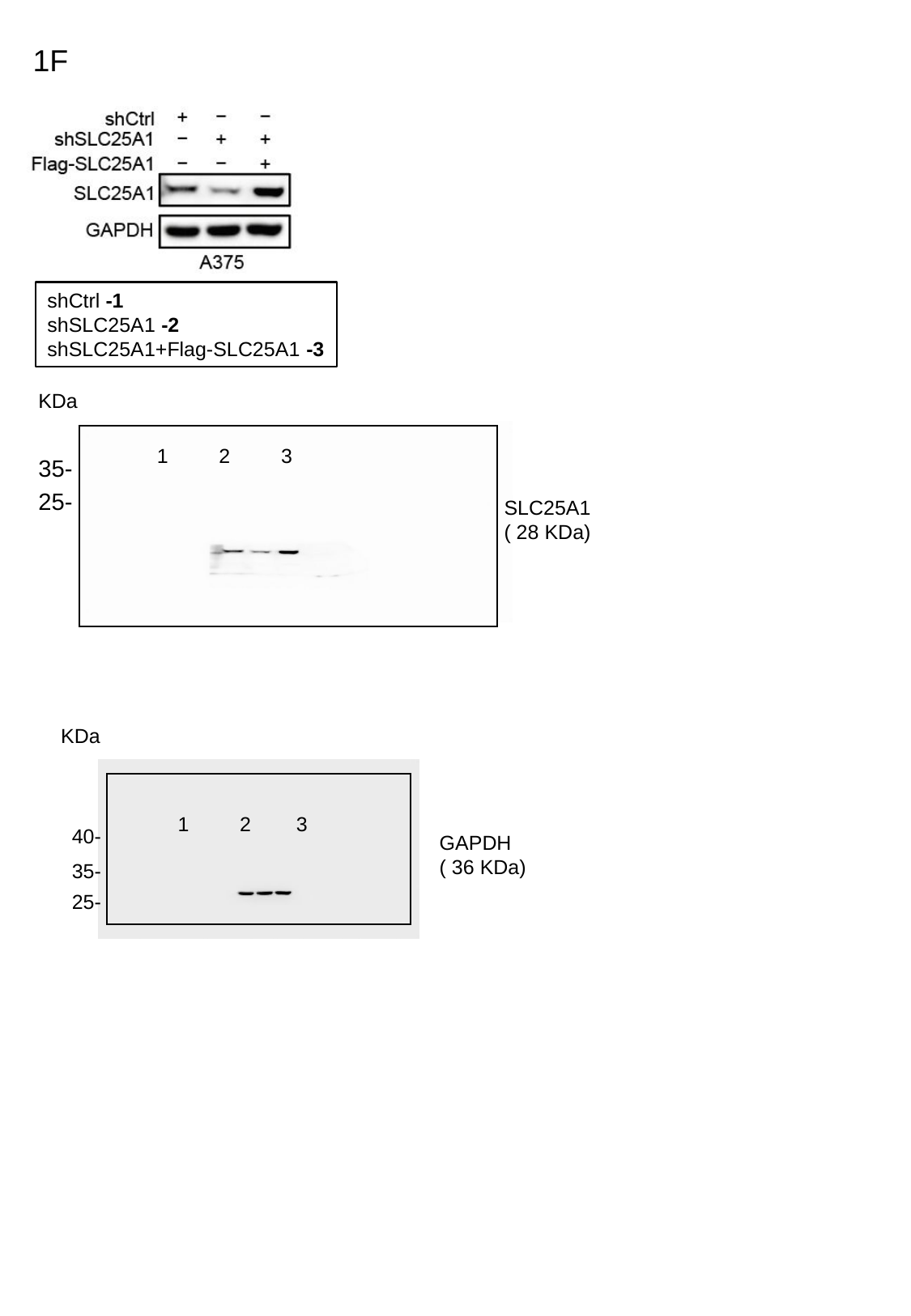

1F
shCtrl -1
shSLC25A1 -2
shSLC25A1+Flag-SLC25A1 -3
KDa
 1 2 3
35-
25-
SLC25A1
( 28 KDa)
KDa
 1 2 3
40-
GAPDH
( 36 KDa)
35-
25-

Supplement: Supplementary file 3 — Source data Fig. 1 [file 44318_2025_369_MOESM3_ESM.zip › Figure 1/1F/1F-A375-WB.pptx]

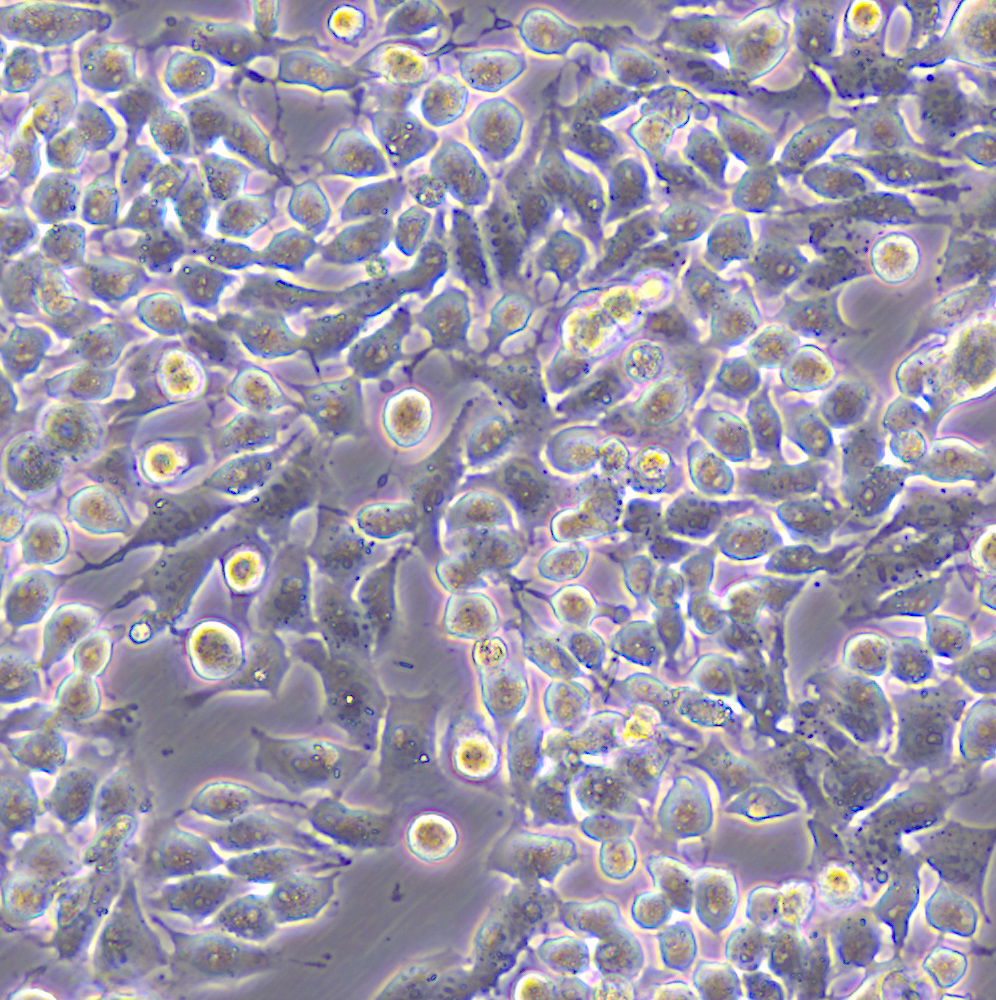

Supplement: Supplementary file 3 — Source data Fig. 1 [file 44318_2025_369_MOESM3_ESM.zip › Figure 1/1I/Bright filed/shCtrl+RSL3.png]

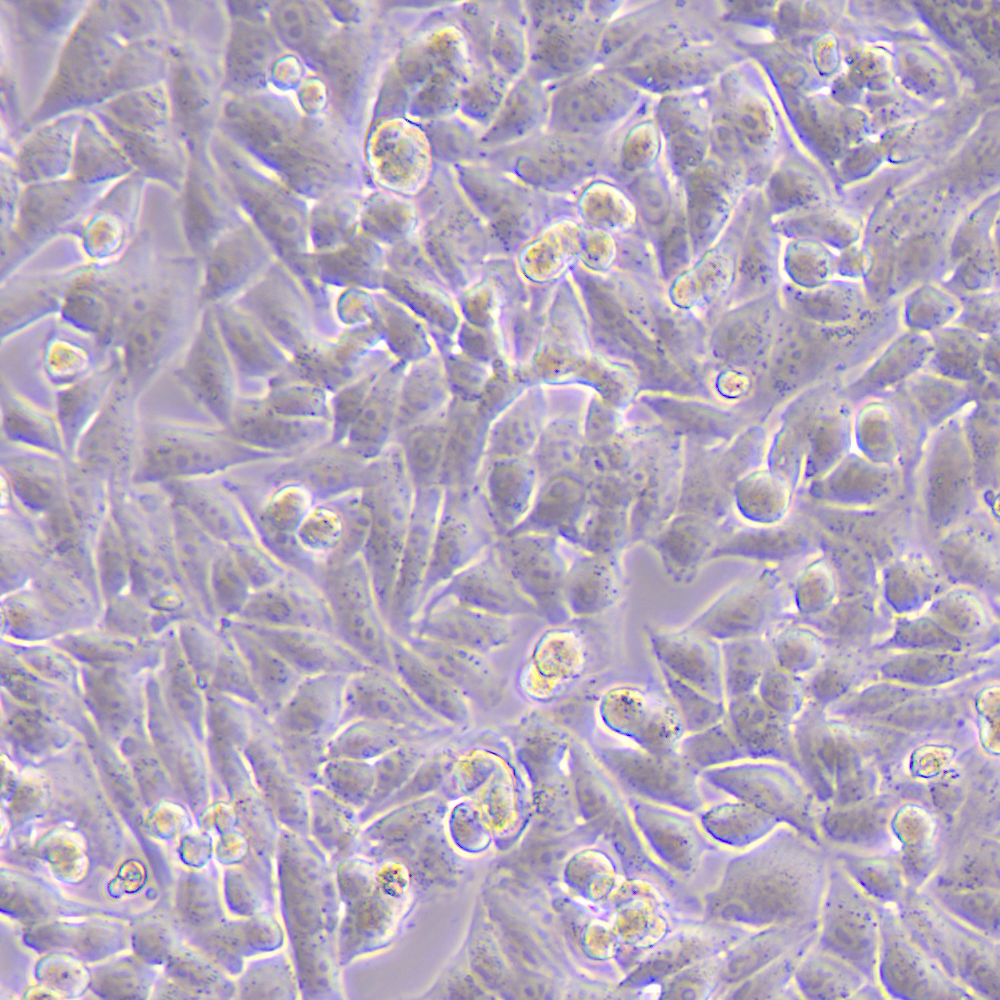

Supplement: Supplementary file 3 — Source data Fig. 1 [file 44318_2025_369_MOESM3_ESM.zip › Figure 1/1I/Bright filed/shCtrl.png]

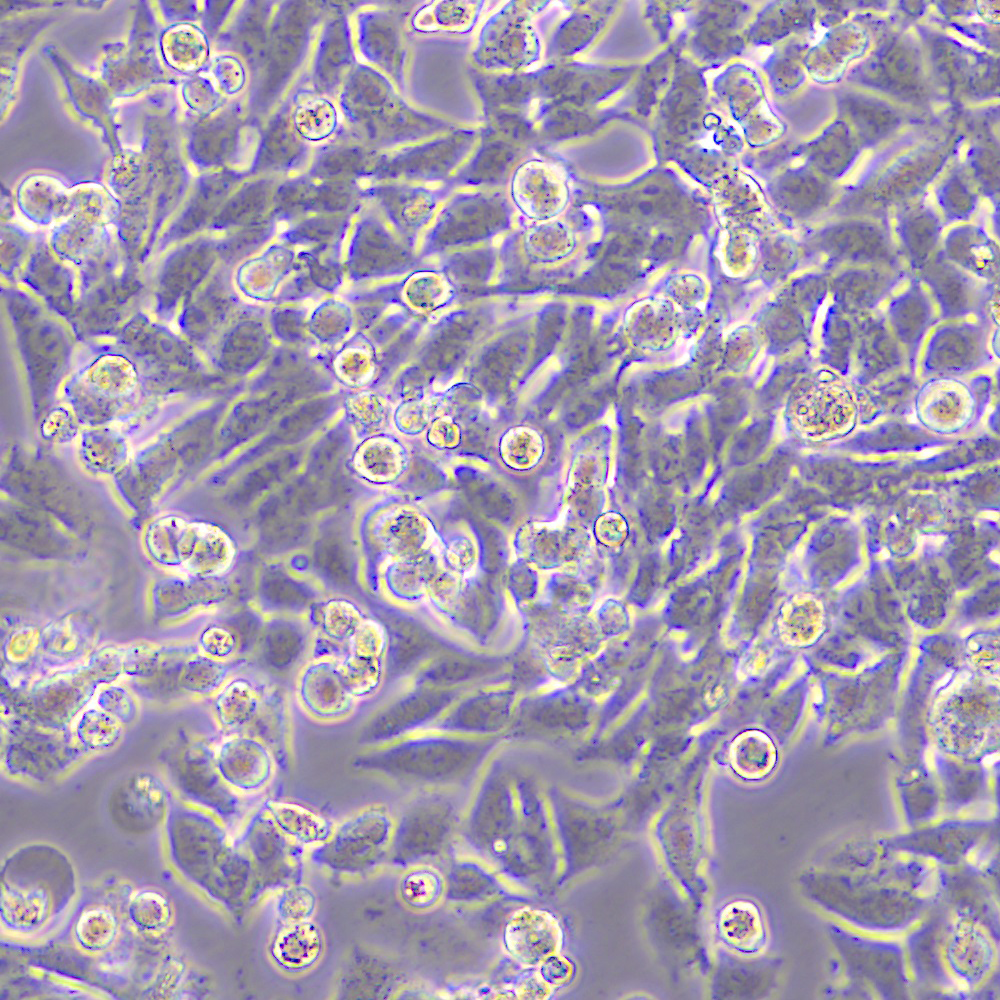

Supplement: Supplementary file 3 — Source data Fig. 1 [file 44318_2025_369_MOESM3_ESM.zip › Figure 1/1I/Bright filed/shSLC25A1+RSL3+Fer-1.png]

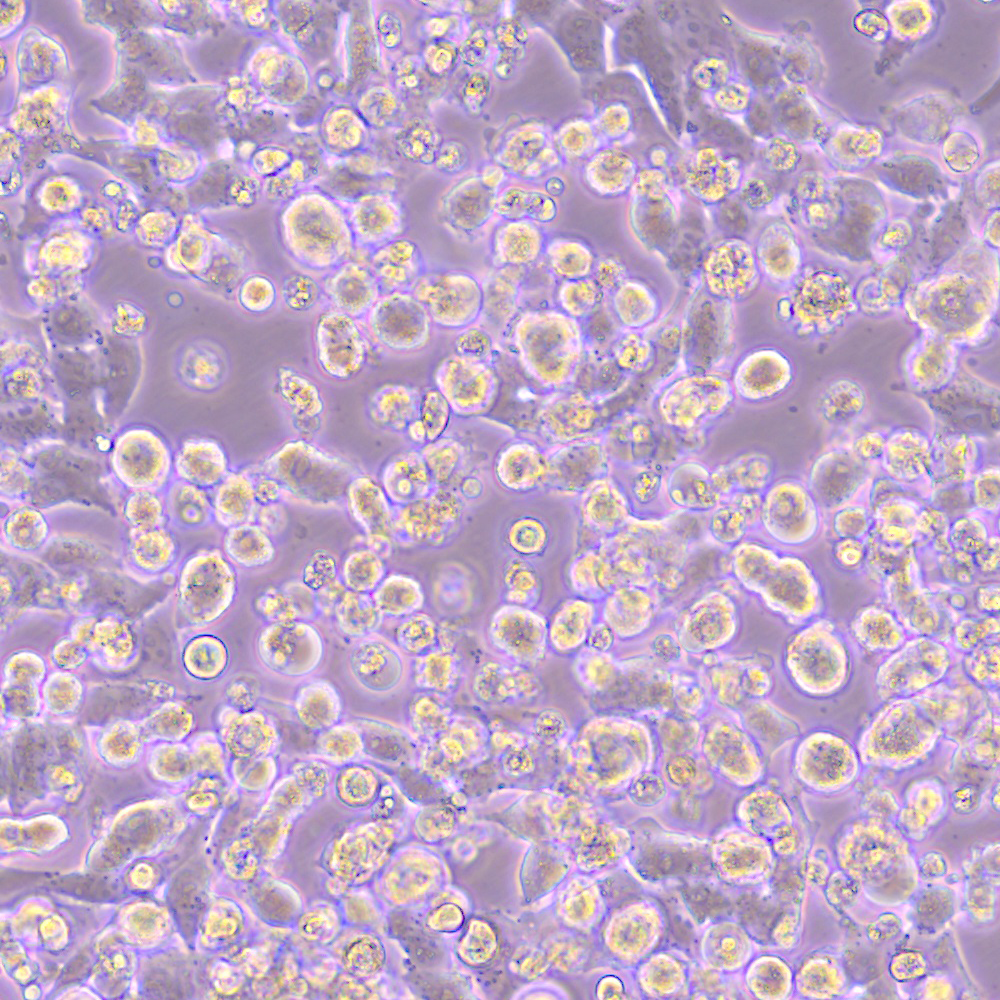

Supplement: Supplementary file 3 — Source data Fig. 1 [file 44318_2025_369_MOESM3_ESM.zip › Figure 1/1I/Bright filed/shSLC25A1+RSL3.png]

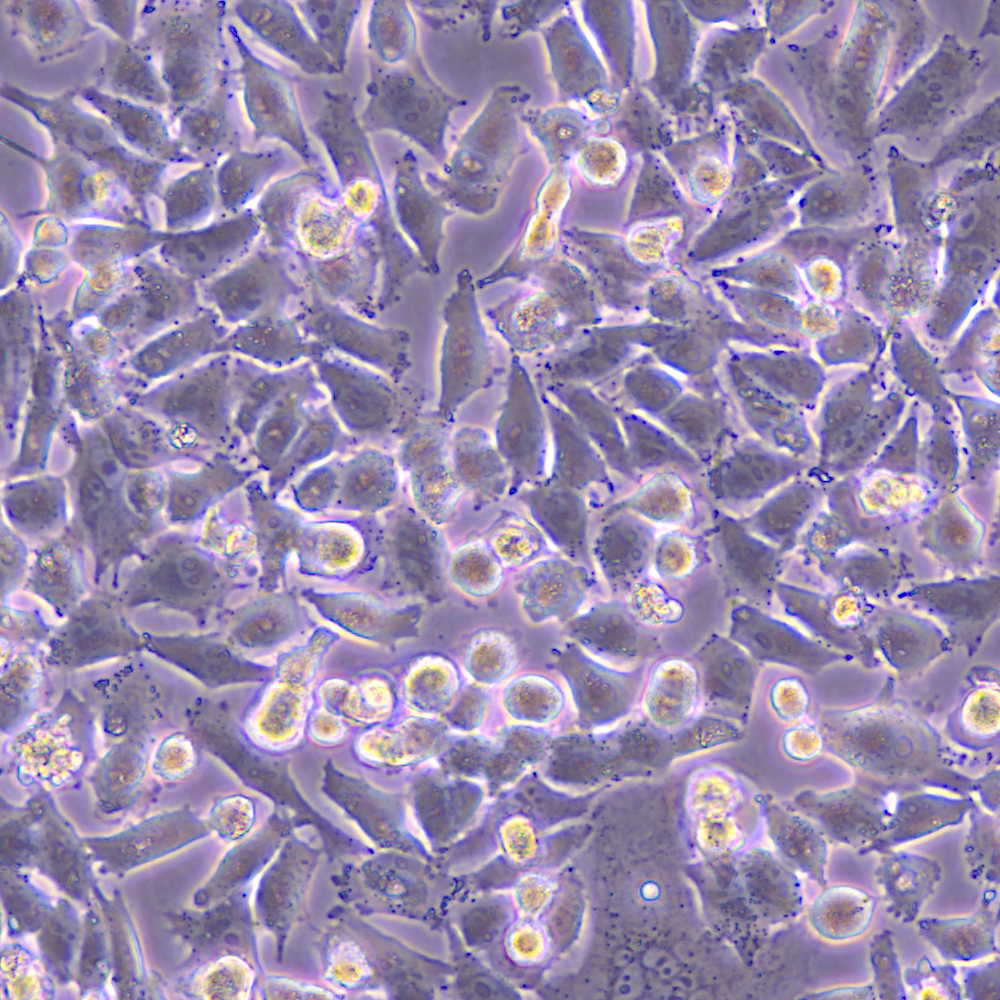

Supplement: Supplementary file 3 — Source data Fig. 1 [file 44318_2025_369_MOESM3_ESM.zip › Figure 1/1I/Bright filed/shSLC25A1.png]

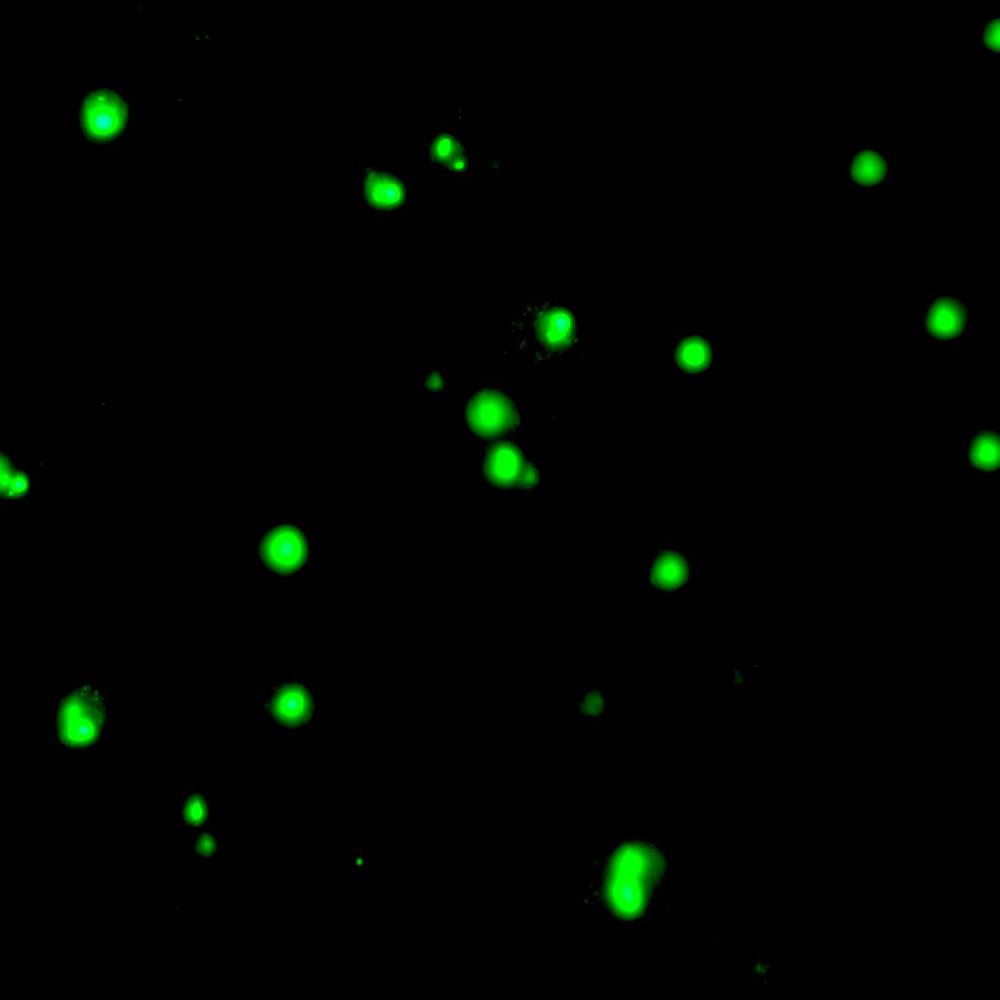

Supplement: Supplementary file 3 — Source data Fig. 1 [file 44318_2025_369_MOESM3_ESM.zip › Figure 1/1I/SYTOX Green/shCtrl+RSL3.png]

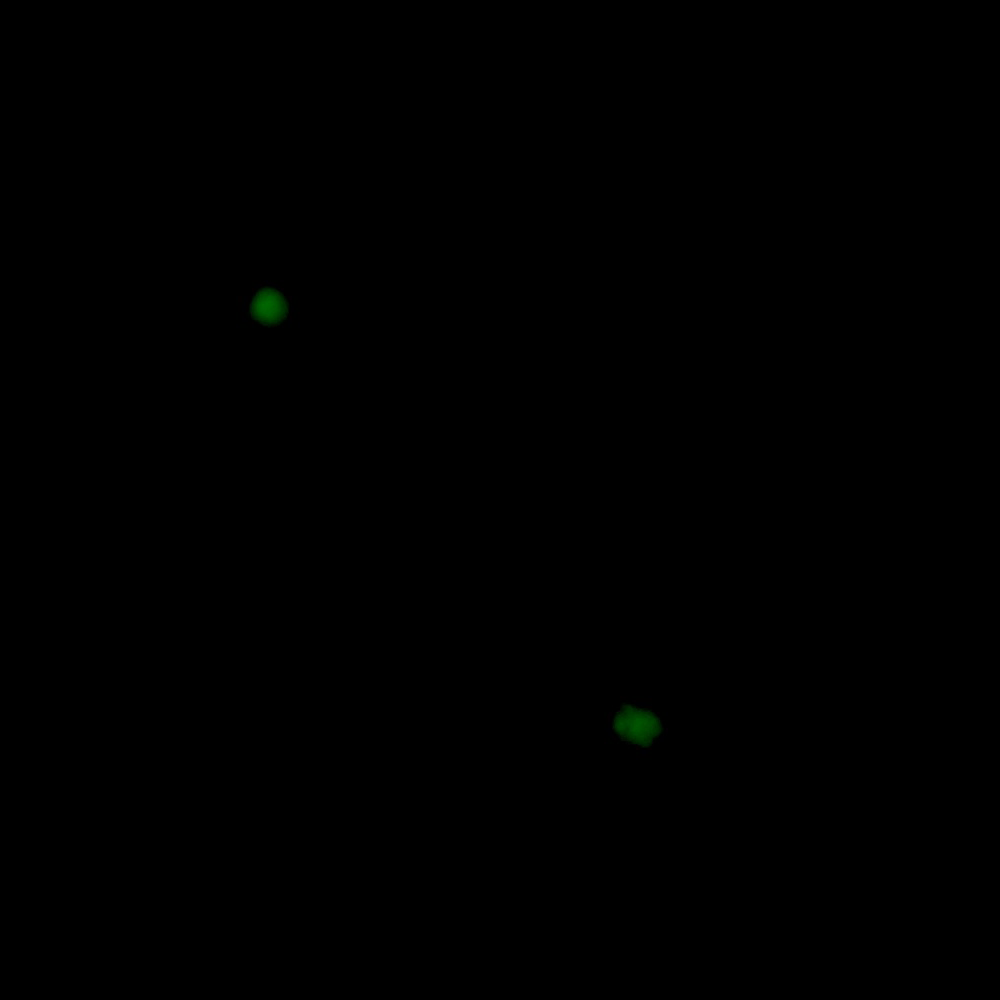

Supplement: Supplementary file 3 — Source data Fig. 1 [file 44318_2025_369_MOESM3_ESM.zip › Figure 1/1I/SYTOX Green/shCtrl.png]

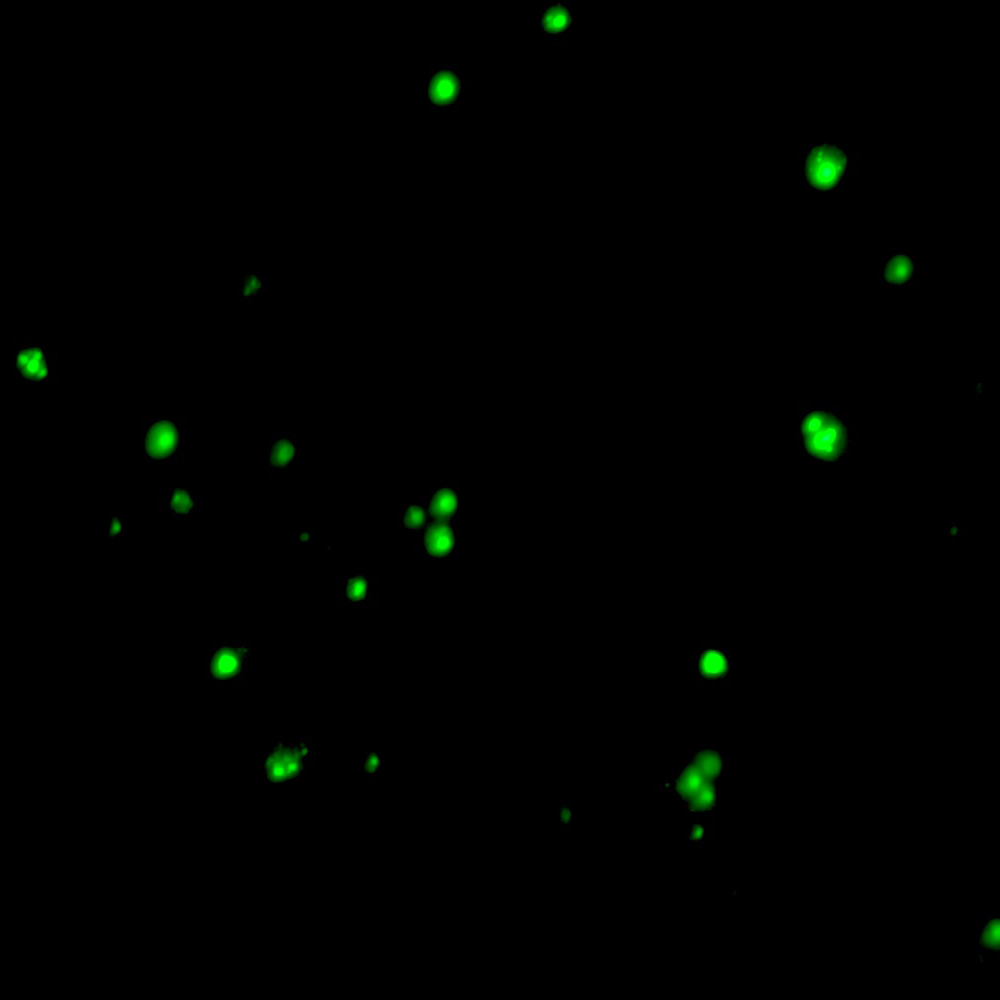

Supplement: Supplementary file 3 — Source data Fig. 1 [file 44318_2025_369_MOESM3_ESM.zip › Figure 1/1I/SYTOX Green/shSLC25A1+RSL3-Fer-1.png]

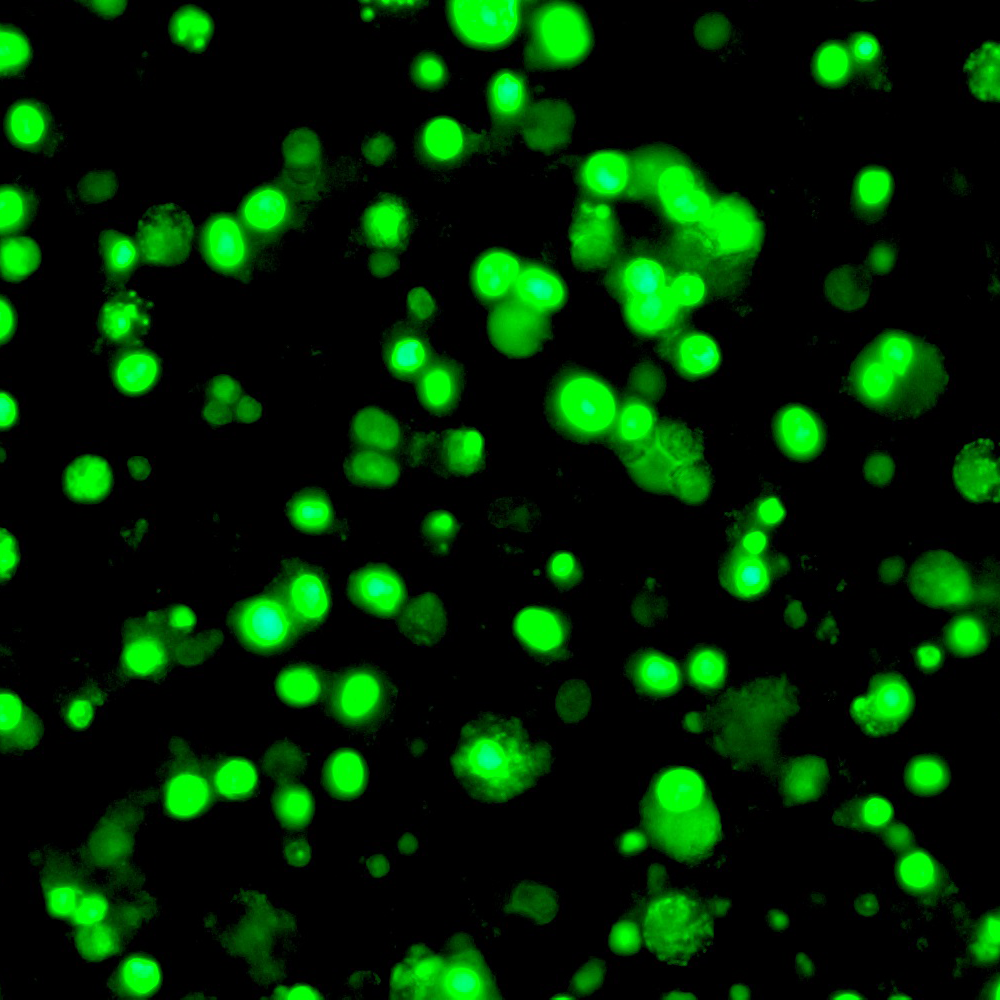

Supplement: Supplementary file 3 — Source data Fig. 1 [file 44318_2025_369_MOESM3_ESM.zip › Figure 1/1I/SYTOX Green/shSLC25A1+RSL3.png]

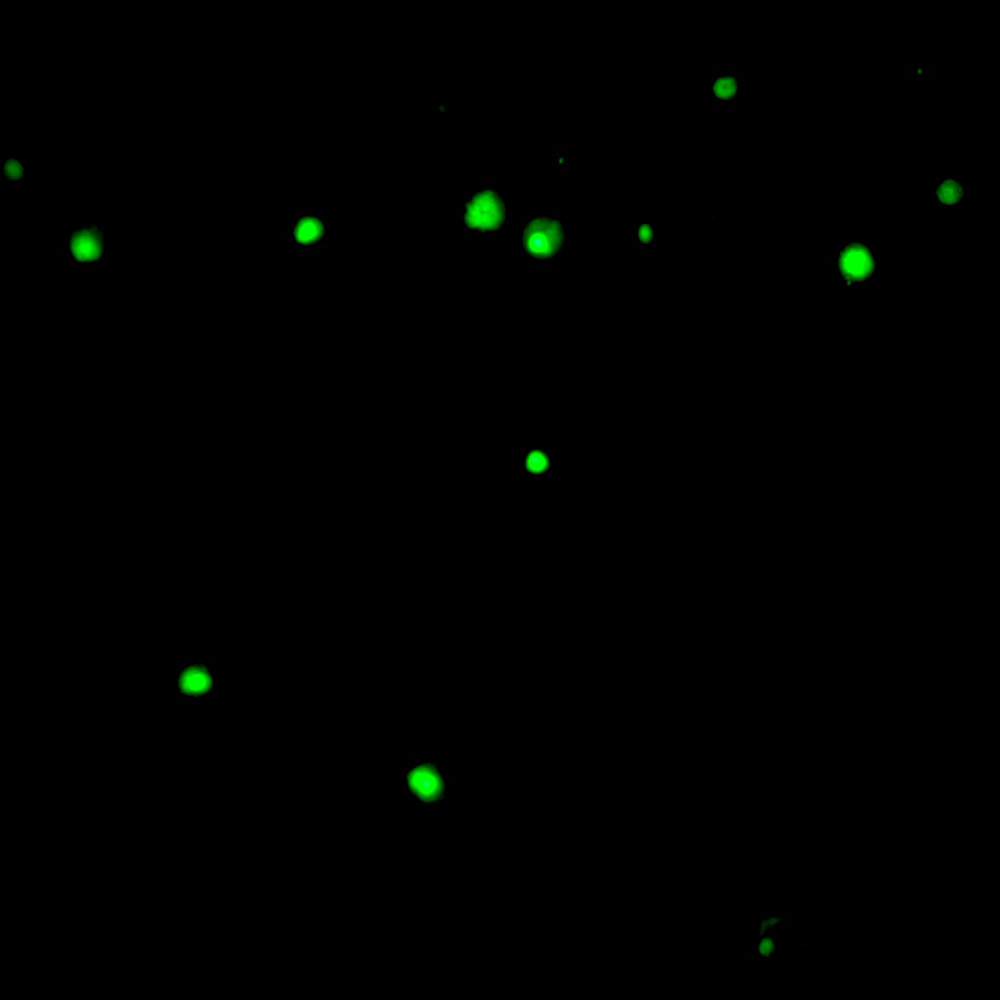

Supplement: Supplementary file 3 — Source data Fig. 1 [file 44318_2025_369_MOESM3_ESM.zip › Figure 1/1I/SYTOX Green/shSLC25A1.png]

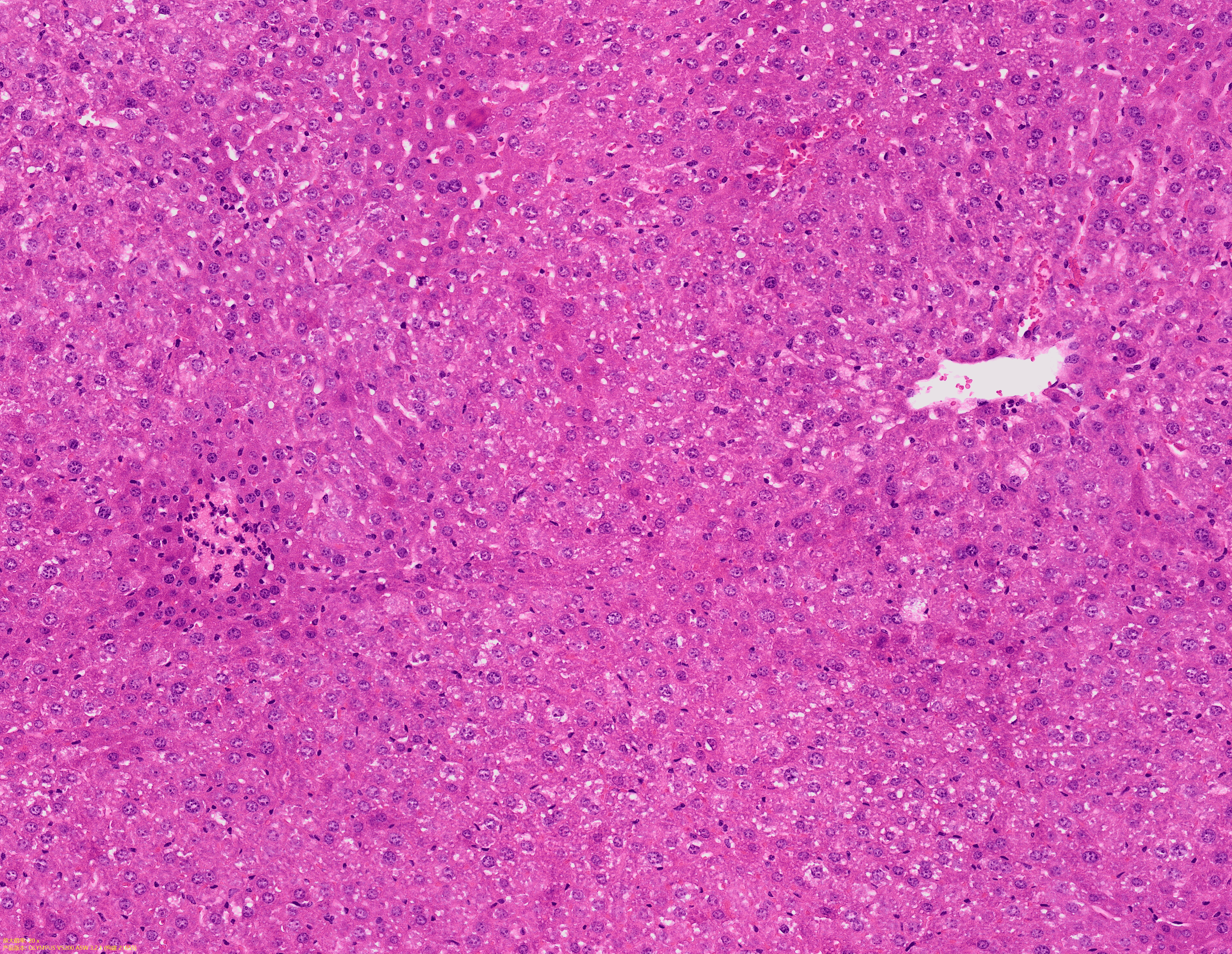

Supplement: Supplementary file 3 — Source data Fig. 1 [file 44318_2025_369_MOESM3_ESM.zip › Figure 1/1N/HE/Slc25a1-KD+IRI+Lip-1.png]

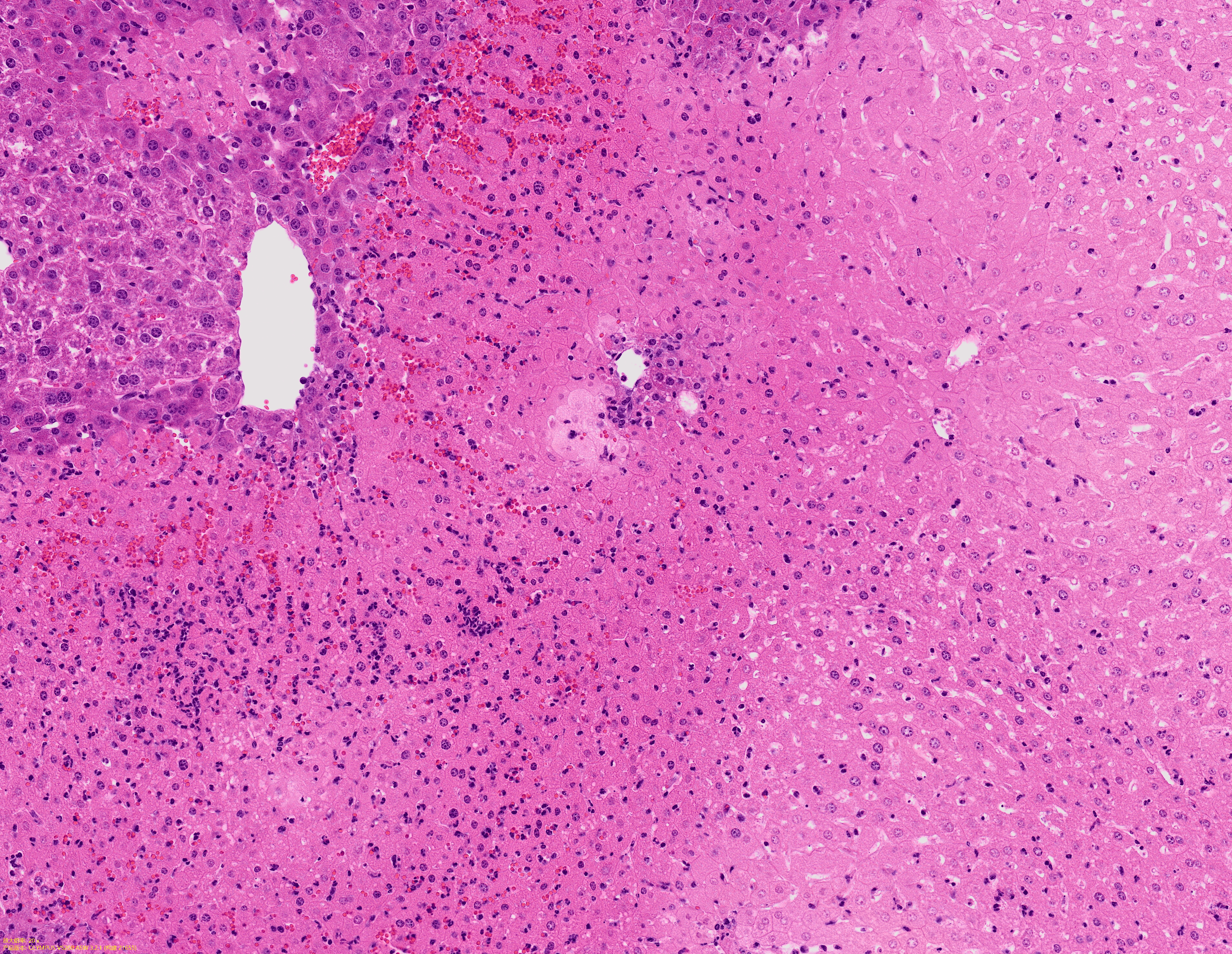

Supplement: Supplementary file 3 — Source data Fig. 1 [file 44318_2025_369_MOESM3_ESM.zip › Figure 1/1N/HE/Slc25a1-KD+IRI.png]

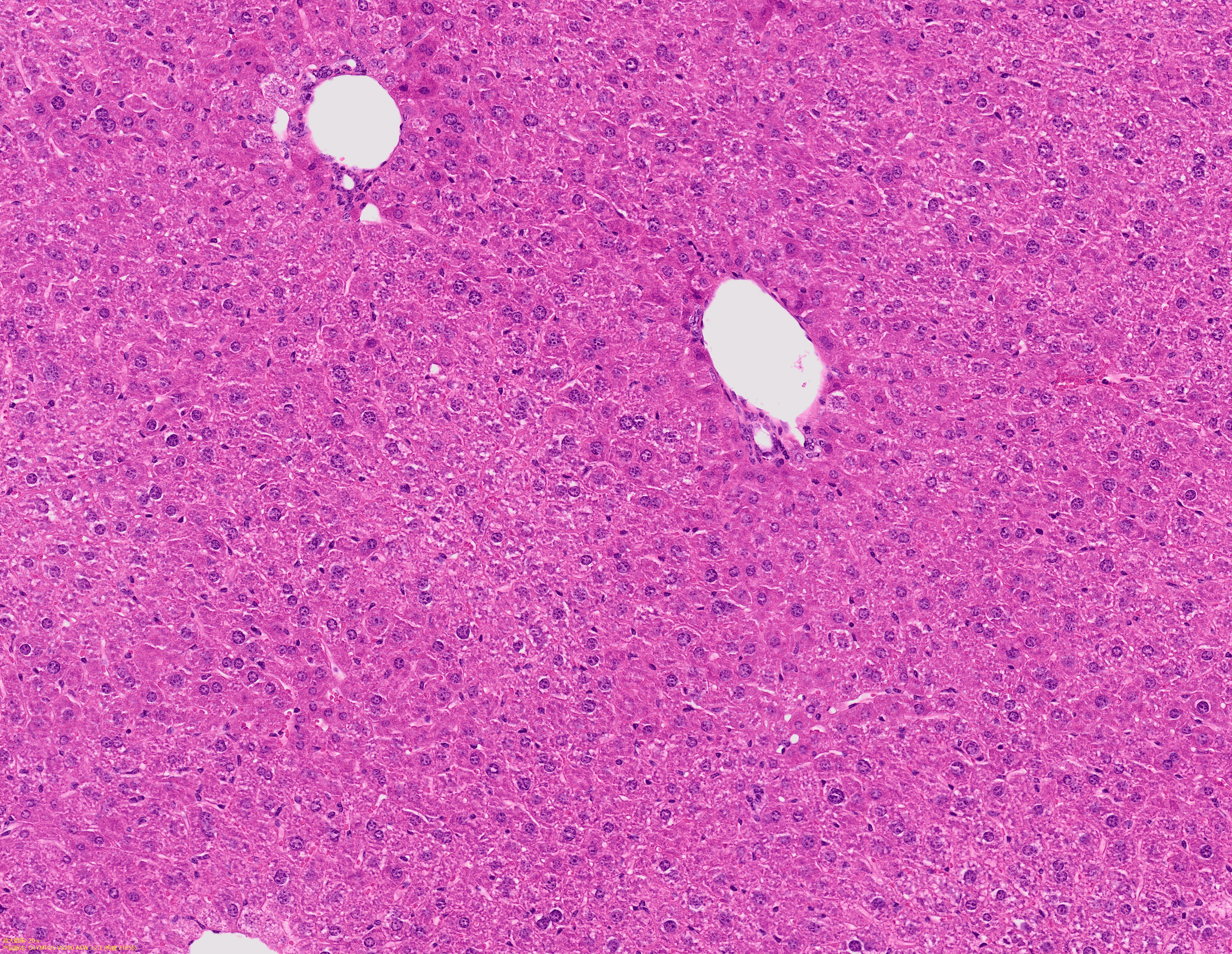

Supplement: Supplementary file 3 — Source data Fig. 1 [file 44318_2025_369_MOESM3_ESM.zip › Figure 1/1N/HE/Slc25a1-KD.png]

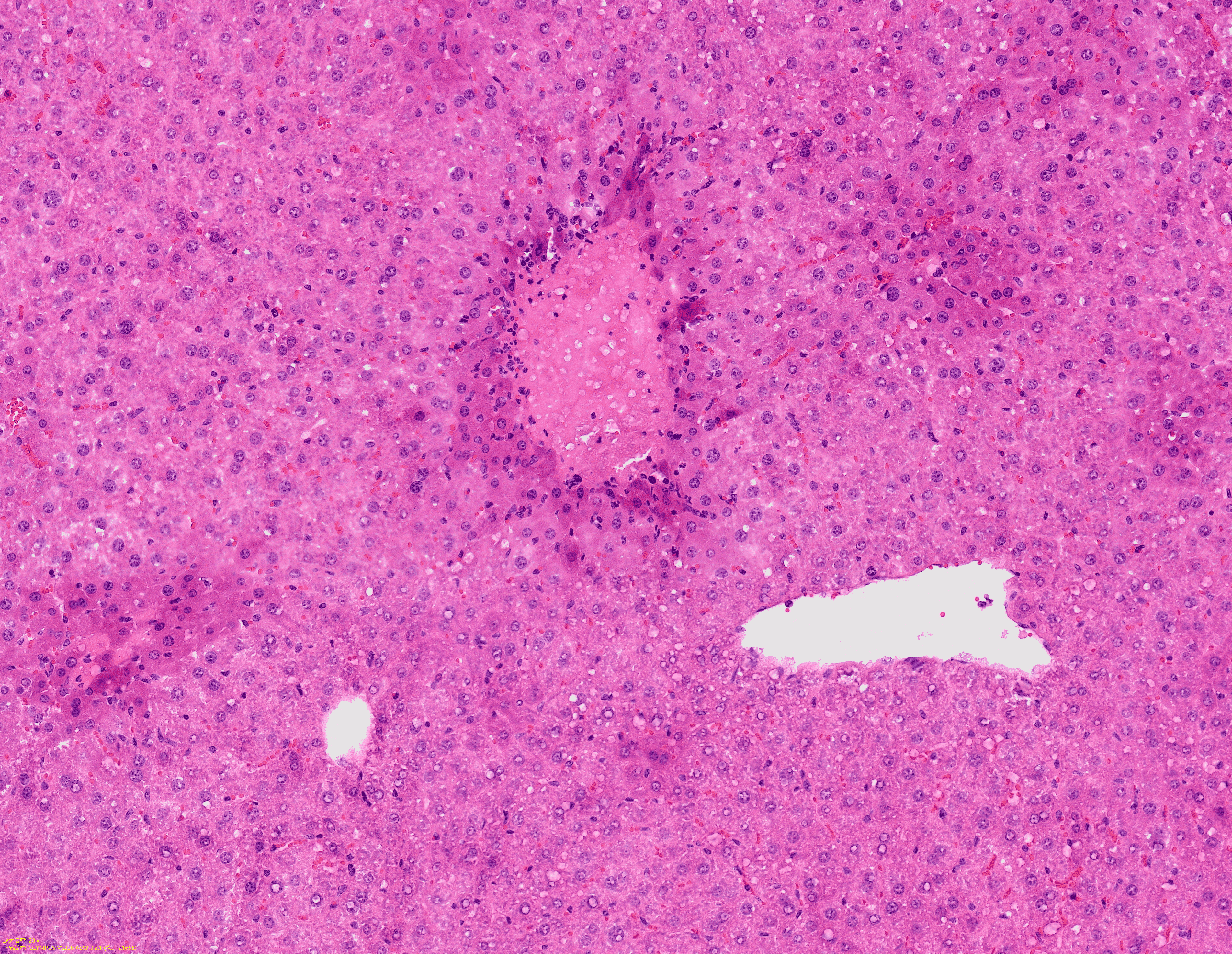

Supplement: Supplementary file 3 — Source data Fig. 1 [file 44318_2025_369_MOESM3_ESM.zip › Figure 1/1N/HE/Vehicle+IRI.png]

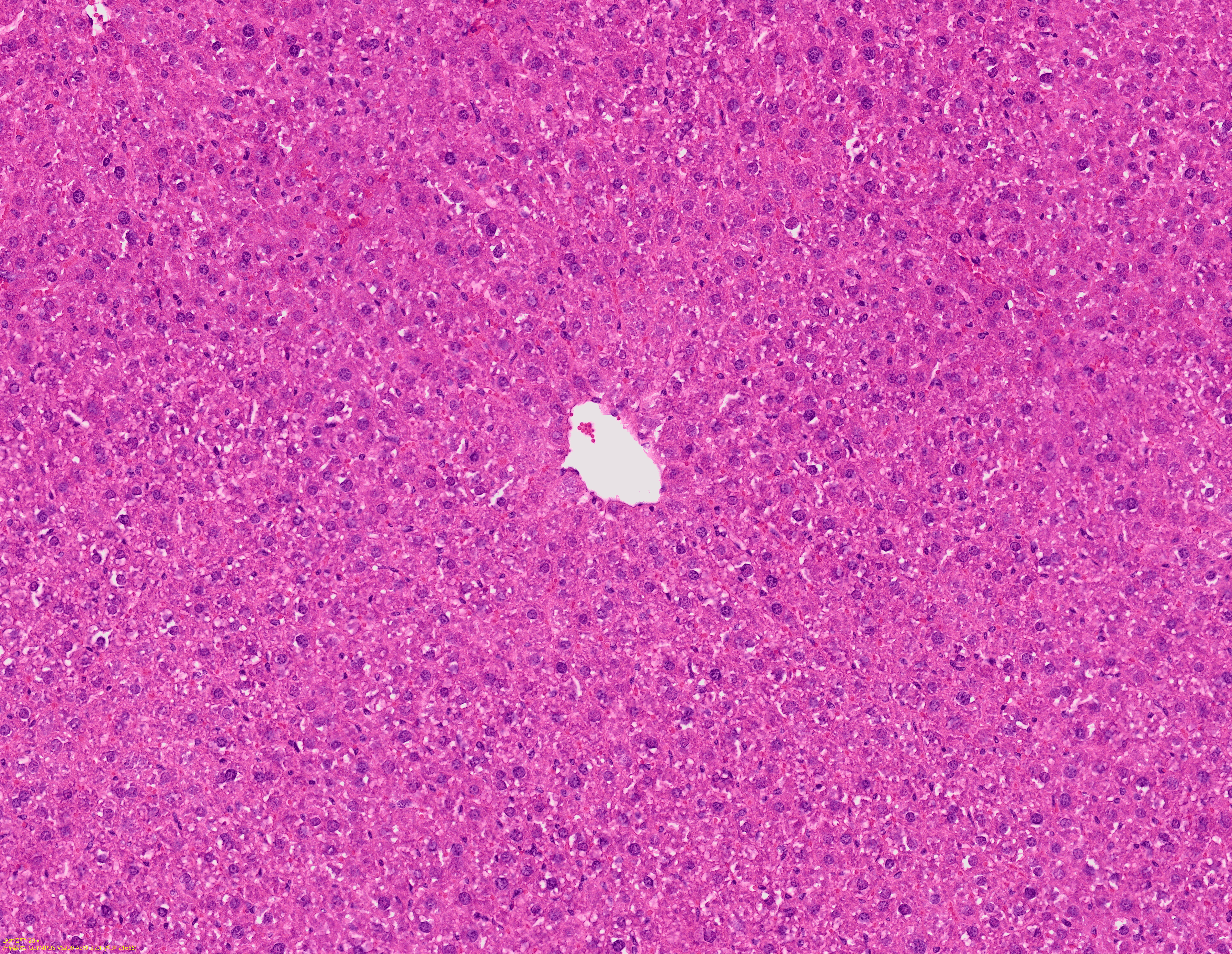

Supplement: Supplementary file 3 — Source data Fig. 1 [file 44318_2025_369_MOESM3_ESM.zip › Figure 1/1N/HE/Vehicle+Sham+Lip-1.png]

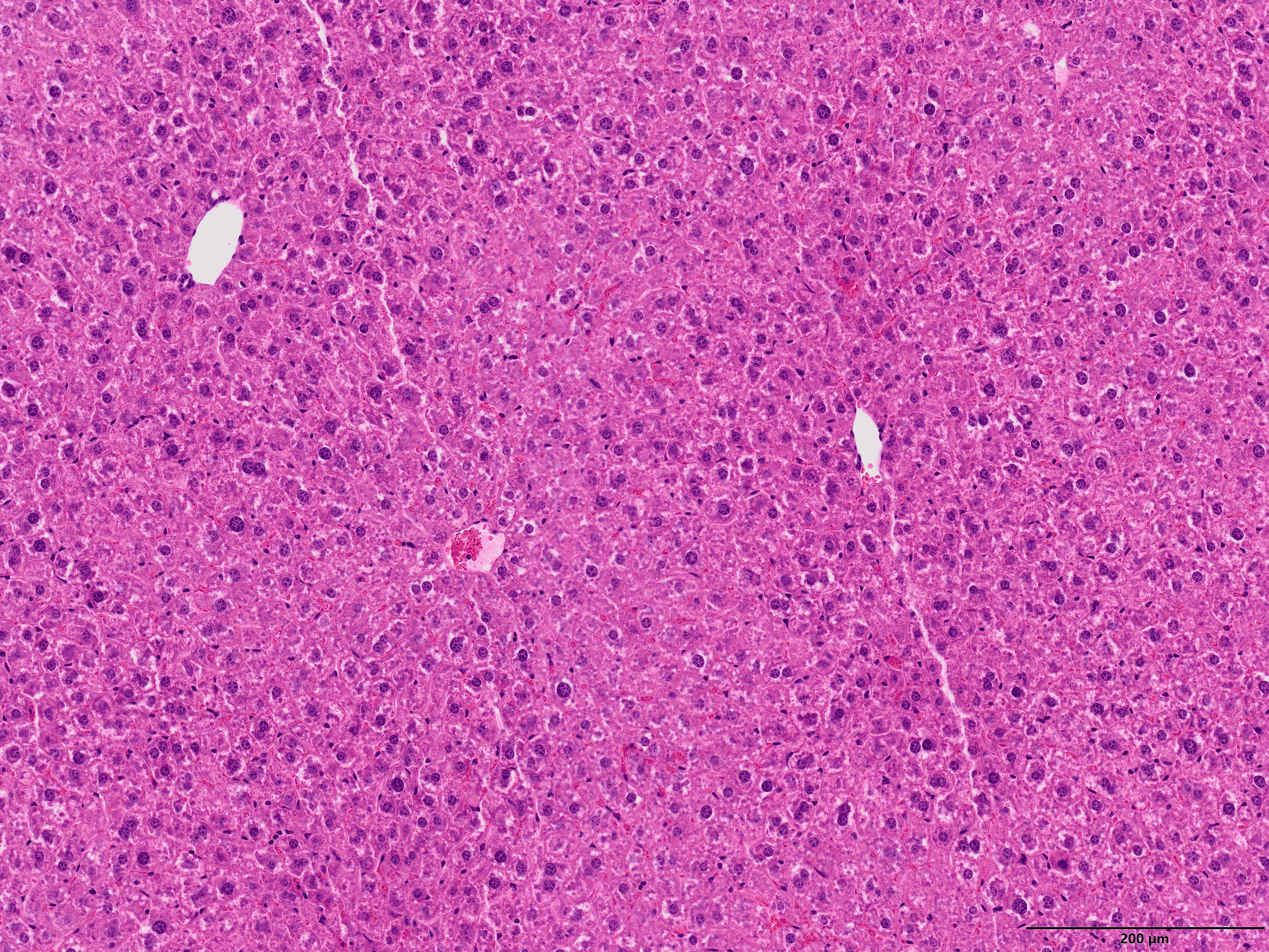

Supplement: Supplementary file 3 — Source data Fig. 1 [file 44318_2025_369_MOESM3_ESM.zip › Figure 1/1N/HE/Vehicle+Sham.png]

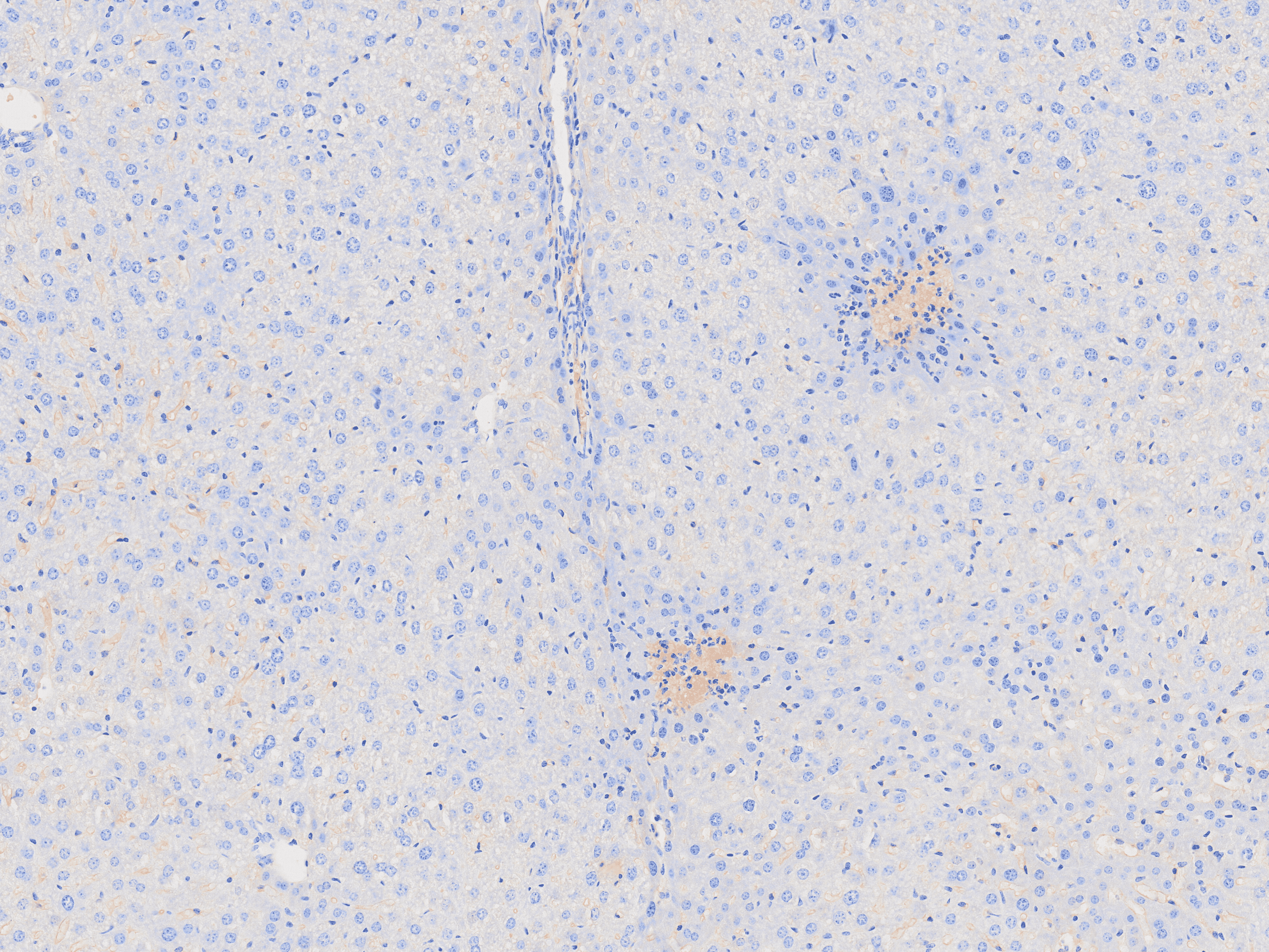

Supplement: Supplementary file 3 — Source data Fig. 1 [file 44318_2025_369_MOESM3_ESM.zip › Figure 1/1N/MDA/Slc25a1-KD+IRI+Lip-1.png]

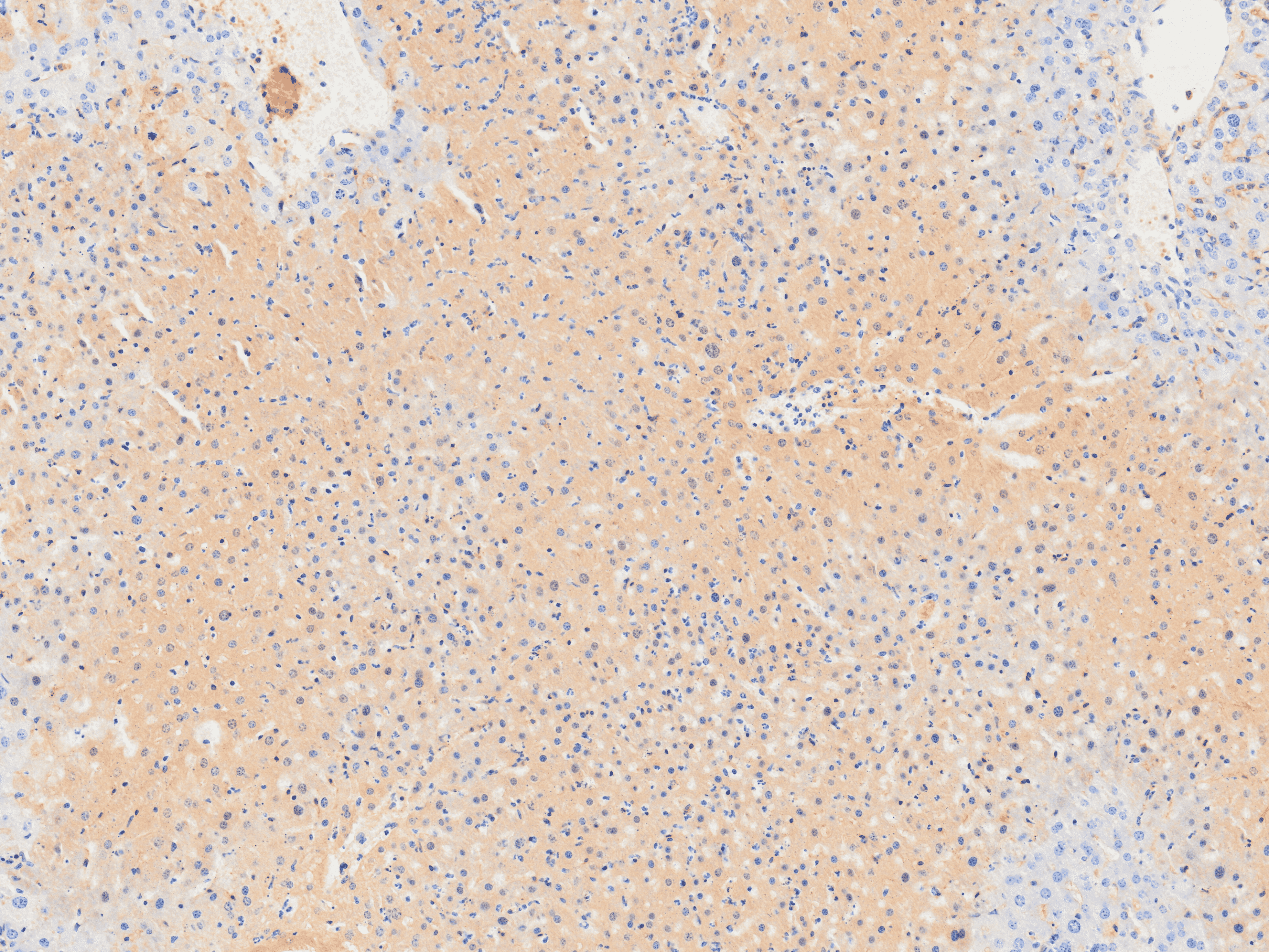

Supplement: Supplementary file 3 — Source data Fig. 1 [file 44318_2025_369_MOESM3_ESM.zip › Figure 1/1N/MDA/Slc25a1-KD+IRI.png]

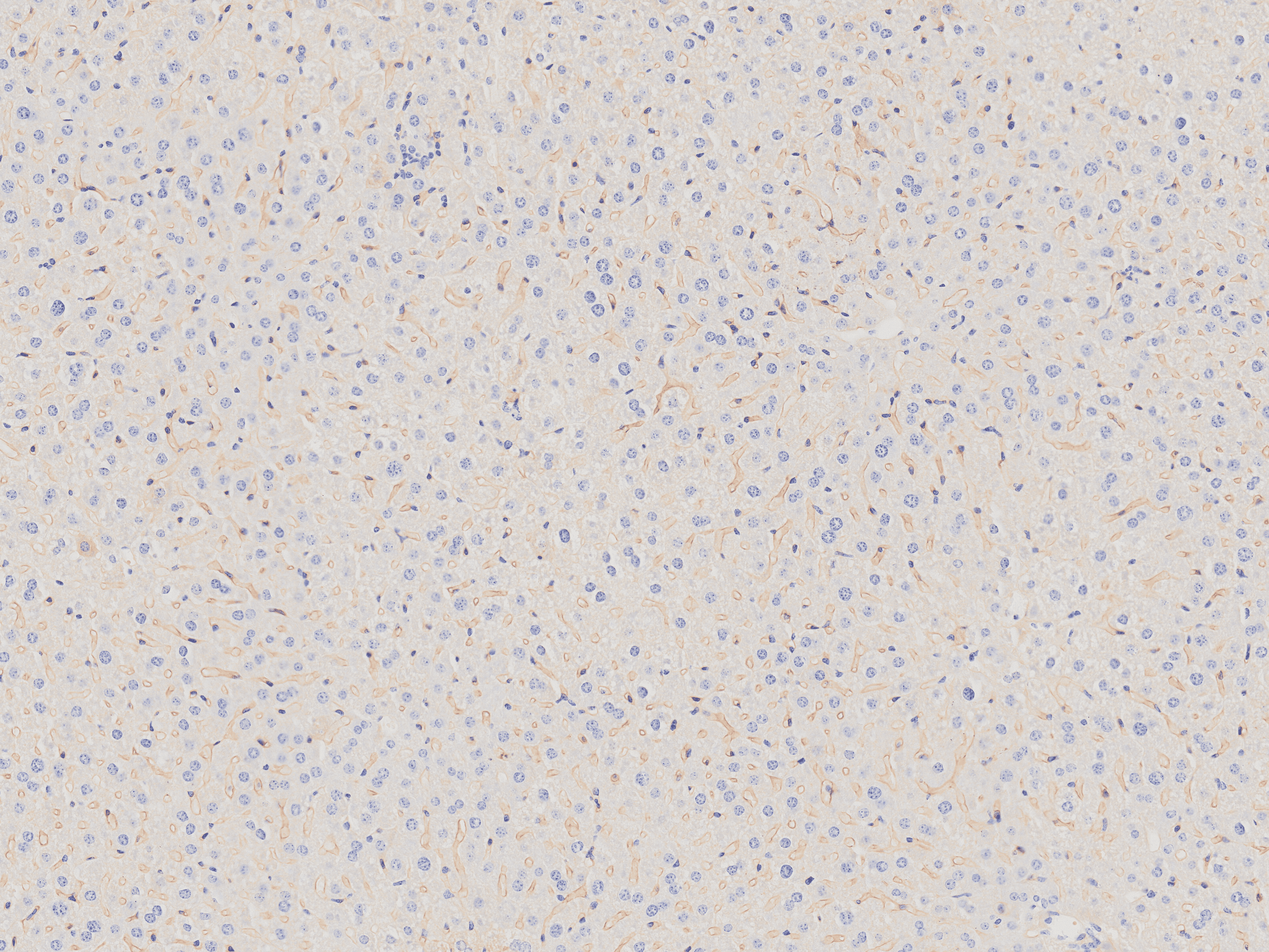

Supplement: Supplementary file 3 — Source data Fig. 1 [file 44318_2025_369_MOESM3_ESM.zip › Figure 1/1N/MDA/Slc25a1-KD.png]

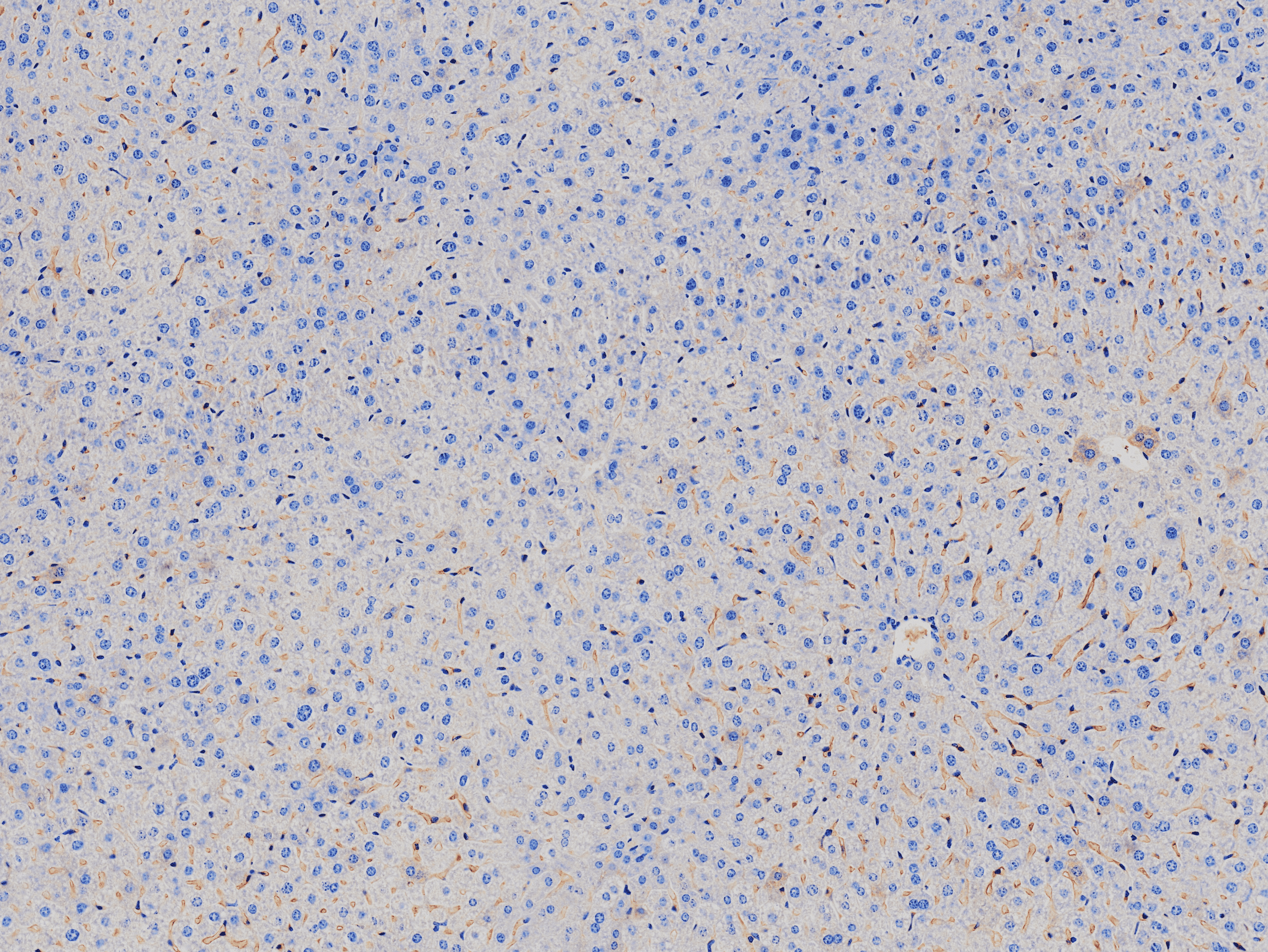

Supplement: Supplementary file 3 — Source data Fig. 1 [file 44318_2025_369_MOESM3_ESM.zip › Figure 1/1N/MDA/Vehicle+IRI+Lip-1.png]

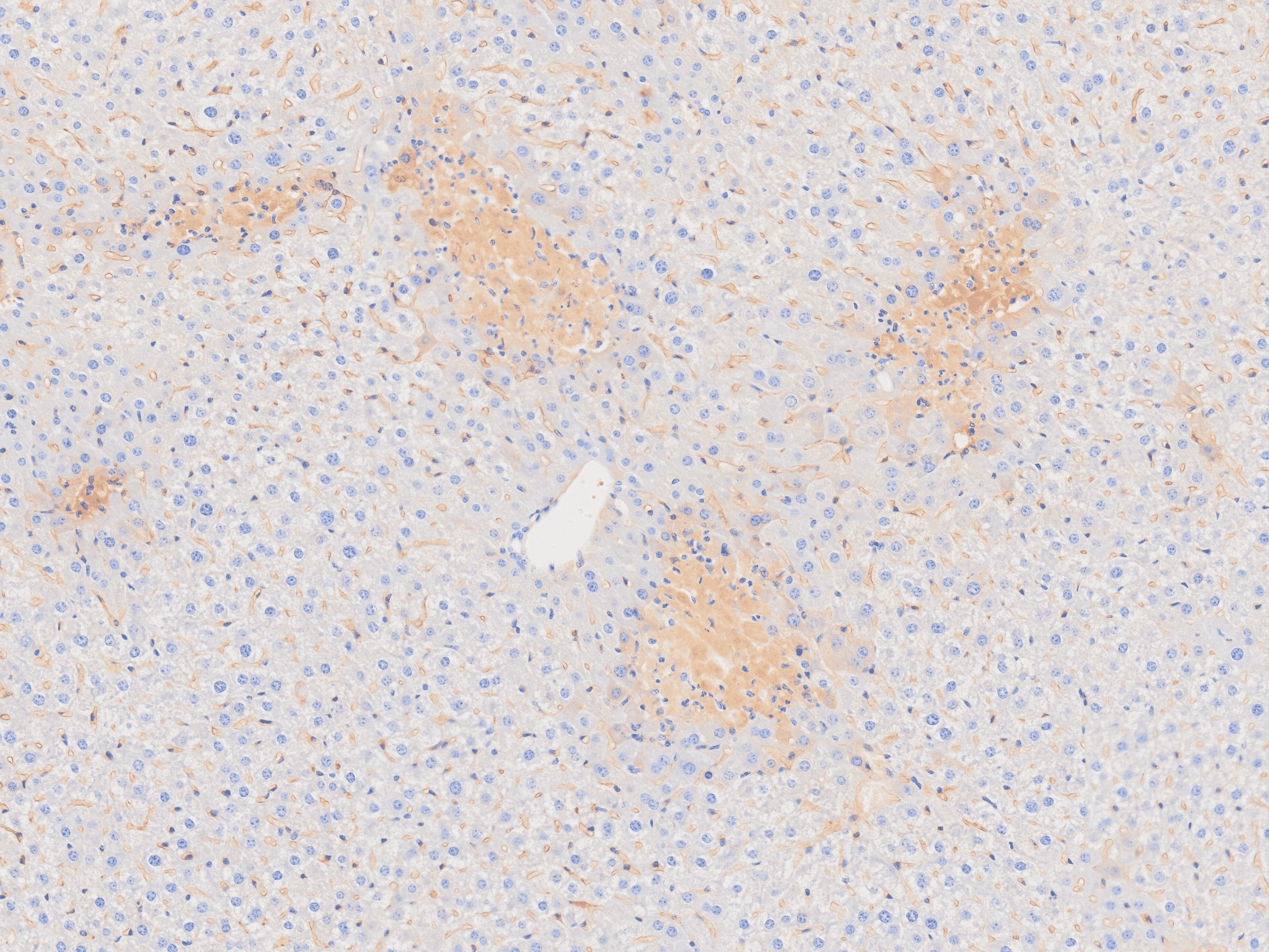

Supplement: Supplementary file 3 — Source data Fig. 1 [file 44318_2025_369_MOESM3_ESM.zip › Figure 1/1N/MDA/Vehicle+IRI.png]

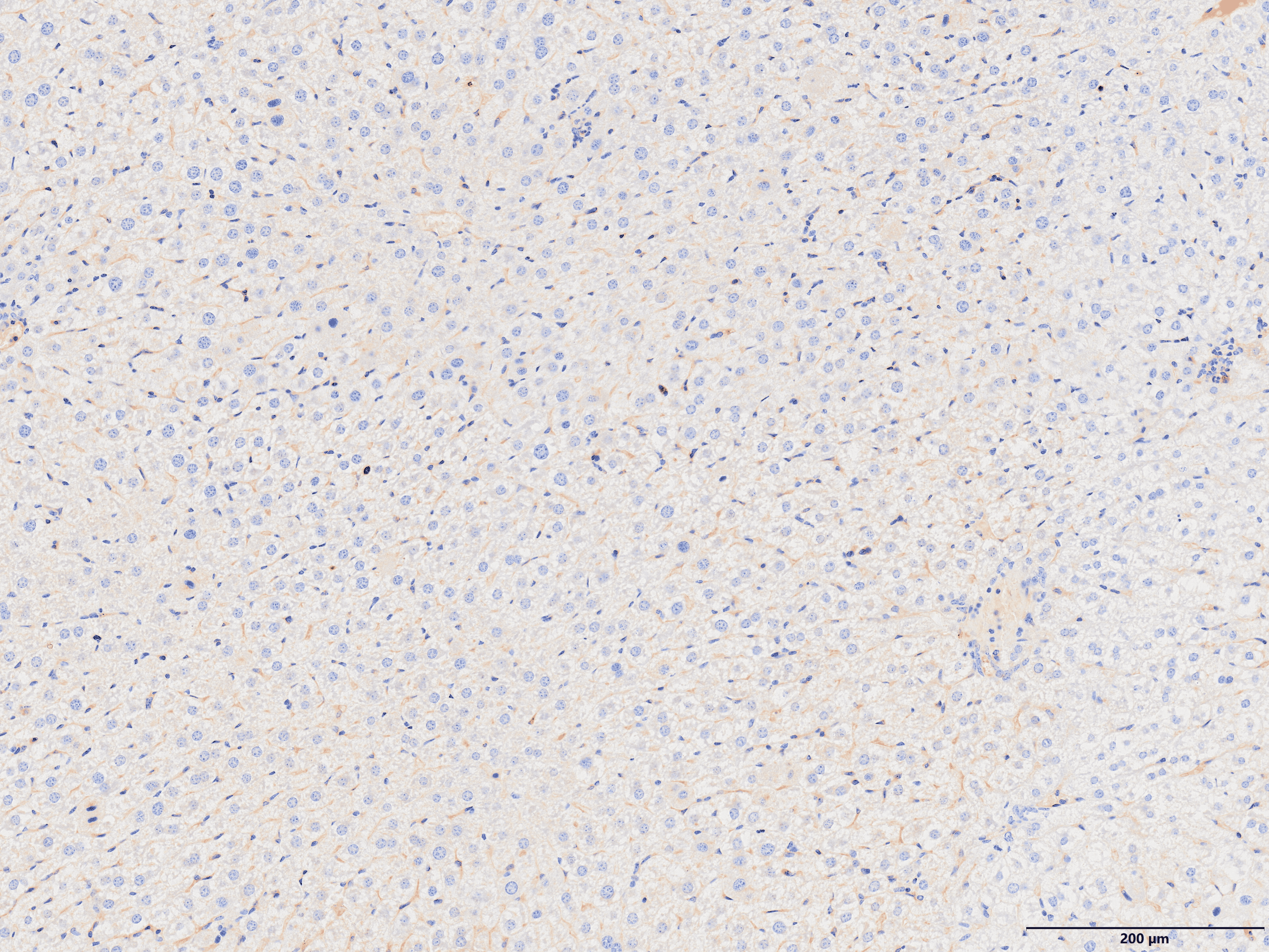

Supplement: Supplementary file 3 — Source data Fig. 1 [file 44318_2025_369_MOESM3_ESM.zip › Figure 1/1N/MDA/Vehicle+Sham.png]

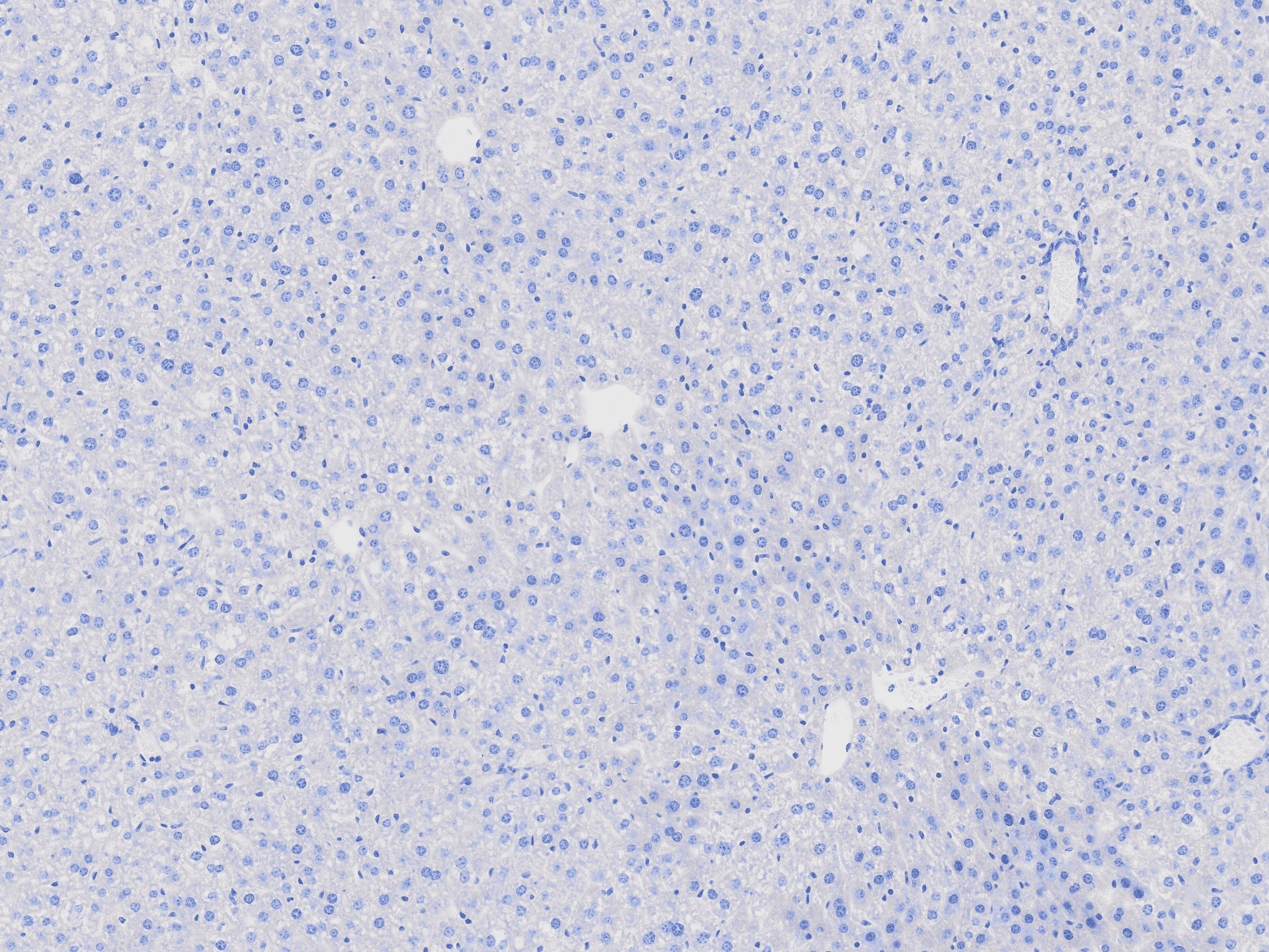

Supplement: Supplementary file 3 — Source data Fig. 1 [file 44318_2025_369_MOESM3_ESM.zip › Figure 1/1N/SLC25A1/Slc25a1-KD+IRI+Lip-1.png]

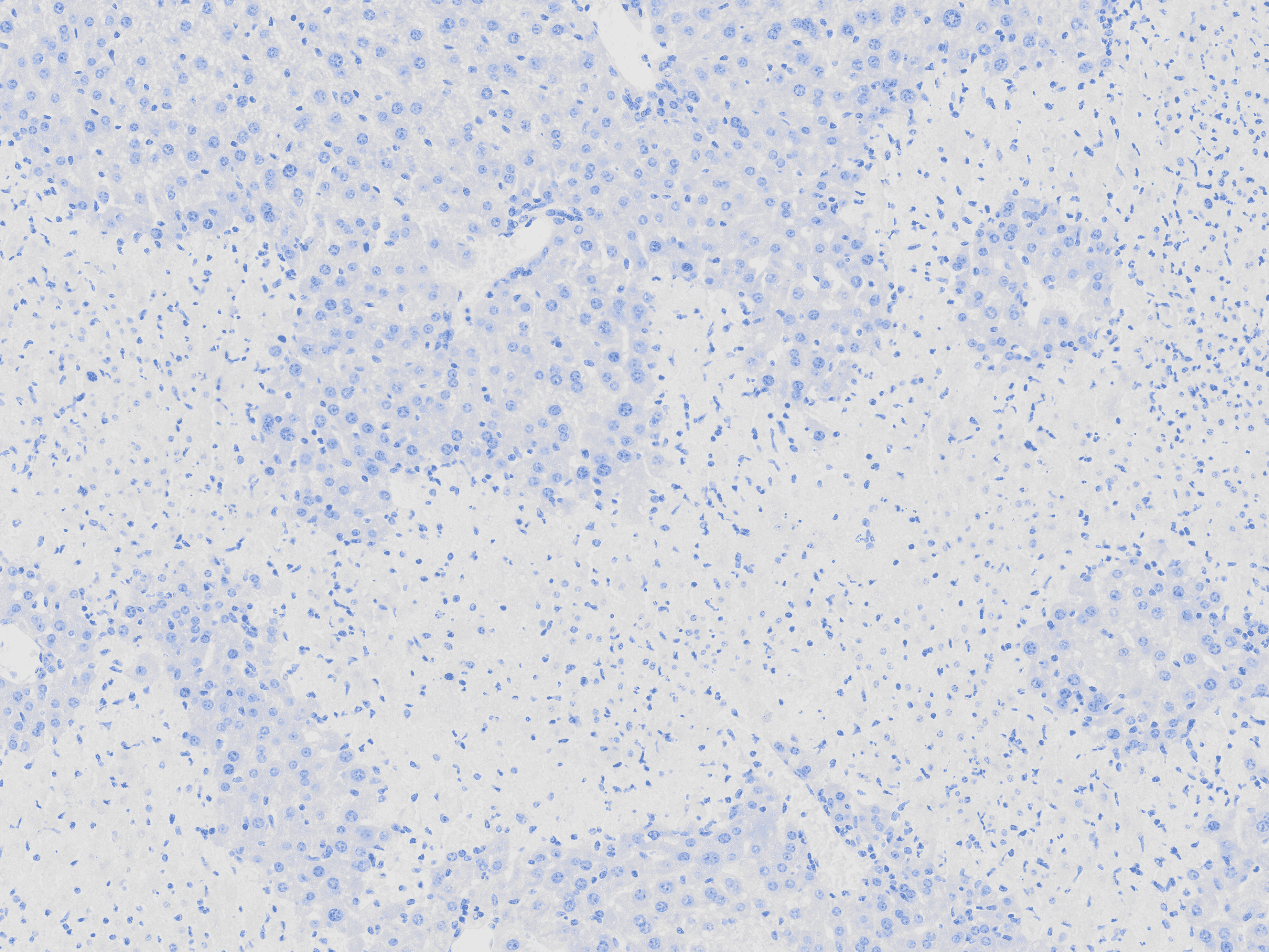

Supplement: Supplementary file 3 — Source data Fig. 1 [file 44318_2025_369_MOESM3_ESM.zip › Figure 1/1N/SLC25A1/Slc25a1-KD+IRI.png]

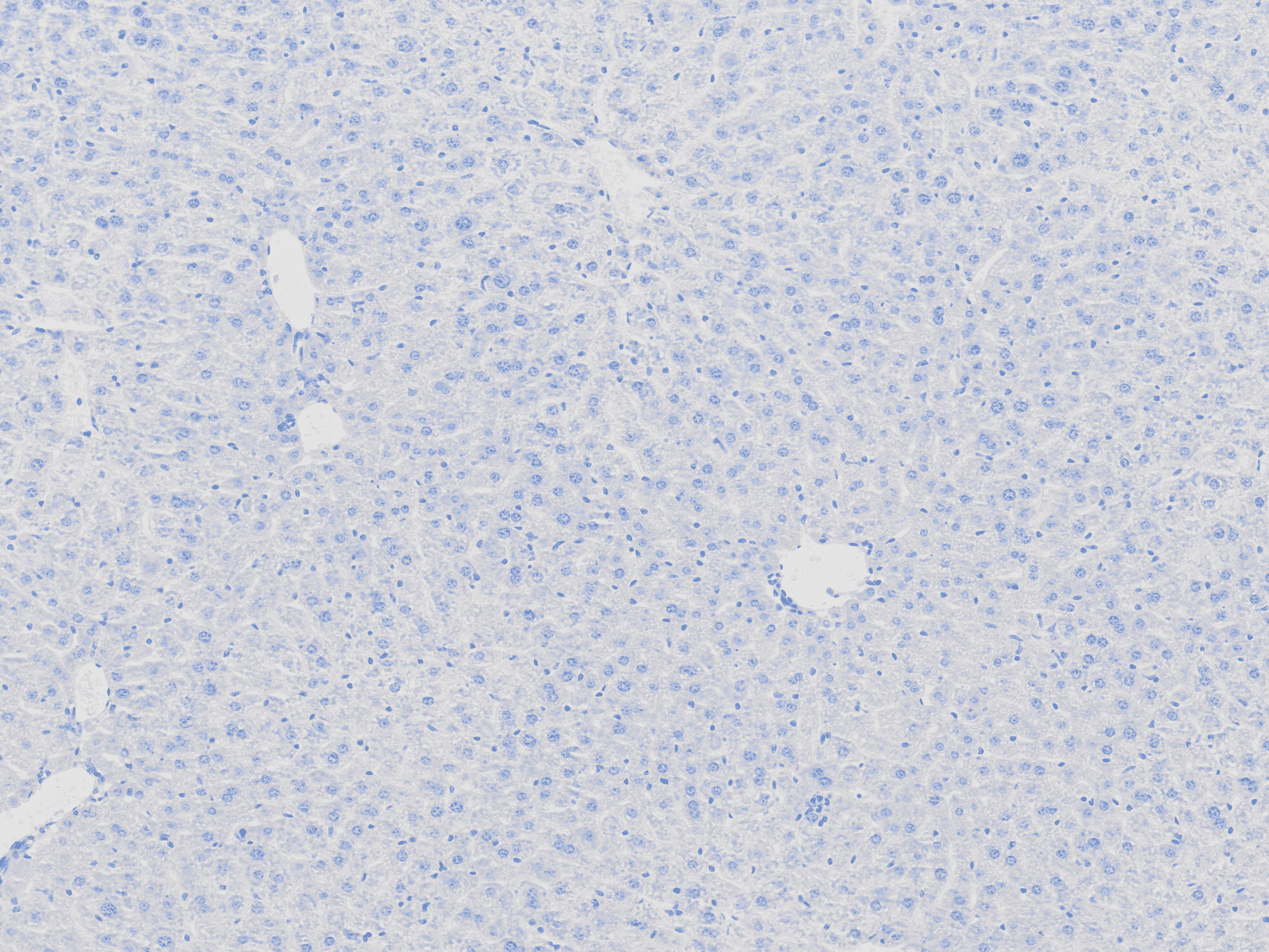

Supplement: Supplementary file 3 — Source data Fig. 1 [file 44318_2025_369_MOESM3_ESM.zip › Figure 1/1N/SLC25A1/Slc25a1-KD.png]

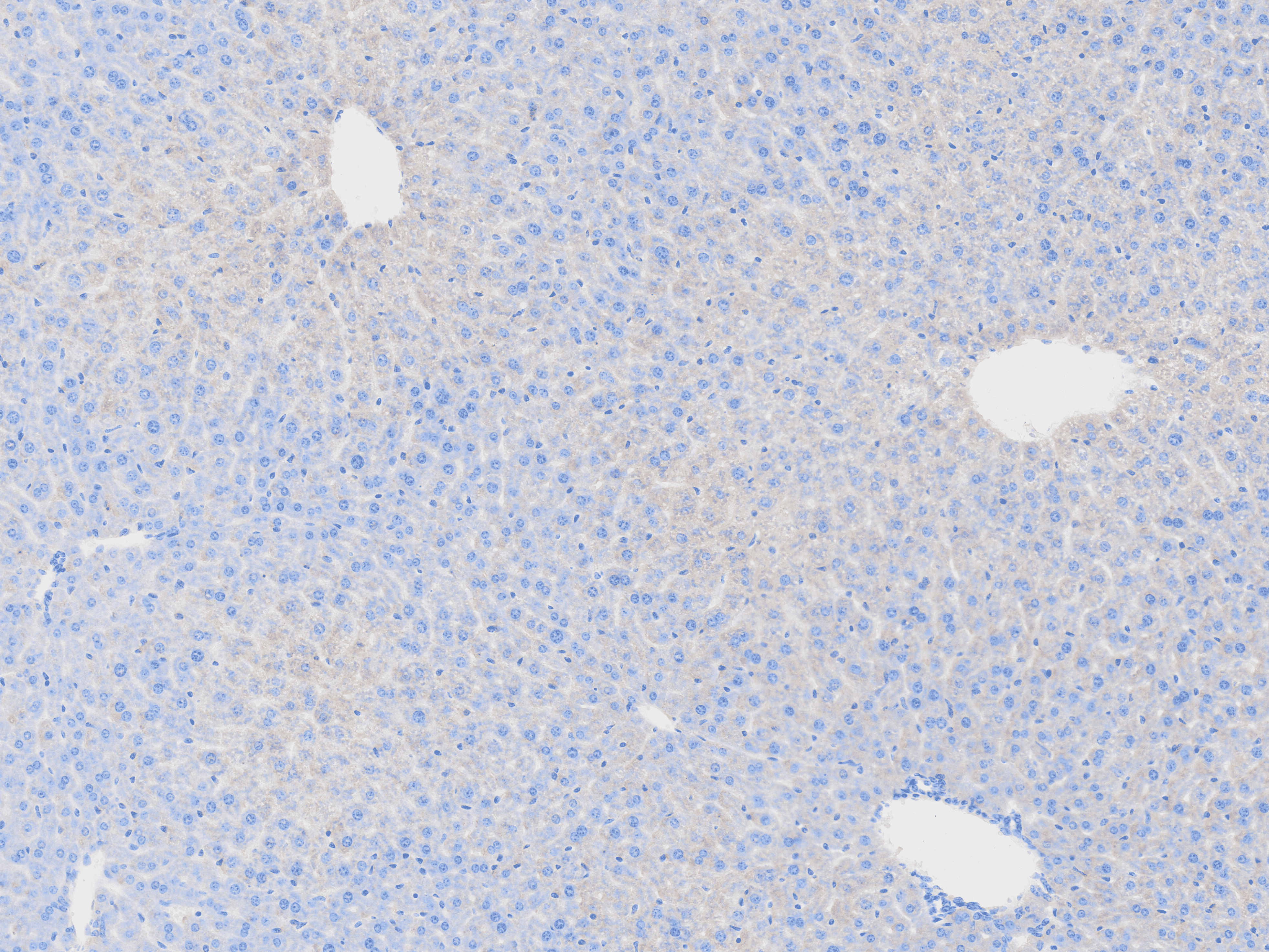

Supplement: Supplementary file 3 — Source data Fig. 1 [file 44318_2025_369_MOESM3_ESM.zip › Figure 1/1N/SLC25A1/Vehicle+IRI+Lip-1.png]

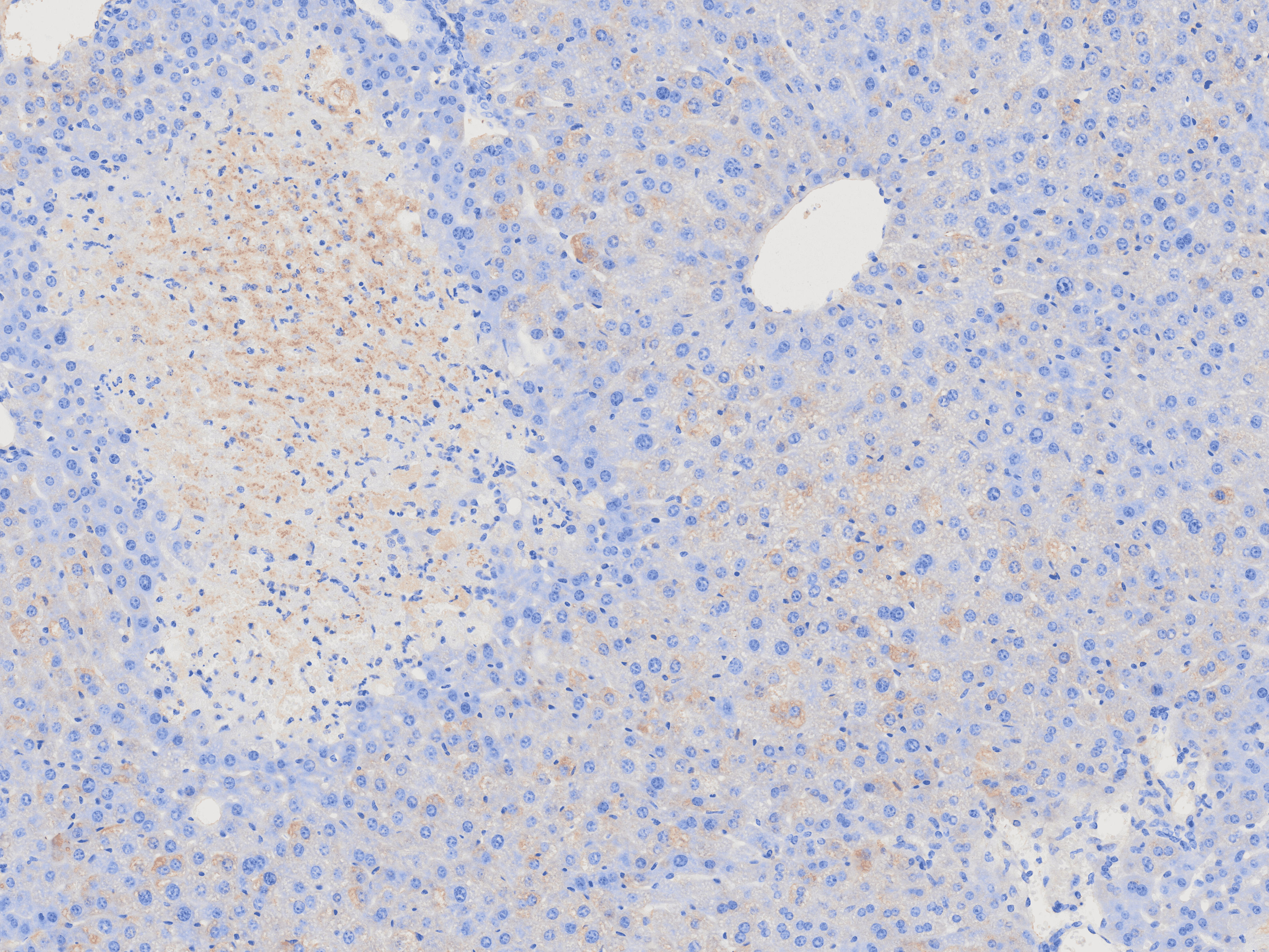

Supplement: Supplementary file 3 — Source data Fig. 1 [file 44318_2025_369_MOESM3_ESM.zip › Figure 1/1N/SLC25A1/Vehicle+IRI.png]

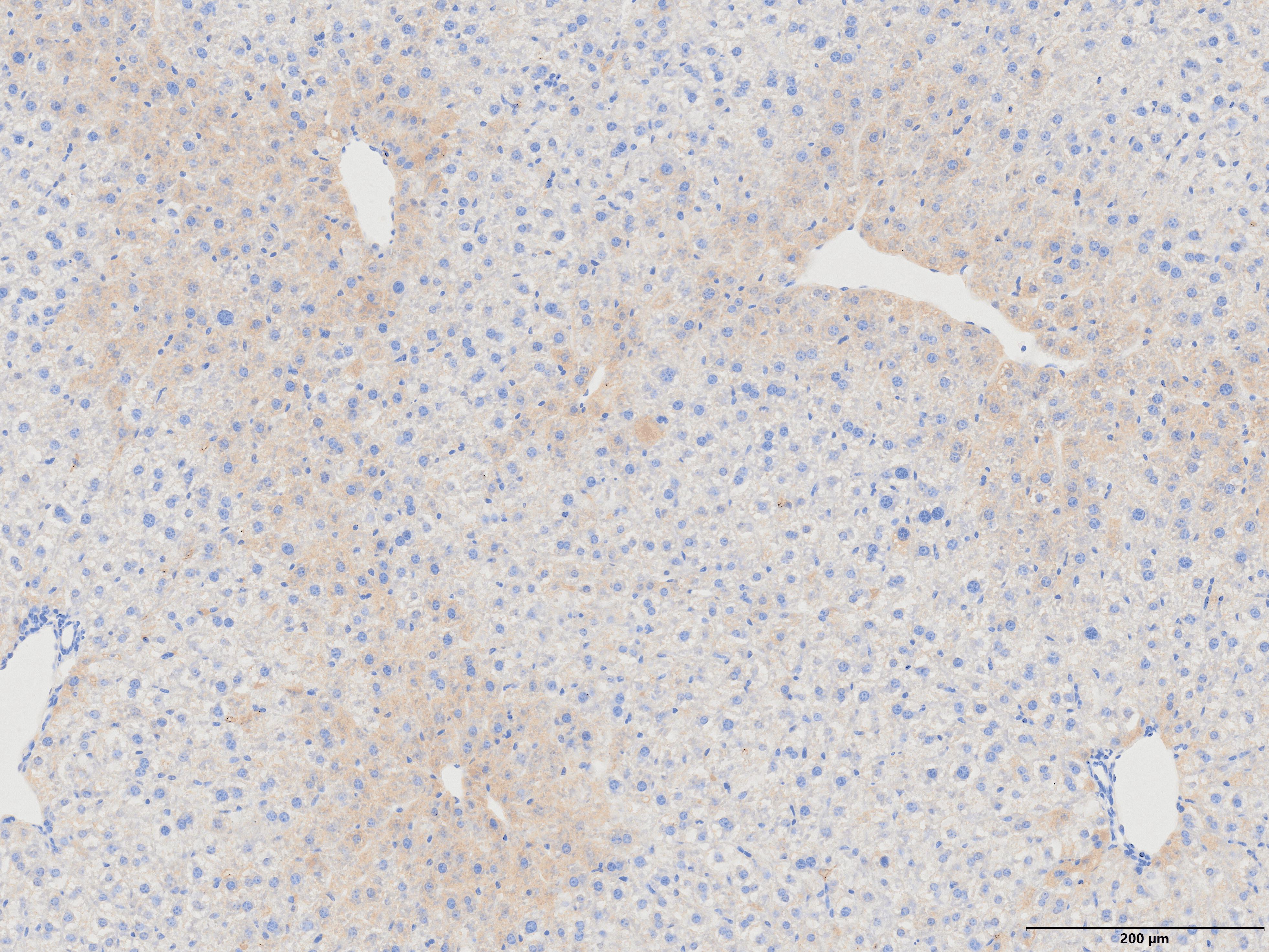

Supplement: Supplementary file 3 — Source data Fig. 1 [file 44318_2025_369_MOESM3_ESM.zip › Figure 1/1N/SLC25A1/Vehicle+Sham.png]

## Slide 1
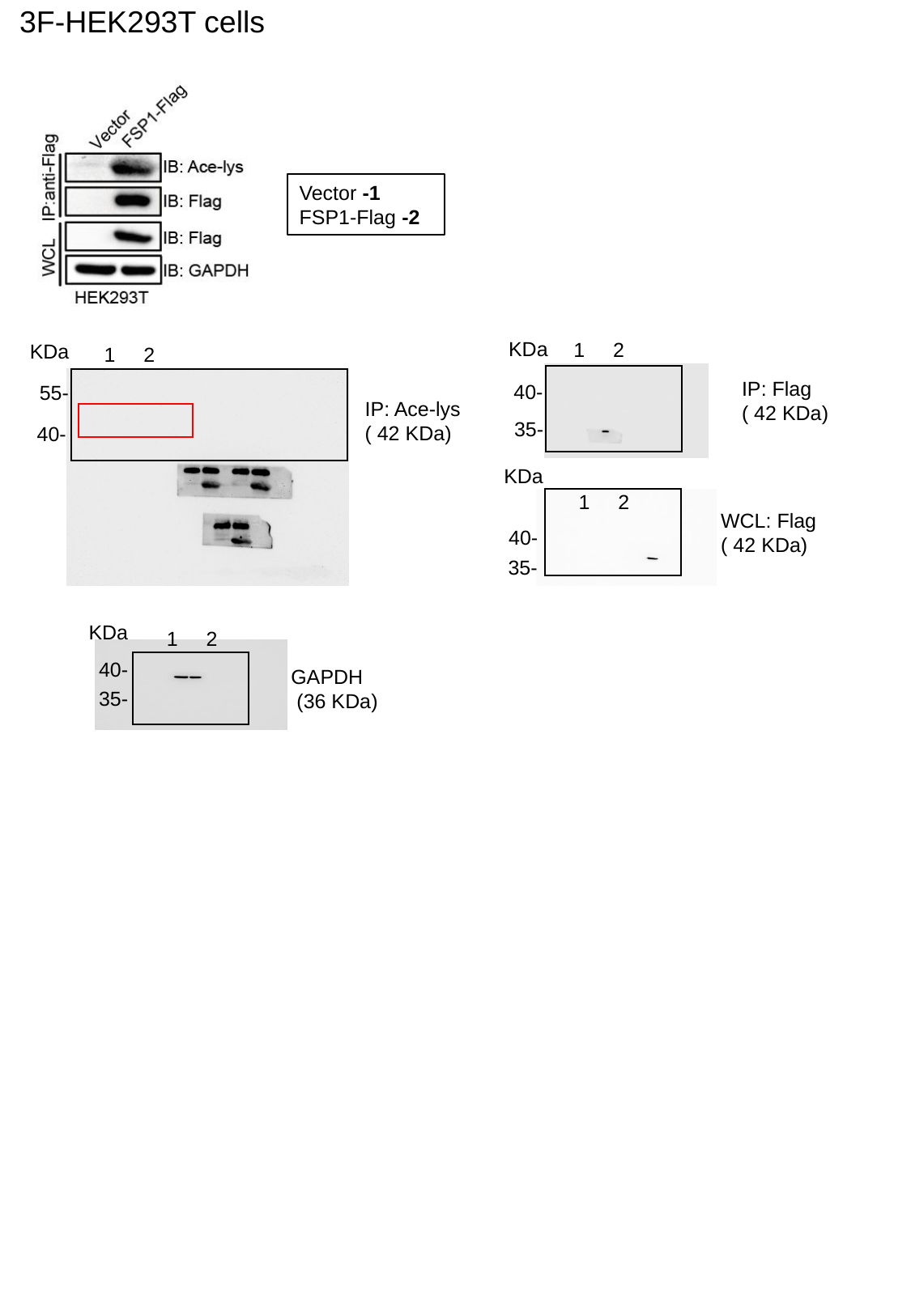

3F-HEK293T cells
Vector -1
FSP1-Flag -2
KDa
 1 2
KDa
 1 2
IP: Flag
( 42 KDa)
40-
55-
IP: Ace-lys
( 42 KDa)
35-
40-
KDa
 1 2
WCL: Flag
( 42 KDa)
40-
35-
KDa
 1 2
40-
GAPDH
 (36 KDa)
35-

Supplement: Supplementary file 5 — Source data Fig. 3 [file 44318_2025_369_MOESM5_ESM.zip › Figure 3/3F/3F-HEK293T-WB.pptx]

## Slide 1
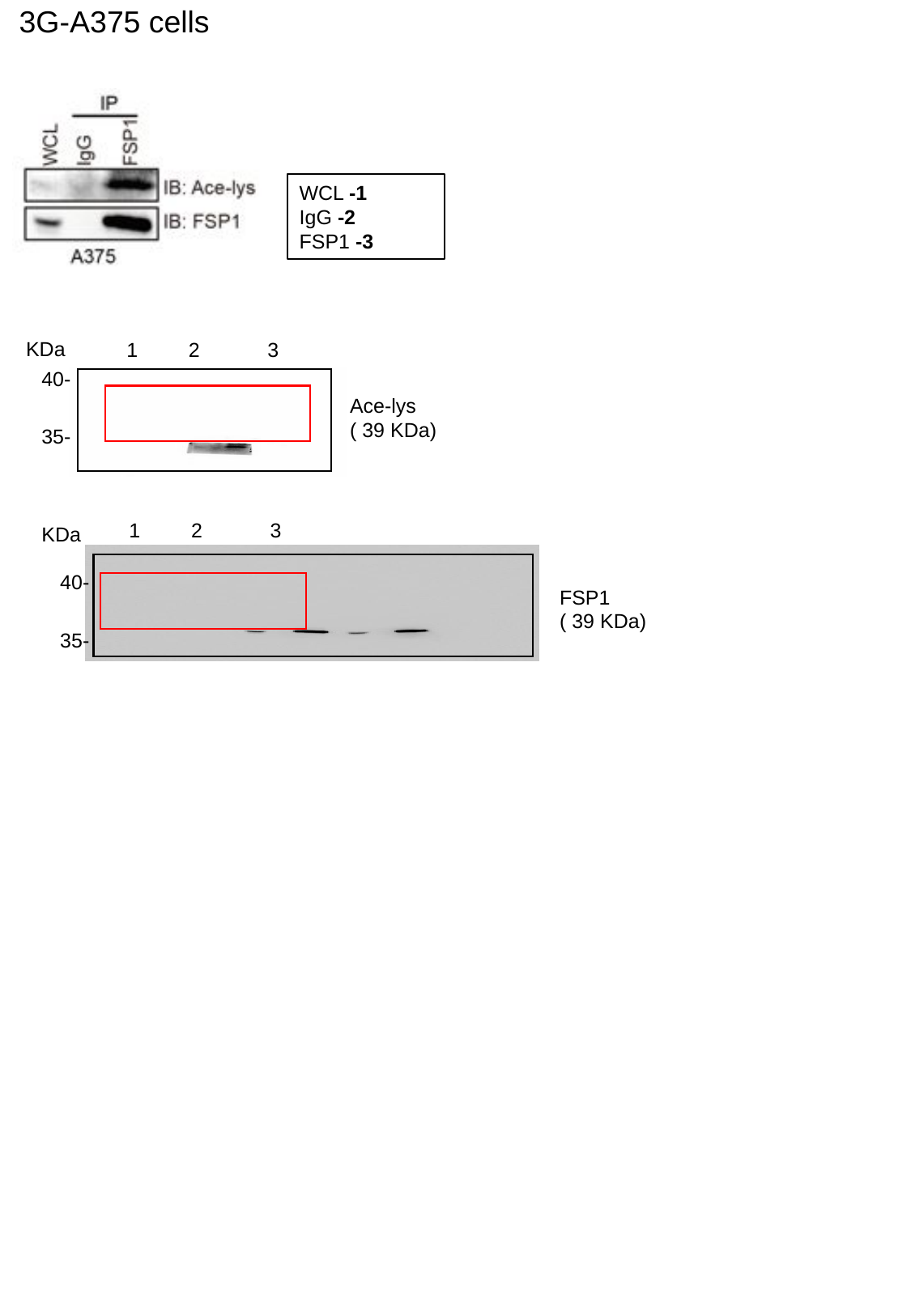

3G-A375 cells
WCL -1
IgG -2
FSP1 -3
KDa
 1 2 3
40-
Ace-lys
( 39 KDa)
35-
 1 2 3
KDa
40-
FSP1
( 39 KDa)
35-

Supplement: Supplementary file 5 — Source data Fig. 3 [file 44318_2025_369_MOESM5_ESM.zip › Figure 3/3G/3G-A375-WB.pptx]

## Slide 1
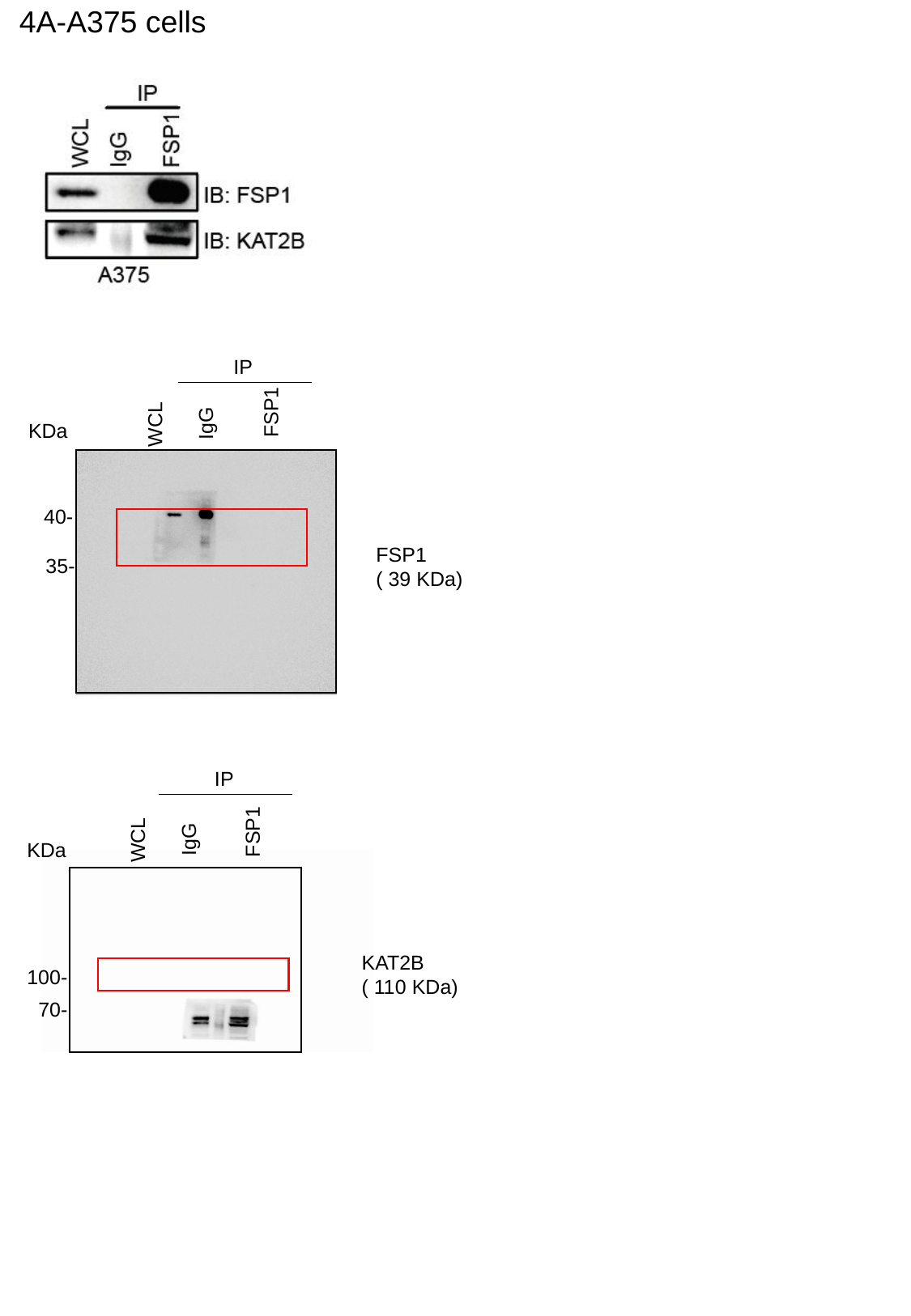

4A-A375 cells
IP
FSP1
IgG
WCL
KDa
40-
FSP1
( 39 KDa)
35-
IP
FSP1
IgG
WCL
KDa
KAT2B
( 110 KDa)
100-
70-

Supplement: Supplementary file 6 — Source data Fig. 4 [file 44318_2025_369_MOESM6_ESM.zip › Figure 4/4A/4A-A375-WB.pptx]

## Slide 1
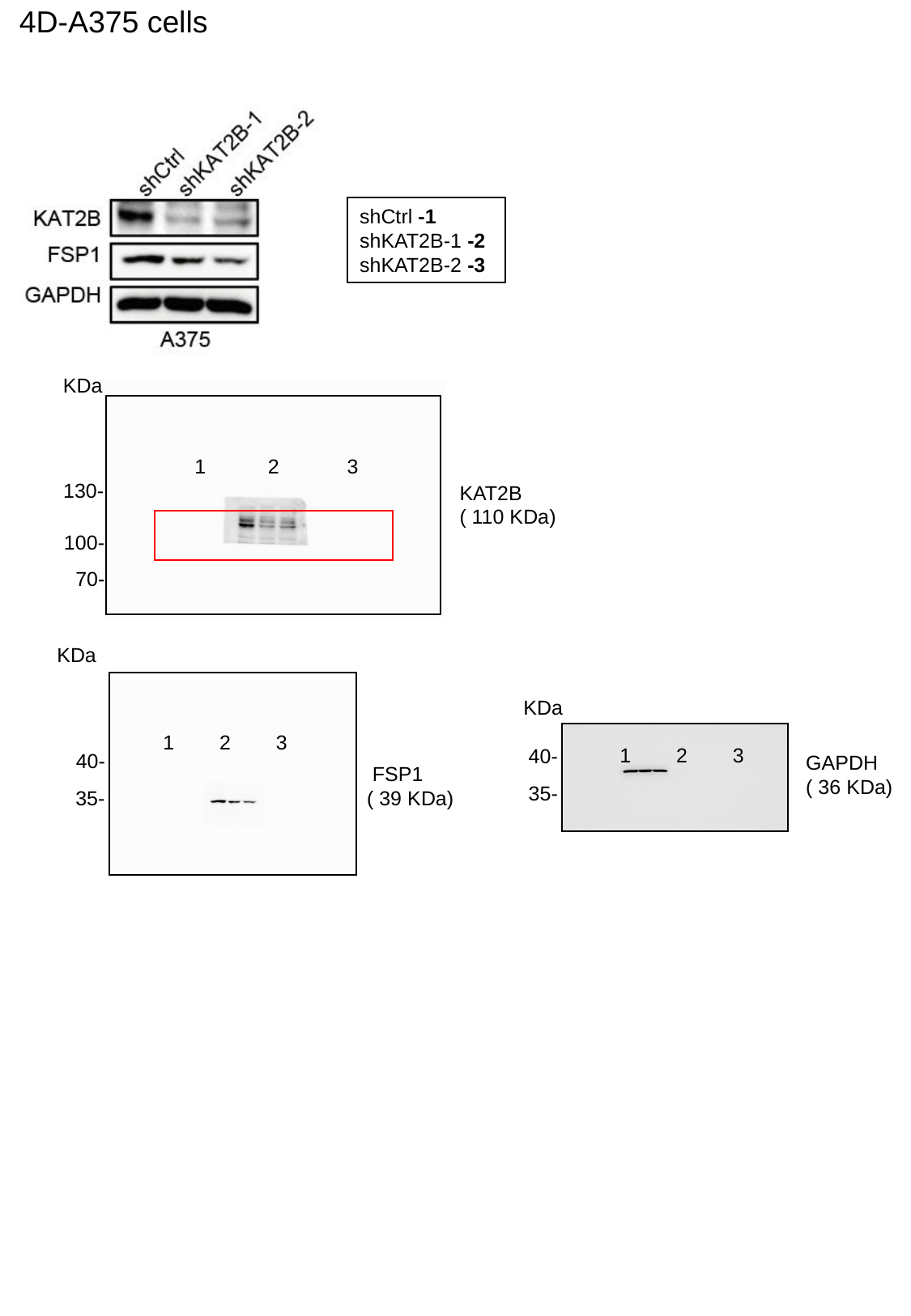

4D-A375 cells
shCtrl -1
shKAT2B-1 -2
shKAT2B-2 -3
KDa
 1 2 3
130-
KAT2B
( 110 KDa)
100-
70-
KDa
KDa
 1 2 3
 1 2 3
40-
40-
GAPDH
( 36 KDa)
 FSP1
( 39 KDa)
35-
35-

Supplement: Supplementary file 6 — Source data Fig. 4 [file 44318_2025_369_MOESM6_ESM.zip › Figure 4/4D/4D-A375-WB.pptx]

## Slide 1
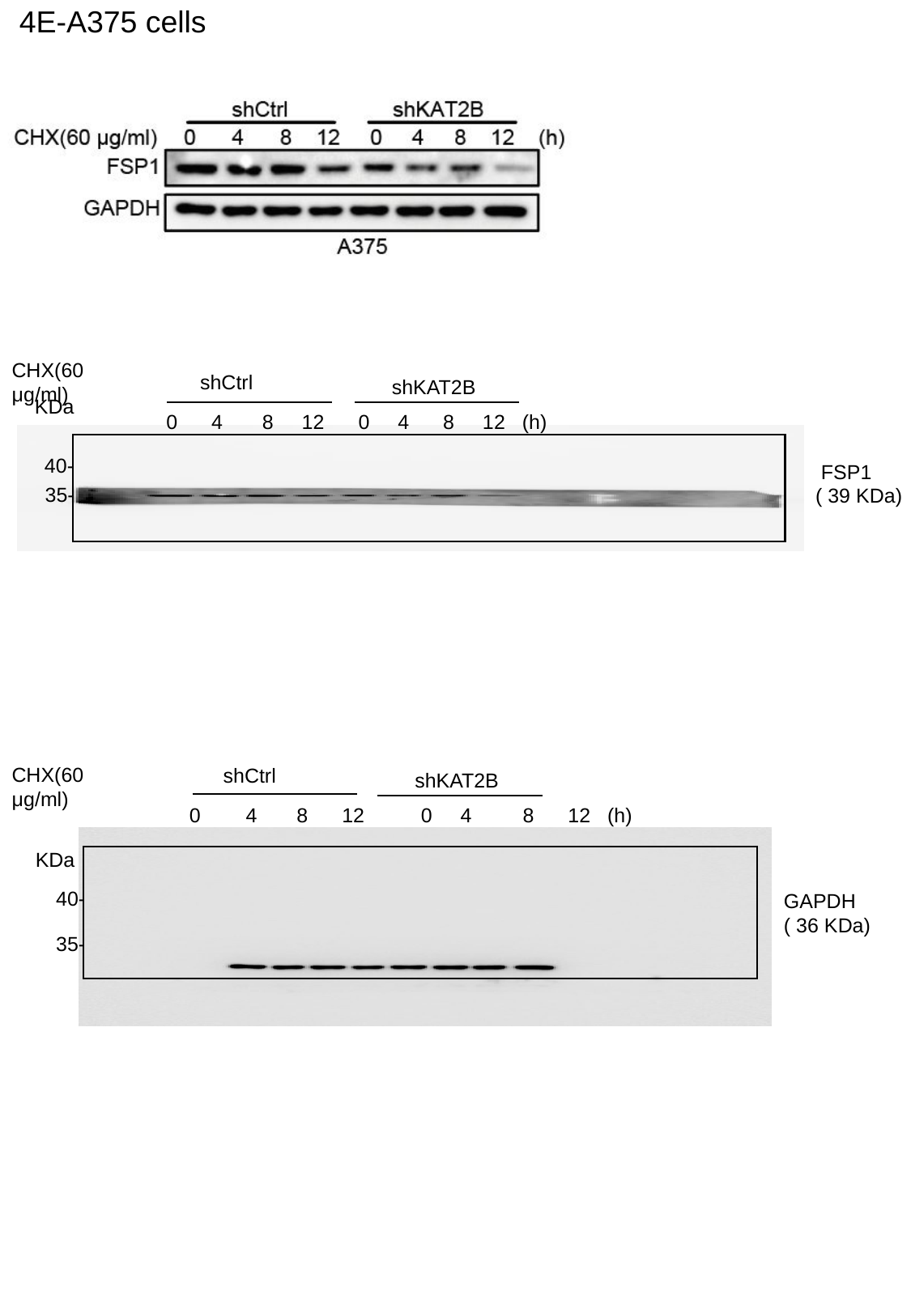

4E-A375 cells
CHX(60 μg/ml)
shCtrl
shKAT2B
KDa
 0 4 8 12 0 4 8 12 (h)
40-
 FSP1
( 39 KDa)
35-
CHX(60 μg/ml)
shCtrl
shKAT2B
 0 4 8 12 0 4 8 12 (h)
KDa
40-
GAPDH
( 36 KDa)
35-

Supplement: Supplementary file 6 — Source data Fig. 4 [file 44318_2025_369_MOESM6_ESM.zip › Figure 4/4E/4E-A375-WB.pptx]

## Slide 1
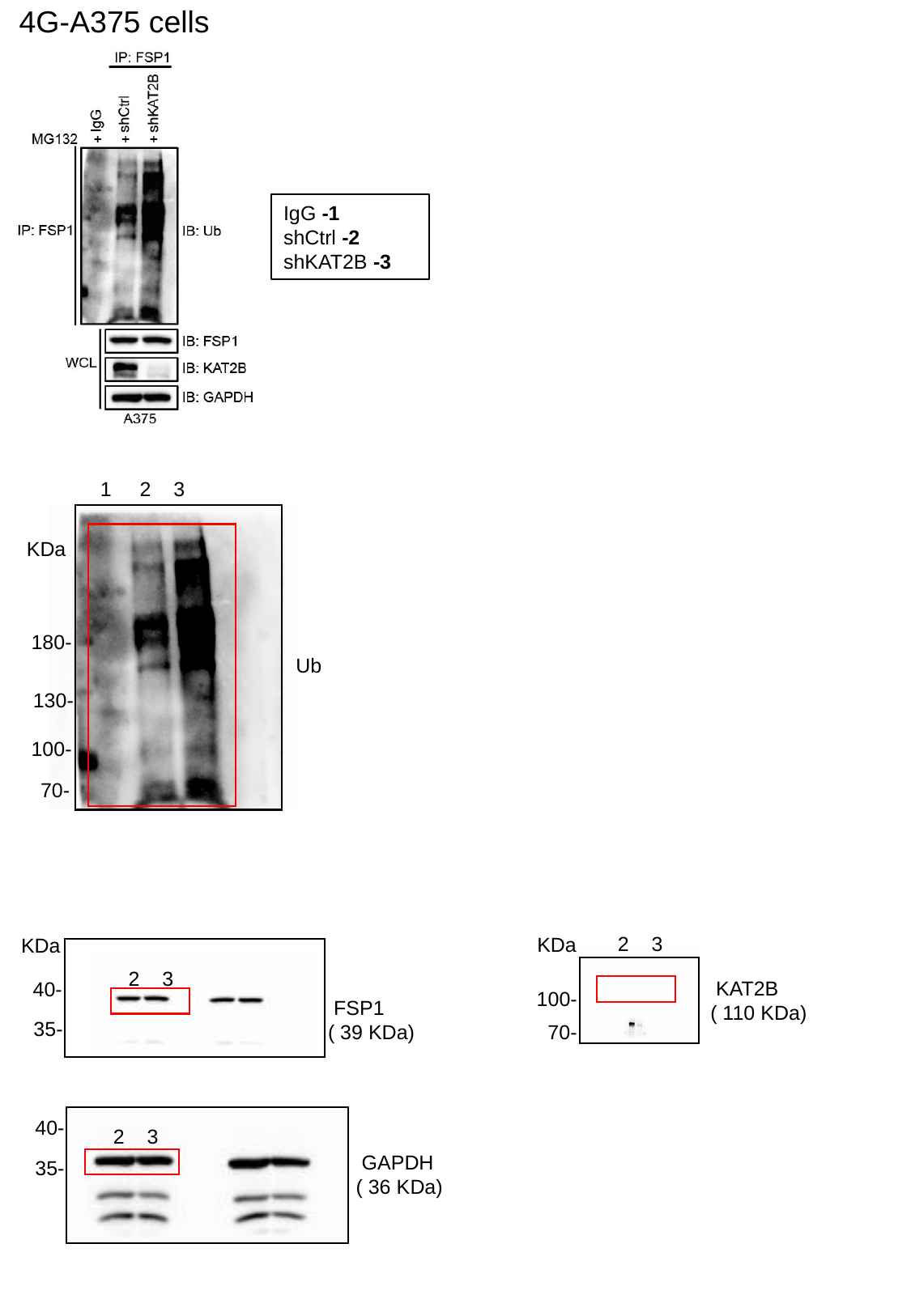

4G-A375 cells
IgG -1
shCtrl -2
shKAT2B -3
 1 2 3
KDa
180-
Ub
130-
100-
70-
2 3
KDa
KDa
2 3
 KAT2B
( 110 KDa)
40-
100-
 FSP1
( 39 KDa)
35-
70-
40-
2 3
 GAPDH
( 36 KDa)
35-

Supplement: Supplementary file 6 — Source data Fig. 4 [file 44318_2025_369_MOESM6_ESM.zip › Figure 4/4G/4G-A375-WB.pptx]

## Slide 1
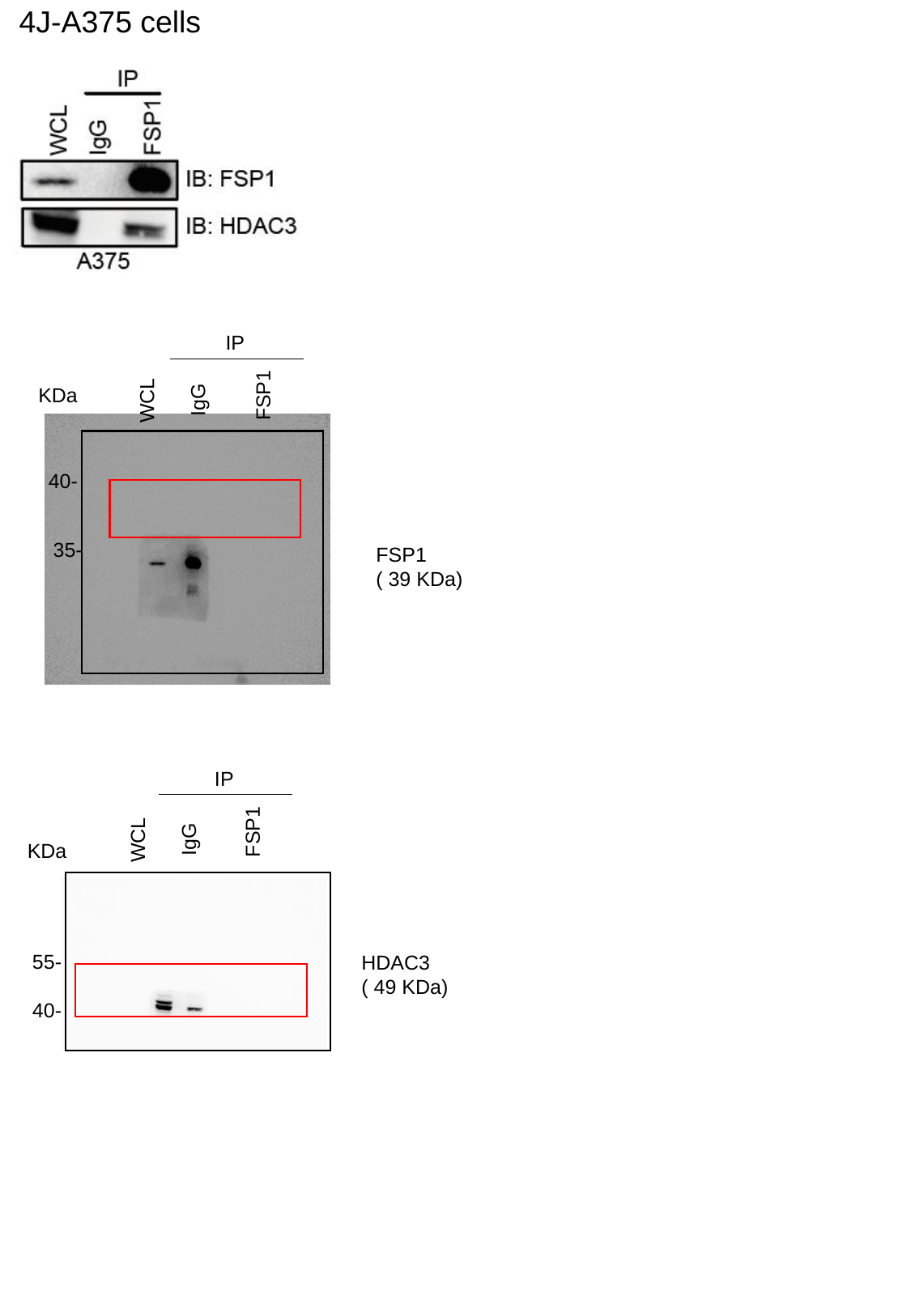

4J-A375 cells
IP
FSP1
IgG
WCL
KDa
40-
35-
FSP1
( 39 KDa)
IP
FSP1
IgG
WCL
KDa
55-
HDAC3
( 49 KDa)
40-

Supplement: Supplementary file 6 — Source data Fig. 4 [file 44318_2025_369_MOESM6_ESM.zip › Figure 4/4J/4J-A375-WB.pptx]

## Slide 1
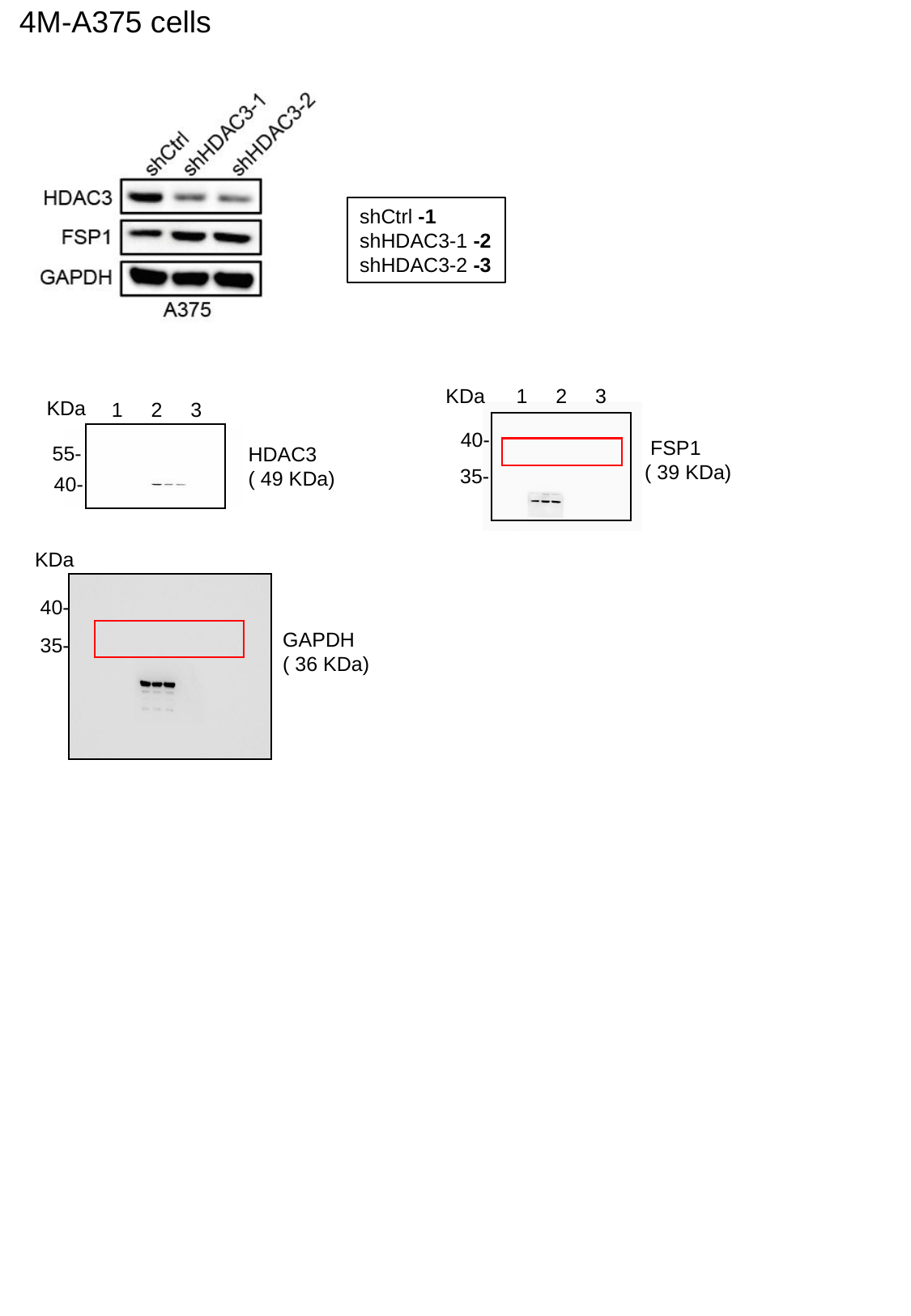

4M-A375 cells
shCtrl -1
shHDAC3-1 -2
shHDAC3-2 -3
 1 2 3
KDa
KDa
 1 2 3
40-
 FSP1
( 39 KDa)
55-
HDAC3
( 49 KDa)
35-
40-
KDa
40-
GAPDH
( 36 KDa)
35-

Supplement: Supplementary file 6 — Source data Fig. 4 [file 44318_2025_369_MOESM6_ESM.zip › Figure 4/4M/4M-A375-WB.pptx]

## Slide 1
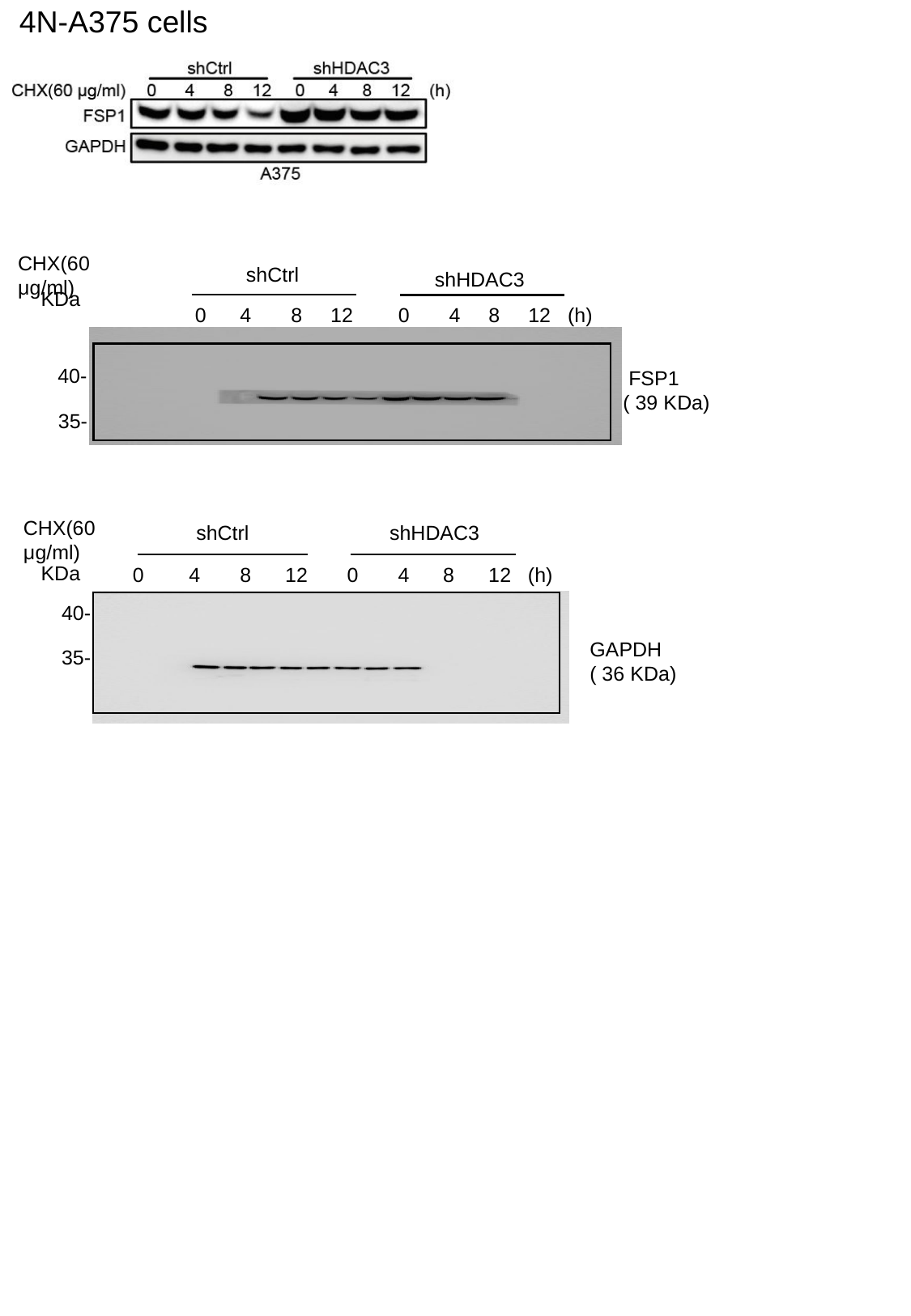

4N-A375 cells
CHX(60 μg/ml)
shCtrl
shHDAC3
KDa
 0 4 8 12 0 4 8 12 (h)
40-
 FSP1
( 39 KDa)
35-
CHX(60 μg/ml)
shHDAC3
shCtrl
KDa
 0 4 8 12 0 4 8 12 (h)
40-
GAPDH
( 36 KDa)
35-

Supplement: Supplementary file 6 — Source data Fig. 4 [file 44318_2025_369_MOESM6_ESM.zip › Figure 4/4N/4N-A375-WB.pptx]

## Slide 1
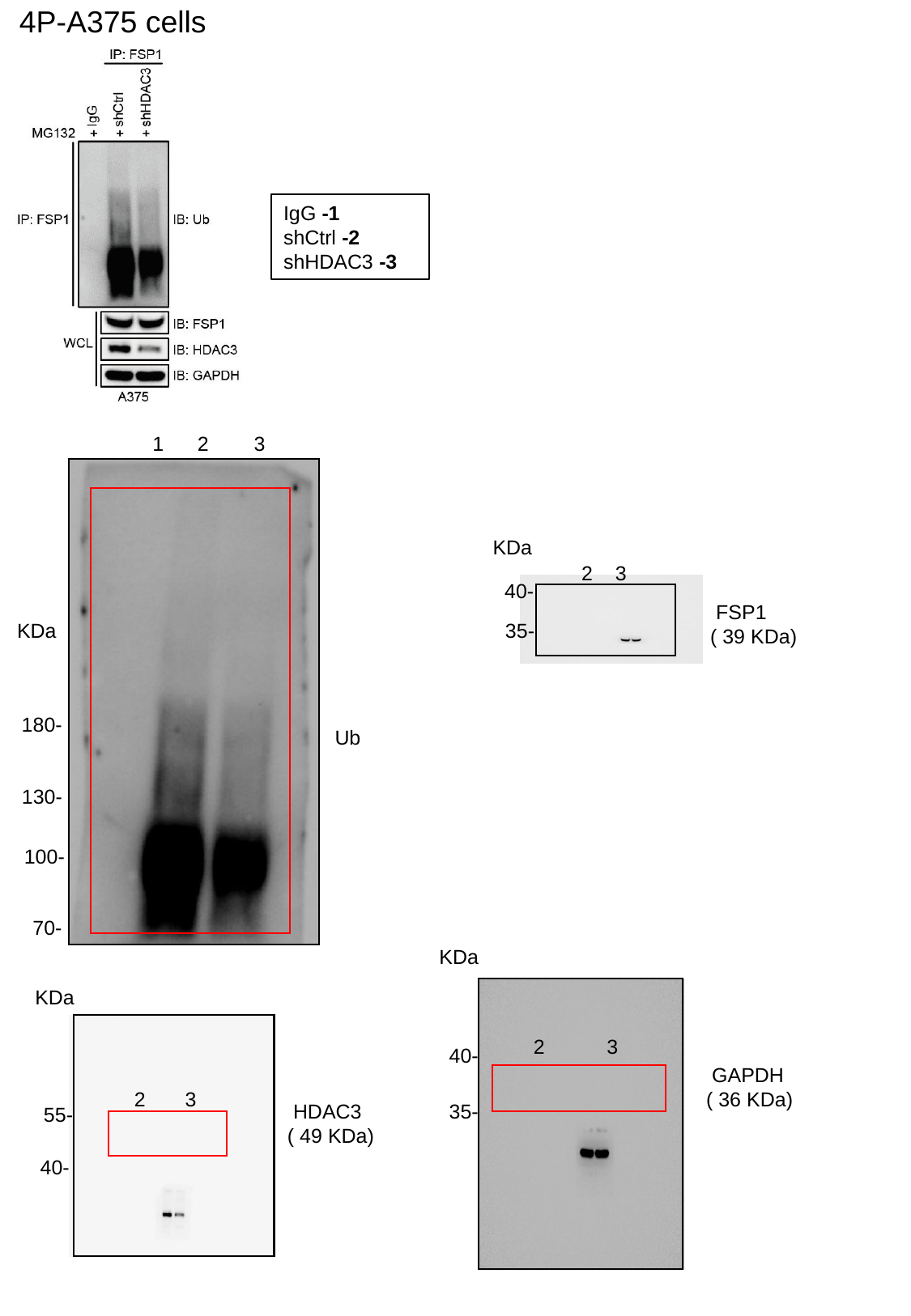

4P-A375 cells
IgG -1
shCtrl -2
shHDAC3 -3
 1 2 3
KDa
2 3
40-
 FSP1
( 39 KDa)
KDa
35-
180-
Ub
130-
100-
70-
KDa
KDa
2 3
40-
 GAPDH
( 36 KDa)
2 3
 HDAC3
( 49 KDa)
35-
55-
40-

Supplement: Supplementary file 6 — Source data Fig. 4 [file 44318_2025_369_MOESM6_ESM.zip › Figure 4/4P/4P-A375-WB.pptx]

## Slide 1
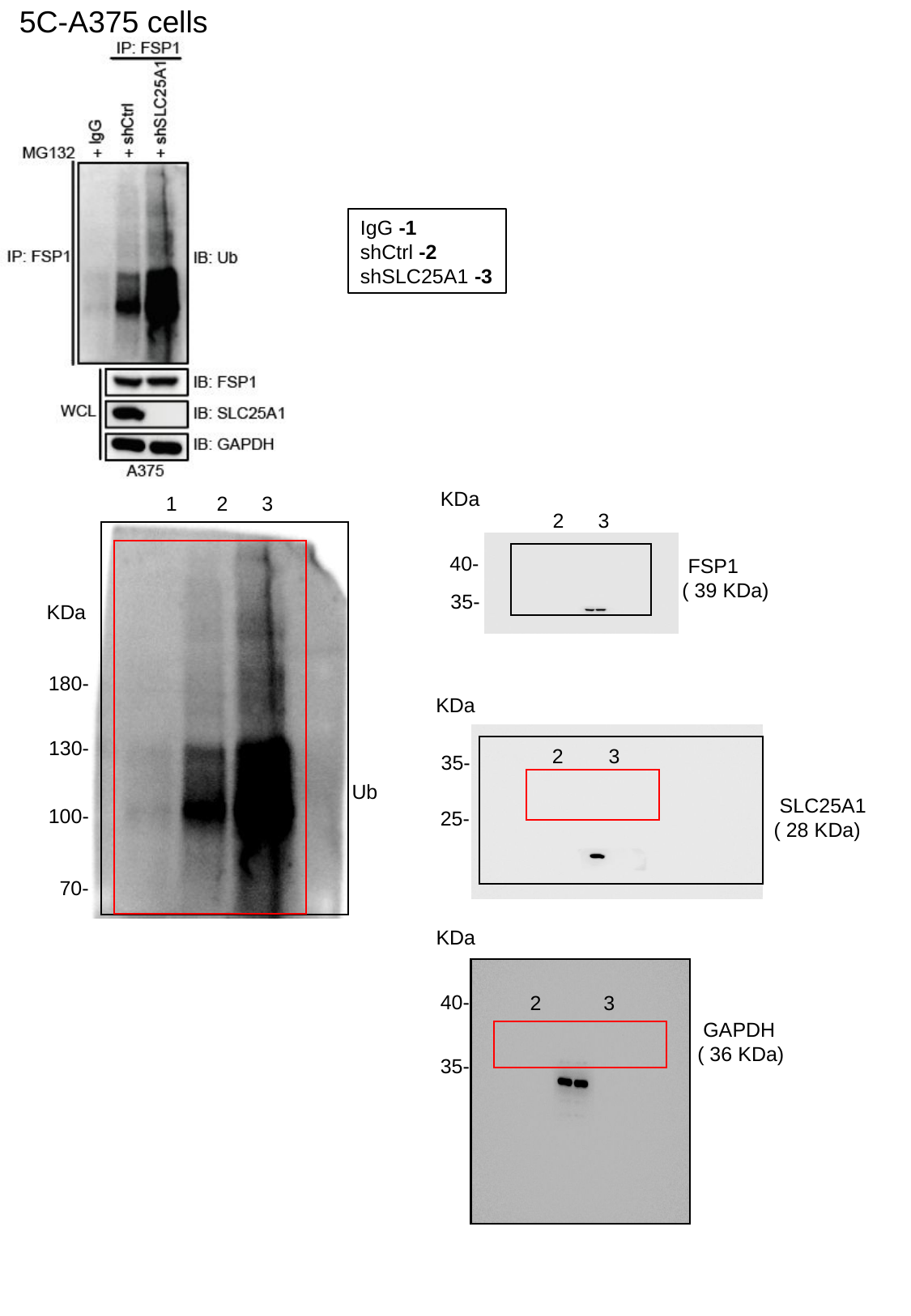

5C-A375 cells
IgG -1
shCtrl -2
shSLC25A1 -3
KDa
 1 2 3
2 3
40-
 FSP1
( 39 KDa)
35-
KDa
180-
KDa
130-
2 3
35-
Ub
 SLC25A1
( 28 KDa)
100-
25-
70-
KDa
40-
2 3
 GAPDH
( 36 KDa)
35-

Supplement: Supplementary file 7 — Source data Fig. 5 [file 44318_2025_369_MOESM7_ESM.zip › Figure 5/5C/5C-A375-WB.pptx]

## Slide 1
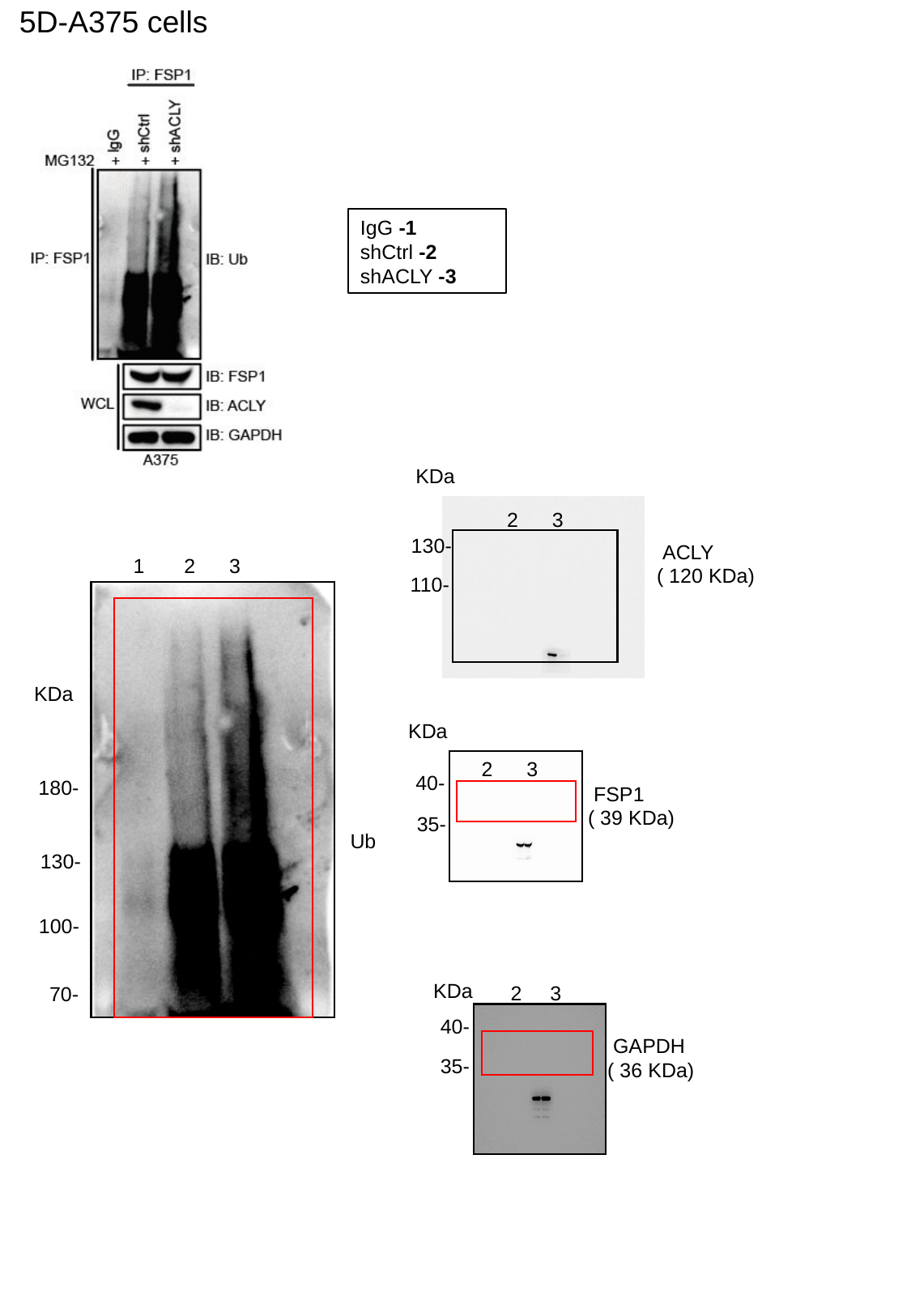

5D-A375 cells
IgG -1
shCtrl -2
shACLY -3
KDa
2 3
130-
 ACLY
( 120 KDa)
 1 2 3
110-
KDa
KDa
 2 3
40-
180-
 FSP1
( 39 KDa)
35-
Ub
130-
100-
KDa
2 3
70-
40-
 GAPDH
( 36 KDa)
35-

Supplement: Supplementary file 7 — Source data Fig. 5 [file 44318_2025_369_MOESM7_ESM.zip › Figure 5/5D/5D-A375-WB.pptx]

## Slide 1
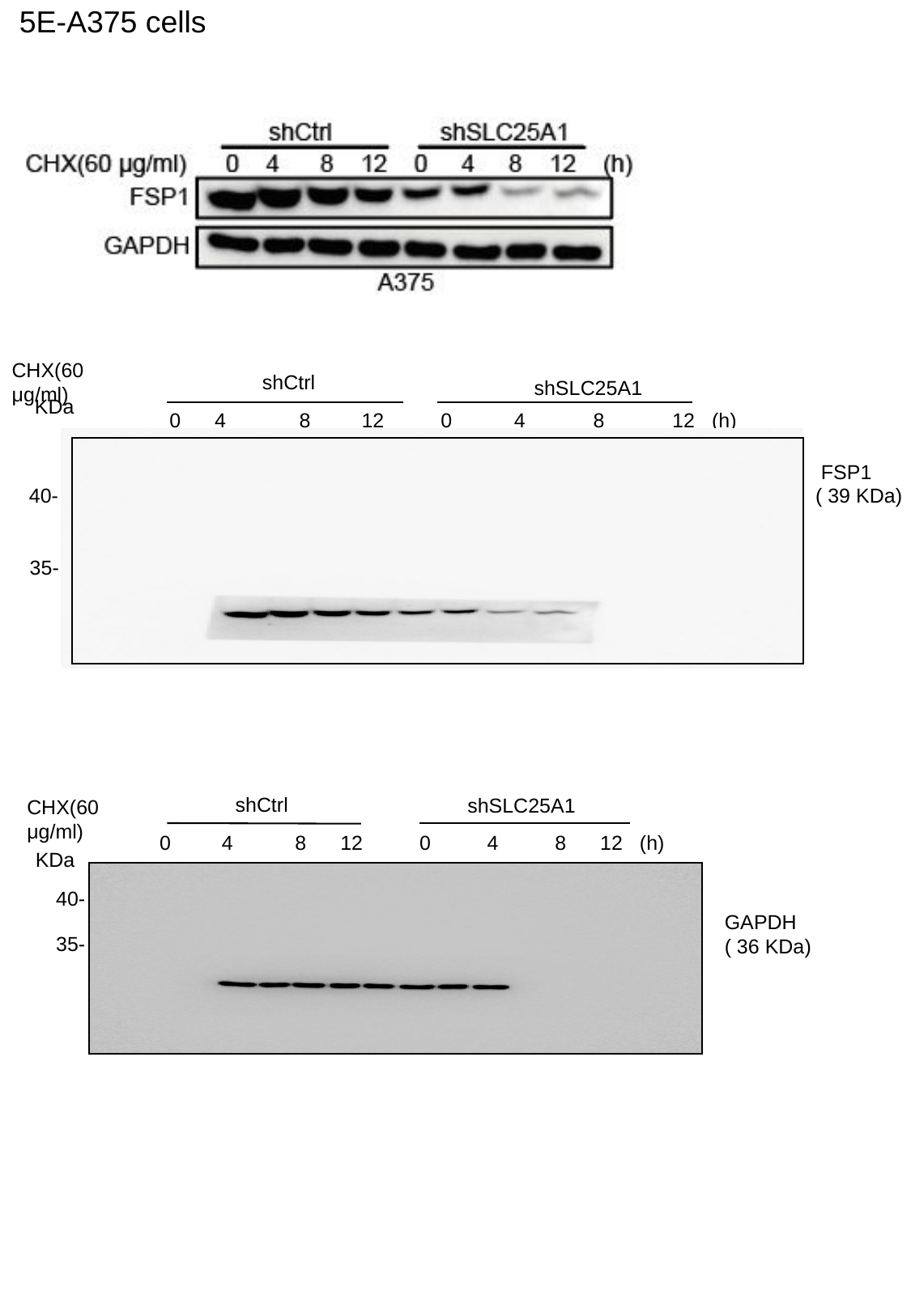

5E-A375 cells
CHX(60 μg/ml)
shCtrl
shSLC25A1
KDa
 0 4 8 12 0 4 8 12 (h)
 FSP1
( 39 KDa)
40-
35-
shCtrl
shSLC25A1
CHX(60 μg/ml)
 0 4 8 12 0 4 8 12 (h)
KDa
40-
GAPDH
( 36 KDa)
35-

Supplement: Supplementary file 7 — Source data Fig. 5 [file 44318_2025_369_MOESM7_ESM.zip › Figure 5/5E/5E-A375-WB.pptx]

## Slide 1
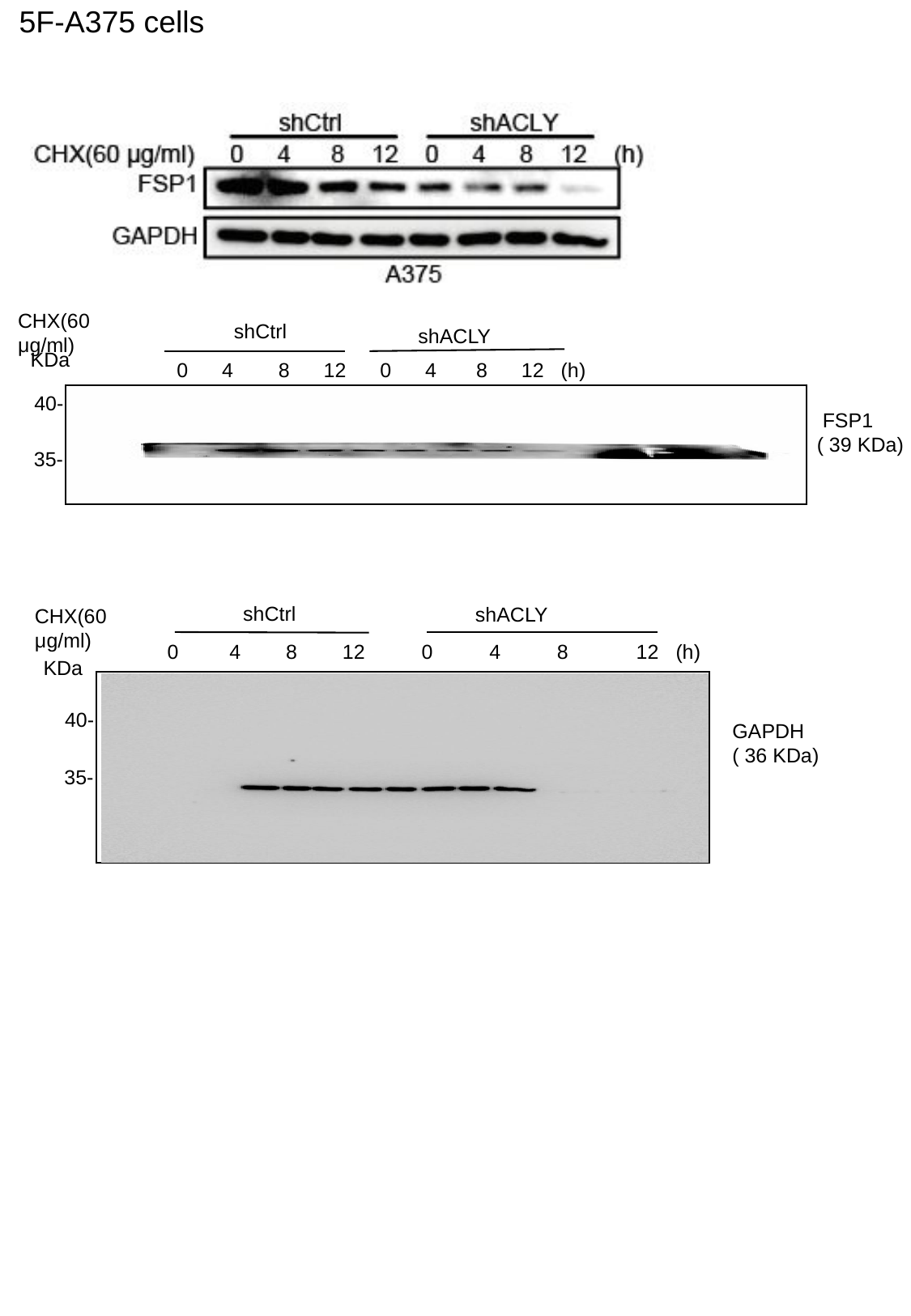

5F-A375 cells
CHX(60 μg/ml)
shCtrl
shACLY
KDa
 0 4 8 12 0 4 8 12 (h)
40-
 FSP1
( 39 KDa)
35-
shCtrl
shACLY
CHX(60 μg/ml)
 0 4 8 12 0 4 8 12 (h)
KDa
40-
GAPDH
( 36 KDa)
35-

Supplement: Supplementary file 7 — Source data Fig. 5 [file 44318_2025_369_MOESM7_ESM.zip › Figure 5/5F/5F-A375-WB.pptx]

## Slide 1
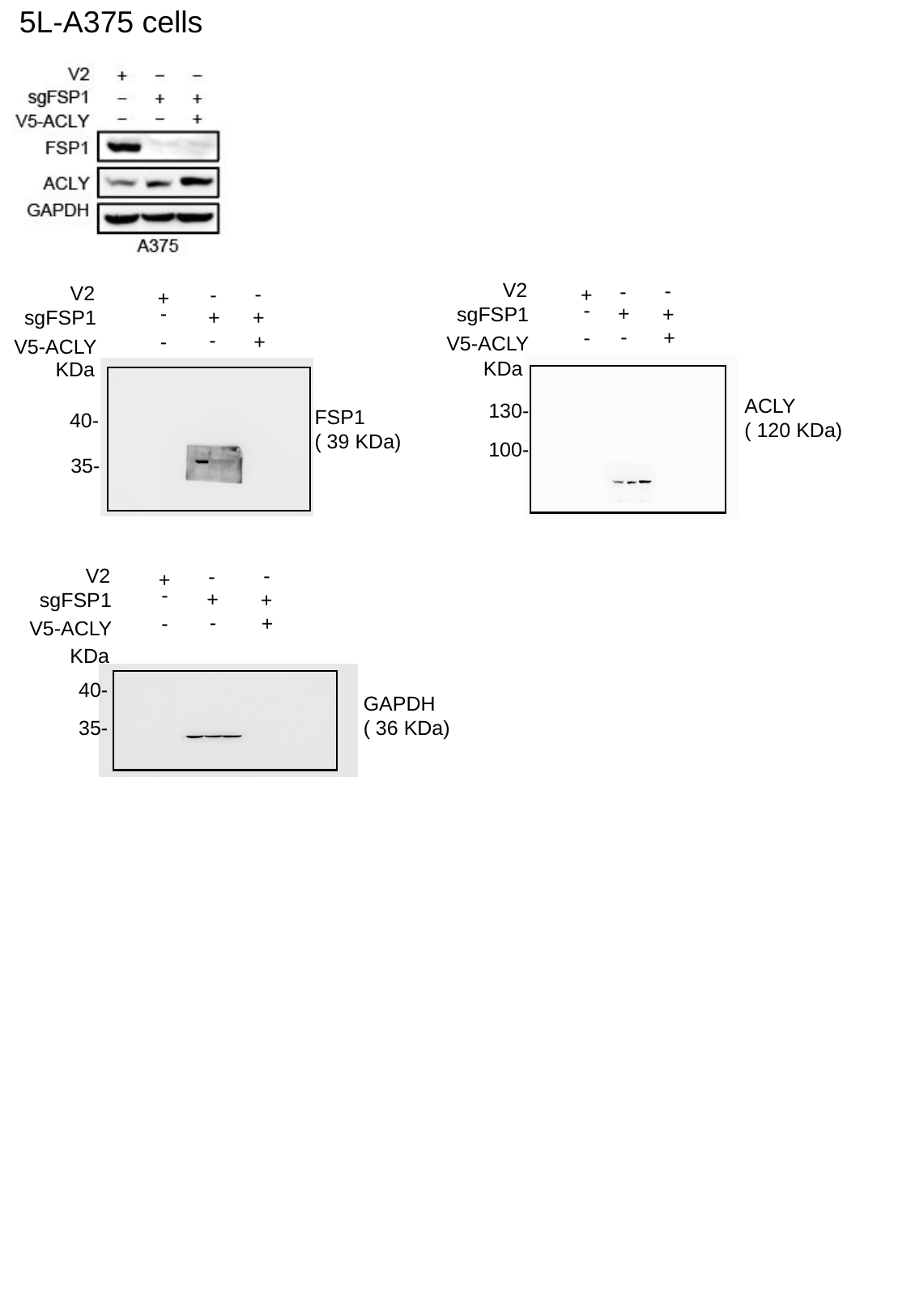

5L-A375 cells
V2
-
-
V2
-
+
-
+
-
-
+
+
sgFSP1
+
+
sgFSP1
-
+
-
-
+
-
V5-ACLY
V5-ACLY
KDa
KDa
ACLY
( 120 KDa)
130-
FSP1
( 39 KDa)
40-
100-
35-
V2
-
-
+
-
+
+
sgFSP1
-
+
-
V5-ACLY
KDa
40-
GAPDH
( 36 KDa)
35-

Supplement: Supplementary file 7 — Source data Fig. 5 [file 44318_2025_369_MOESM7_ESM.zip › Figure 5/5L/5L-A375-WB.pptx]

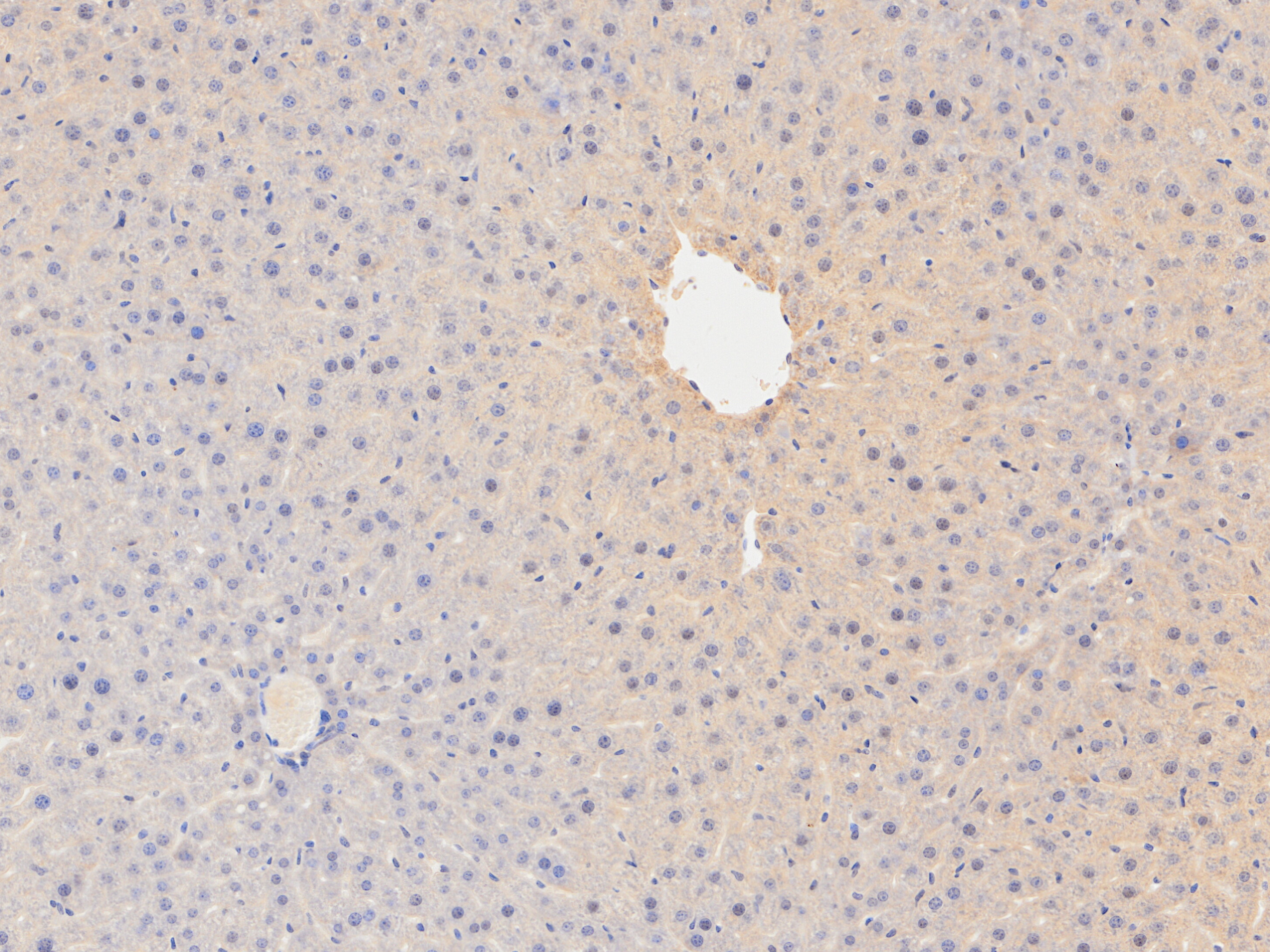

Supplement: Supplementary file 8 — Source data Fig. 6 [file 44318_2025_369_MOESM8_ESM.zip › Figure 6/6G/Fsp1/BTA+IRI+Lip-1.png]

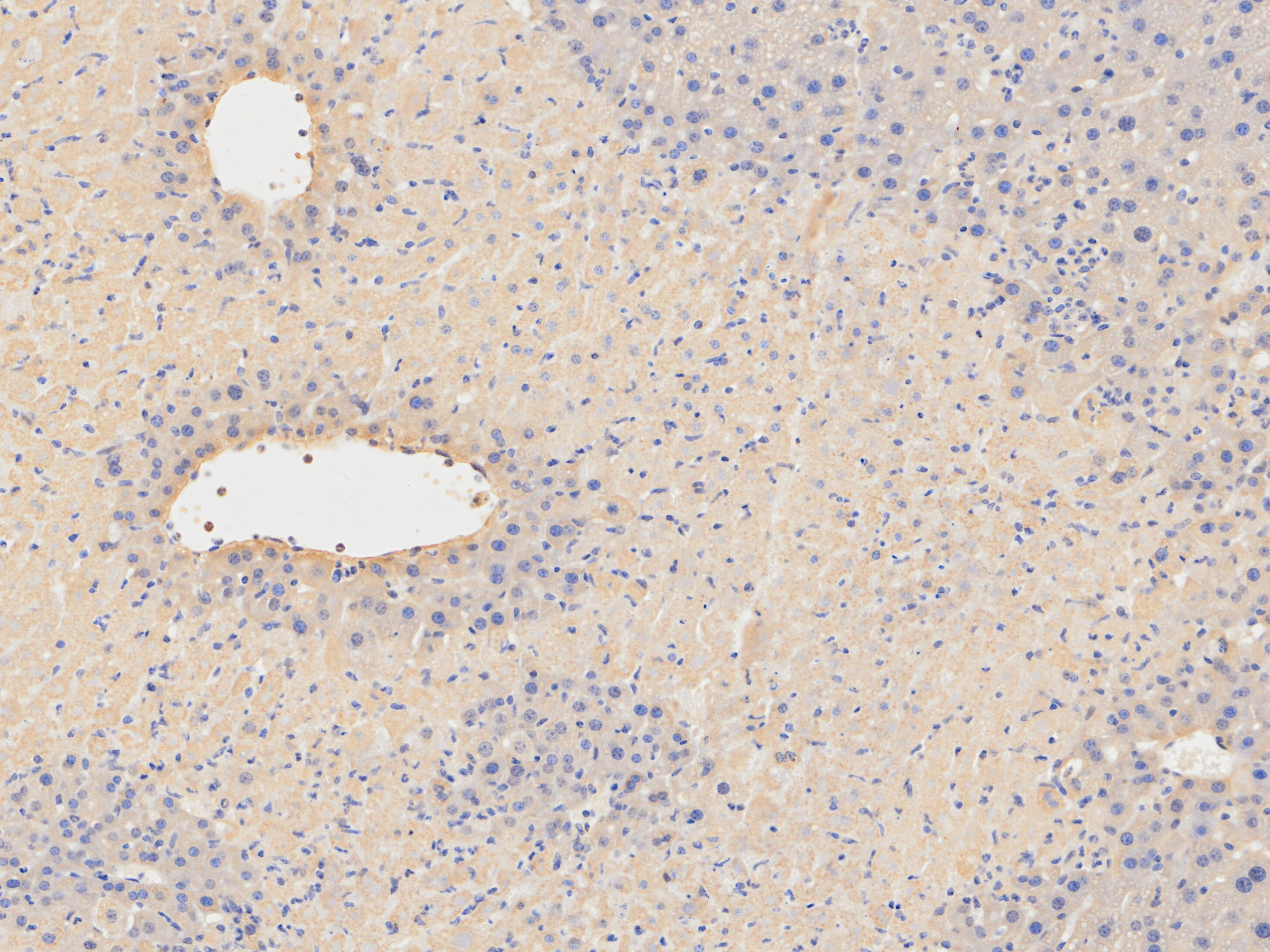

Supplement: Supplementary file 8 — Source data Fig. 6 [file 44318_2025_369_MOESM8_ESM.zip › Figure 6/6G/Fsp1/BTA+IRI.png]

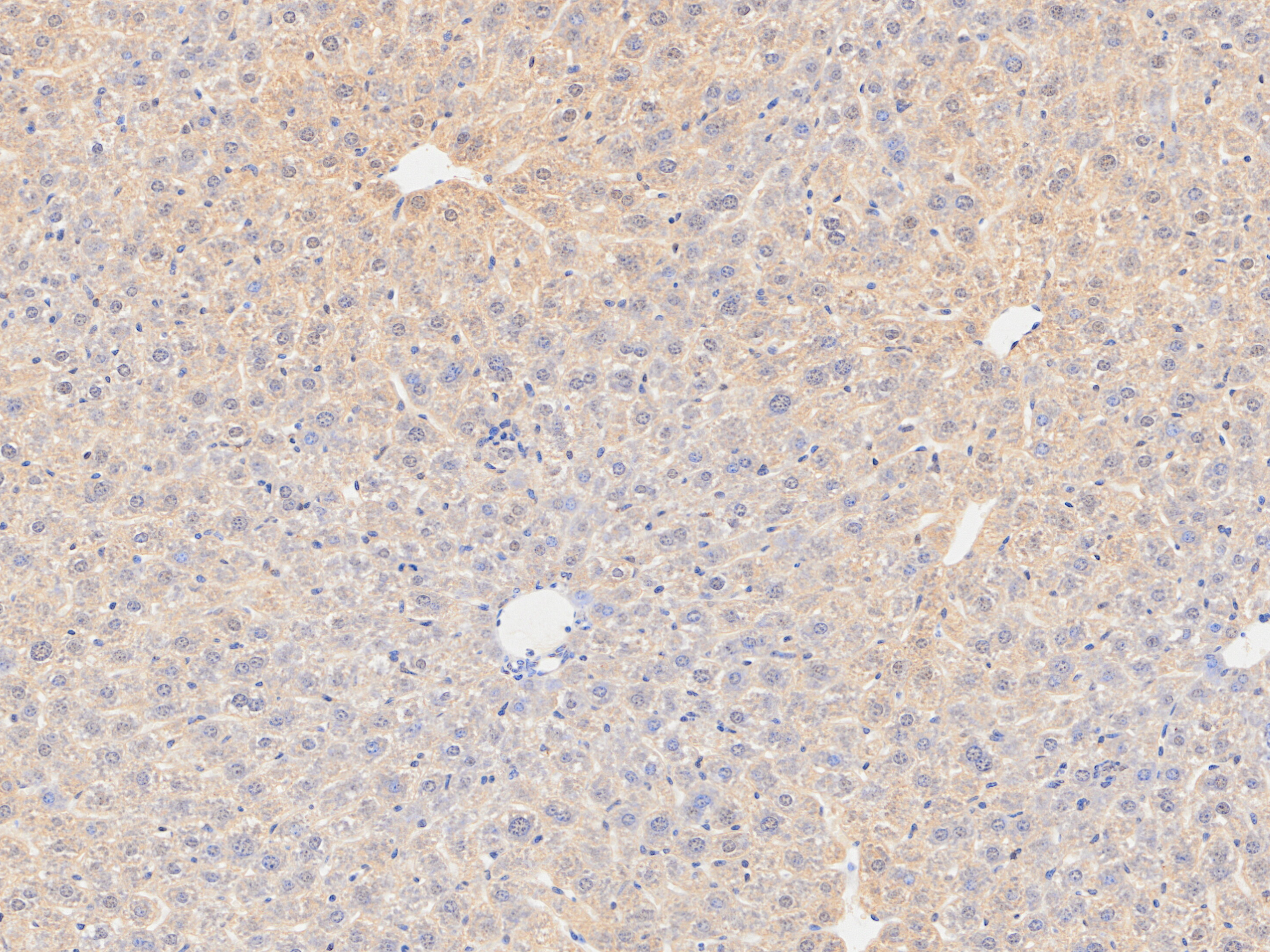

Supplement: Supplementary file 8 — Source data Fig. 6 [file 44318_2025_369_MOESM8_ESM.zip › Figure 6/6G/Fsp1/BTA+Sham.png]

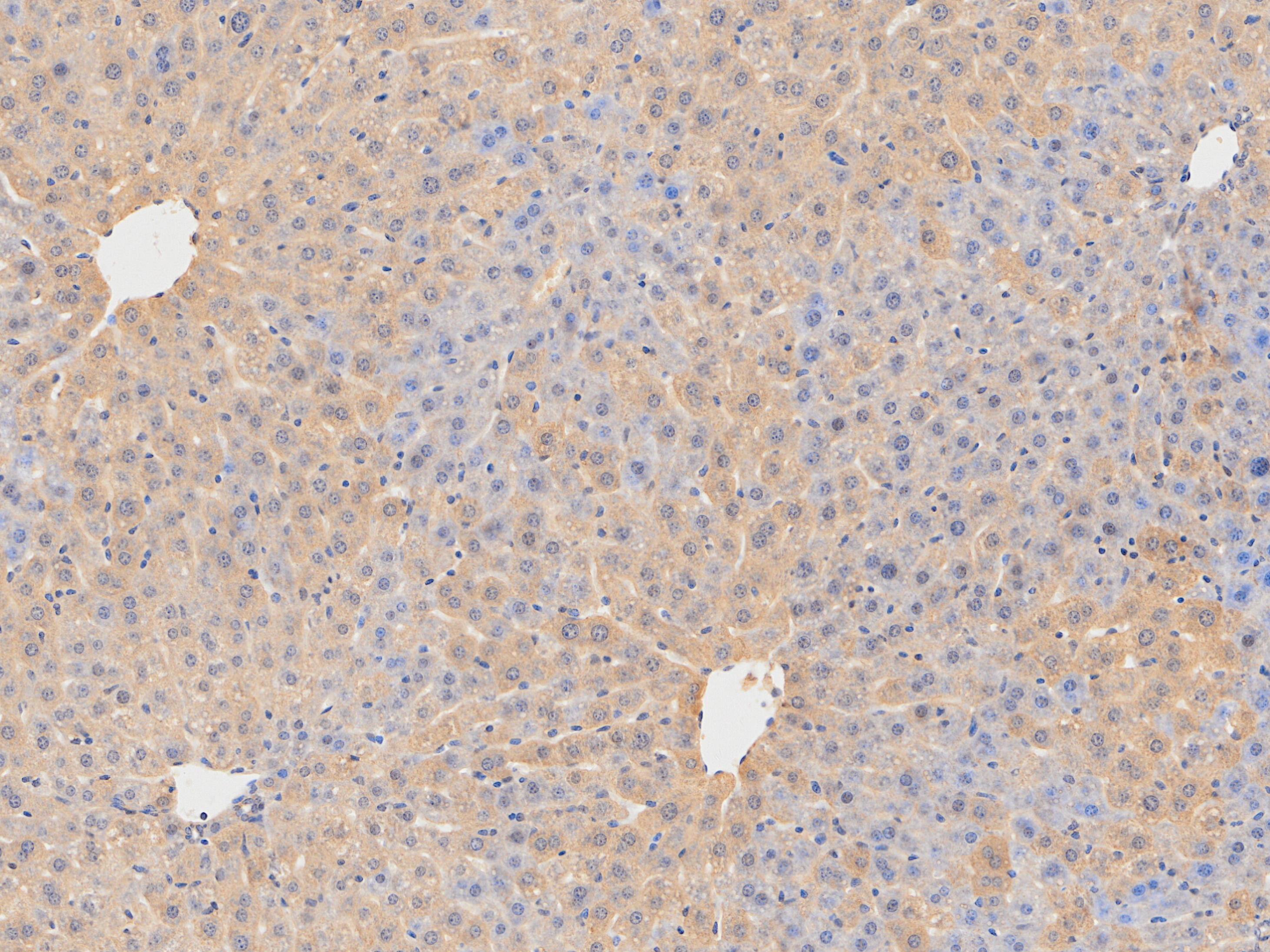

Supplement: Supplementary file 8 — Source data Fig. 6 [file 44318_2025_369_MOESM8_ESM.zip › Figure 6/6G/Fsp1/Vehicle+IRI+Lip-1.png]

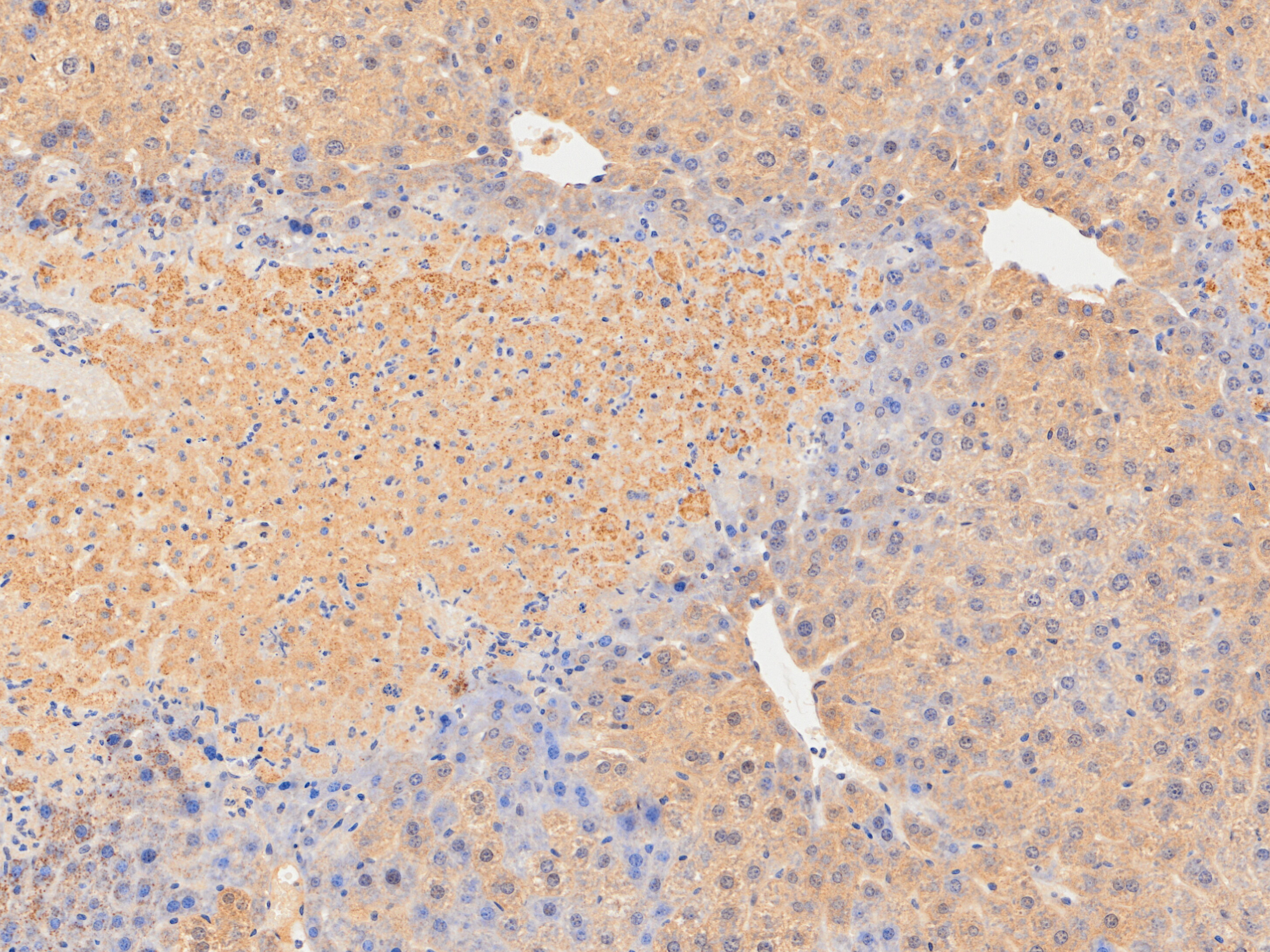

Supplement: Supplementary file 8 — Source data Fig. 6 [file 44318_2025_369_MOESM8_ESM.zip › Figure 6/6G/Fsp1/Vehicle+IRI.png]

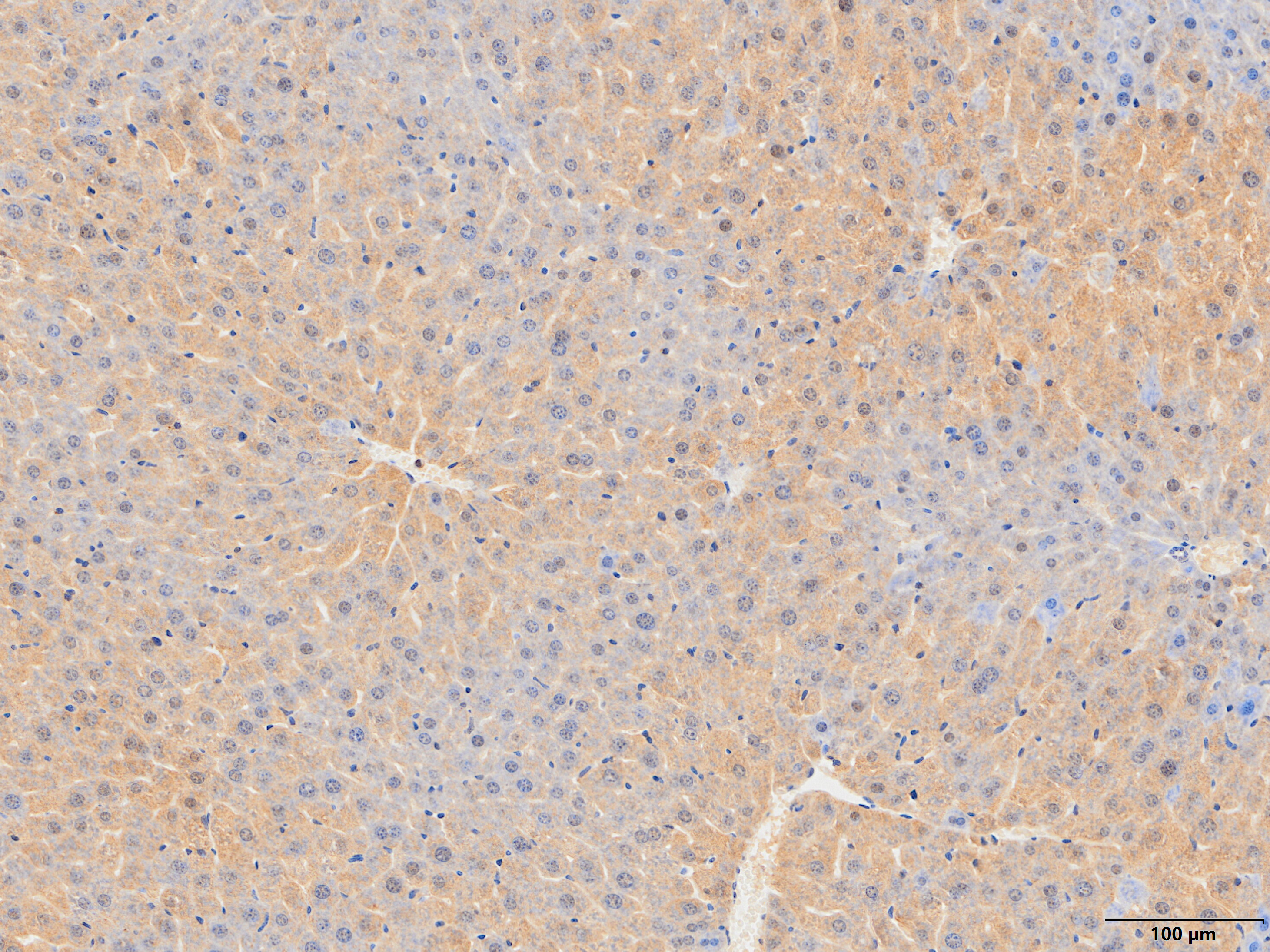

Supplement: Supplementary file 8 — Source data Fig. 6 [file 44318_2025_369_MOESM8_ESM.zip › Figure 6/6G/Fsp1/Vehicle.png]

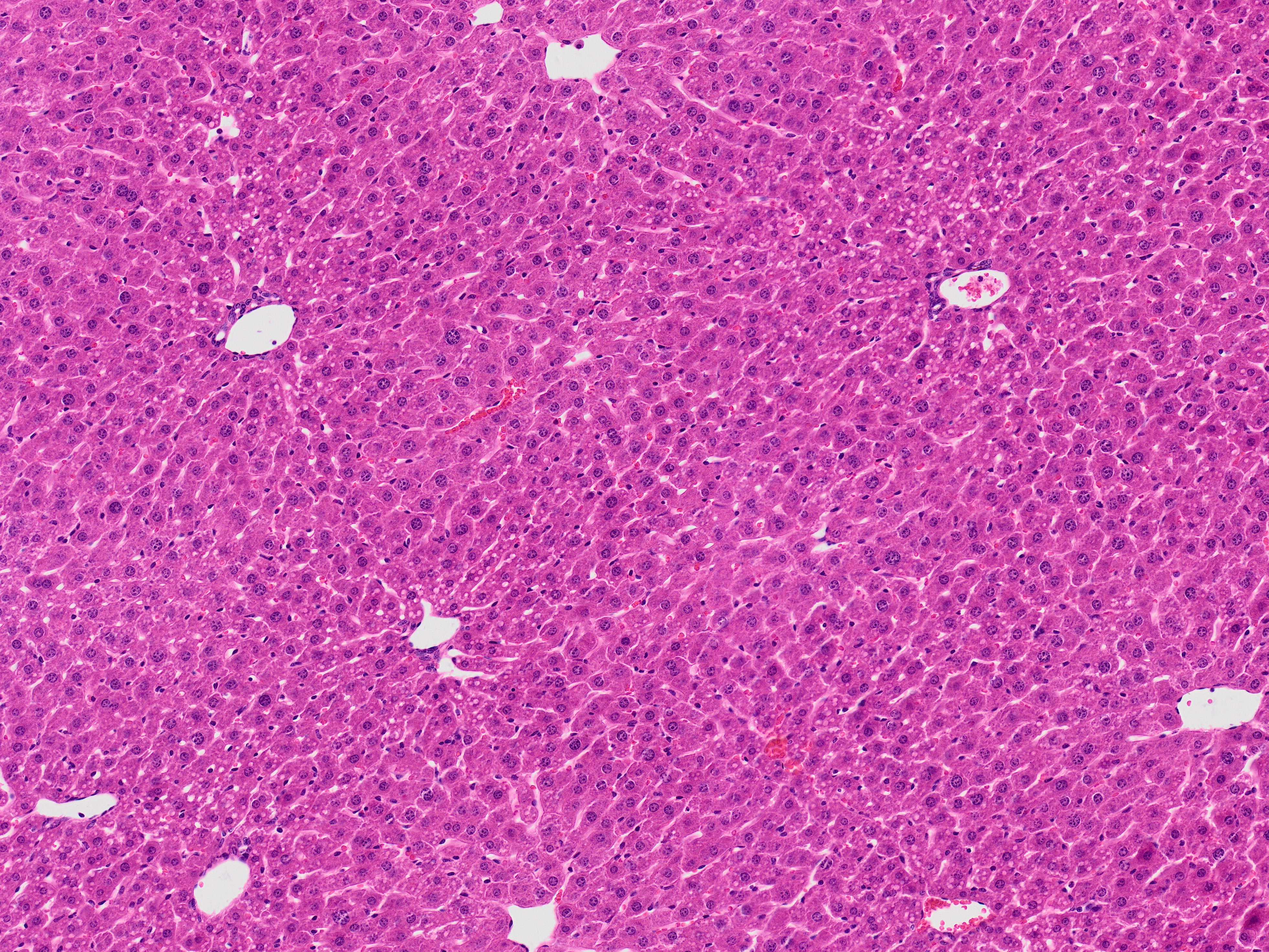

Supplement: Supplementary file 8 — Source data Fig. 6 [file 44318_2025_369_MOESM8_ESM.zip › Figure 6/6G/HE/BTA+IRI+Lip-1.png]

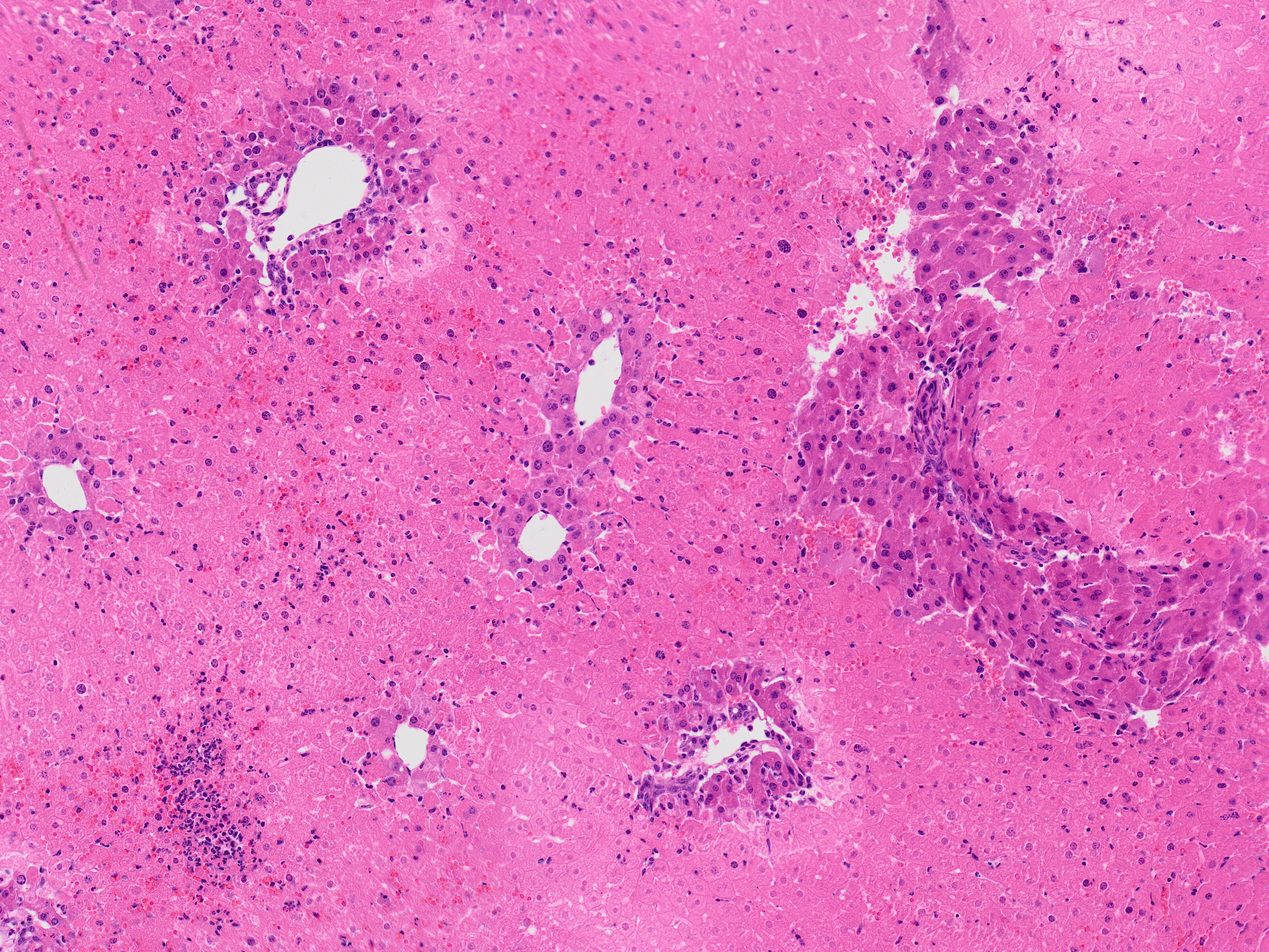

Supplement: Supplementary file 8 — Source data Fig. 6 [file 44318_2025_369_MOESM8_ESM.zip › Figure 6/6G/HE/BTA+IRI.png]

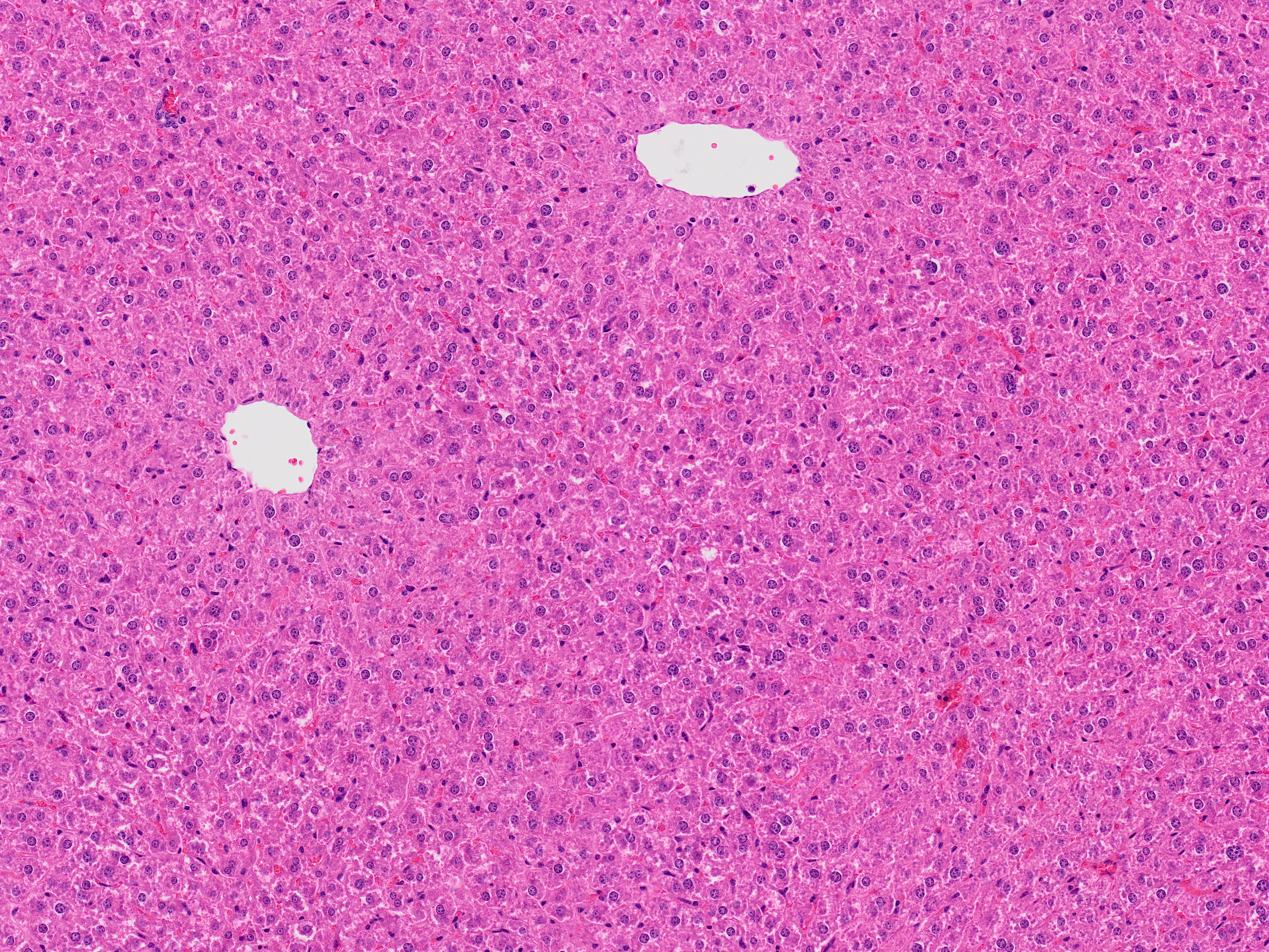

Supplement: Supplementary file 8 — Source data Fig. 6 [file 44318_2025_369_MOESM8_ESM.zip › Figure 6/6G/HE/BTA+Sham.png]

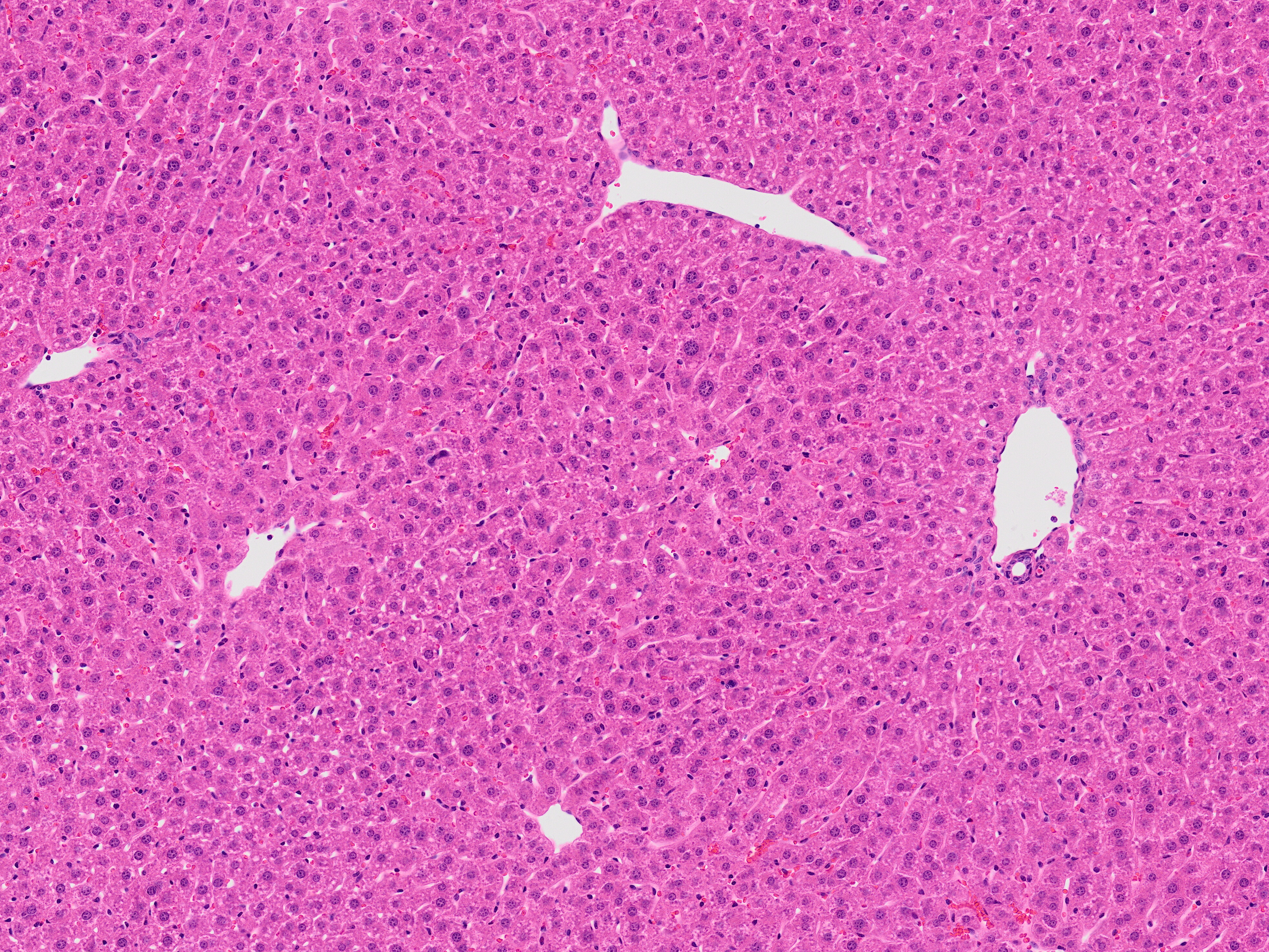

Supplement: Supplementary file 8 — Source data Fig. 6 [file 44318_2025_369_MOESM8_ESM.zip › Figure 6/6G/HE/Vehicle+IRI+Lip-1.png]

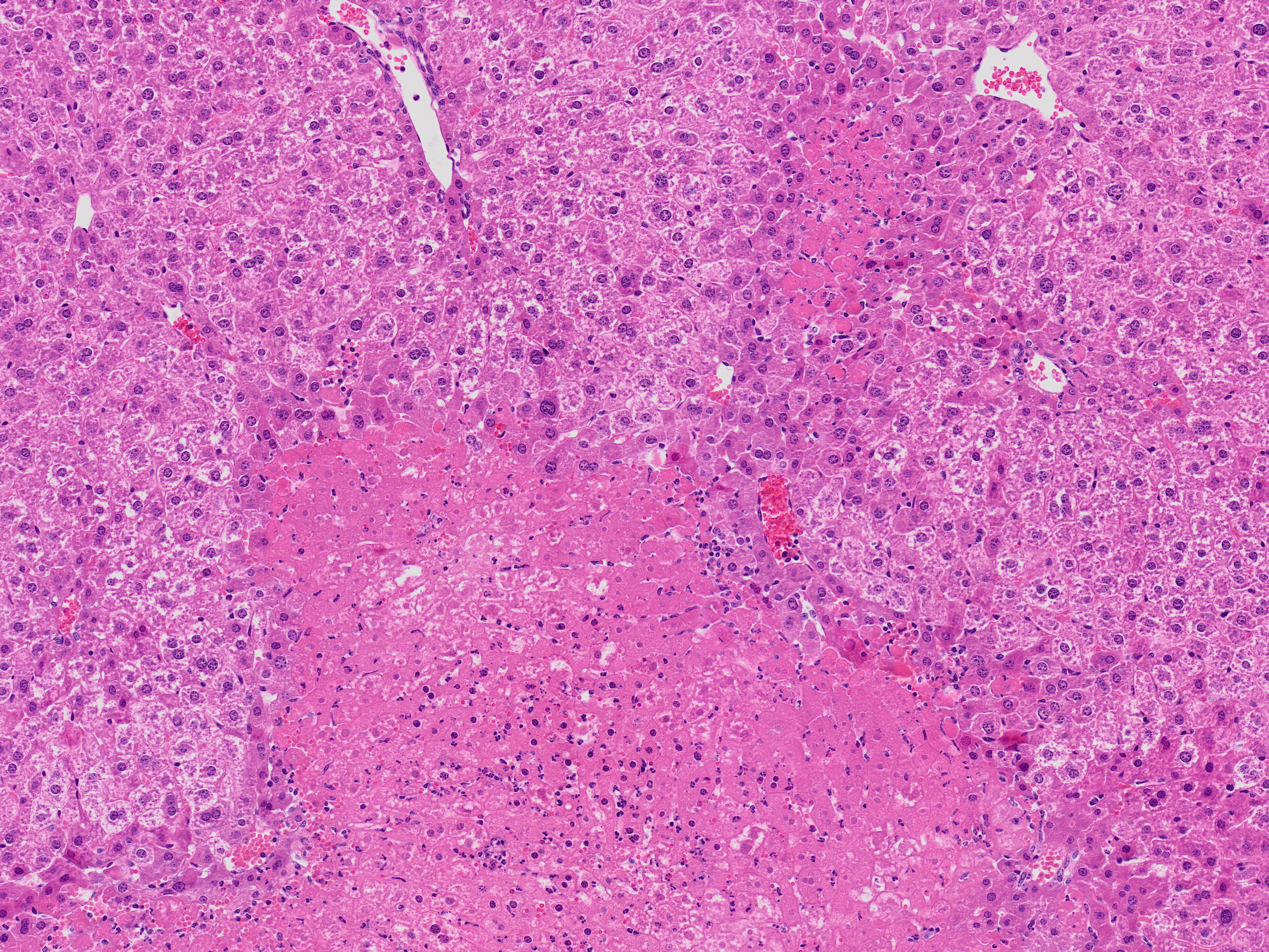

Supplement: Supplementary file 8 — Source data Fig. 6 [file 44318_2025_369_MOESM8_ESM.zip › Figure 6/6G/HE/Vehicle+IRI.png]

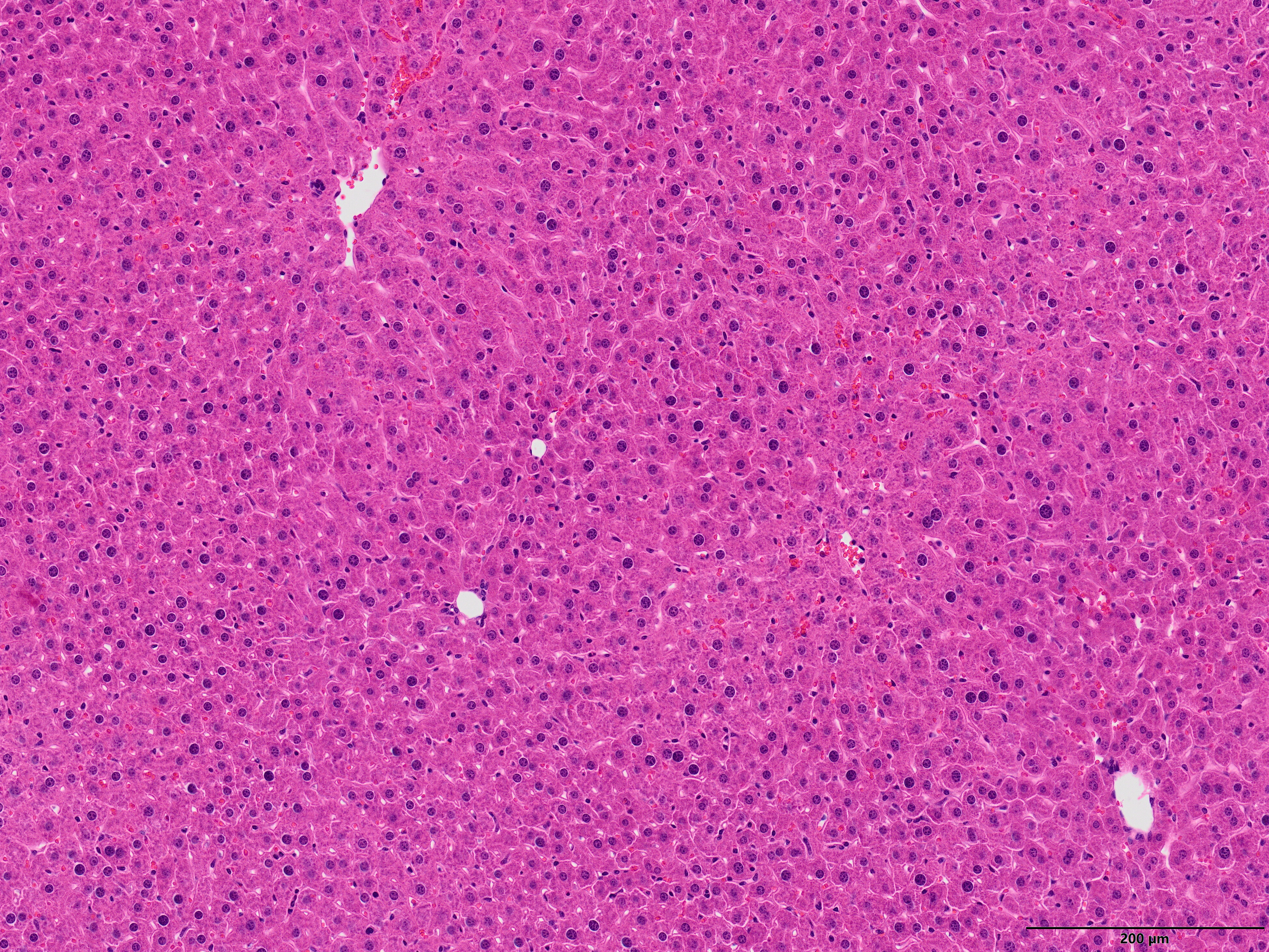

Supplement: Supplementary file 8 — Source data Fig. 6 [file 44318_2025_369_MOESM8_ESM.zip › Figure 6/6G/HE/Vehicle+Sham.png]

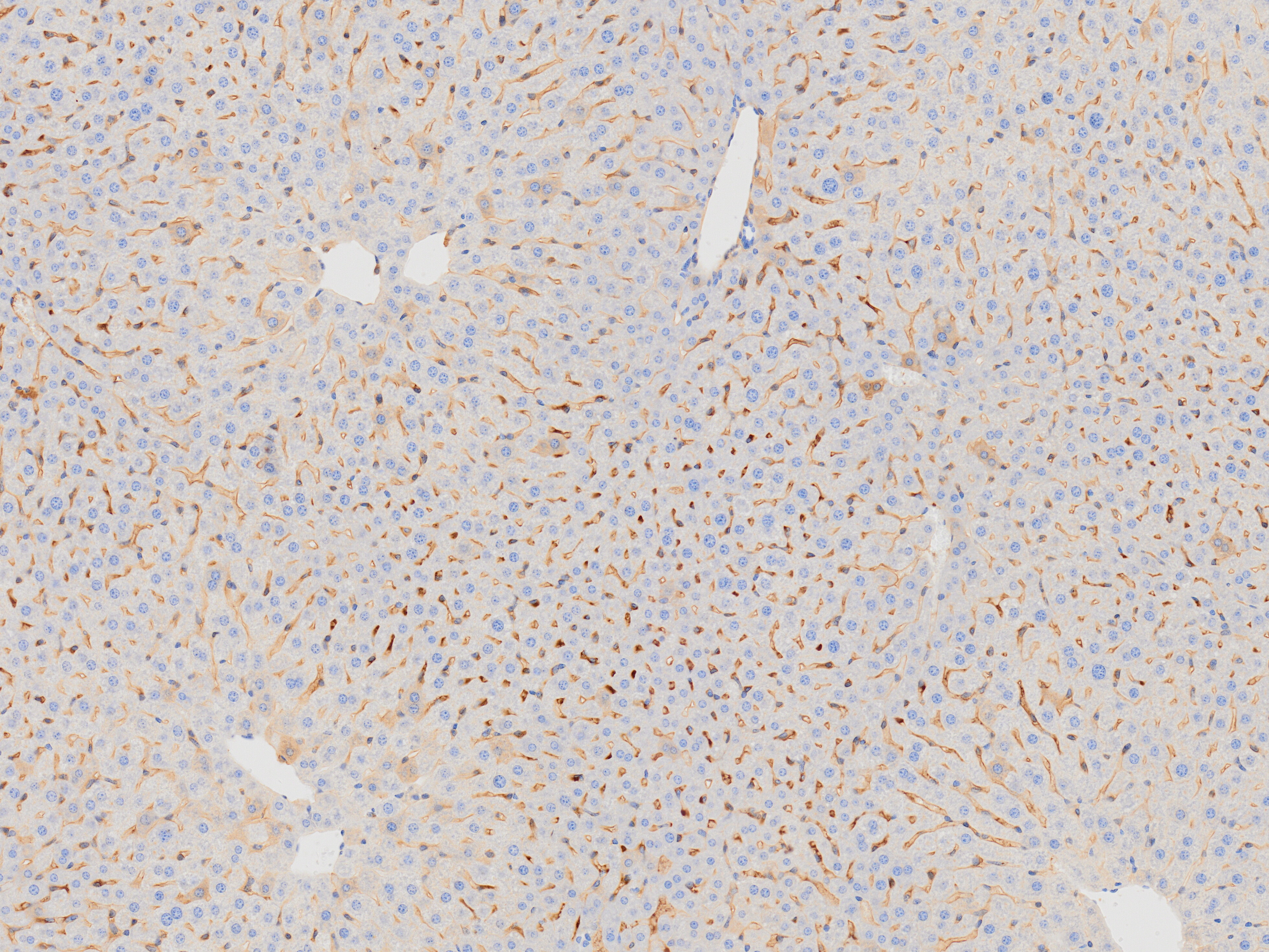

Supplement: Supplementary file 8 — Source data Fig. 6 [file 44318_2025_369_MOESM8_ESM.zip › Figure 6/6G/MDA/BTA+IRI+Lip-1.png]

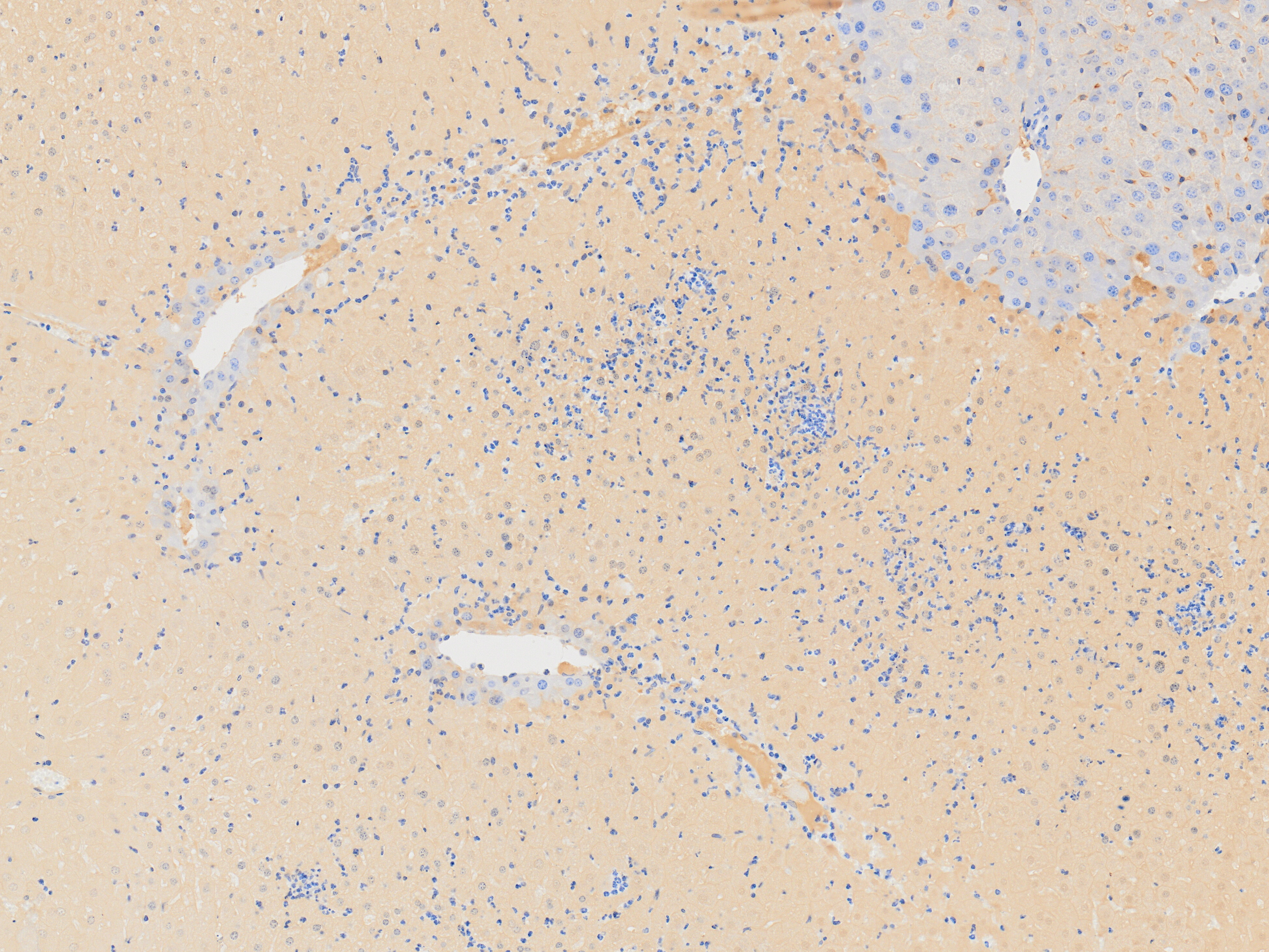

Supplement: Supplementary file 8 — Source data Fig. 6 [file 44318_2025_369_MOESM8_ESM.zip › Figure 6/6G/MDA/BTA+IRI.png]

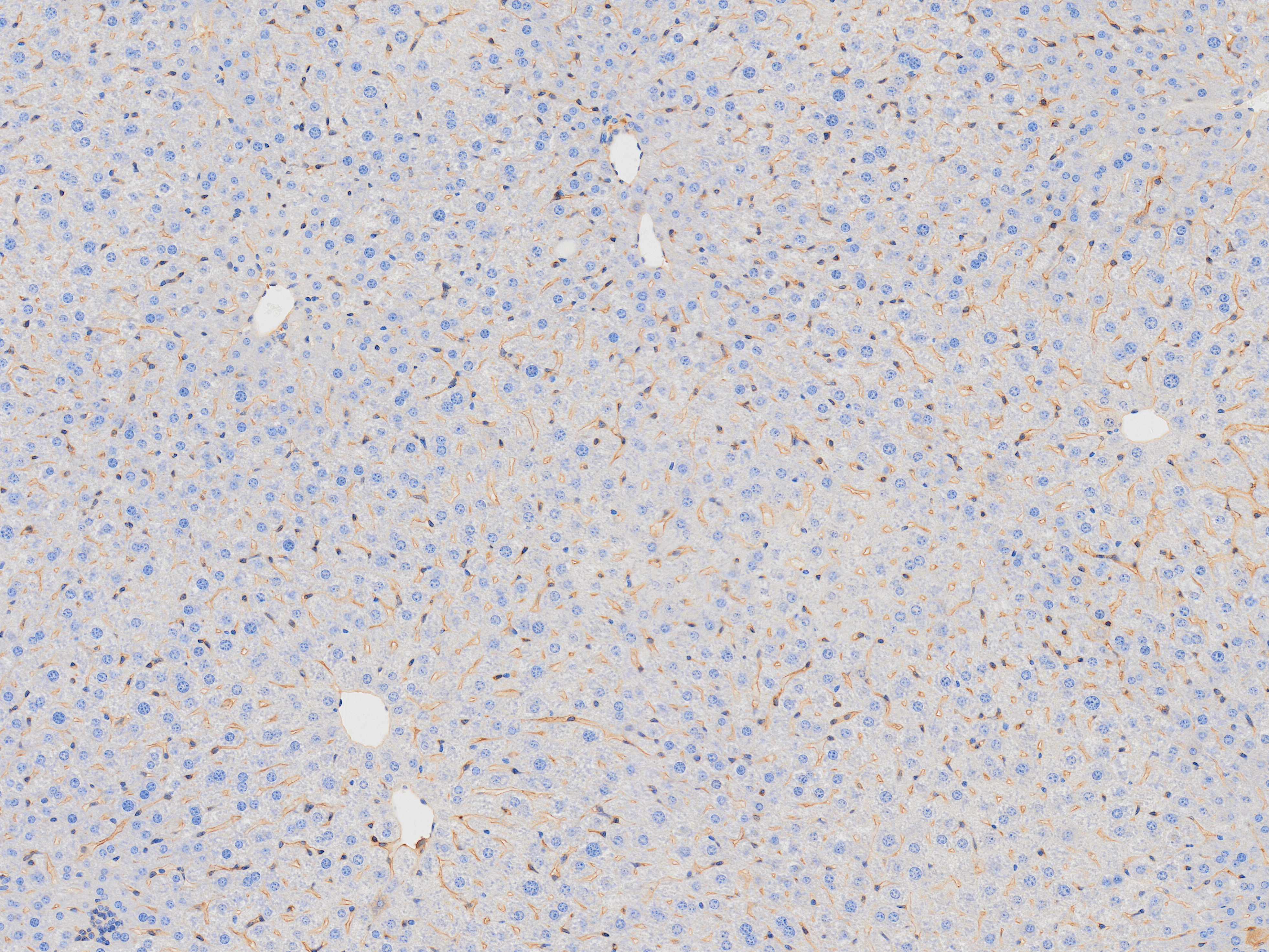

Supplement: Supplementary file 8 — Source data Fig. 6 [file 44318_2025_369_MOESM8_ESM.zip › Figure 6/6G/MDA/BTA+Sham.png]

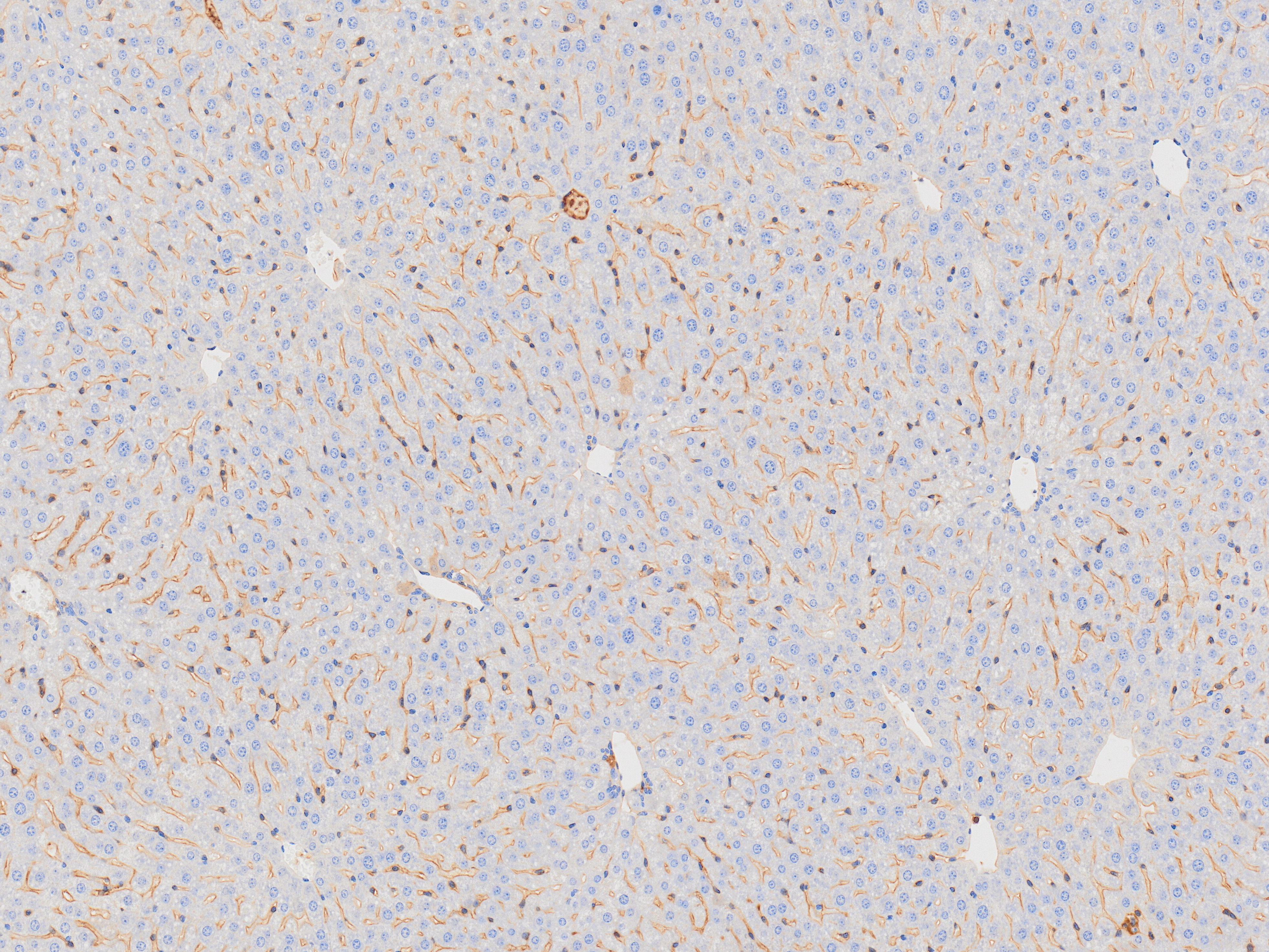

Supplement: Supplementary file 8 — Source data Fig. 6 [file 44318_2025_369_MOESM8_ESM.zip › Figure 6/6G/MDA/Vehicle+IRI+Lip-1.png]

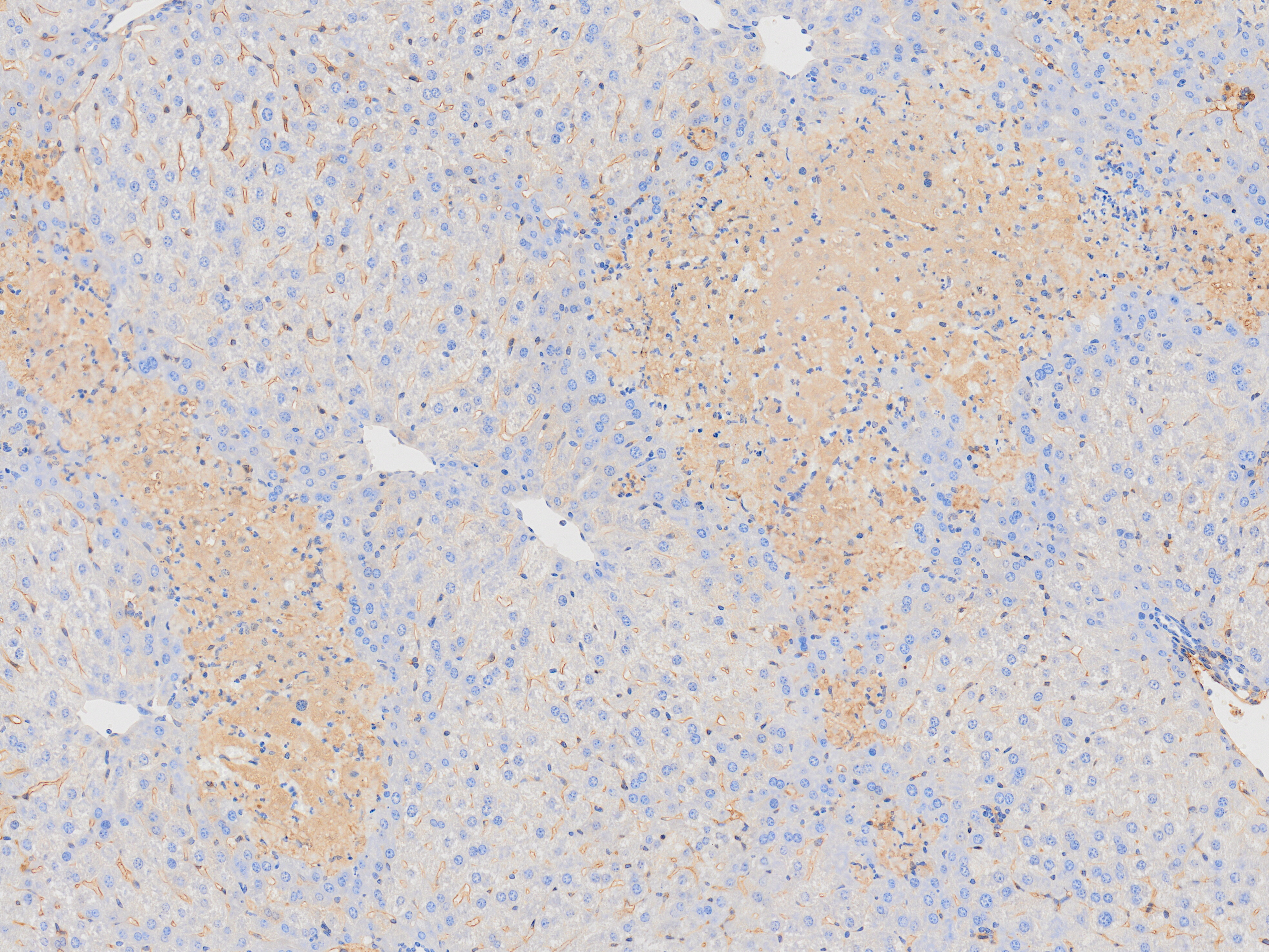

Supplement: Supplementary file 8 — Source data Fig. 6 [file 44318_2025_369_MOESM8_ESM.zip › Figure 6/6G/MDA/Vehicle+IRI.png]

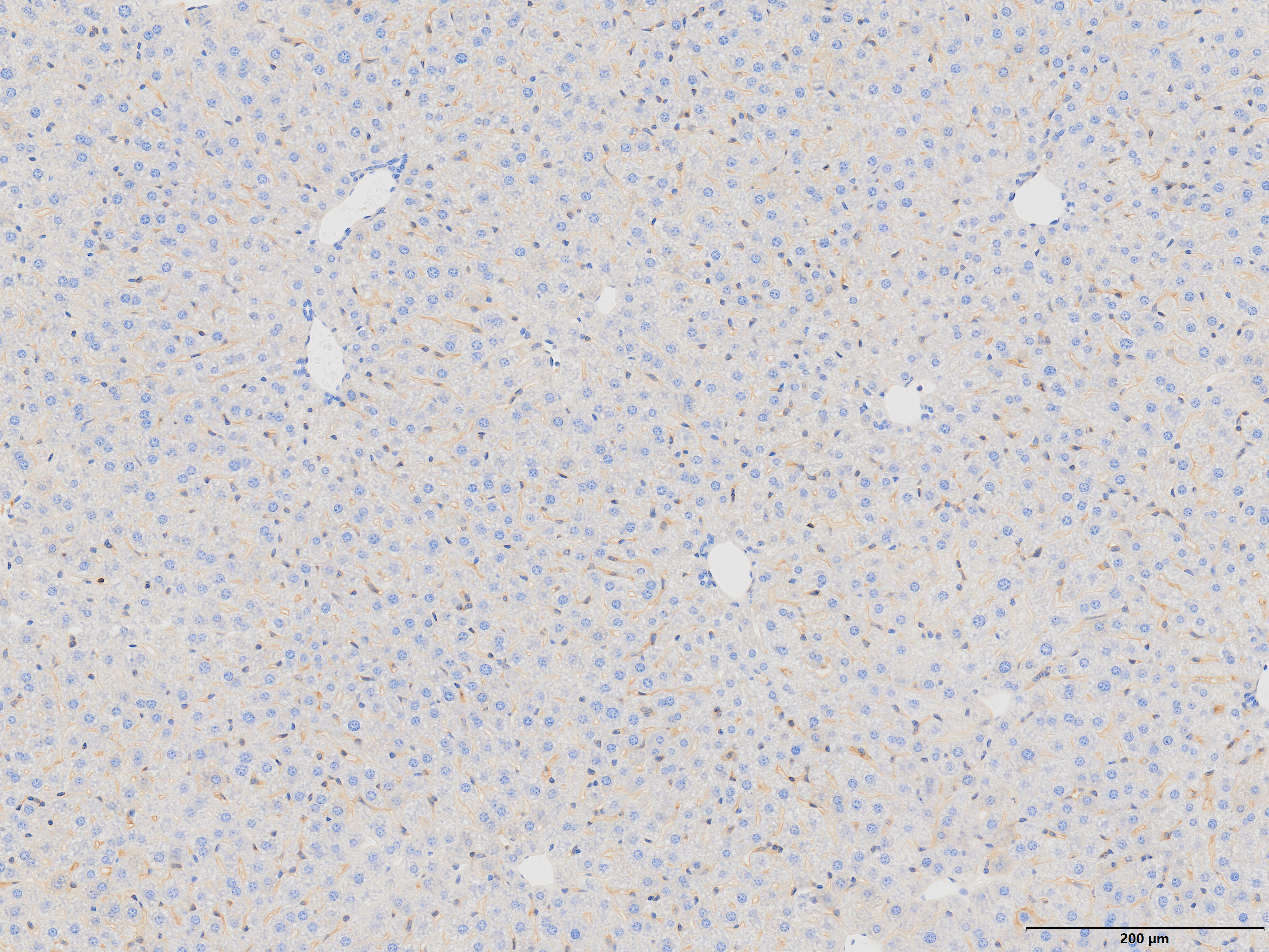

Supplement: Supplementary file 8 — Source data Fig. 6 [file 44318_2025_369_MOESM8_ESM.zip › Figure 6/6G/MDA/Vehicle+Sham.png]

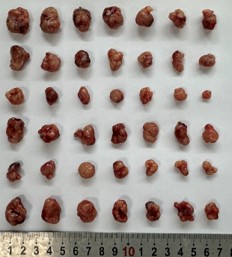

Supplement: Supplementary file 8 — Source data Fig. 6 [file 44318_2025_369_MOESM8_ESM.zip › Figure 6/6L/A375 Tumor photo.jpg]

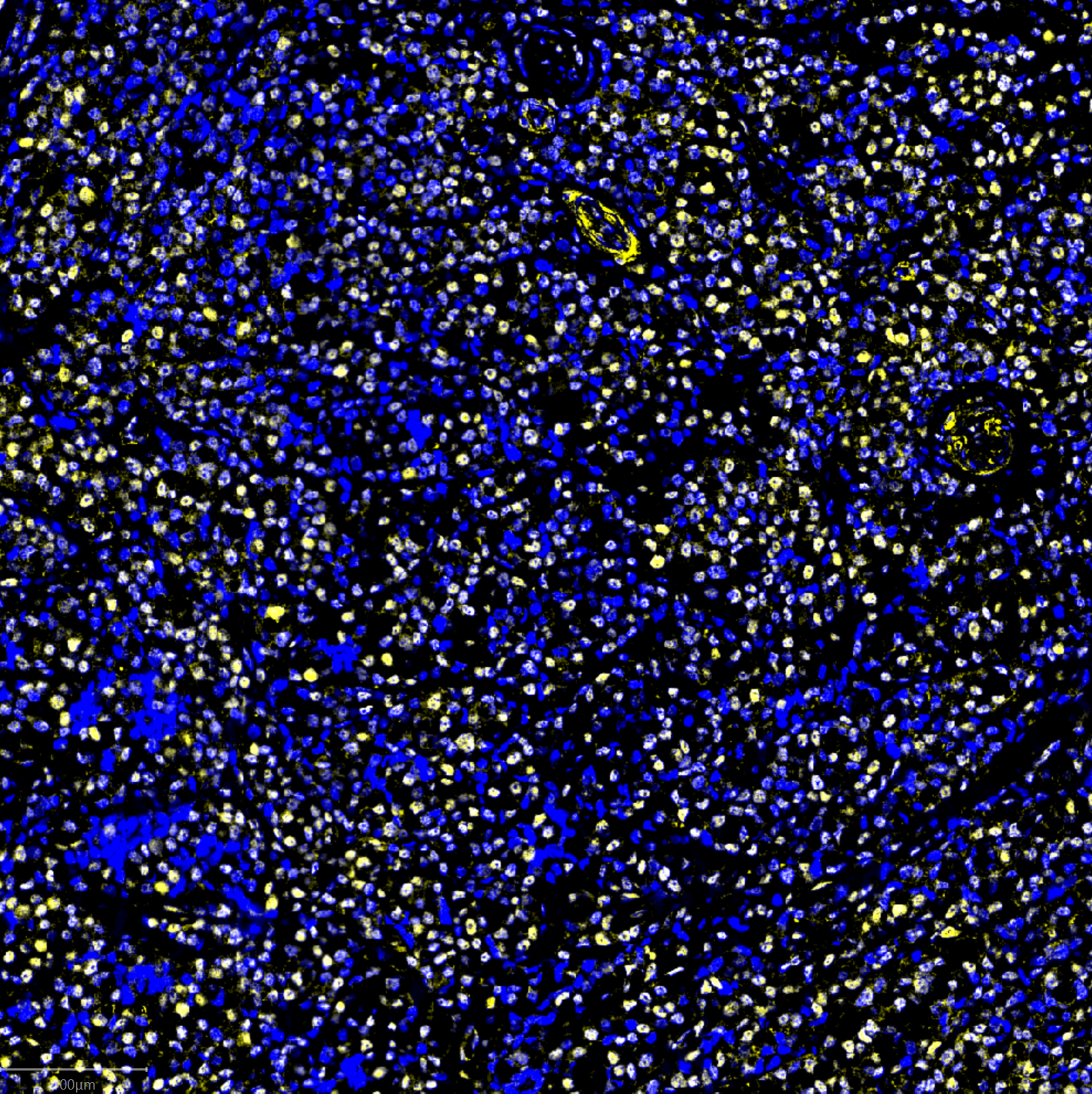

Supplement: Supplementary file 9 — Source data Fig. 7 [file 44318_2025_369_MOESM9_ESM.zip › Figure 7/7B/ACLY-high.png]

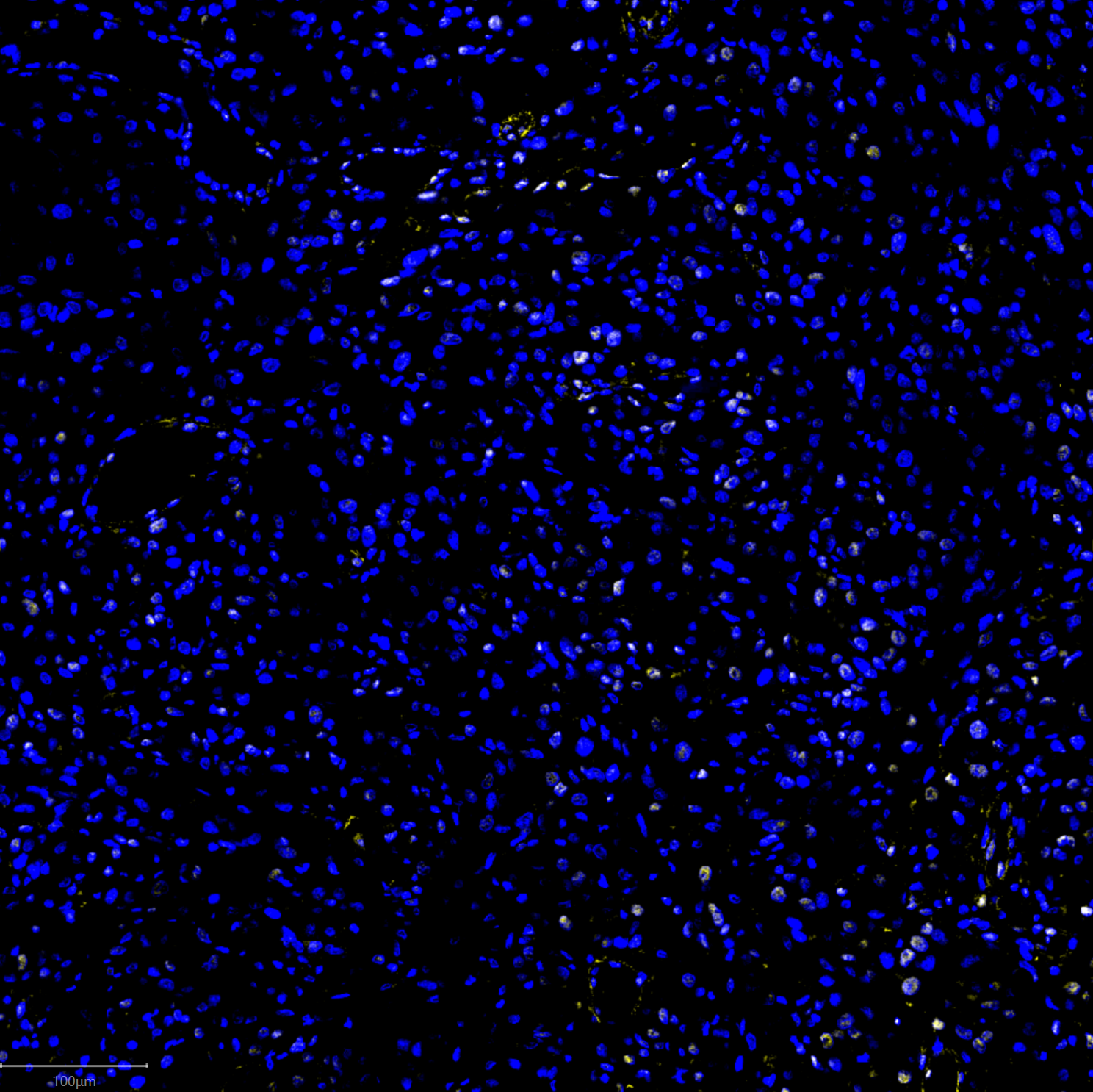

Supplement: Supplementary file 9 — Source data Fig. 7 [file 44318_2025_369_MOESM9_ESM.zip › Figure 7/7B/ACLY-low.png]

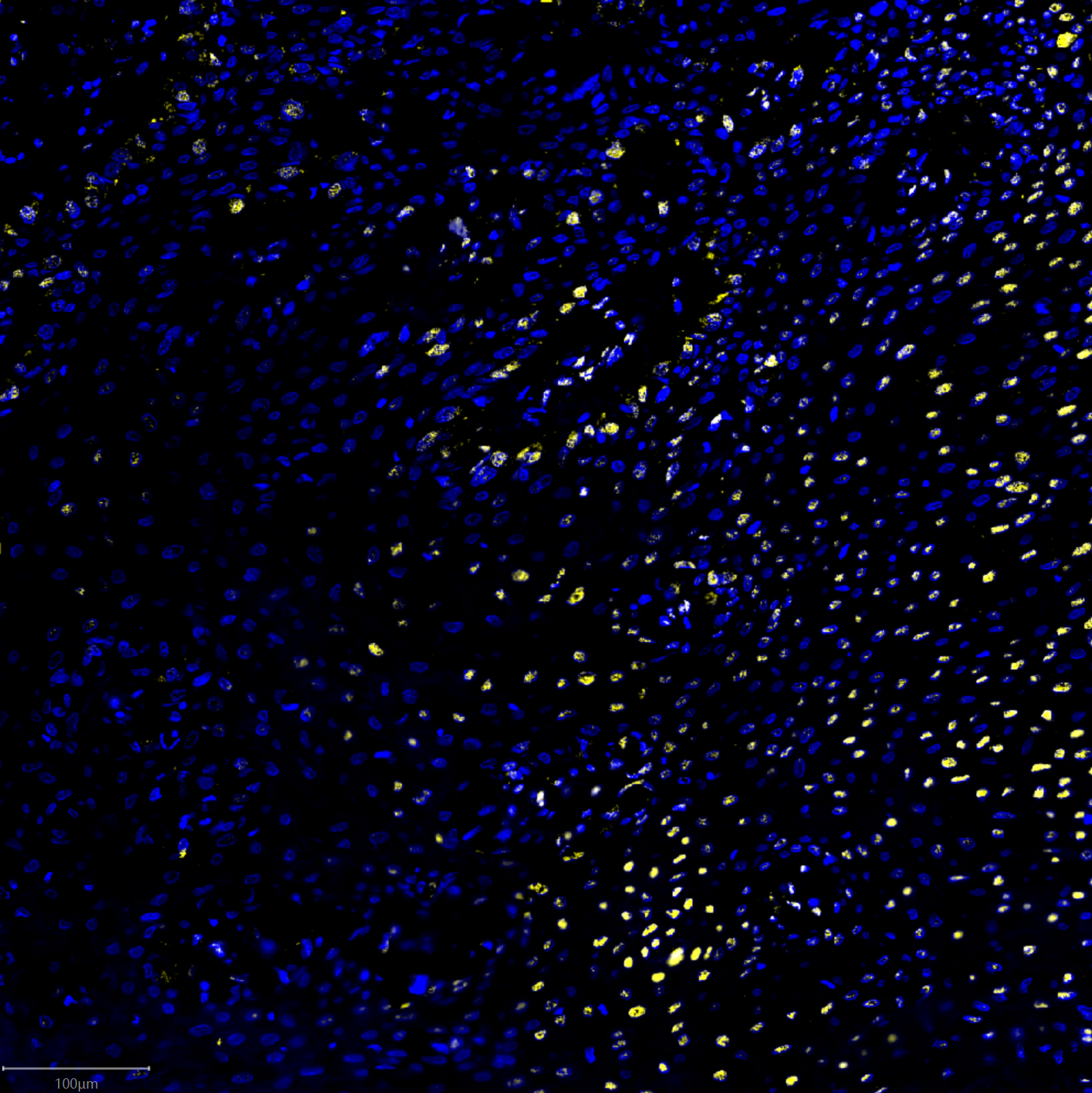

Supplement: Supplementary file 9 — Source data Fig. 7 [file 44318_2025_369_MOESM9_ESM.zip › Figure 7/7B/ACLY-medium.png]

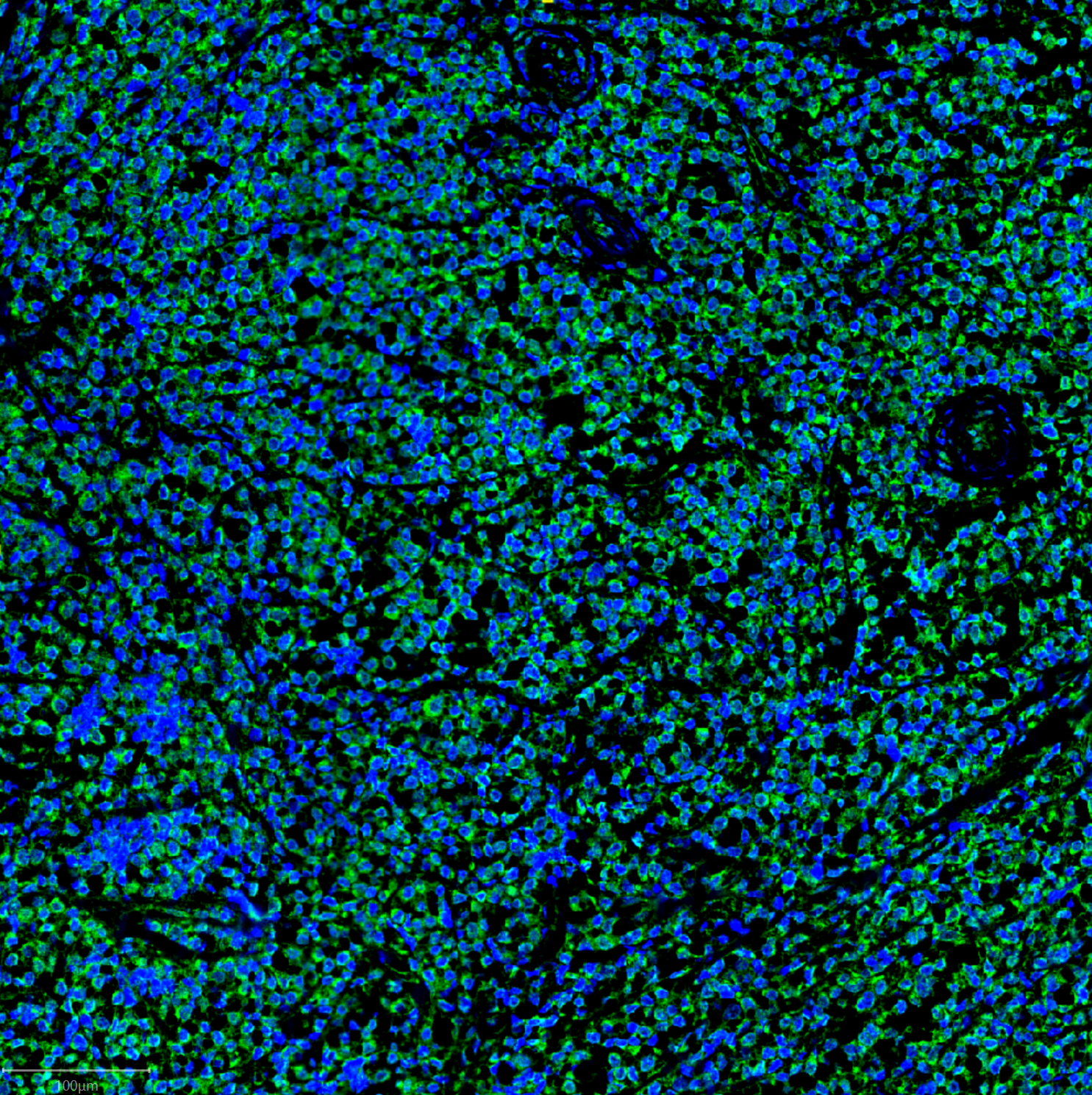

Supplement: Supplementary file 9 — Source data Fig. 7 [file 44318_2025_369_MOESM9_ESM.zip › Figure 7/7B/FSP1-high.png]

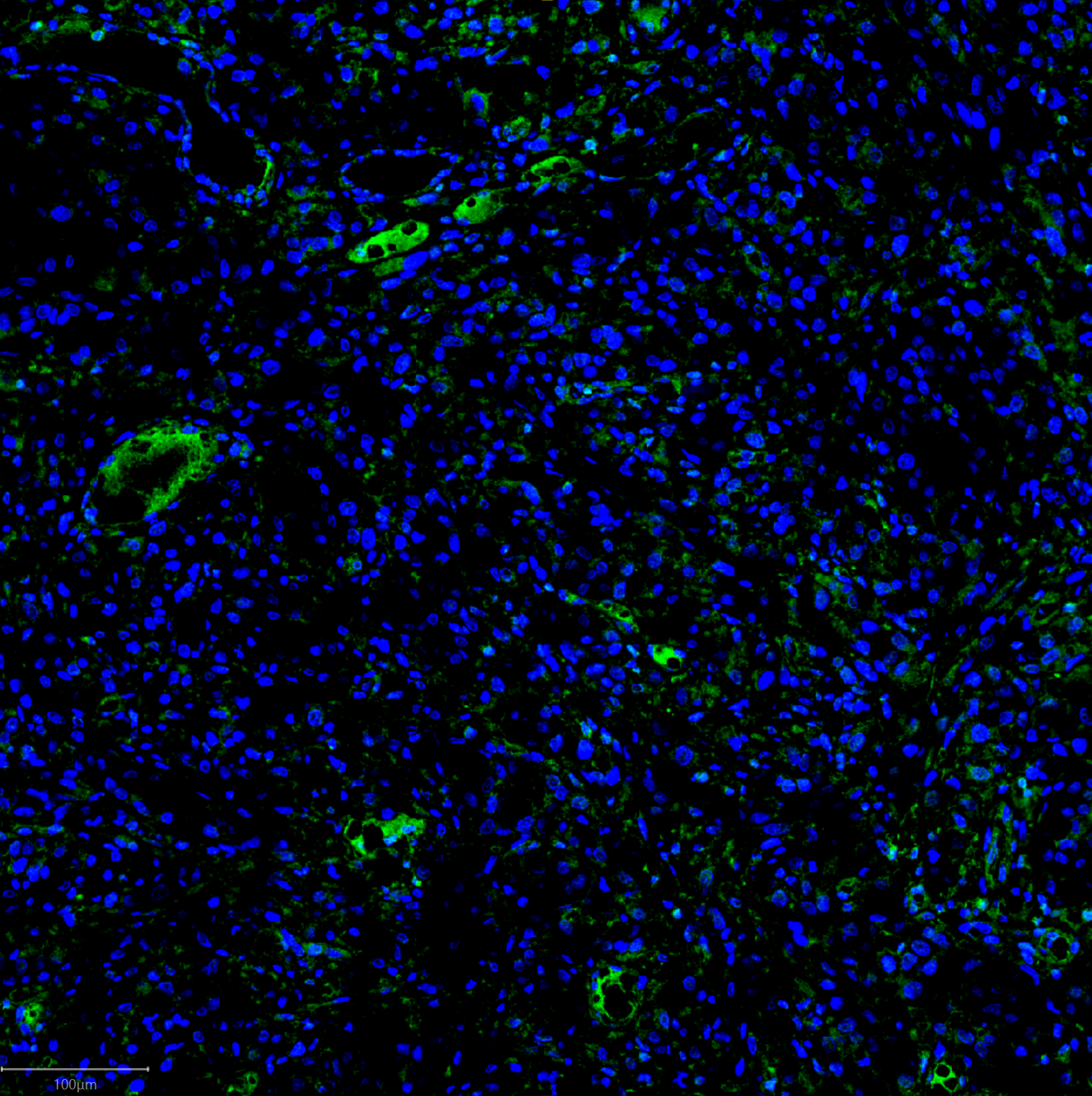

Supplement: Supplementary file 9 — Source data Fig. 7 [file 44318_2025_369_MOESM9_ESM.zip › Figure 7/7B/FSP1-low.png]

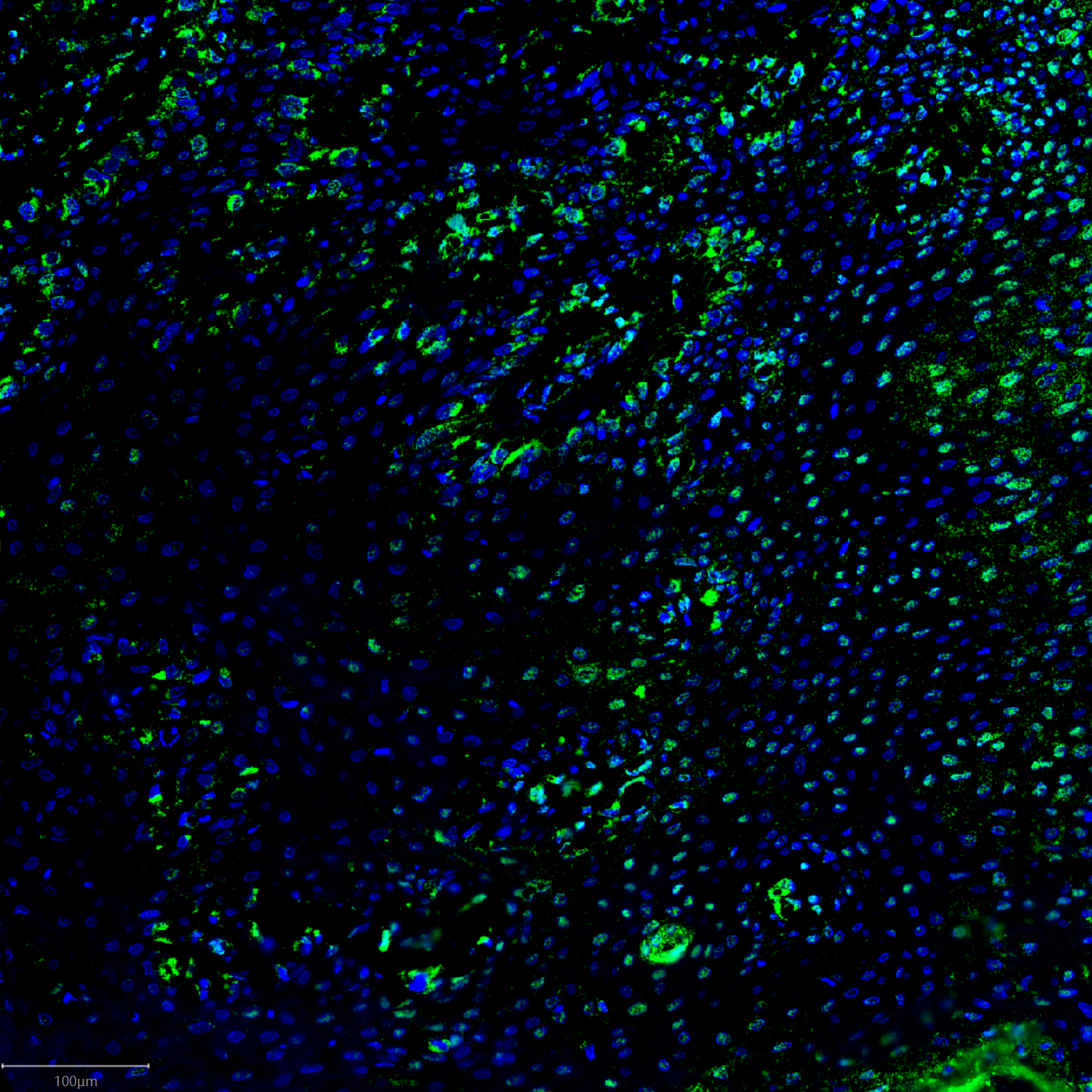

Supplement: Supplementary file 9 — Source data Fig. 7 [file 44318_2025_369_MOESM9_ESM.zip › Figure 7/7B/FSP1-medium.png]

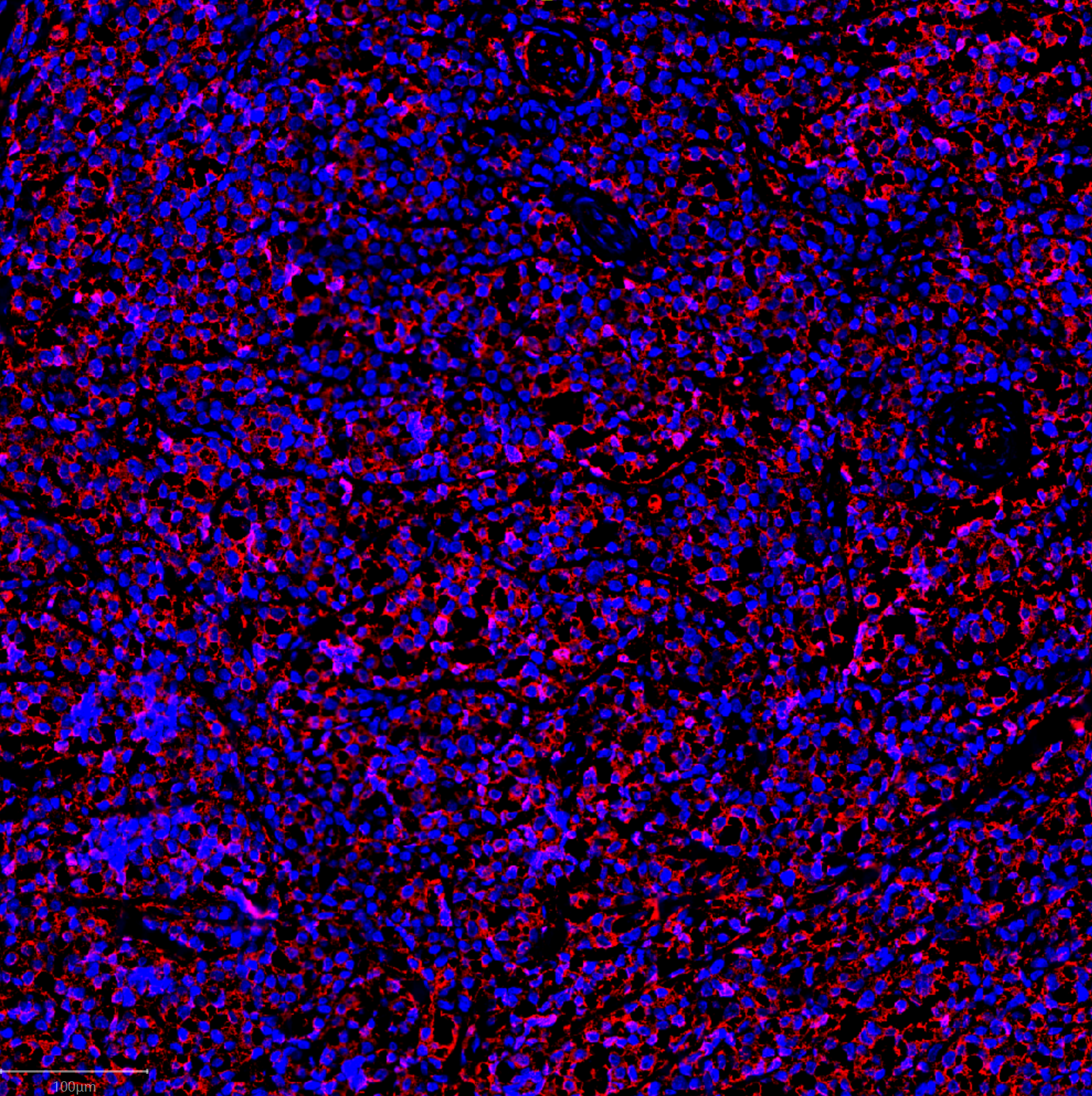

Supplement: Supplementary file 9 — Source data Fig. 7 [file 44318_2025_369_MOESM9_ESM.zip › Figure 7/7B/SLC25A1-high.png]

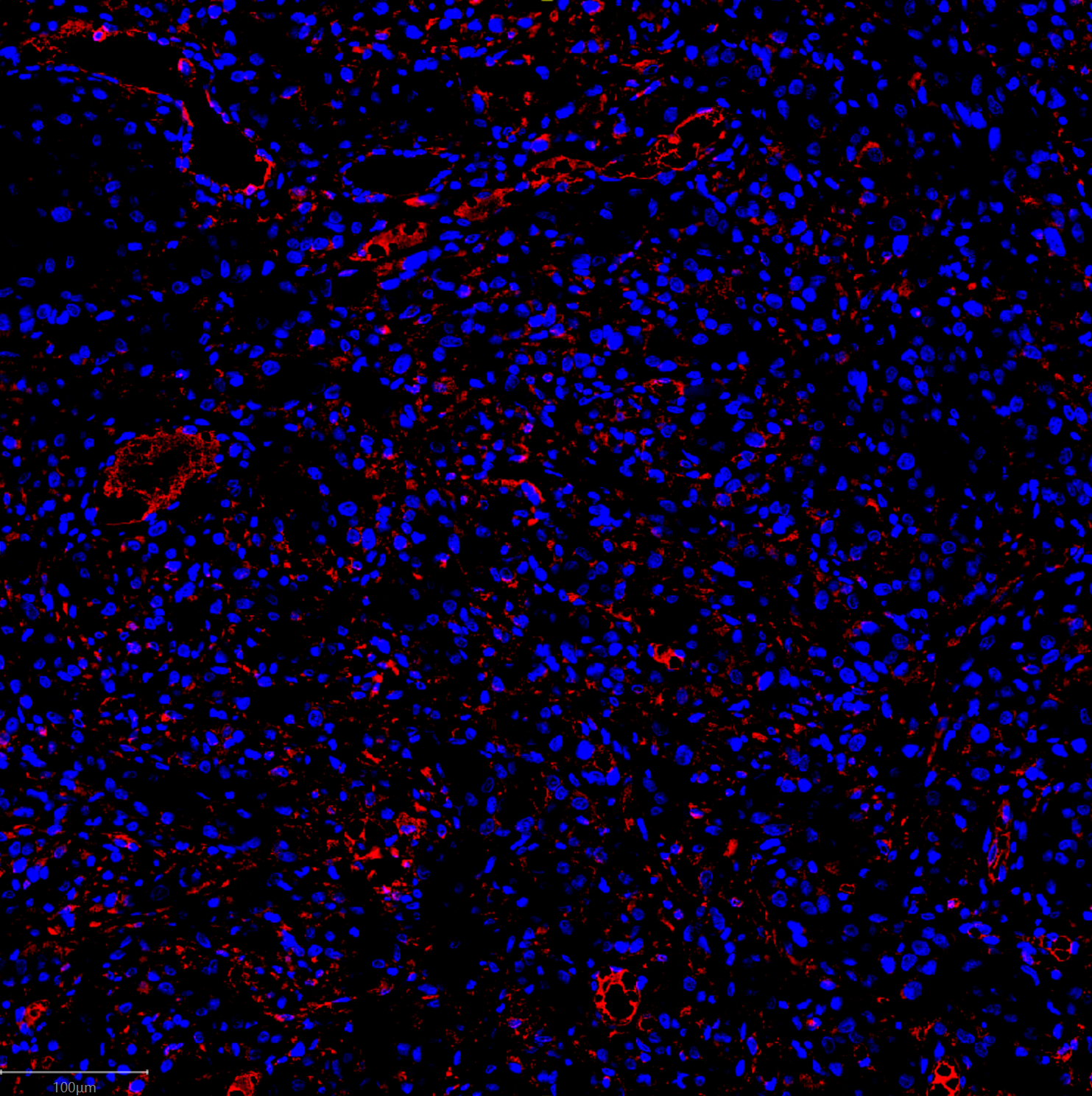

Supplement: Supplementary file 9 — Source data Fig. 7 [file 44318_2025_369_MOESM9_ESM.zip › Figure 7/7B/SLC25A1-low.png]

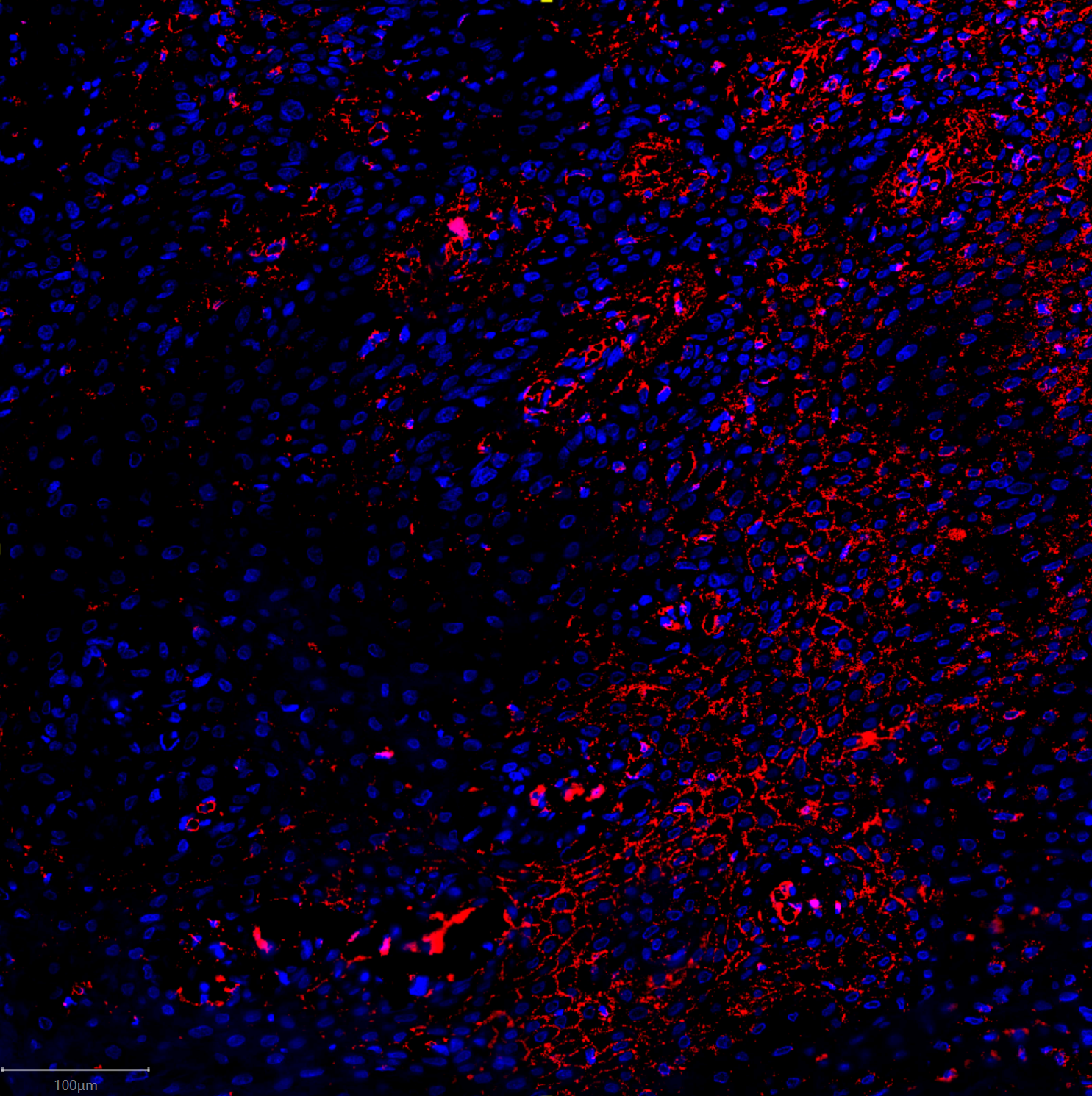

Supplement: Supplementary file 9 — Source data Fig. 7 [file 44318_2025_369_MOESM9_ESM.zip › Figure 7/7B/SLC25A1-medium.png]
