# Supplementary material for: SLC25A1 and ACLY maintain cytosolic acetyl-CoA and regulate ferroptosis susceptibility via FSP1 acetylation
Source: EMBO J. 2025 Jan 29;44(6):1641–62. doi: 10.1038/s44318-025-00369-5 (PMC11914110; doi:10.1038/s44318-025-00369-5)
Supplement: Supplementary file 4 — Source data Fig. 2 [file 44318_2025_369_MOESM4_ESM.zip › Figure 2/2C/2C-A375-A549-WB.pptx]

## Slide 1
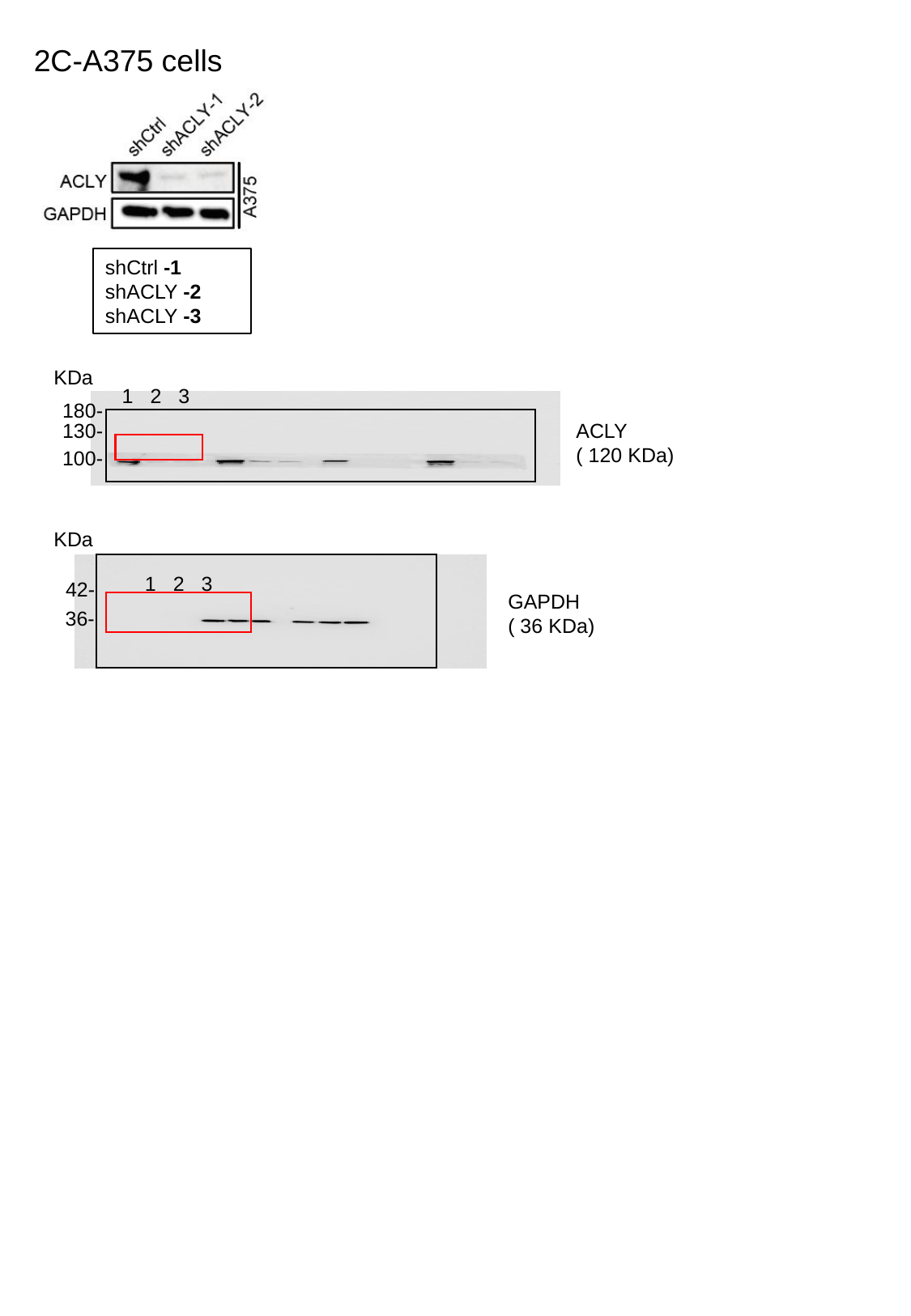

2C-A375 cells
shCtrl -1
shACLY -2
shACLY -3
KDa
1 2 3
180-
130-
ACLY
( 120 KDa)
100-
KDa
1 2 3
42-
GAPDH
( 36 KDa)
36-

## Slide 2
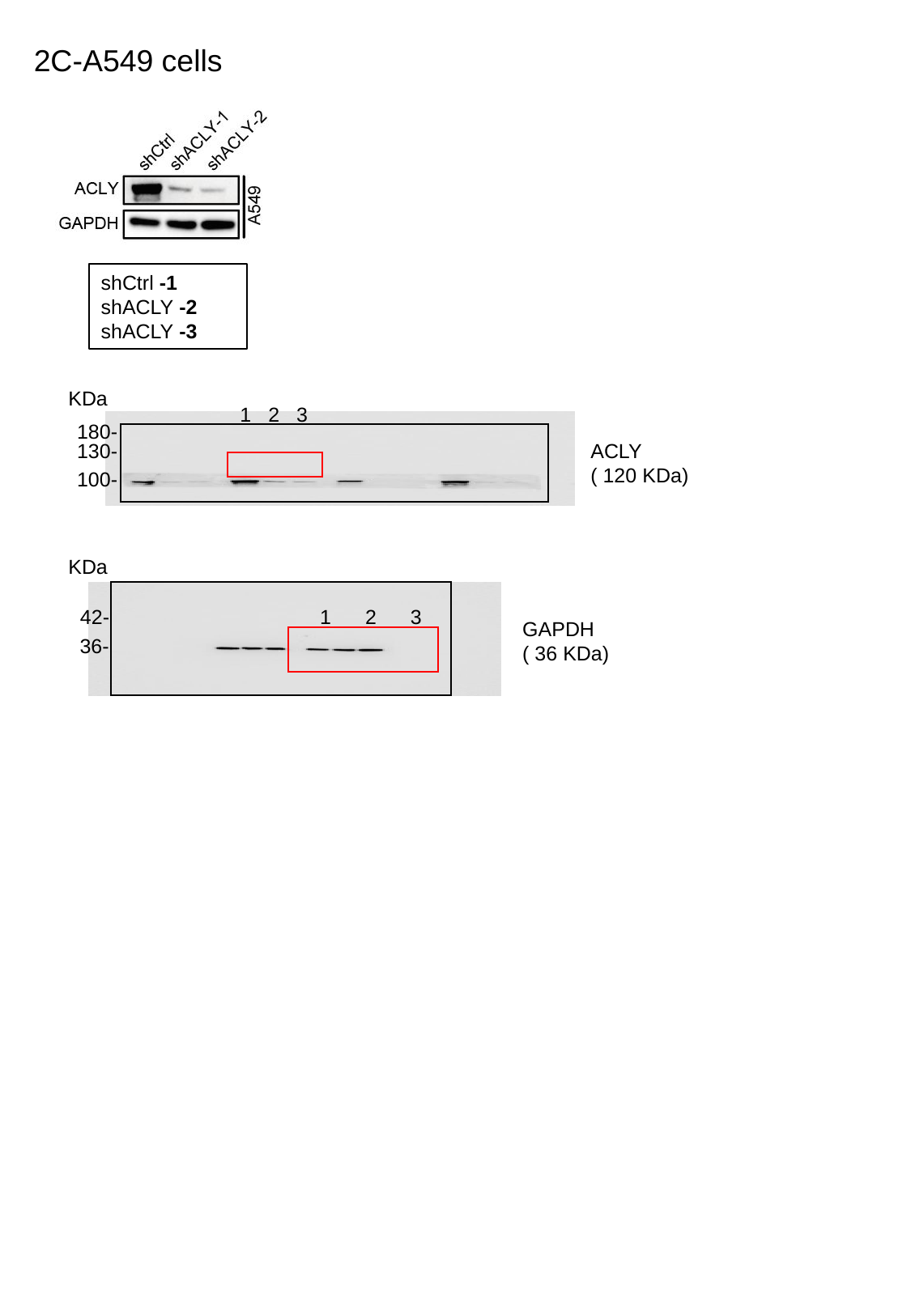

2C-A549 cells
shCtrl -1
shACLY -2
shACLY -3
KDa
1 2 3
180-
130-
ACLY
( 120 KDa)
100-
KDa
1 2 3
42-
GAPDH
( 36 KDa)
36-
