# Supplementary material for: SLC25A1 and ACLY maintain cytosolic acetyl-CoA and regulate ferroptosis susceptibility via FSP1 acetylation
Source: EMBO J. 2025 Jan 29;44(6):1641–62. doi: 10.1038/s44318-025-00369-5 (PMC11914110; doi:10.1038/s44318-025-00369-5)
Supplement: Supplementary file 4 — Source data Fig. 2 [file 44318_2025_369_MOESM4_ESM.zip › Figure 2/2G/2G-A375-A549-WB.pptx]

## Slide 1
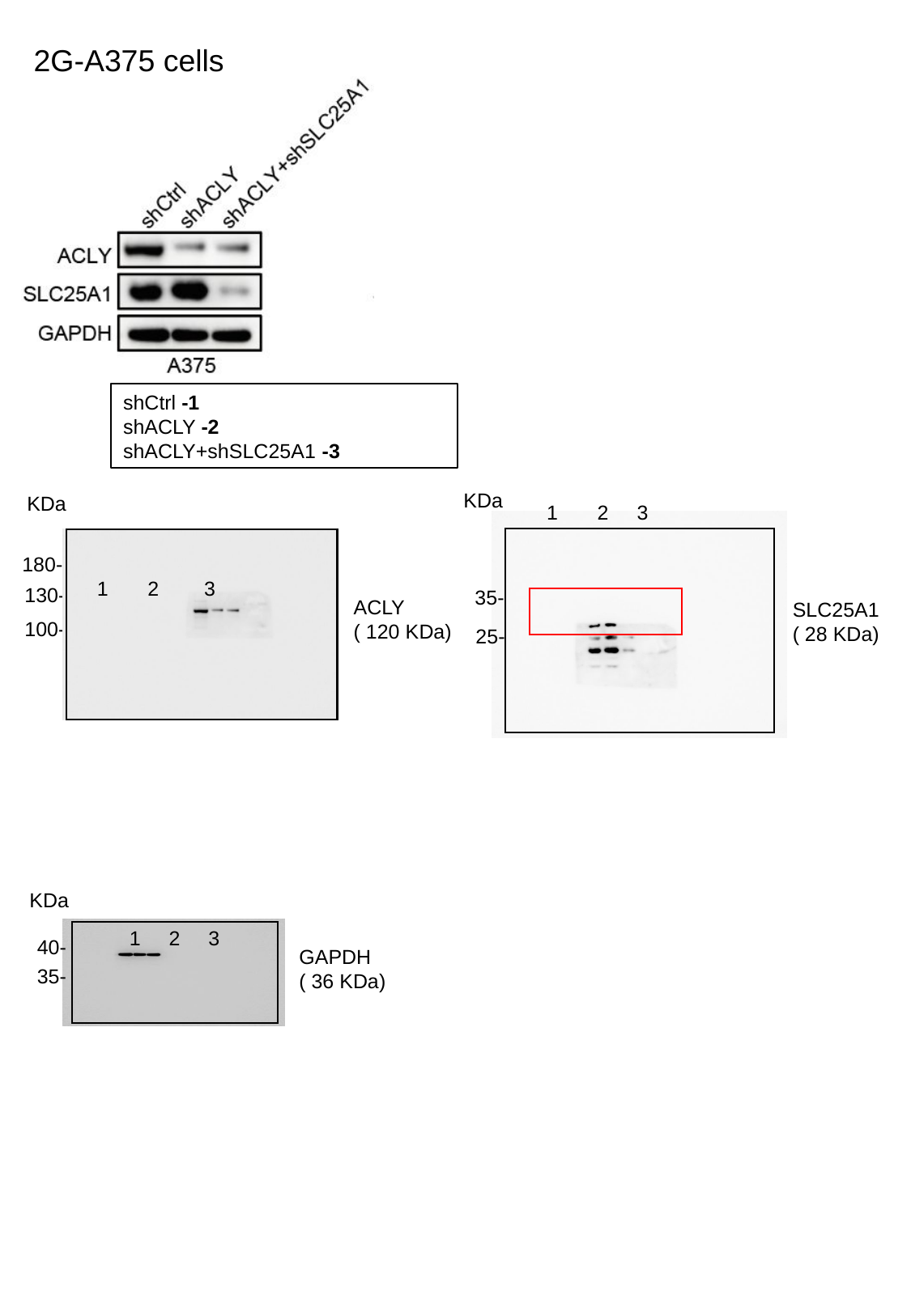

2G-A375 cells
shCtrl -1
shACLY -2
shACLY+shSLC25A1 -3
KDa
KDa
1 2 3
180-
1 2 3
130-
35-
ACLY
( 120 KDa)
SLC25A1
( 28 KDa)
100-
25-
KDa
1 2 3
40-
GAPDH
( 36 KDa)
35-

## Slide 2
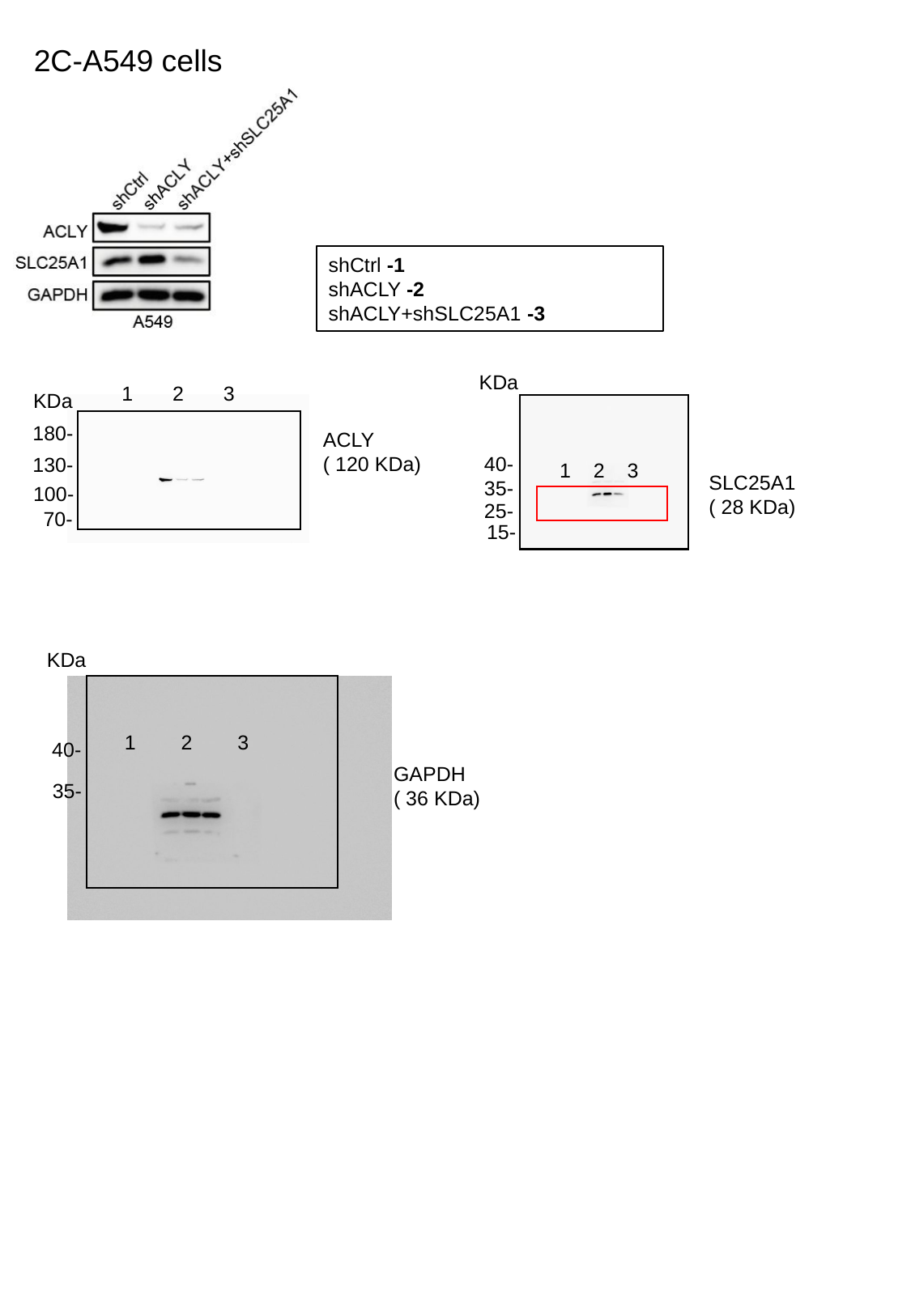

2C-A549 cells
shCtrl -1
shACLY -2
shACLY+shSLC25A1 -3
KDa
 1 2 3
KDa
180-
ACLY
( 120 KDa)
40-
130-
 1 2 3
SLC25A1
( 28 KDa)
35-
100-
25-
70-
15-
KDa
 1 2 3
40-
GAPDH
( 36 KDa)
35-
