# Supplementary material for: SLC25A1 and ACLY maintain cytosolic acetyl-CoA and regulate ferroptosis susceptibility via FSP1 acetylation
Source: EMBO J. 2025 Jan 29;44(6):1641–62. doi: 10.1038/s44318-025-00369-5 (PMC11914110; doi:10.1038/s44318-025-00369-5)
Supplement: Supplementary file 5 — Source data Fig. 3 [file 44318_2025_369_MOESM5_ESM.zip › Figure 3/3B/3B-A375-A549-WB.pptx]

## Slide 1
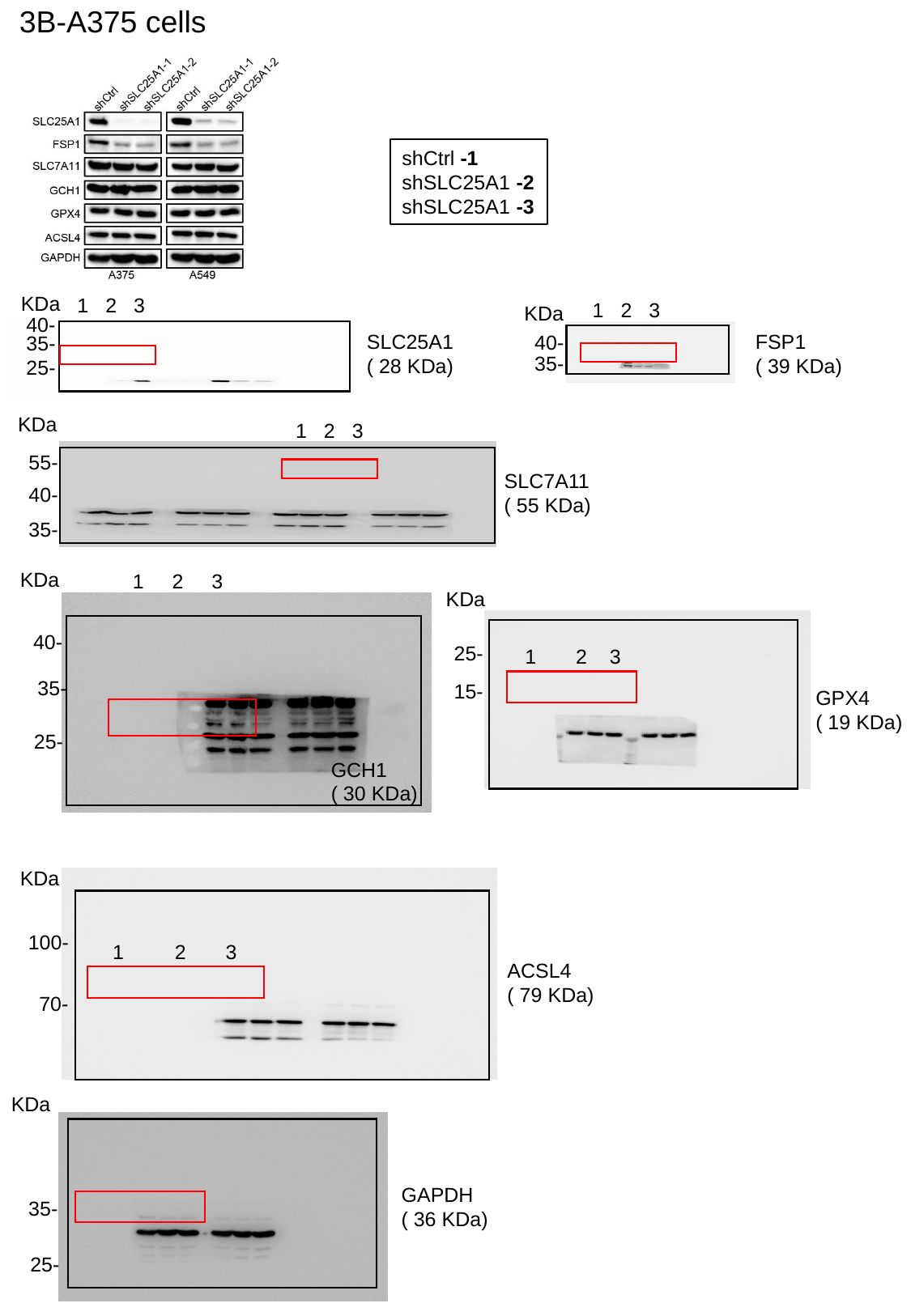

3B-A375 cells
shCtrl -1
shSLC25A1 -2
shSLC25A1 -3
KDa
 1 2 3
 1 2 3
KDa
40-
SLC25A1
( 28 KDa)
FSP1
( 39 KDa)
40-
35-
35-
25-
KDa
 1 2 3
55-
SLC7A11
( 55 KDa)
40-
35-
KDa
 1 2 3
KDa
40-
25-
 1 2 3
35-
15-
GPX4
( 19 KDa)
25-
GCH1
( 30 KDa)
KDa
100-
 1 2 3
ACSL4
( 79 KDa)
70-
KDa
GAPDH
( 36 KDa)
35-
25-

## Slide 2
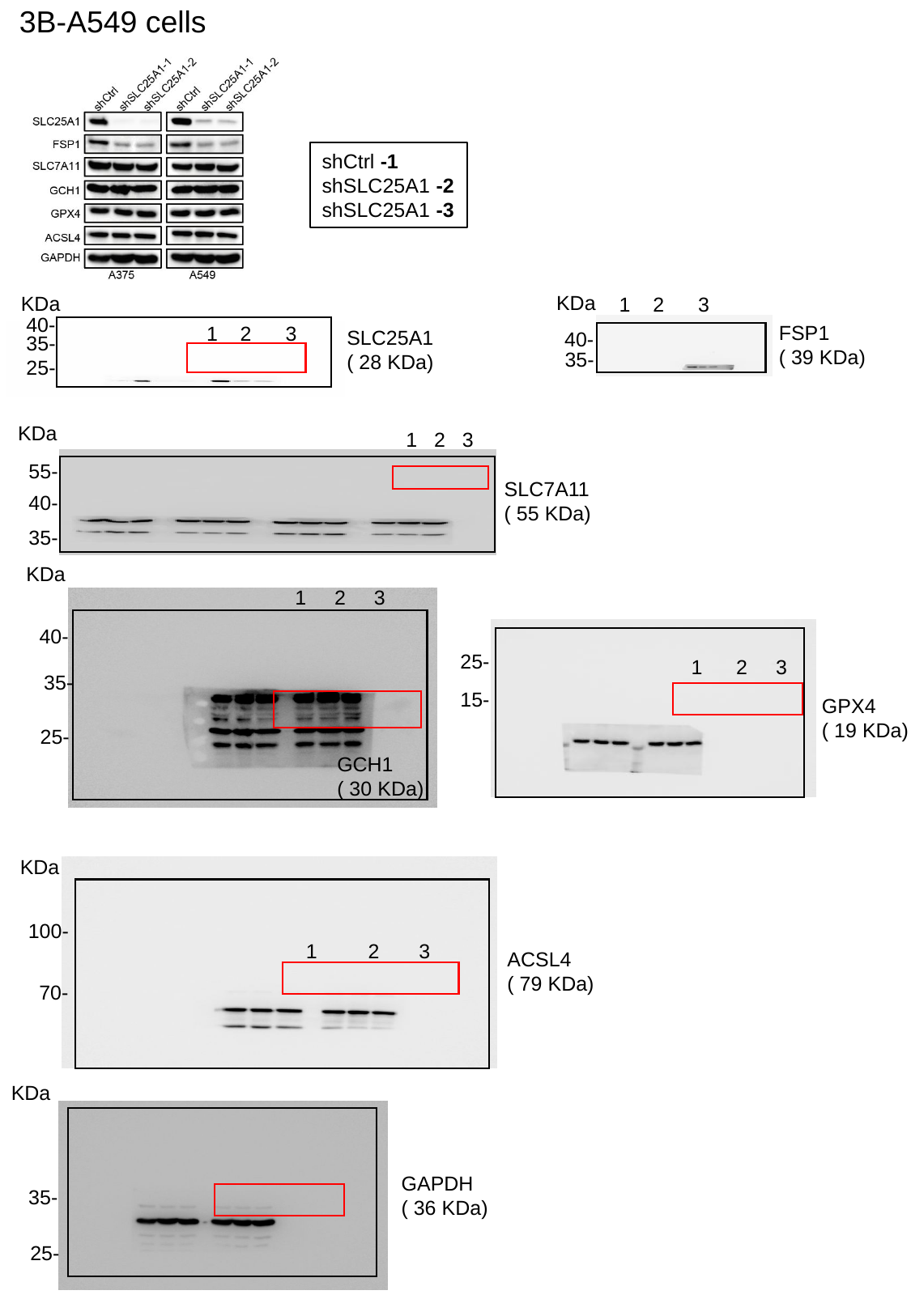

3B-A549 cells
shCtrl -1
shSLC25A1 -2
shSLC25A1 -3
KDa
KDa
 1 2 3
40-
100-
FSP1
( 39 KDa)
 1 2 3
SLC25A1
( 28 KDa)
40-
35-
35-
25-
KDa
 1 2 3
55-
SLC7A11
( 55 KDa)
40-
35-
KDa
 1 2 3
40-
25-
 1 2 3
35-
15-
GPX4
( 19 KDa)
25-
GCH1
( 30 KDa)
KDa
100-
 1 2 3
ACSL4
( 79 KDa)
70-
KDa
GAPDH
( 36 KDa)
35-
25-
