# Supplementary material for: SLC25A1 and ACLY maintain cytosolic acetyl-CoA and regulate ferroptosis susceptibility via FSP1 acetylation
Source: EMBO J. 2025 Jan 29;44(6):1641–62. doi: 10.1038/s44318-025-00369-5 (PMC11914110; doi:10.1038/s44318-025-00369-5)
Supplement: Supplementary file 5 — Source data Fig. 3 [file 44318_2025_369_MOESM5_ESM.zip › Figure 3/3D/3D-A375-A549-WB.pptx]

## Slide 1
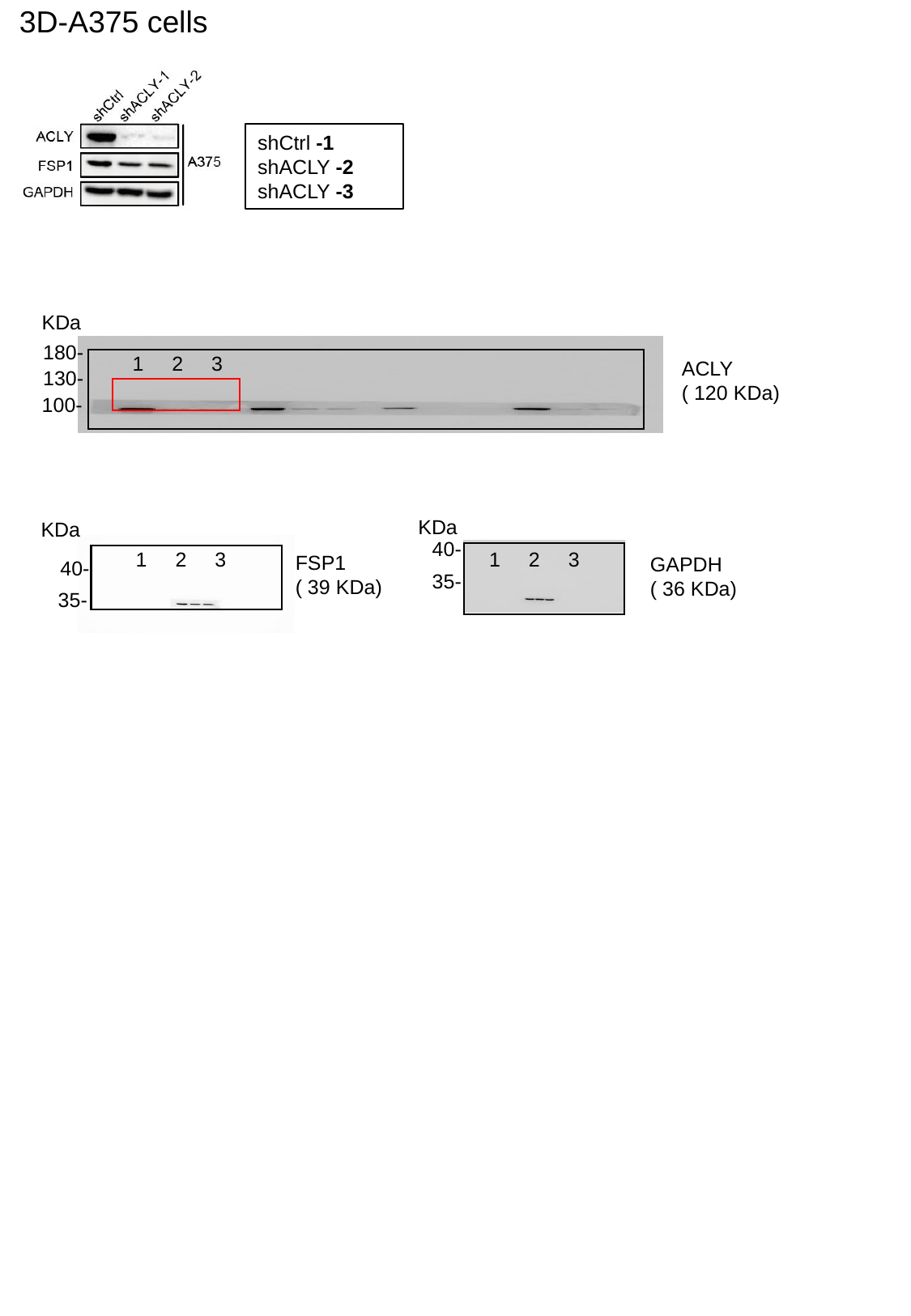

3D-A375 cells
shCtrl -1
shACLY -2
shACLY -3
KDa
180-
 1 2 3
ACLY
( 120 KDa)
130-
100-
KDa
KDa
40-
 1 2 3
 1 2 3
FSP1
( 39 KDa)
GAPDH
( 36 KDa)
40-
35-
35-

## Slide 2
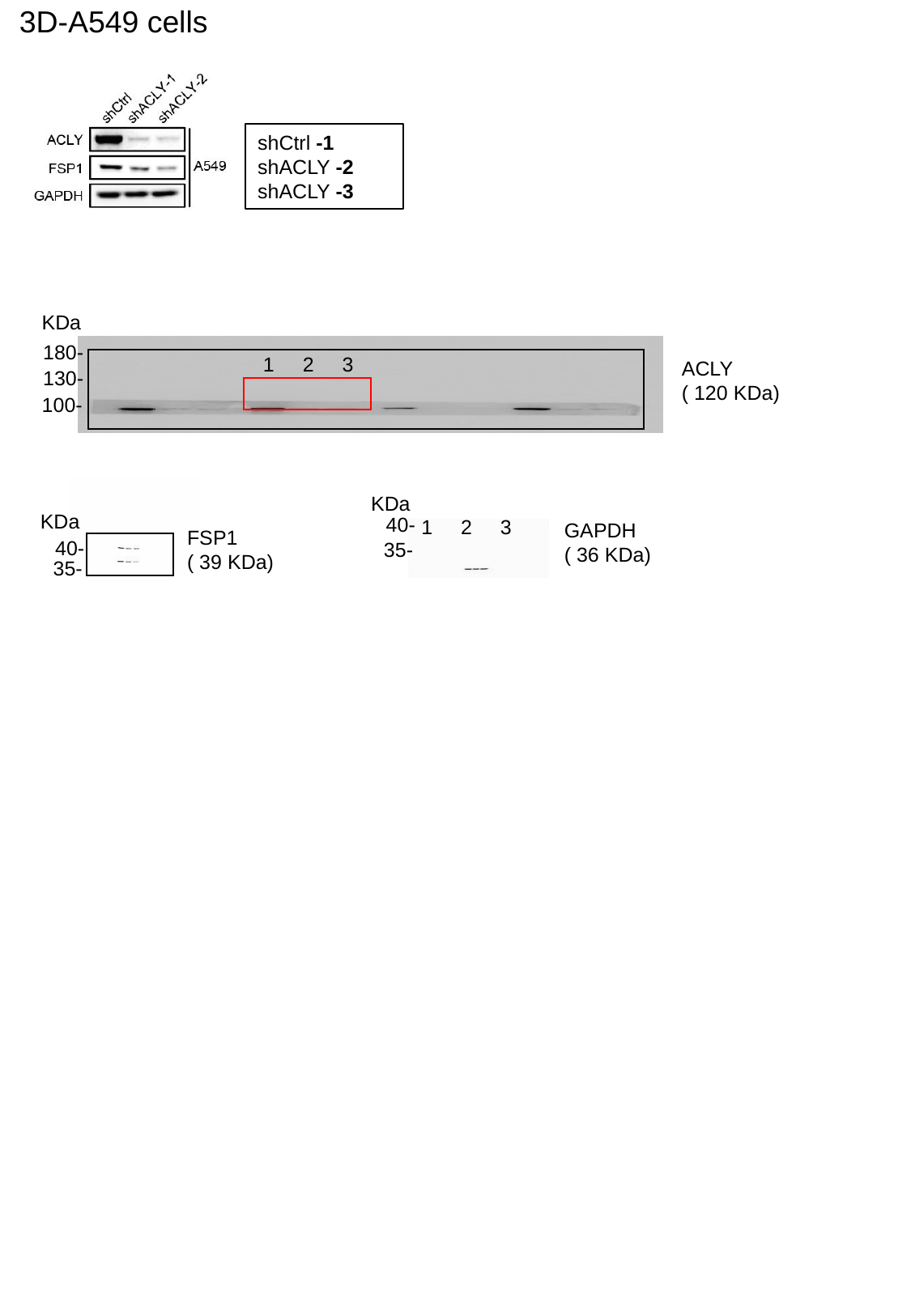

3D-A549 cells
shCtrl -1
shACLY -2
shACLY -3
KDa
180-
 1 2 3
ACLY
( 120 KDa)
130-
100-
KDa
KDa
40-
 1 2 3
GAPDH
( 36 KDa)
FSP1
( 39 KDa)
40-
35-
35-
