# Supplementary material for: SLC25A1 and ACLY maintain cytosolic acetyl-CoA and regulate ferroptosis susceptibility via FSP1 acetylation
Source: EMBO J. 2025 Jan 29;44(6):1641–62. doi: 10.1038/s44318-025-00369-5 (PMC11914110; doi:10.1038/s44318-025-00369-5)
Supplement: Supplementary file 5 — Source data Fig. 3 [file 44318_2025_369_MOESM5_ESM.zip › Figure 3/3H/3H-HEK293T-WB.pptx]

## Slide 1
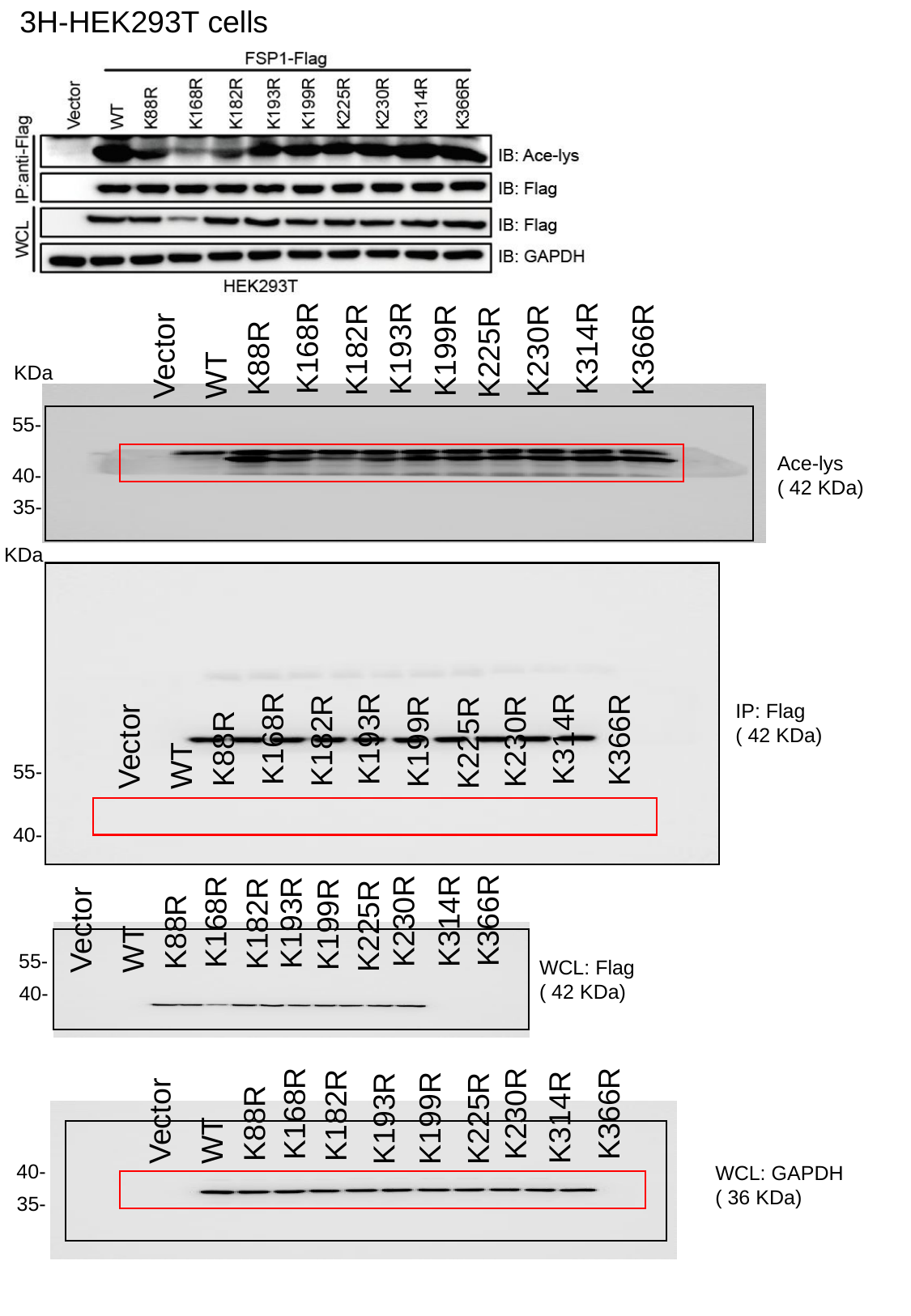

3H-HEK293T cells
K168R
K88R
K182R
Vector
WT
K314R
K366R
K193R
K230R
K225R
K199R
KDa
55-
Ace-lys
( 42 KDa)
40-
35-
KDa
K168R
K88R
K182R
Vector
IP: Flag
( 42 KDa)
WT
K314R
K366R
K193R
K230R
K225R
K199R
55-
K168R
40-
K88R
K182R
Vector
WT
K366R
K230R
K314R
K193R
K225R
K199R
55-
WCL: Flag
( 42 KDa)
40-
K168R
K88R
K182R
Vector
WT
K366R
K230R
K314R
K225R
K193R
K199R
40-
WCL: GAPDH
( 36 KDa)
35-
