# Supplementary material for: SLC25A1 and ACLY maintain cytosolic acetyl-CoA and regulate ferroptosis susceptibility via FSP1 acetylation
Source: EMBO J. 2025 Jan 29;44(6):1641–62. doi: 10.1038/s44318-025-00369-5 (PMC11914110; doi:10.1038/s44318-025-00369-5)
Supplement: Supplementary file 5 — Source data Fig. 3 [file 44318_2025_369_MOESM5_ESM.zip › Figure 3/3I/3I-A375-FSP1-KO-WB.pptx]

## Slide 1
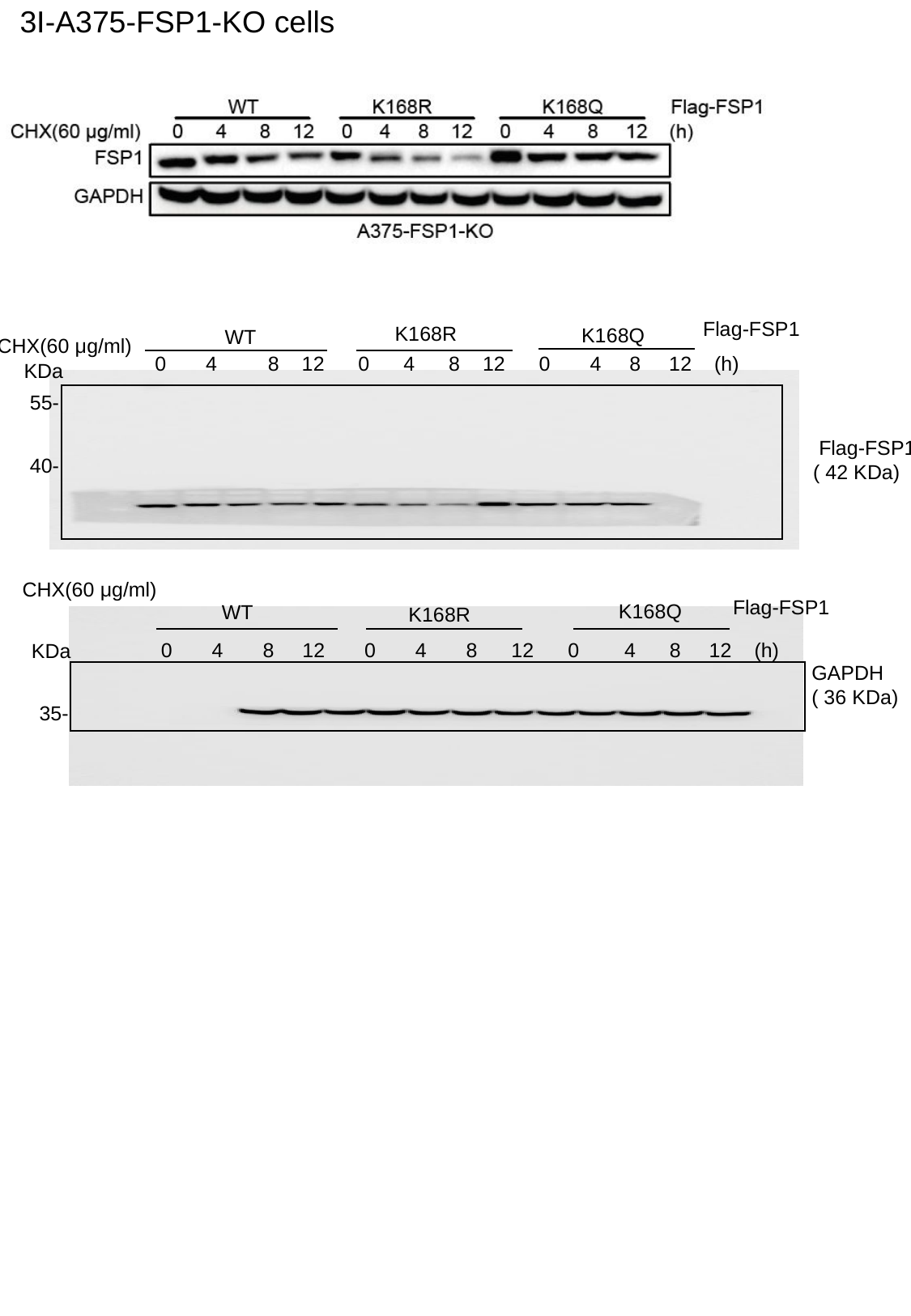

3I-A375-FSP1-KO cells
Flag-FSP1
K168R
K168Q
 WT
CHX(60 μg/ml)
 0 4 8 12 0 4 8 12 0 4 8 12 (h)
KDa
55-
 Flag-FSP1
( 42 KDa)
40-
CHX(60 μg/ml)
Flag-FSP1
K168Q
 WT
K168R
 0 4 8 12 0 4 8 12 0 4 8 12 (h)
KDa
GAPDH
( 36 KDa)
35-
