# Supplementary material for: SLC25A1 and ACLY maintain cytosolic acetyl-CoA and regulate ferroptosis susceptibility via FSP1 acetylation
Source: EMBO J. 2025 Jan 29;44(6):1641–62. doi: 10.1038/s44318-025-00369-5 (PMC11914110; doi:10.1038/s44318-025-00369-5)
Supplement: Supplementary file 5 — Source data Fig. 3 [file 44318_2025_369_MOESM5_ESM.zip › Figure 3/3K/3K-A375-FSP1-KO-WB.pptx]

## Slide 1
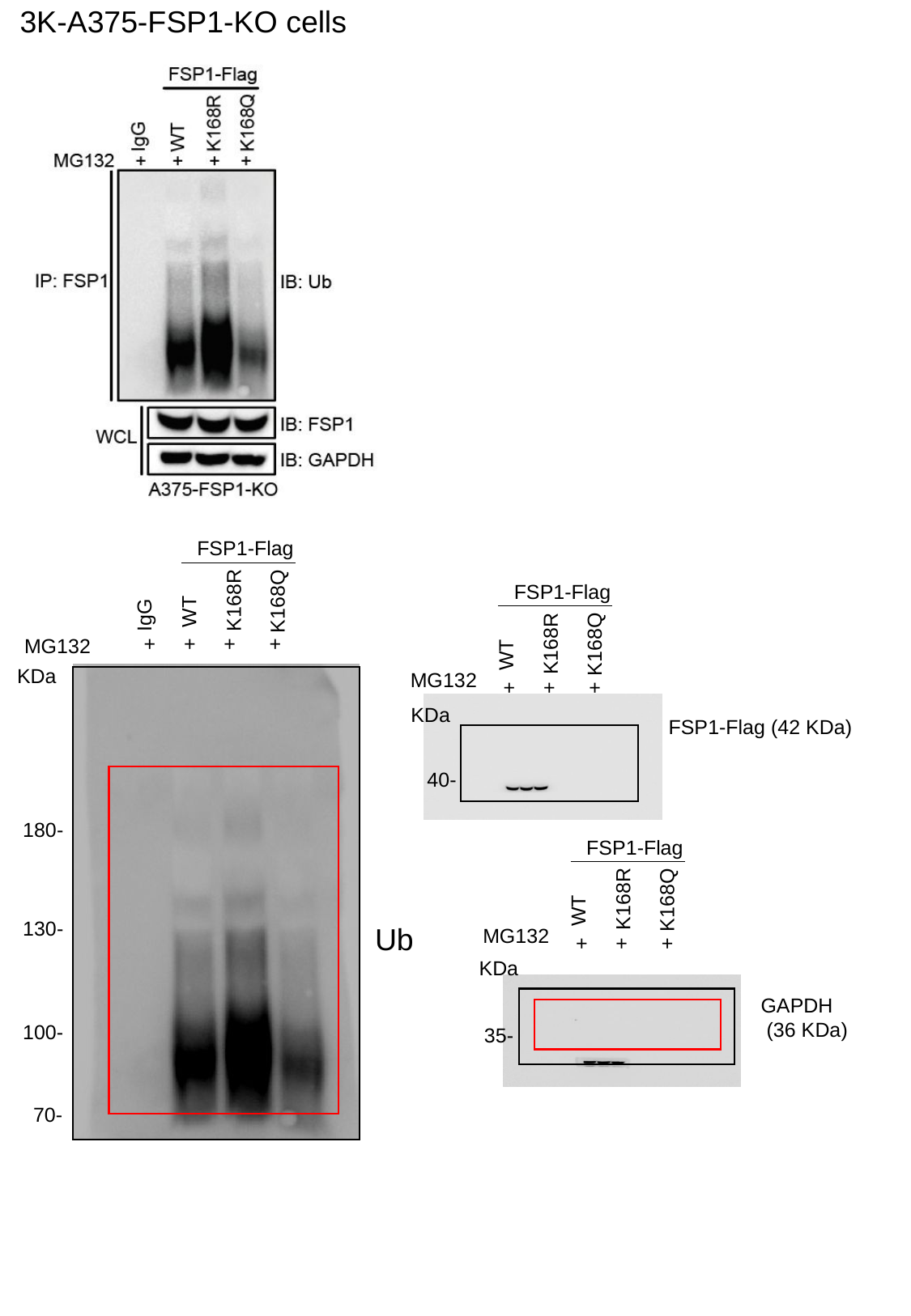

3K-A375-FSP1-KO cells
FSP1-Flag
FSP1-Flag
K168R
K168Q
WT
IgG
K168R
K168Q
 + + + +
MG132
WT
KDa
MG132
 + + +
KDa
FSP1-Flag (42 KDa)
40-
180-
FSP1-Flag
K168R
K168Q
WT
130-
Ub
MG132
 + + +
KDa
GAPDH
 (36 KDa)
100-
35-
70-
