# Supplementary material for: SLC25A1 and ACLY maintain cytosolic acetyl-CoA and regulate ferroptosis susceptibility via FSP1 acetylation
Source: EMBO J. 2025 Jan 29;44(6):1641–62. doi: 10.1038/s44318-025-00369-5 (PMC11914110; doi:10.1038/s44318-025-00369-5)
Supplement: Supplementary file 6 — Source data Fig. 4 [file 44318_2025_369_MOESM6_ESM.zip › Figure 4/4B/4B-HEK293T-WB.pptx]

## Slide 1
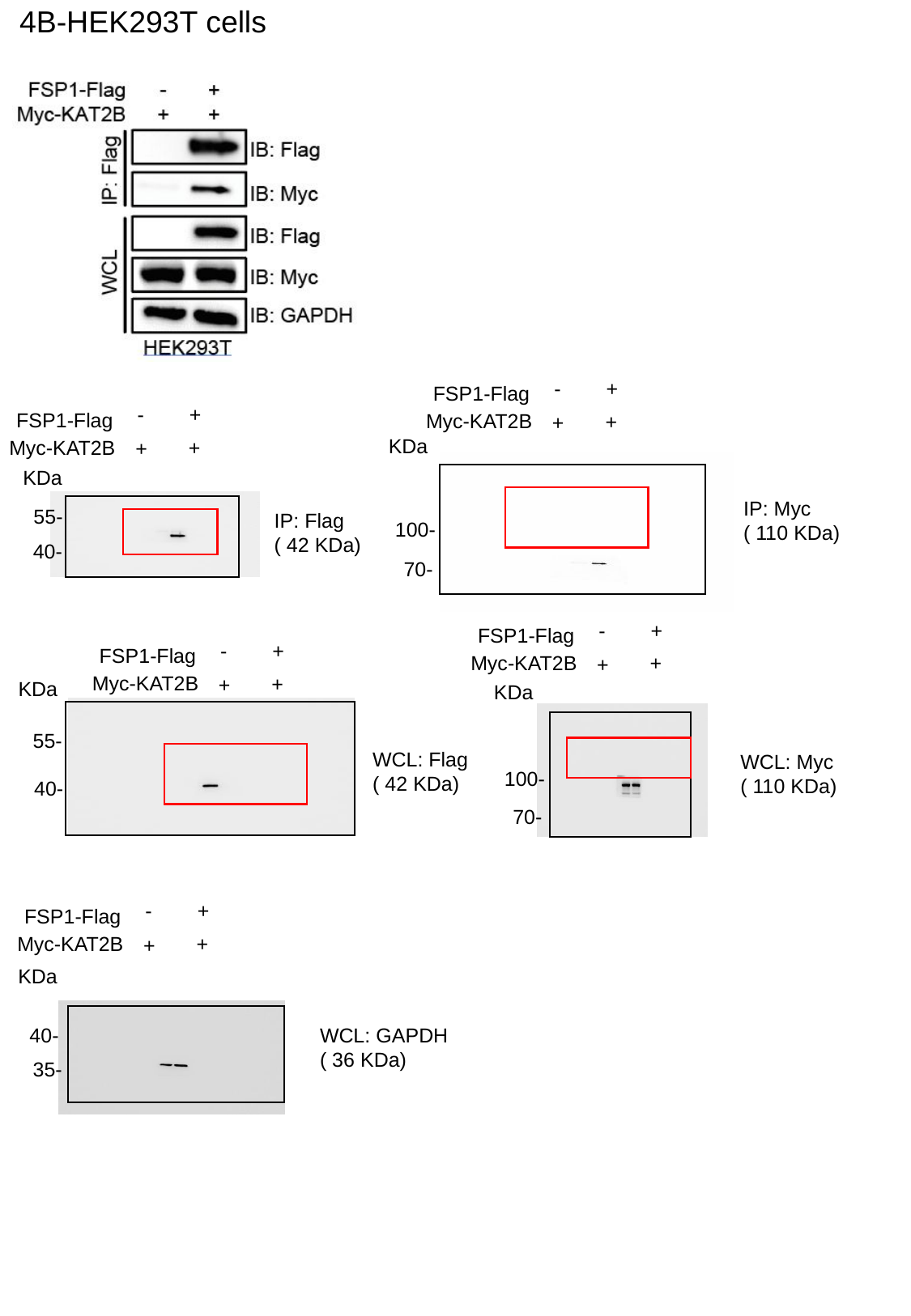

4B-HEK293T cells
-
+
FSP1-Flag
-
+
FSP1-Flag
Myc-KAT2B
+
+
KDa
Myc-KAT2B
+
+
KDa
IP: Myc
( 110 KDa)
55-
IP: Flag
( 42 KDa)
100-
40-
70-
-
+
FSP1-Flag
-
+
FSP1-Flag
Myc-KAT2B
+
+
Myc-KAT2B
+
+
KDa
KDa
55-
WCL: Flag
( 42 KDa)
WCL: Myc
( 110 KDa)
100-
40-
70-
-
+
FSP1-Flag
Myc-KAT2B
+
+
KDa
WCL: GAPDH
( 36 KDa)
40-
35-
