# Supplementary material for: SLC25A1 and ACLY maintain cytosolic acetyl-CoA and regulate ferroptosis susceptibility via FSP1 acetylation
Source: EMBO J. 2025 Jan 29;44(6):1641–62. doi: 10.1038/s44318-025-00369-5 (PMC11914110; doi:10.1038/s44318-025-00369-5)
Supplement: Supplementary file 6 — Source data Fig. 4 [file 44318_2025_369_MOESM6_ESM.zip › Figure 4/4C/4C-A375-WB.pptx]

## Slide 1
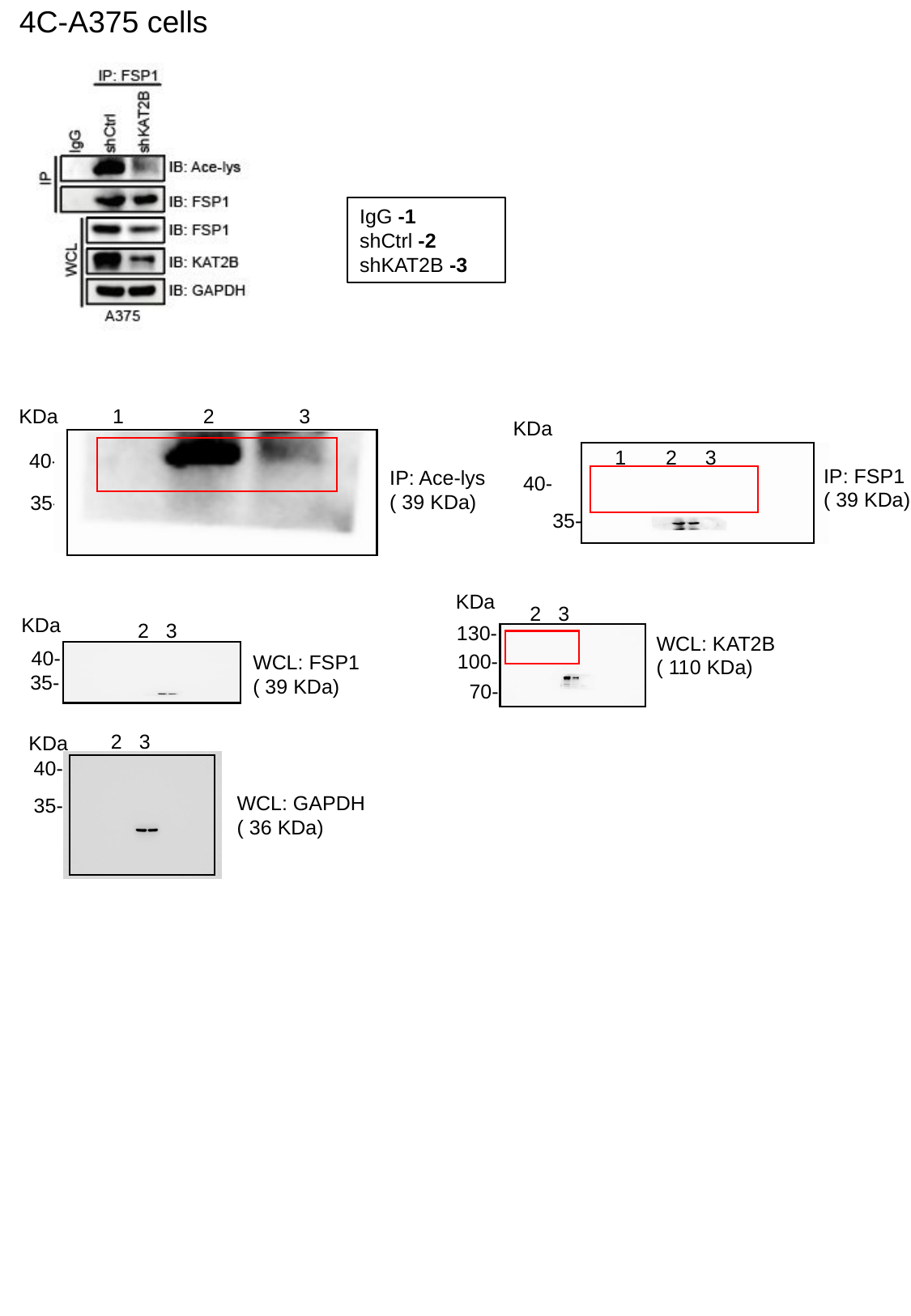

4C-A375 cells
IgG -1
shCtrl -2
shKAT2B -3
KDa
 1 2 3
KDa
 1 2 3
40-
IP: FSP1
( 39 KDa)
IP: Ace-lys
( 39 KDa)
40-
35-
35-
KDa
 2 3
KDa
 2 3
130-
WCL: KAT2B
( 110 KDa)
40-
100-
WCL: FSP1
( 39 KDa)
35-
70-
 2 3
KDa
40-
WCL: GAPDH
( 36 KDa)
35-
