# Supplementary material for: SLC25A1 and ACLY maintain cytosolic acetyl-CoA and regulate ferroptosis susceptibility via FSP1 acetylation
Source: EMBO J. 2025 Jan 29;44(6):1641–62. doi: 10.1038/s44318-025-00369-5 (PMC11914110; doi:10.1038/s44318-025-00369-5)
Supplement: Supplementary file 6 — Source data Fig. 4 [file 44318_2025_369_MOESM6_ESM.zip › Figure 4/4H/4H-HEK293T-WB.pptx]

## Slide 1
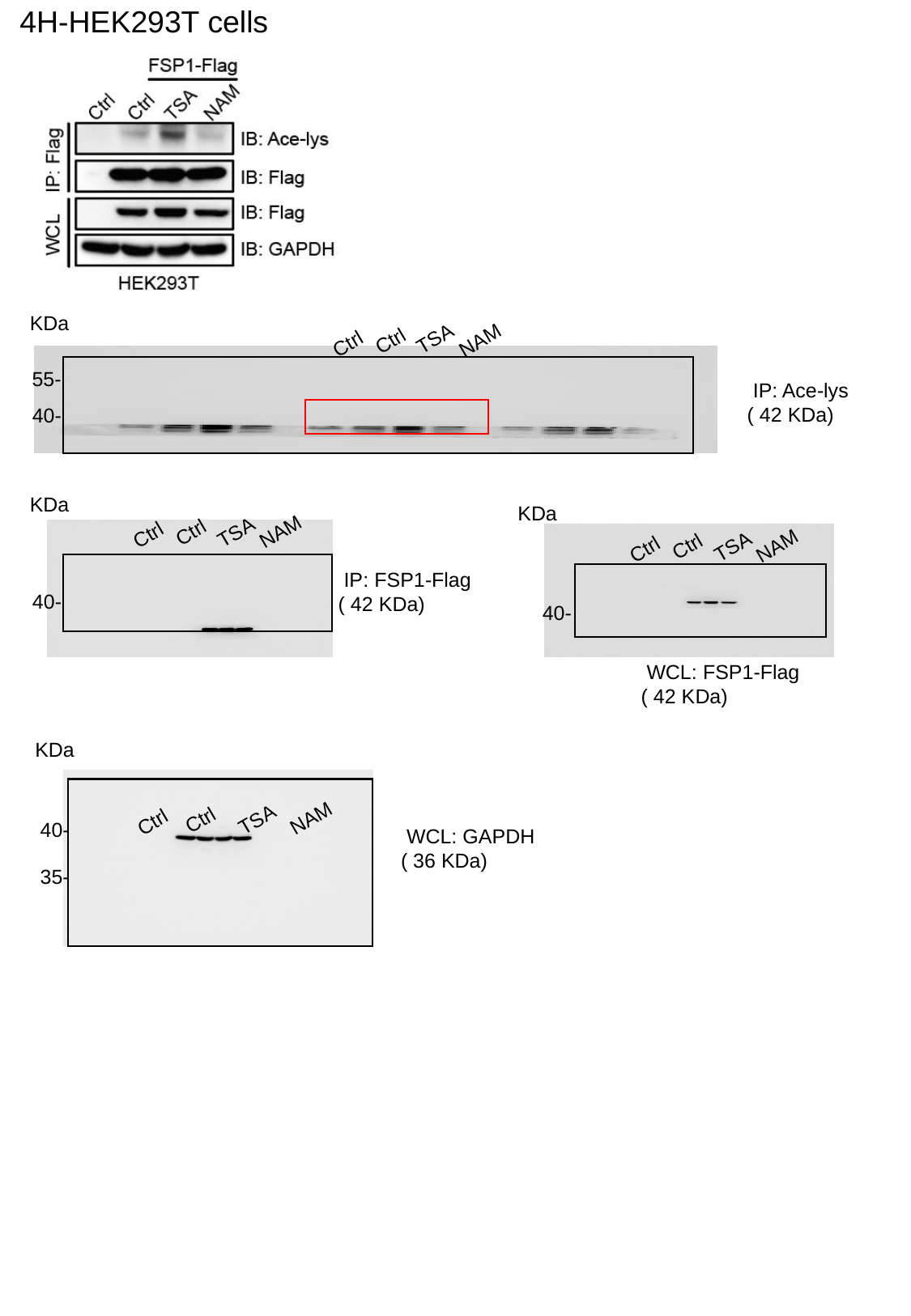

4H-HEK293T cells
KDa
TSA
Ctrl
Ctrl
NAM
55-
 IP: Ace-lys
( 42 KDa)
40-
KDa
KDa
Ctrl
TSA
Ctrl
NAM
Ctrl
TSA
Ctrl
NAM
 IP: FSP1-Flag
( 42 KDa)
40-
40-
 WCL: FSP1-Flag
( 42 KDa)
KDa
Ctrl
NAM
TSA
Ctrl
40-
 WCL: GAPDH
( 36 KDa)
35-
