# Supplementary material for: SLC25A1 and ACLY maintain cytosolic acetyl-CoA and regulate ferroptosis susceptibility via FSP1 acetylation
Source: EMBO J. 2025 Jan 29;44(6):1641–62. doi: 10.1038/s44318-025-00369-5 (PMC11914110; doi:10.1038/s44318-025-00369-5)
Supplement: Supplementary file 6 — Source data Fig. 4 [file 44318_2025_369_MOESM6_ESM.zip › Figure 4/4I/4I-HEK293T-WB.pptx]

## Slide 1
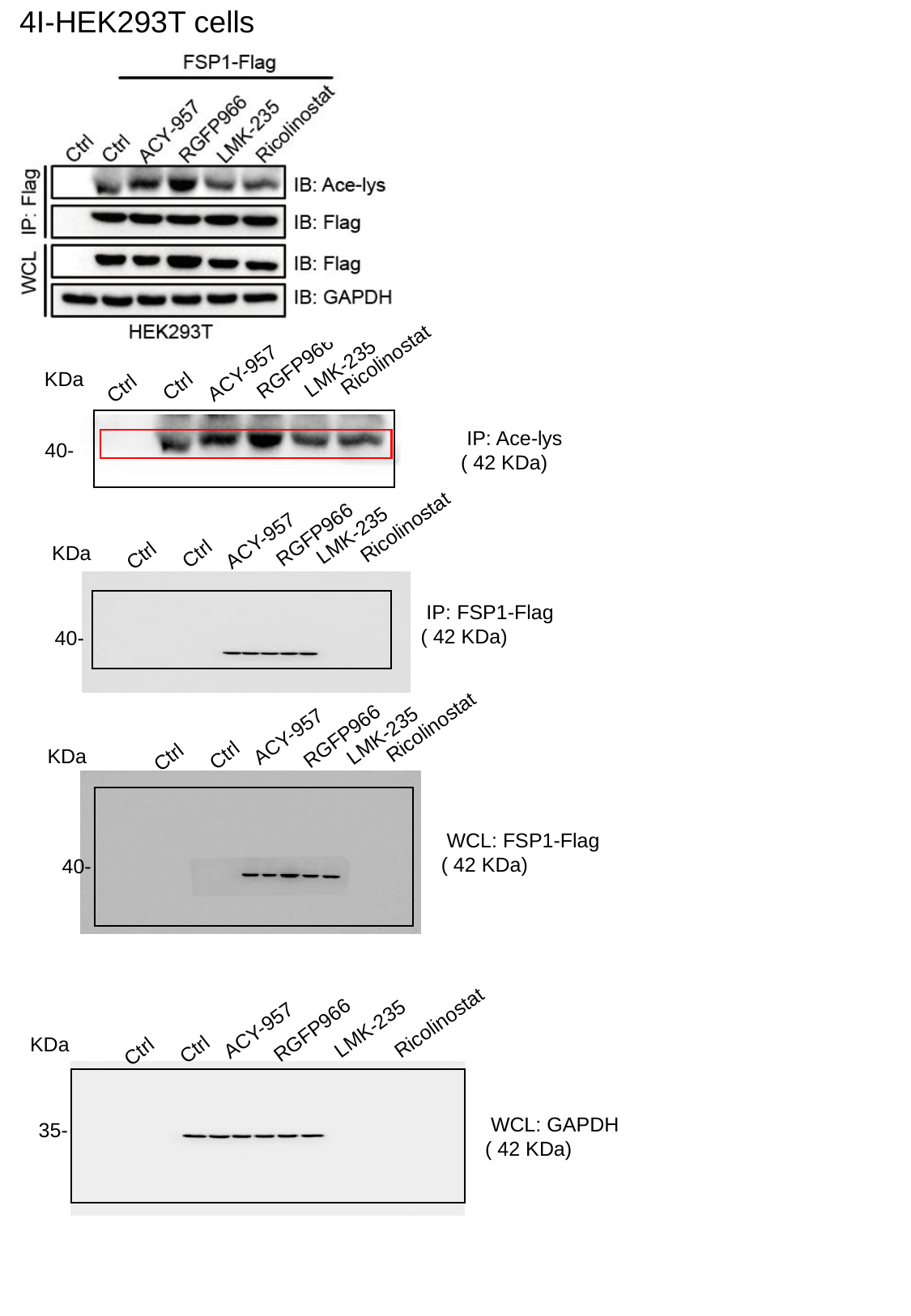

4I-HEK293T cells
LMK-235
ACY-957
Ricolinostat
RGFP966
Ctrl
KDa
Ctrl
 IP: Ace-lys
( 42 KDa)
40-
LMK-235
ACY-957
Ricolinostat
RGFP966
Ctrl
Ctrl
KDa
 IP: FSP1-Flag
( 42 KDa)
40-
LMK-235
ACY-957
Ricolinostat
RGFP966
Ctrl
Ctrl
KDa
 WCL: FSP1-Flag
( 42 KDa)
40-
LMK-235
ACY-957
Ricolinostat
RGFP966
Ctrl
Ctrl
KDa
 WCL: GAPDH
( 42 KDa)
35-
