# Supplementary material for: SLC25A1 and ACLY maintain cytosolic acetyl-CoA and regulate ferroptosis susceptibility via FSP1 acetylation
Source: EMBO J. 2025 Jan 29;44(6):1641–62. doi: 10.1038/s44318-025-00369-5 (PMC11914110; doi:10.1038/s44318-025-00369-5)
Supplement: Supplementary file 6 — Source data Fig. 4 [file 44318_2025_369_MOESM6_ESM.zip › Figure 4/4K/4K-HEK293T-WB.pptx]

## Slide 1
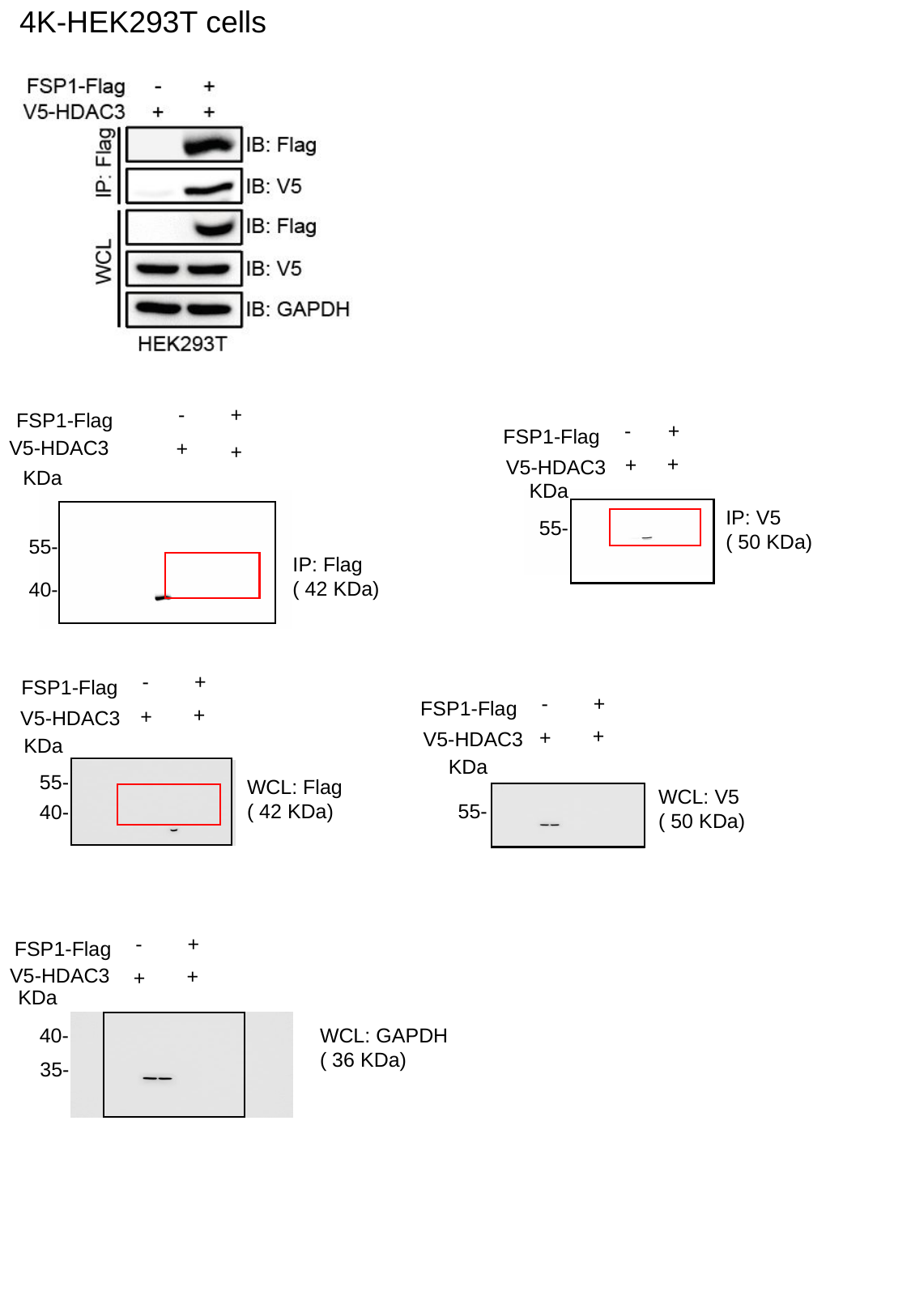

4K-HEK293T cells
-
+
FSP1-Flag
-
+
FSP1-Flag
V5-HDAC3
+
+
+
+
V5-HDAC3
KDa
KDa
IP: V5
( 50 KDa)
55-
55-
IP: Flag
( 42 KDa)
40-
-
+
FSP1-Flag
-
+
FSP1-Flag
+
+
V5-HDAC3
+
+
V5-HDAC3
KDa
KDa
55-
WCL: Flag
( 42 KDa)
WCL: V5
( 50 KDa)
55-
40-
-
+
FSP1-Flag
V5-HDAC3
+
+
KDa
WCL: GAPDH
( 36 KDa)
40-
35-
