# Supplementary material for: SLC25A1 and ACLY maintain cytosolic acetyl-CoA and regulate ferroptosis susceptibility via FSP1 acetylation
Source: EMBO J. 2025 Jan 29;44(6):1641–62. doi: 10.1038/s44318-025-00369-5 (PMC11914110; doi:10.1038/s44318-025-00369-5)
Supplement: Supplementary file 7 — Source data Fig. 5 [file 44318_2025_369_MOESM7_ESM.zip › Figure 5/5A/5A-A375-WB.pptx]

## Slide 1
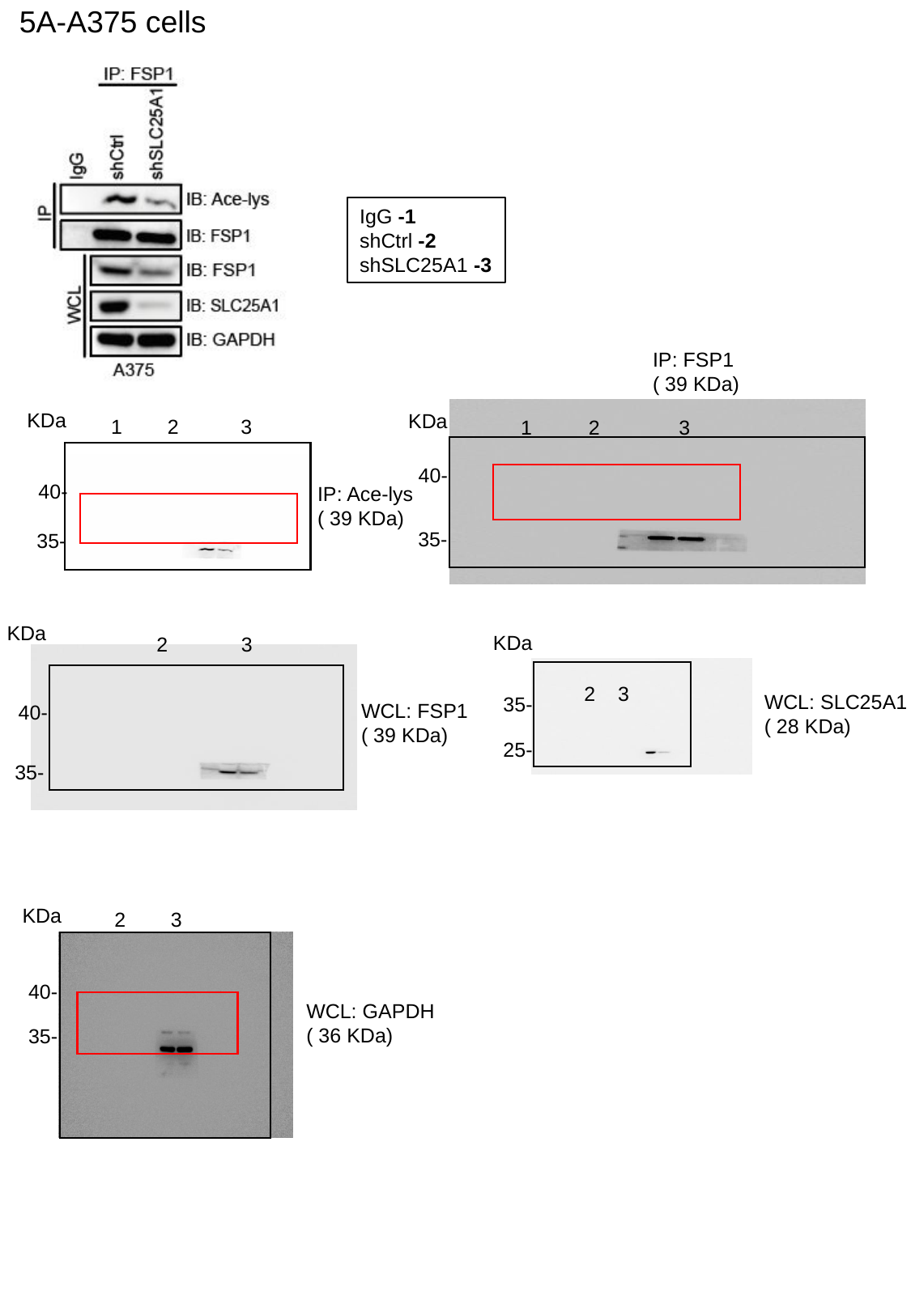

5A-A375 cells
IgG -1
shCtrl -2
shSLC25A1 -3
IP: FSP1
( 39 KDa)
KDa
KDa
 1 2 3
 1 2 3
40-
40-
IP: Ace-lys
( 39 KDa)
35-
35-
KDa
KDa
 2 3
 2 3
WCL: SLC25A1
( 28 KDa)
35-
WCL: FSP1
( 39 KDa)
40-
25-
35-
KDa
 2 3
40-
WCL: GAPDH
( 36 KDa)
35-
