# Supplementary material for: SLC25A1 and ACLY maintain cytosolic acetyl-CoA and regulate ferroptosis susceptibility via FSP1 acetylation
Source: EMBO J. 2025 Jan 29;44(6):1641–62. doi: 10.1038/s44318-025-00369-5 (PMC11914110; doi:10.1038/s44318-025-00369-5)
Supplement: Supplementary file 7 — Source data Fig. 5 [file 44318_2025_369_MOESM7_ESM.zip › Figure 5/5G/5G-A375-WB.pptx]

## Slide 1
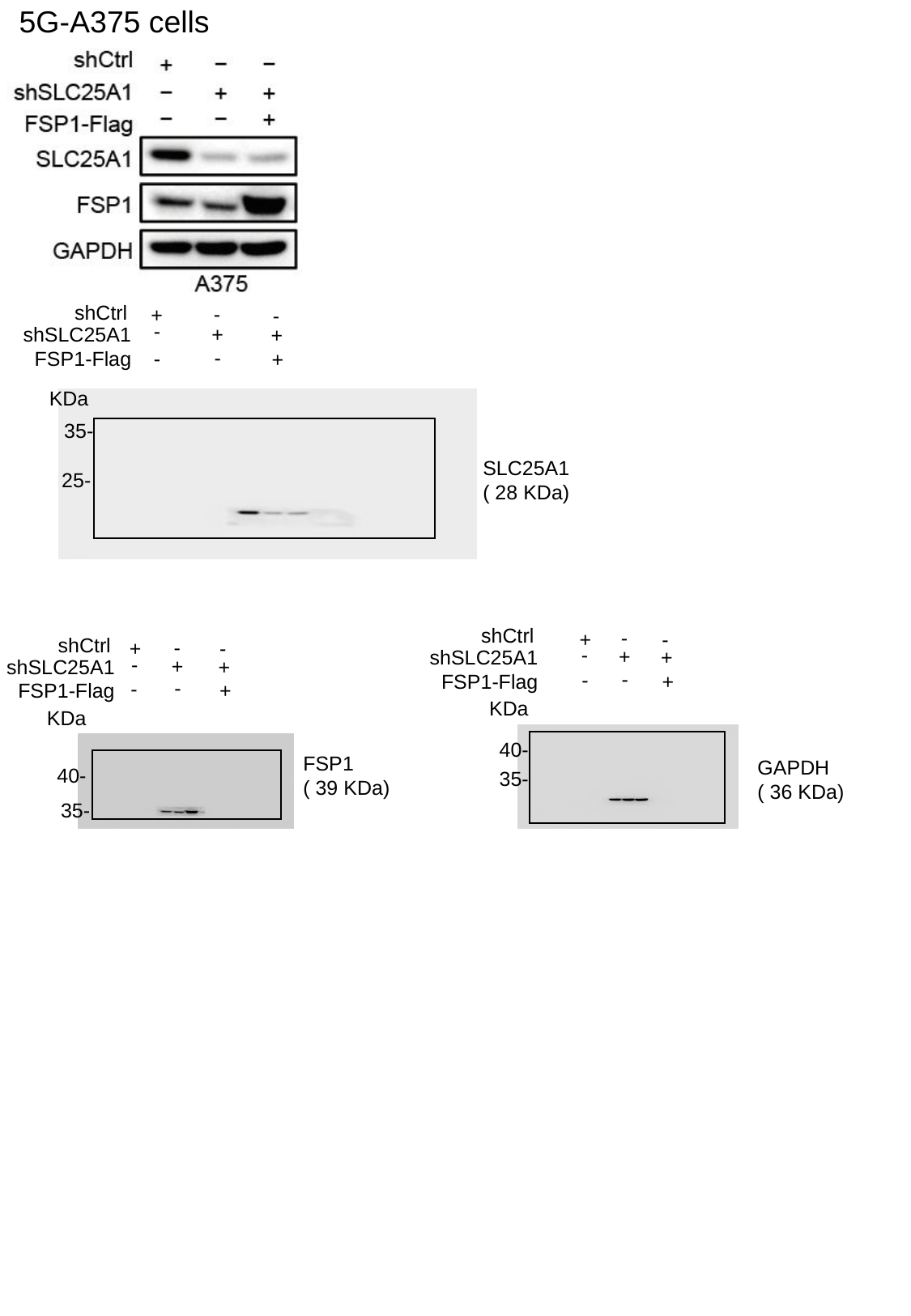

5G-A375 cells
shCtrl
-
+
-
-
shSLC25A1
+
+
-
FSP1-Flag
-
+
KDa
35-
SLC25A1
( 28 KDa)
25-
shCtrl
-
+
-
shCtrl
-
+
-
-
+
shSLC25A1
+
-
+
shSLC25A1
+
-
-
FSP1-Flag
+
-
-
+
FSP1-Flag
KDa
KDa
40-
FSP1
( 39 KDa)
GAPDH
( 36 KDa)
40-
35-
35-
