# Supplementary material for: SLC25A1 and ACLY maintain cytosolic acetyl-CoA and regulate ferroptosis susceptibility via FSP1 acetylation
Source: EMBO J. 2025 Jan 29;44(6):1641–62. doi: 10.1038/s44318-025-00369-5 (PMC11914110; doi:10.1038/s44318-025-00369-5)
Supplement: Supplementary file 7 — Source data Fig. 5 [file 44318_2025_369_MOESM7_ESM.zip › Figure 5/5K/5K-A375-WB.pptx]

## Slide 1
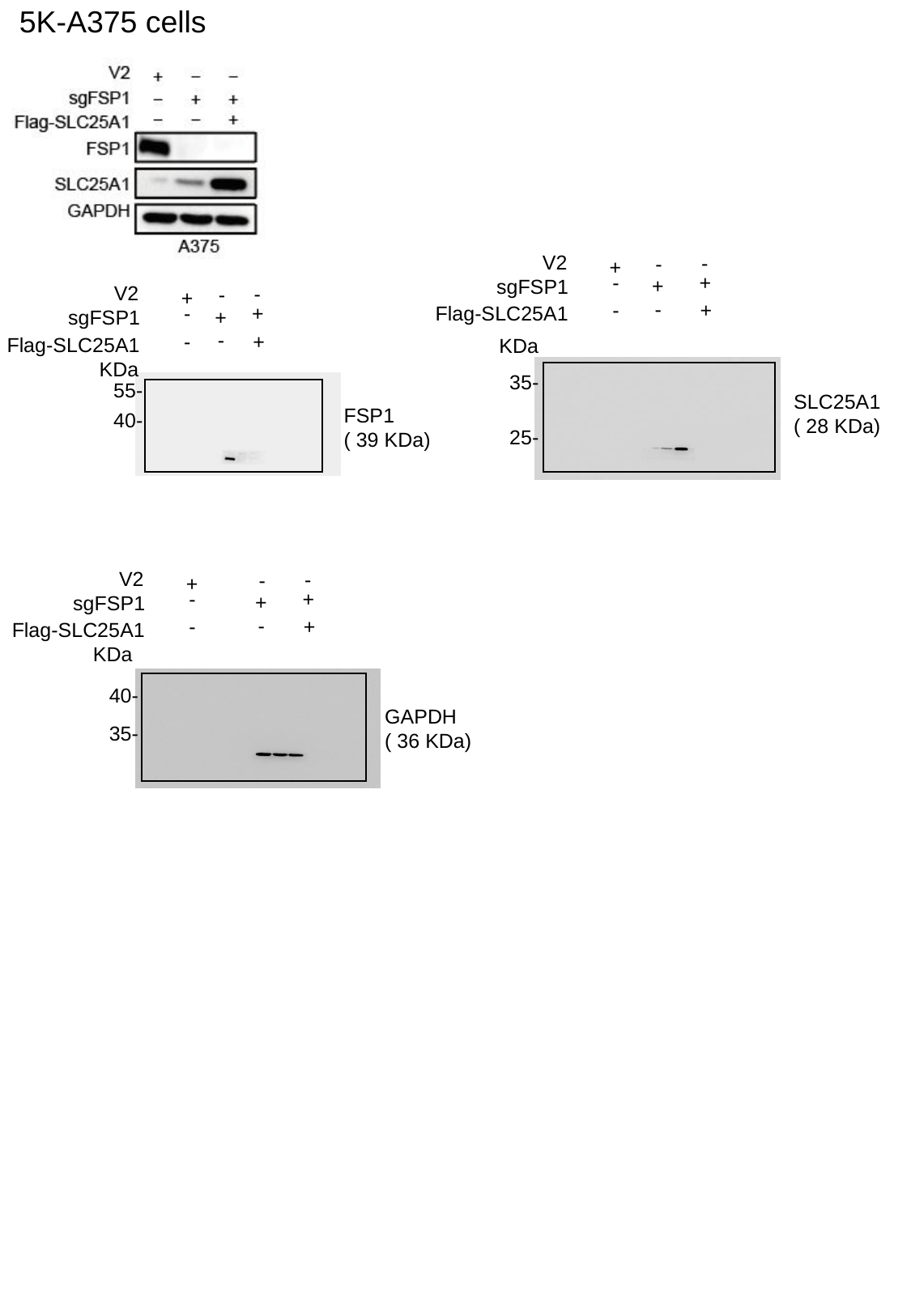

5K-A375 cells
V2
-
-
+
-
+
+
sgFSP1
V2
-
-
+
-
+
-
-
+
Flag-SLC25A1
+
sgFSP1
-
+
-
Flag-SLC25A1
KDa
KDa
35-
55-
SLC25A1
( 28 KDa)
FSP1
( 39 KDa)
40-
25-
V2
-
-
+
-
+
+
sgFSP1
-
+
-
Flag-SLC25A1
KDa
40-
GAPDH
( 36 KDa)
35-
