# Supplementary material for: SLC25A1 and ACLY maintain cytosolic acetyl-CoA and regulate ferroptosis susceptibility via FSP1 acetylation
Source: EMBO J. 2025 Jan 29;44(6):1641–62. doi: 10.1038/s44318-025-00369-5 (PMC11914110; doi:10.1038/s44318-025-00369-5)
Supplement: Supplementary file 8 — Source data Fig. 6 [file 44318_2025_369_MOESM8_ESM.zip › Figure 6/6E/6E-A375-A549-WB.pptx]

## Slide 1
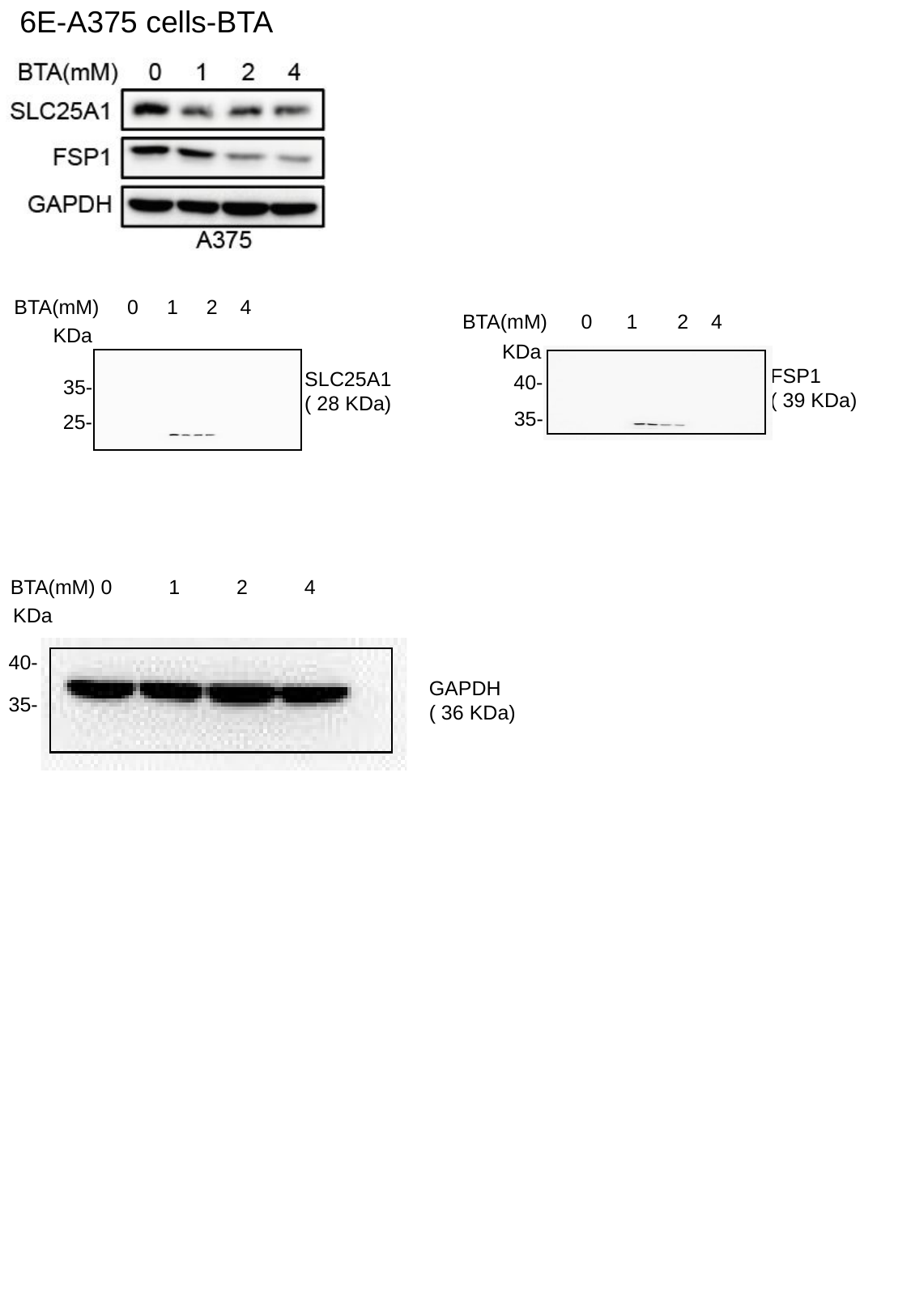

6E-A375 cells-BTA
BTA(mM) 0 1 2 4
BTA(mM) 0 1 2 4
KDa
KDa
FSP1
( 39 KDa)
SLC25A1
( 28 KDa)
40-
35-
35-
25-
BTA(mM) 0 1 2 4
KDa
40-
GAPDH
( 36 KDa)
35-

## Slide 2
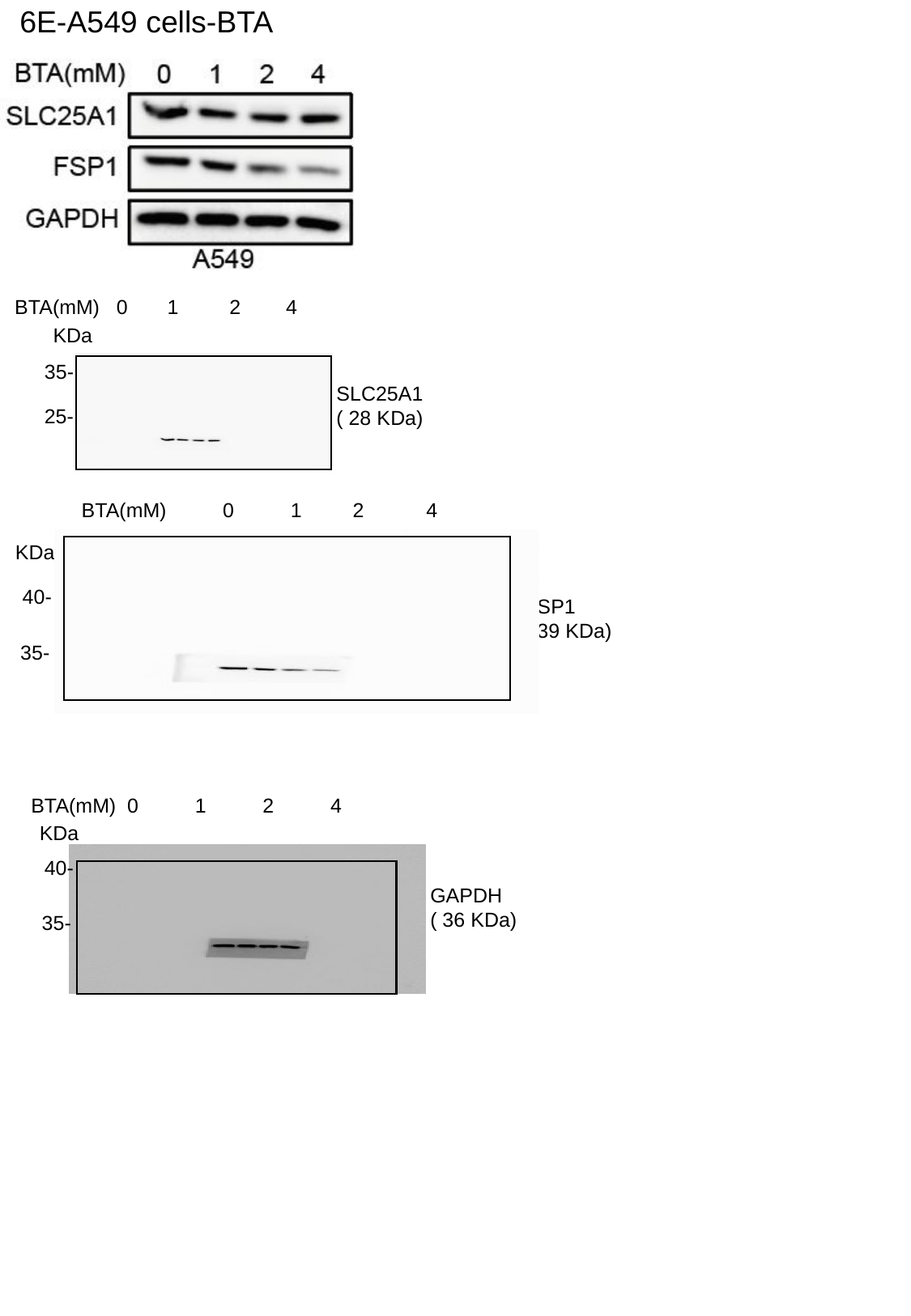

6E-A549 cells-BTA
BTA(mM) 0 1 2 4
KDa
35-
SLC25A1
( 28 KDa)
25-
BTA(mM) 0 1 2 4
KDa
40-
FSP1
( 39 KDa)
35-
BTA(mM) 0 1 2 4
KDa
40-
GAPDH
( 36 KDa)
35-

## Slide 3
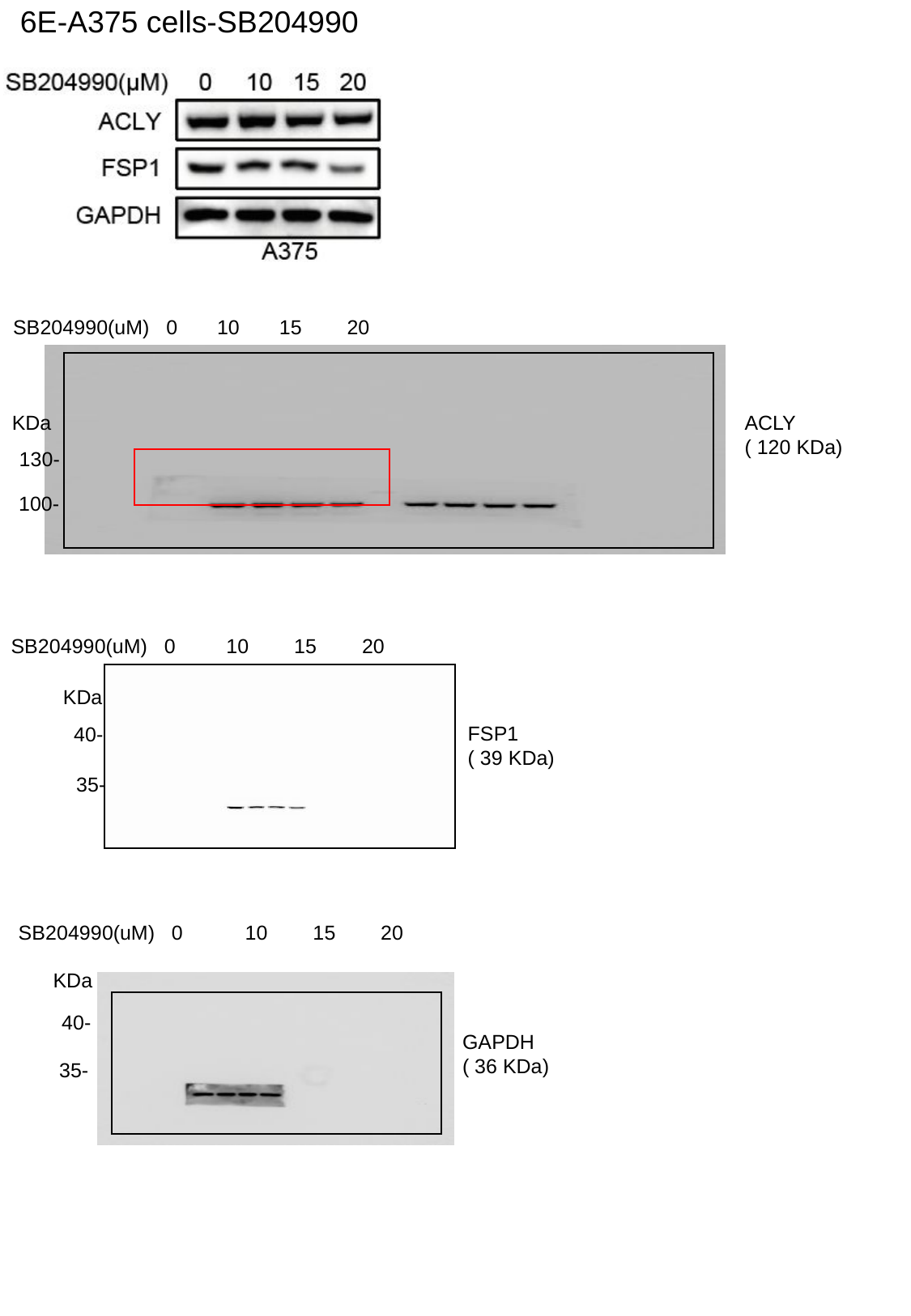

6E-A375 cells-SB204990
SB204990(uM) 0 10 15 20
KDa
ACLY
( 120 KDa)
130-
100-
SB204990(uM) 0 10 15 20
KDa
FSP1
( 39 KDa)
40-
35-
SB204990(uM) 0 10 15 20
KDa
40-
GAPDH
( 36 KDa)
35-

## Slide 4
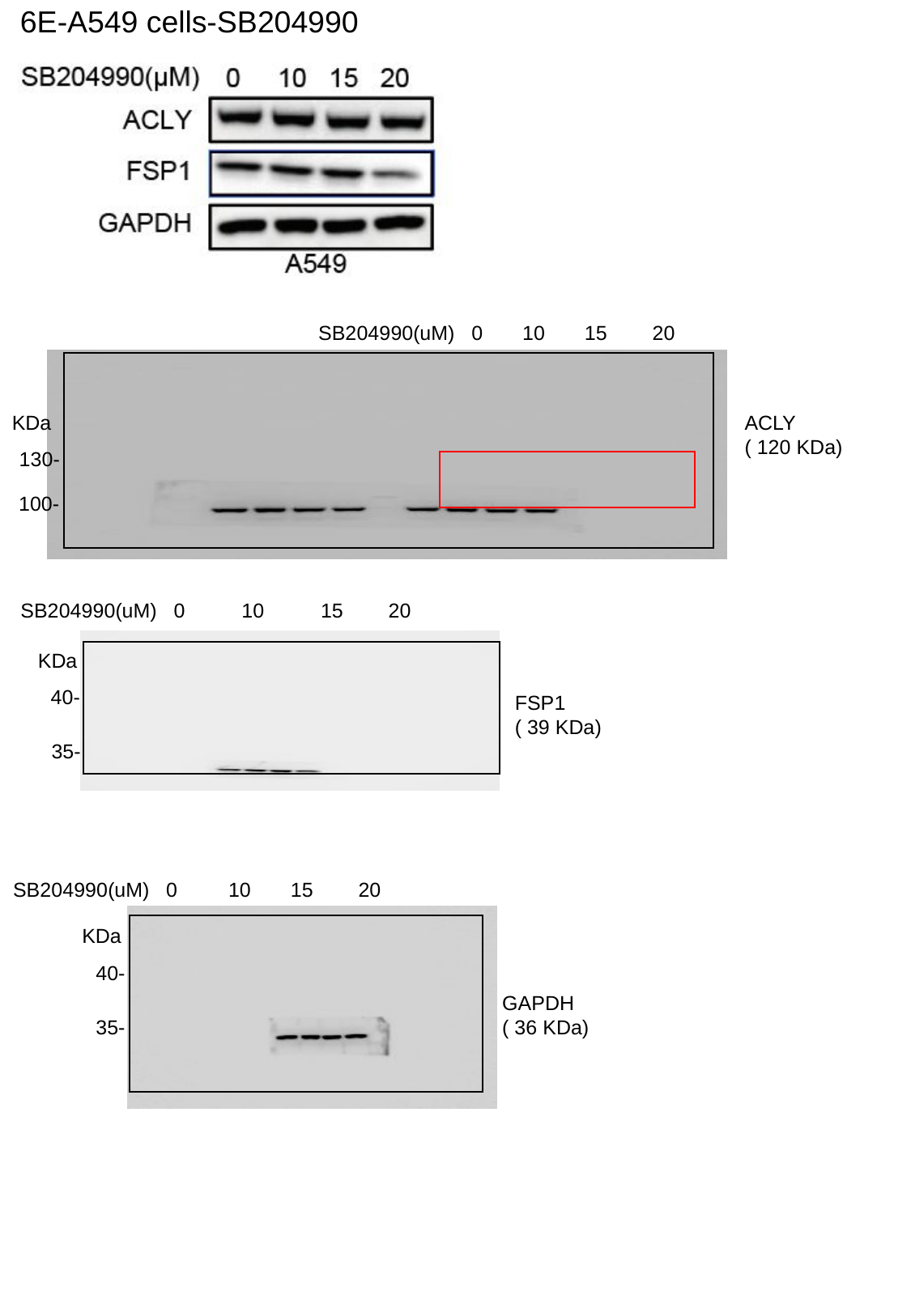

6E-A549 cells-SB204990
SB204990(uM) 0 10 15 20
KDa
ACLY
( 120 KDa)
130-
100-
SB204990(uM) 0 10 15 20
KDa
40-
FSP1
( 39 KDa)
35-
SB204990(uM) 0 10 15 20
KDa
40-
GAPDH
( 36 KDa)
35-
