# Supplementary material for: SLC25A1 and ACLY maintain cytosolic acetyl-CoA and regulate ferroptosis susceptibility via FSP1 acetylation
Source: EMBO J. 2025 Jan 29;44(6):1641–62. doi: 10.1038/s44318-025-00369-5 (PMC11914110; doi:10.1038/s44318-025-00369-5)
Supplement: Supplementary file 8 — Source data Fig. 6 [file 44318_2025_369_MOESM8_ESM.zip › Figure 6/6F/6F-A375-A549-WB.pptx]

## Slide 1
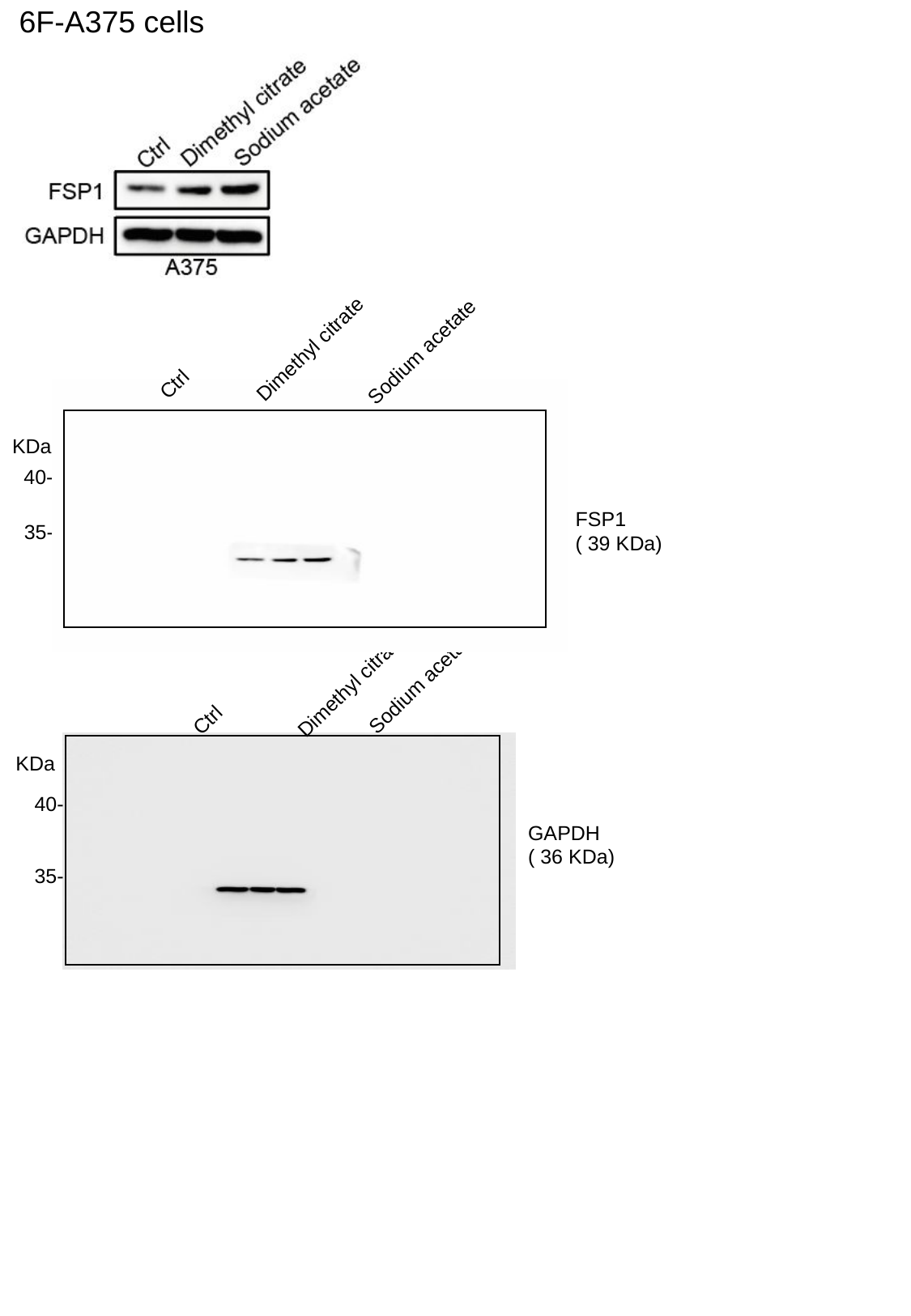

6F-A375 cells
Dimethyl citrate
Sodium acetate
Ctrl
KDa
40-
FSP1
( 39 KDa)
35-
Sodium acetate
Dimethyl citrate
Ctrl
KDa
40-
GAPDH
( 36 KDa)
35-

## Slide 2
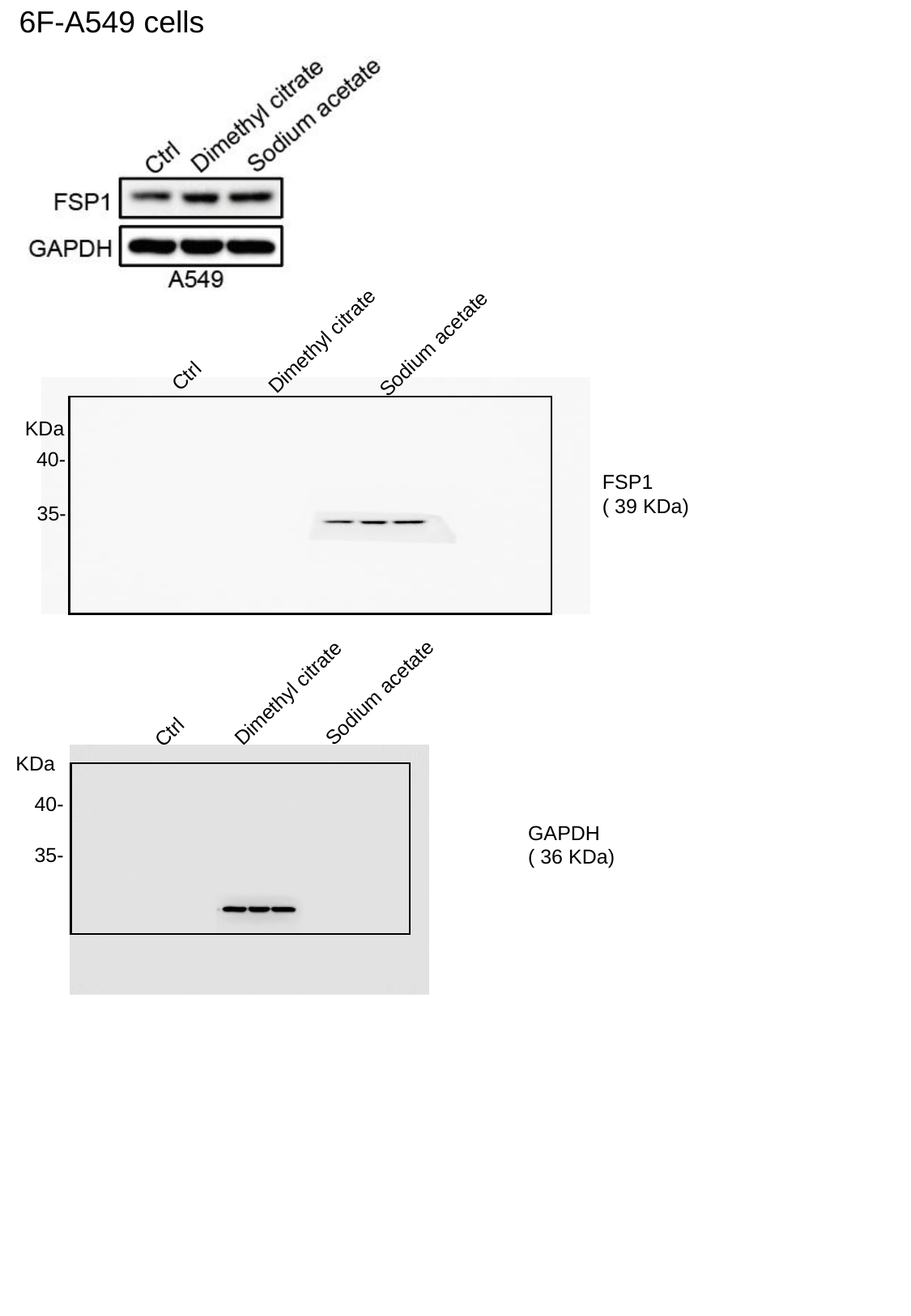

6F-A549 cells
Dimethyl citrate
Sodium acetate
Ctrl
KDa
40-
FSP1
( 39 KDa)
35-
Sodium acetate
Dimethyl citrate
Ctrl
KDa
40-
GAPDH
( 36 KDa)
35-
